# Immune cell-derived extracellular vesicular microRNAs induce pancreatic beta cell apoptosis

**DOI:** 10.1016/j.heliyon.2022.e11995

**Published:** 2022-12-02

**Authors:** Yueyang Yu, Mengyin Li, Yuxuan Zhao, Fangzhou Fan, Wenxiang Wu, Yuhua Gao, Chunyu Bai

**Affiliations:** a Institute of Precision Medicine, Jining Medical University, Jining, Shandong 272067, PR China; b Department of Otorhinolaryngology Head and Neck Surgery, Affiliated Hospital of Jining Medical University, Jining, Shandong Province, 272067, PR China

**Keywords:** Type 1 diabetes mellitus, Immune cells, Extracellular vesicles, miRNA, β cells, Apoptosis

## Abstract

**Background:**

Type 1 diabetes mellitus (T1DM) is an autoimmune disease caused by an autoimmune response against pancreatic islet β cells. Increasing evidence indicates that specific microRNAs (miRNAs) from immune cells extracellular vesicles are involved in islet β cells apoptosis.

**Methods:**

In this study, the microarray datasets GSE27997 and GSE137637 were downloaded from the Gene Expression Omnibus (GEO) database. miRNAs that promote islet β cells apoptosis in T1DM were searched in PubMed. We used the FunRich tool to determine the miRNA expression in extracellular vesicles derived from immune cells associated with islet β cell apoptosis, of which we selected candidate miRNAs based on fold change expression. Potential upstream transcription factors and downstream target genes of candidate miRNAs were predicted using TransmiR V2.0 and starBase database, respectively.

**Results:**

Candidate miRNAs expressed in extracellular vesicles derived from T cells, pro-inflammatory macrophages, B cells, and dendritic cells were analyzed to identify the miRNAs involved in β cells apoptosis. Based on these candidate miRNAs, 25 downstream candidate genes, which positively regulate β cell functions, were predicted and screened; 17 transcription factors that positively regulate the candidate miRNAs were also identified.

**Conclusions:**

Our study demonstrated that immune cell-derived extracellular vesicular miRNAs could promote islet β cell dysfunction and apoptosis. Based on these findings, we have constructed a transcription factor-miRNA-gene regulatory network, which provides a theoretical basis for clinical management of T1DM. This study provides novel insights into the mechanism underlying immune cell-derived extracellular vesicle-mediated islet β cell apoptosis.

## Graphical abstract

**Figure:**
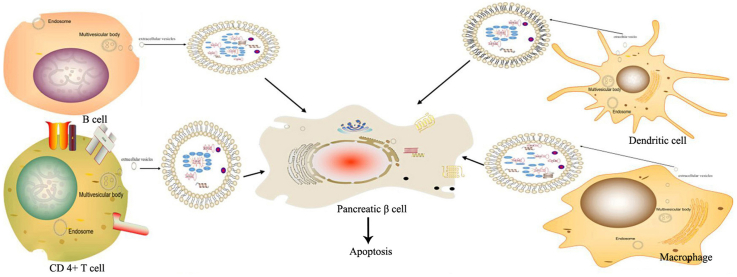

## 1 Introduction

Type 1 diabetes mellitus (T1DM) is an autoimmune disease attributed to the interaction between genetic and environmental factors such as enterovirus (EV) infection. Recent studies have reported a link between EVs, such as the rotavirus (RV), and the coxsackievirus B (CVB) family and the development of T1DM [1, 2]. EV infection triggers an autoimmune response via inflammatory mediators and other factors; consequently, immune cells such as CD8^+^ T lymphocytes attack pancreatic β-cells, thereby decreasing their number [3] and impairing islet function, ultimately resulting in insufficient insulin secretion [4].

During the early stages of T1DM, due to the presence of autoantigens on the surface of β-cells, immune cells, especially T lymphocytes and pro-inflammatory macrophages, infiltrate the islet and release pro-inflammatory factors such as interleukin (IL)-β, tumor necrosis factor α (TNFα), and interferon gamma (IFN-γ). These pro-inflammatory cytokines bind to the corresponding β-cell receptors, thereby causing β-cells to release a series of chemokines, which in turn recruit more immune cells to the islet, thus forming a vicious cycle that eventually leads to β-cell death via apoptosis, ultimately resulting in insulin deficiency [3, 5]. To date, there have been no effective treatment strategies to reverse T1DM, and patients require life-long insulin treatment to maintain normal blood glucose levels [6].

Immune cells can release extracellular vesicles during an immune response [7, 8]. Extracellular vesicles, approximately 50–150 nm in diameter and originate from the late endoplasmic reticulum pathway, are secreted into the extracellular space during the fusion of multivesicular bodies with the plasma membrane [7]. Released extracellular vesicles can transport proteins, lipids, mRNAs, and non-coding RNAs from parent to recipient cells, thereby regulating the biological functions of recipient cells [8, 9, 10, 11, 12]. In T1DM, extracellular vesicles released from immune cells are transferred to islet β-cells where they activate intracellular signaling pathways, causing the release of cytokines and chemokines [8]; extracellular vesicles also promote the formation of immune complexes and immune cell-mediated islet β-cell damage. These findings suggest that extracellular vesicles may contribute to the progression of T1DM and are therefore potential early diagnostic markers and effective therapeutic targets.

MicroRNAs (miRNAs) are non-coding single-stranded RNA molecules of 18–25 nucleotides in length and contain some of the main extracellular vesicle components [9]. miRNAs are important regulators of post-transcriptional gene expression and inactivate their target mRNAs by binding to the 3′ UTR region. miRNAs play an important role in β-cell functions, such as proliferation, development, differentiation, functional maturation, and the regulation of insulin secretion [13, 14]. Guay et al. have reported that T lymphocytes release specific miRNAs-containing extracellular vesicles, which bind to islet β-cells and induce the release of chemokines, thereby exacerbating immune system-mediated islet β-cell attack [8].

Although several studies have evaluated the role of miRNAs in promoting islet β-cell apoptosis, limited number of studies have assessed the role of immune cell-derived extracellular vesicular miRNAs in islet β-cell apoptosis. Herein, we construct a transcription factor-miRNA-gene regulatory network in T1DM to provide potential targets for T1DM diagnosis and treatment.

## 2 Experimental materials and methods

### 2.1 Experimental design

**Figure 1:**
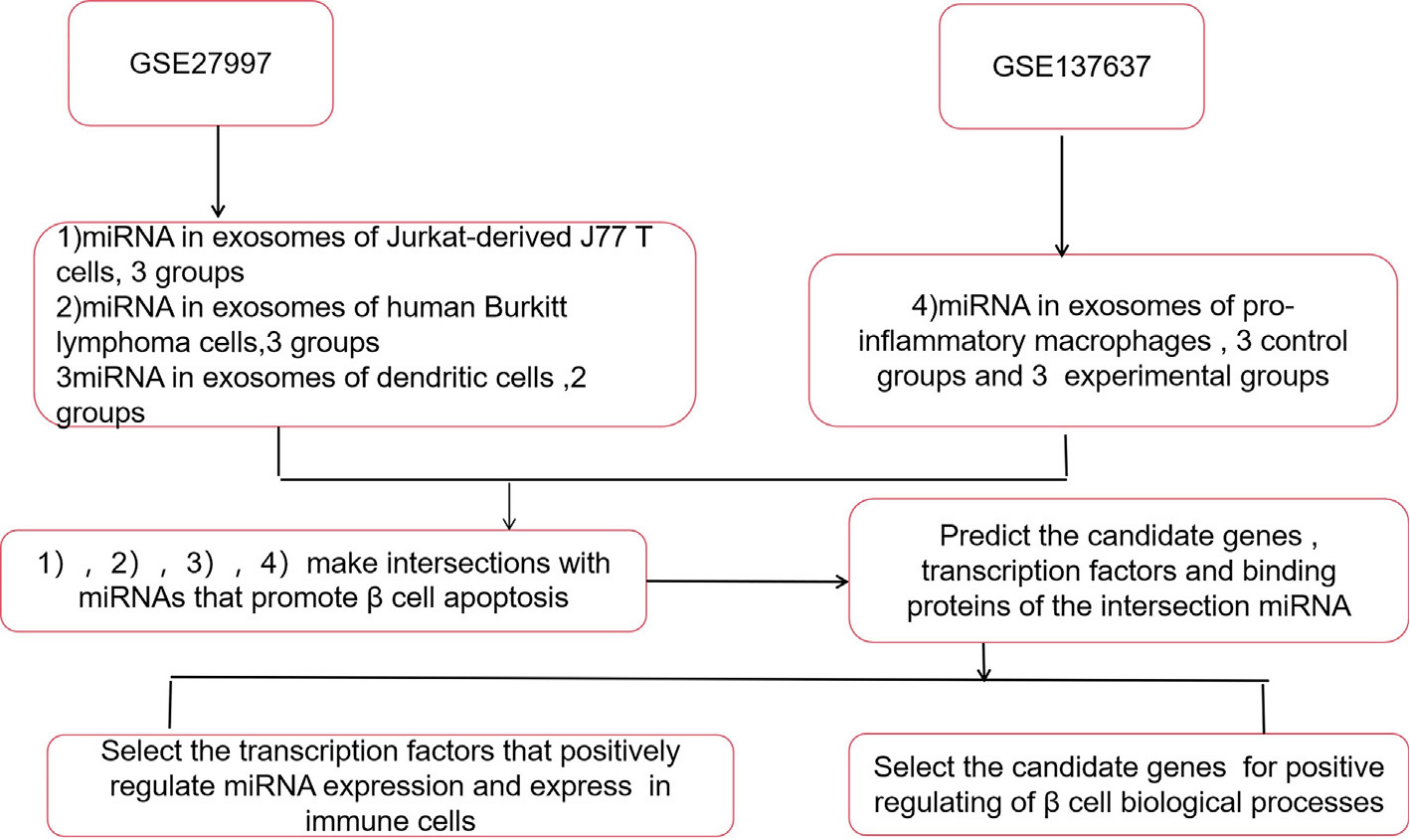
Flow diagram showing process the underlying identification of β cell apoptosis-inducing immune cell-derived extracellular vesicular miRNAs.

We chronologically searched the GEO database using the keywords, “lymphocyte,” “Exosome,” “miRNA,” and “macrophage.” The research type used was “matrix analysis of non-coding RNA,” and the tissue type was “Homo sapiens; ” the sample source was “extracellular vesicles.” We obtained two qualified datasets, GSE27997 and GSE137637. Using the GSE27997 dataset, the miRNA expression profiles of extracellular vesicles derived from T-, B-, and dendritic cells were analyzed. Conversely, using the GSE137637 dataset, the miRNA expression profiles of extracellular vesicles derived from pro-inflammatory macrophages following treatment with *Treponema pallidum* (experimental group) or not (control group) were analyzed. The fold change difference in expression of these miRNAs was >|1.5|. Subsequently, using Venn diagrams in the FunRich software [15], we generated intersections between miRNAs expressed in immune cell-derived extracellular vesicles and 17 miRNAs that reportedly promote β cell apoptosis; the latter were obtained from PubMed using the keywords, “microRNA,” “Diabetes,” and “apoptosis.” We identified miRNAs derived from T lymphocytes, B lymphocytes, dendritic cells, and pro-inflammatory macrophages, and selected candidate miRNAs based on fold change difference in their expression by selecting those with significant differential expression; the transcription factors and candidate genes of these miRNAs were predicted using the MISIM V2.0 [16] and starBase [17] software packages, respectively. Finally, we conducted an extensive literature search in PubMed and identified transcription factors (TFs) expressed in immune cells, positively regulated candidate miRNAs, and candidate genes that positively regulate biological functions in islet β-cells. The flow diagram illustrated in Figure 1 depicts the study design.

### 2.2 Data collection

We searched the GEO database (https://www.ncbi.nlm.nih.gov/gds/term=) using the keywords, “lymphocyte,” “exosome or Extracellular vesicle,” “miRNA,” and “macrophage”. The research type used was “Matrix analysis of non-coding RNA,” the tissue type was “Homo sapiens,” and the sample source was “exosome.” Finally, two datasets (GSE27997 and GSE137637) were obtained for subsequent data analysis. Detailed information on these datasets is listed in Table 1. The expression spectrum is provided in supplementary Tables S1–S4.

**Table 1:** Detailed information on the GSE27997 and GSE137637 datasets.

Accession	Platform	Cell line	Tissue type
GSE27997	GPL8227	The Jurkat-derived J77 T cell line; The Raji B cell line; primary dendritic cells	Homo sapiens
GSE137637	GPL25134	THP-1	Homo sapiens

### 2.3 miRNAs present in immune cell-derived extracellular vesicles promote islet β-cell apoptosis

**Figure 2:**
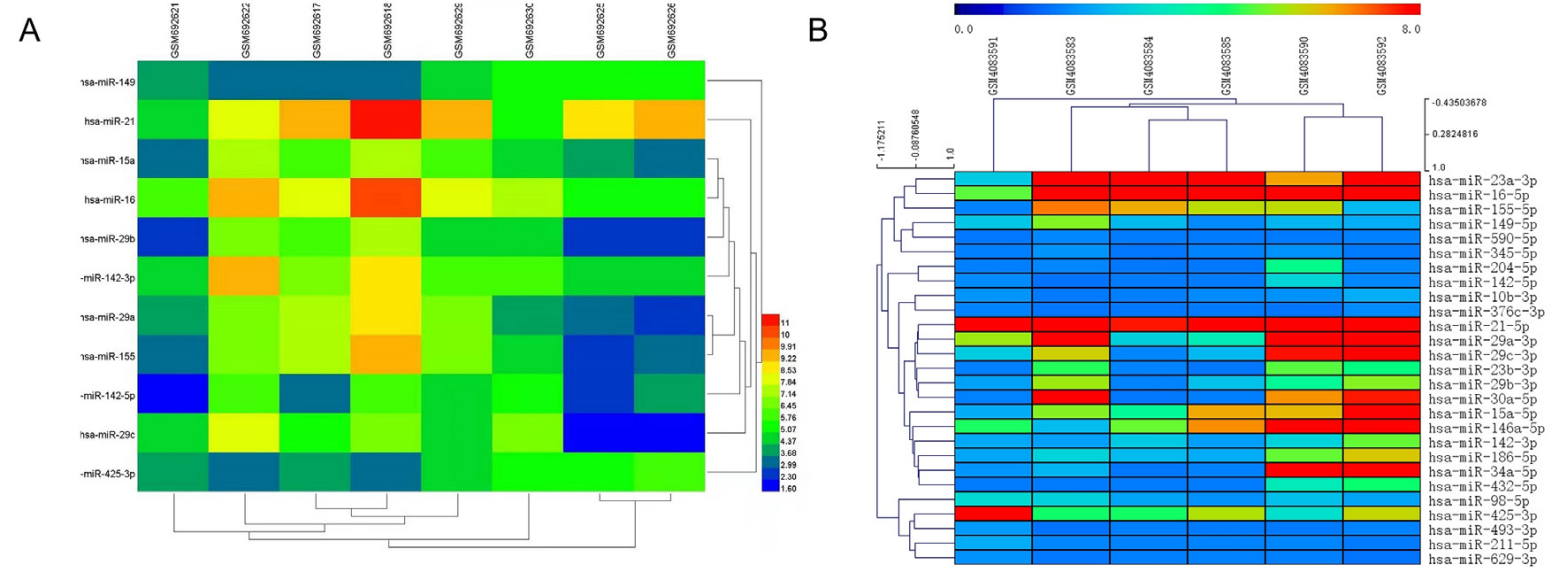
Heatmap of the candidate miRNAs. A. Heatmap of candidate miRNAs in T lymphocytes (GSM692621, GSM692622, and GSM692630), B lymphocytes (GSM692617, GSM692618, and GSM692629), and dendritic cells (GSM692625 and GSM692626); B. Heatmap of candidate miRNAs in macrophages; GSM4083583, GSM4083584, and GSM4083585 represent the control groups, while GSM4083590, GSM4083591, and GSM4083592 represent the experimental groups.

To identify the miRNAs involved in islet β-cell apoptosis, we conducted an extensive literature search in PubMed using the keywords, “microRNA,” “Diabetes,” and “apoptosis,” and selected 17 miRNAs that promote islet β-cell apoptosis during T1DM. FunRich software was used to analyze the intersection of these 17 miRNAs with miRNAs identified in extracellular vesicles derived from T cells, pro-inflammatory macrophages, dendritic cells, and B cells. The heatmap of miRNAs present at the insertion that were selected based on fold change difference in their expression level are shown in Figure 2A and B. Candidate miRNAs with significantly different expression levels were selected. To further clarify the regulatory relationship among candidate miRNAs, MISIM V2.0 was used to construct an miRNA-miRNA interaction network, and the sequences of each candidate miRNA were searched in the miRbase database. Based on the sequences obtained, miRNA-binding proteins were searched using the RBPDP analytical tool [18]. Furthermore, proteins in extracellular vesicles secreted by immune cells were confirmed using the EVpedia database [19]. Finally, miRNAs that stably existed in immune cell-derived extracellular vesicles that could promote islet β-cell apoptosis were identified.

### 2.4 Prediction of candidate miRNAs, TFs, and candidate genes

The TransmiR V2.0 [20] database was used to predict the upstream TFs of each candidate miRNA, and the “NetWork” platform was used to identify TFs that could positively regulate candidate miRNAs. In addition, TFs predicted to positively regulate candidate miRNAs were searched in PubMed. Furthermore, TFs expressed in immune cells that could positively regulate the expression of candidate miRNAs were screened.

The starBase tool was used to predict the candidate target genes of identified miRNAs. To confirm these candidate genes, we integrated the potential target genes of candidate miRNAs in four immune cells and confirmed them in PubMed using relevant keywords. Finally, the candidate genes that positively regulate the biological functions of islet β-cells, such as cell proliferation and glucose-stimulated insulin secretion (GSIS), were identified.

## 3 Results

### 3.1 miRNAs present in immune cell-derived extracellular vesicles promote β-cell apoptosis

**Figure 3:**
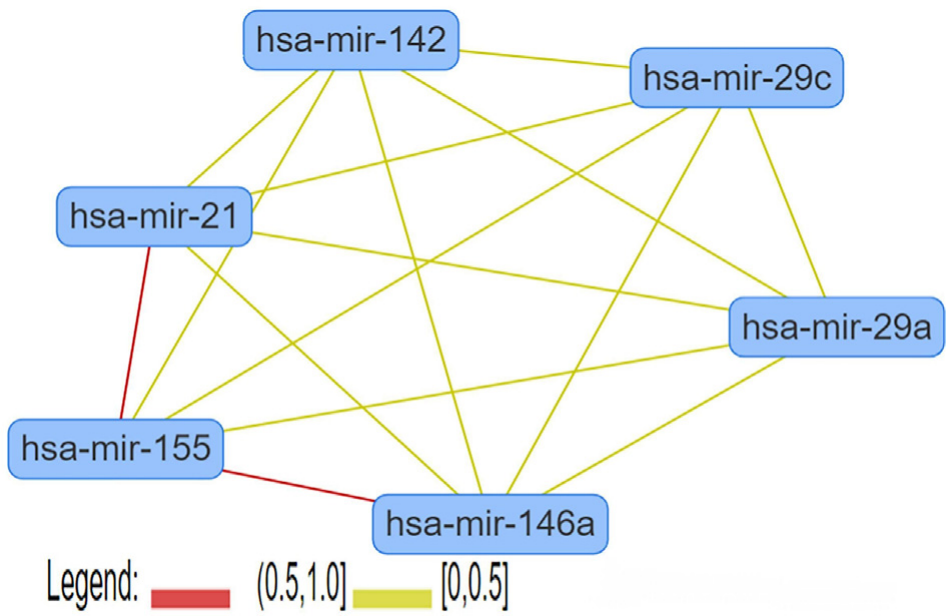
miRNA-miRNA interaction network. All miRNA interactions were positively correlated, which noted as red and yellow lines; the red lines indicate stronger correlation than the yellow lines.

miRNAs play important roles in biological function of immune cell-derived extracellular vesicles; the level of some miRNAs were significantly changed following immunoreaction in immune cells using GEO database. After removing redundancies from the two datasets retrieved from the GSE27997 and GSE137637, we selected miRNAs in extracellular vesicles derived from T lymphocytes, B lymphocytes, dendritic cells, and pro-inflammatory macrophages with fold change >|1.5| in their expression. In addition, we identified 17 miRNAs that promote islet β cell apoptosis in the context of T1DM (Table 2) based on previous studies. Upon analyzing the intersection between miRNAs in immune cell-derived extracellular vesicles and these 17 miRNAs (Supplementary Tables S5–S8), we obtained specific miRNAs and used them for subsequent analyses. Heat maps were drawn for these specific miRNAs based on their expression levels (Figure 2); miRNAs with high fold-change difference in their expression levels were identified as candidate miRNAs and included hsa-miR-142-3p, -142-5p, and -155-5p in extracellular vesicles derived from T lymphocytes, hsa-miR-21-5p, -155-5p, -29a-3p, -29b-3p, and -29c-3p in extracellular vesicles derived from B lymphocytes, hsa-miR-21-5p, -142-3p, and -142-5p in extracellular vesicles derived from dendritic cells, and hsa-miR-21-5p, -29a-3p, -29c-3p, and -146a-5p in extracellular vesicles derived from pro-inflammatory macrophages. Using candidate miRNAs, we constructed a miRNA-miRNA interaction network using MISIM V2.0 (http://www.lirmed.com/misim/allvsall) (Figure 3). All miRNA interactions were positively correlated (red and yellow lines in Figure 3). These miRNAs inhibited the expression of their target candidate genes and impaired the normal functioning of islet β cells. In addition, a strong correlation was observed between hsa-miR-21 and hsa-miR-155 (between 0.5 and 1), indicating that hsa-miR-21 and hsa-miR-155 have a stronger correlation with the occurrence of T1DM.

**Table 2:** miRNAs known to promote β cell apoptosis.

miRNA	Mechanism	References
hsa-miR-21-5p	pro-inflammatory cytokines induce miR-21 expression in human islet β cells by activating the transcription factor, NF-KB; miR-21 targets the pro-apoptotic mRNA, BCL2, thereby promoting islet β cell apoptosis	[23, 24, 79]
hsa-miR-34a	The P53 apoptotic pathway can be activated, thereby promoting β cell apoptosis.	[79]
hsa-miR-29a/b/c	miR-29 family RNA molecules promote β cell apoptosis by decreasing the expression levels of the anti-apoptotic protein, Mcl1	[25]
hsa-miR-142-3p	It regulates the NF-KB signaling pathway and stimulates the expression of the chemokine gene, *CCL2*, thereby promoting islet β cell apoptosis	[8]
hsa-miR-142-5p	It regulates the NF-KB signaling pathway and stimulates the expression of the chemokine gene, *CCL7,* thereby promoting islet β cell apoptosis	[8]
hsa-miR-155	hsa-miR-155 regulates the NF-KB signaling pathway and stimulates the expression of chemokine gene, *CXCL10*. In addition, increased hsa-miR-155 expression may reduce the expression levels of the anti-apoptotic gene, *GLIS3*, ultimately promoting islet β cell apoptosis	[2, 8]
hsa-miR-146a-5p	Human islet β cell exposure to the pro-inflammatory cytokines, IL-1β and TNF-α, increases miR-146 expression, resulting in islet β cell apoptosis and a decrease in glucose-induced insulin secretion	[13]
hsa-miR-10b-3p hsa-miR-186-5p hsa-miR-345-5p hsa-miR-376c-3p hsa-miR-425-3p hsa-miR-432-5p hsa-miR-493-3p hsa-miR-629-3p	Aside from miR-10b-3p, the levels of the other miRNAs are dysregulated following Coxsackievirus B5 injection. These miRNAs target various pattern recognition receptors (PRRs), such as IFHI1, TLR7, and TLR8, and induce pro-inflammatory cytokine production	[2]

In extracellular vesicles, miRNAs bind to RNA-binding proteins to increase their stability during transport to target cells where they exert their effects on cells. Using the miRbase (https://www.miRbase.org/) and RBPDB (http://rbpdb.ccbr.utoronto.ca/) databases, we analyzed candidate miRNAs. hsa-miR-142-3p present in extracellular vesicles secreted by T lymphocytes bound to the SFRS1 protein, while hsa-miR-21-5p and hsa-miR-29b-3p in extracellular vesicles derived from B lymphocytes bound to the PABPC1 and ACO1 proteins, respectively; hsa-miR-21-5p and hsa-miR-29c-3p in extracellular vesicles derived from pro-inflammatory macrophages bound to the ELAVL1 protein. Candidate miRNAs in extracellular vesicles derived from dendritic cells remain to be evaluated as no protein was found in published databases that bound to them. Information regarding the binding of miRNAs to proteins in extracellular vesicles secreted by T lymphocytes, pro-inflammatory macrophages, and B lymphocytes is listed in Table 3. Based on the existing database, we identified no RNA-binding proteins that could bind to miRNAs in the dendritic cells derived extracellular vesicles. Moreover, the EVpedia database (http://evpedia.info/evpedia2_xe/) suggests that these proteins are present in extracellular vesicles containing specific miRNAs of the related immune cells. Therefore, we concluded that hsa-miR-21-5p, hsa-miR-29b, hsa-miR-29c-3p, and hsa-miR-142-3p stably existed in extracellular vesicles derived from immune cells and promote islet β cell apoptosis.

**Table 3:** Binding situation between miRNAs and proteins in immune cell-derived extracellular vesicles.

	miRNA	RBP name	Matching sequence	Motif logo
miRNAs that bind to proteins in extracellular vesicles derived from T cells	hsa-miR-142-5p	SFRS9	AGCAC	
		KHDRBS3	CAUAAA	/
	hsa-miR-142-3p	Pum2	UGUA	
		ELAVL1	GUUU	/
		SFRS1	UGGA	/
	hsa-miR-155-5p	MBNL1	UGCU	
		YTHDC1	UAAUGC	
		KHDRBS3	GCUAAU	/
miRNAs that bind to proteins in extracellular vesicles derived from B lymphocytes	hsa-miR-155-5P	MBNL1	UGCU	
		YTHDC1	UAAUGC	
		KHDRBS3	GCUAAU	/
	hsa-miR-21-5p	PABPC1	ACUGAUG	
		ELAVL1	GUUG	/
	hsa-miR-29a-3p	SFRS9	AGCAC	
		RBMX	CCAU	
	hsa-miR-29b-3p	ACO1	CAGUGU	
		SFRS9	AGCAC	
		RBMX	CCAU	
	hsa-miR-29c-3p	SFRS9	AGCAC	
		RBMX	CCAU	
		FLAVL1	AUUU	/
miRNAs that bind to proteins in extracellular vesicles derived from pro-inflammatory macrophages	hsa-miR-21-5p	PABPC1	ACUGAUG	
		ELAVL1	GUUG	/
	hsa-miR-29a-3p	SFRS9	AGCAC	
		RBMX	CCAU	
	hsa-miR-29c-3p	SFRS9	AGCAC	
		RBMX	CCAU	
		ELAVL1	AUUU	/
	hsa-miR-146a-5p	RBMX	CCAU	

### 3.2 Transcriptional regulation of candidate miRNAs and their targeted effects on mRNAs

The level of some miRNAs of extracellular vesicles was significantly elevated following immunoreaction in immune cells, suggesting that the miRNA transcription was activated by special transcription factor in immune cells. Using the TransmiR V2.0 (http://www.cuilab.cn/transmir) database, we predicted the upstream transcription factors of candidate miRNAs to be hsa-miR-21, hsa-miR-29a, hsa-miR-29b, hsa-miR-29c, hsa-miR-142, hsa-miR-155, and hsa-miR-146a, and selected TFs that upregulated the candidate miRNAs.

**Figure 4:**
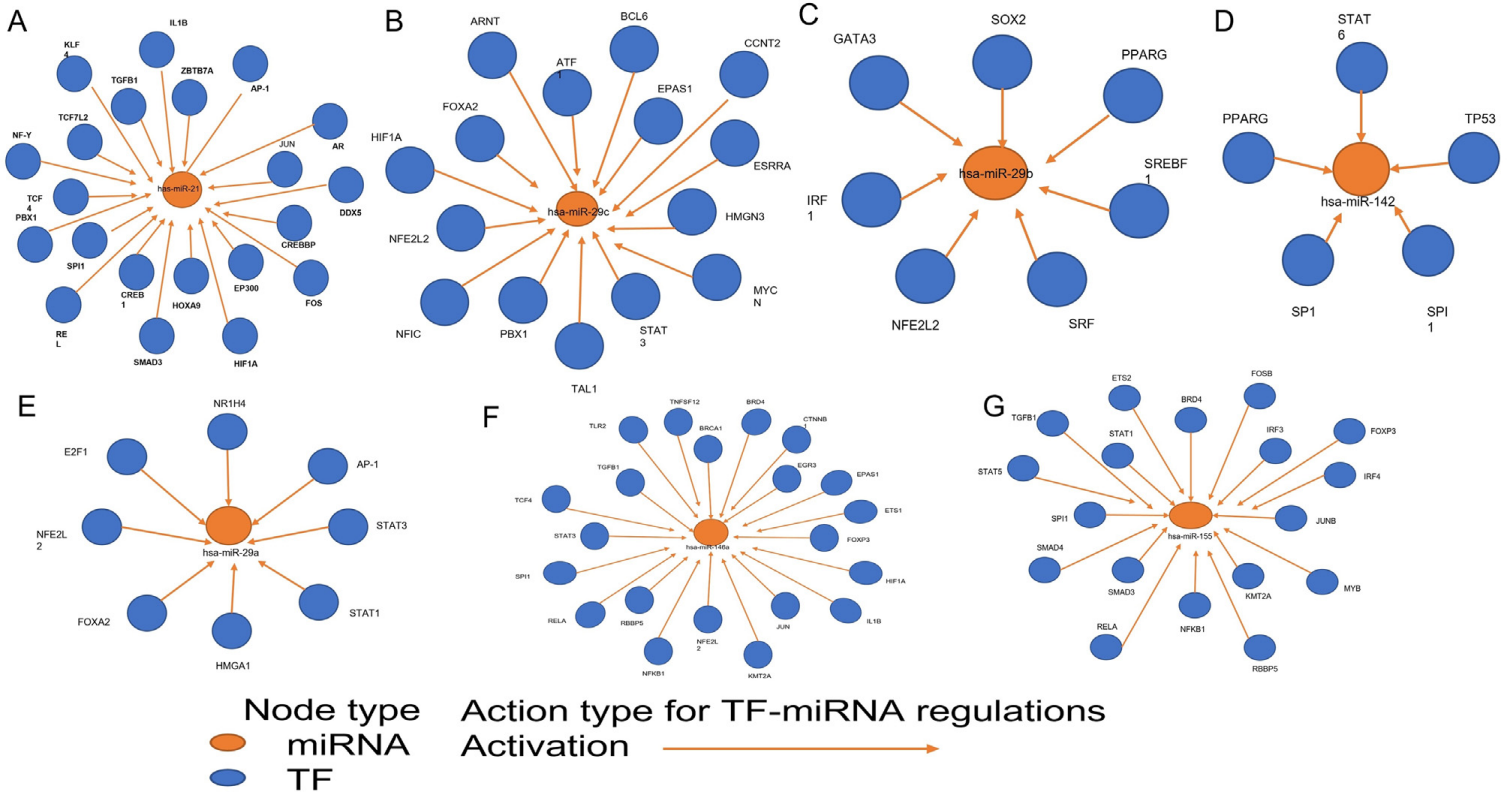
TF-miRNA interaction network. A. hsa-miR-21 and its upstream transcription factors; TFs that could positively regulate hsa-miR-21 included AP-1, JUN, KLF4, SMAD3, and TCF4; B. has-miR-29c and its upstream transcription factors; C. hsa-miR-29b and its upstream transcription factors; D. hsa-miR-142 and its upstream transcription factors; E. hsa-miR-29a and its upstream transcription factors, STAT3 and STAT1; F. hsa-miR-146a and its upstream transcription factors; TFs that exerted positive regulatory effects on hsa-miR-146a included EGR3, FOXP3, RELA, and STAT3; G. hsa-miR-155 and its upstream transcription factors; TFs that exerted positive effects on hsa-miR-155 included BRD4, ETS2, FOSB, FOXP3, IRF4, JUNB, NFKB1, SMAD3, and SMAD4.

TF-miRNA regulatory networks are shown in Figure 4. Five transcription factors, Ap-1, JUN, KLF4, SMAD3, and TCF4, appeared to have a positive effect on hsa-miR-21-5p (Figure 4A); three transcription factors, EGR3, RELA, and STAT3, positively regulated hsa-miR-146a (Figure 4B); nine transcription factors, BRD4, ETS2, FOSB, FOXP3, IRF4, JUNB, NFKB1, SMAD3, and SMAD4, upregulated hsa-miR-155 (Figure 4C); two transcription factors, STAT3 and STAT1, regulated hsa-miR-29a (Figure 4D). Intriguingly, SMAD3 and STAT3 do not only regulate one miRNA type; thus, their roles in islet β cell apoptosis remain to be fully evaluated. Meanwhile, we observed no transcription factors that simultaneously upregulated the levels of hsa-miR-29b, hsa-miR-29c, and hsa-miR-142 in immune cells (Figure 4E–G). Detailed information on these transcription factors is presented in Table 4.

**Table 4:** Upstream TFs of the candidate miRNAs in immune cells.

TF	Candidate miRNA(s)	Expression in immune cell(s)	References
AP-1	hsa-miR-21	Regulates the polarization of pro-inflammatory macrophages	[26, 32]
JUN	hsa-miR-21	M-CSF can promote its expression in macrophages, and JUN together with NF-KB promote the transformation of macrophages from M1 to M2	[27, 31]
KLF4	hsa-miR-21	Regulates the polarization of pro-inflammatory macrophages	[28, 33]
SMAD3	hsa-miR-21/hsa-miR-155	It mediates macrophage phenotype and anti-inflammatory transformation, and acts synergistically with TGFβ to maintain CD4^+^ T cell immune tolerance	[29, 34, 45]
TCF4	hsa-miR-21	It regulates development and maintains normal physiological functions in dendritic cells	[30, 35]
EGR3	hsa-miR-146a	EGR3 is expressed in bone pro-inflammatory macrophages; it binds to the ifngr1 promoter and inhibits its transcription under IFNβ stimulation, and this may be associated with anti-inflammatory effects	[42, 43]
FOXP3	hsa-miR-155	FOXP3 plays a role in FOXP3+/CD4+ regulatory T cell development	[46, 52]
RELA	hsa-miR-146a	RELA expression in pro-inflammatory macrophages promotes cytokine production, which is beneficial for immune function	[41, 44]
STAT3	hsa-miR-146a/hsa-miR-29a	STAT3 plays a role in B cell development	[37, 38, 42]
BRD4	hsa-miR-155	It plays a role in the transcription of some specific genes associated with CD4^+^T cells	[47, 53]
ETS2	hsa-miR-155	It binds to the miR-155 promoter sequence and induces high miR-155 expression in B cells	[48]
FOSB	hsa-miR-155	FOSB dimerizes with c-jun to induce T cell death	[49, 54]
IRF4	hsa-miR-155	It binds with SPI1 and BATF to form a transcription factor complex and plays an important role in the activation of B and T lymphocytes	[50]
JUNB	hsa-miR-155	After TCR activation, JUNB expression and transcriptional activity increases in T cells	[49, 54]
NFKB1	hsa-miR-155	It plays a role in B cell maturation	[51, 56]
SMAD4	hsa-miR-155	It plays a role in the expression of the ligand, selectin, in CD4^+^T cells	[45, 55]
STAT1	hsa-miR-29a	STAT1 indirectly regulates macrophage polarization in synergy with METTL3	[36, 39]

As for miRNAs in extracellular vesicles derived from T lymphocytes, B lymphocytes, dendritic cells, and pro-inflammatory macrophages, which were selected based on fold change, starBase was used to predict their potential downstream candidate genes. Based on the data from previous studies, we preliminarily identified candidate genes associated with the biological functions of beta cells; we found eight downstream candidate genes, *CDC42*, *SLC30A7*, *GNPNAT1*, *SMAD2*, *RICTOR*, *PELO*, *NNT*, and *RHEB*, associated with miRNAs in T lymphocyte-derived extracellular. There were 13 downstream candidate genes, *DHCR24*, *CAMTA1*, *FBXO28*, *CD59*, *GIT2*, *ZDHHC17*, *STAT3*, *FLOT2*, *ONECUT2*, *ARFRP1*, *OPA1*, *CDK6*, and *MTPN*, associated with miRNAs in extracellular vesicles derived from pro-inflammatory macrophages. Meanwhile, there were nine downstream candidate genes, *DHCR24*, *KDM5B*, *CAMTA1*, *CD59*, *DICER1*, *XRN1*, *HMGCR*, *CDK6*, and *MTPN*, associated with miRNAs in B lymphocyte-derived extracellular vesicles (Table 5).

**Table 5:** Target genes of the candidate miRNAs.

miRNA	Target genes	Functions	Mechanism	References
hsa-miR-21/29a/29b/29c/146a/155	DHCR24	↓β cell apoptosis	Inhibits the generation of reactive oxygen species (ROS)	[58]
hsa-miR-21/29a/29b/29c/146a/155	CAMTA1	Maintains β cell identity, and promotes insulin synthesis and secretion	Regulates NKX2-2 expression and promotes SLC2a2 and Mafa expression	[62]
hsa-miR-21/29a/29b/29c/146a/155	CD59	↑glucose-induced insulin secretion (GSIS)	CD59 interacts with SNARE proteins to promote insulin secretion	[61]
hsa-miR-21/29a/29b/29c/155	DICER1	Affects β cell mass, insulin secretion, and β cell development	Processes pre-miRNA and guarantees the expression of functional miRNAs in β cells	[80]
hsa-miR-21/29a/29b/29c/155	XRN1	↑insulin synthesis and protects β cells against cytokine-induced apoptosis	Promotes pro-apoptotic protein degradation	[59]
hsa-miR-142-3p/155	PELO	↑insulin synthesis and protects cells against cytokine-induced apoptosis	Promotes pro-apoptotic protein degradation	[59]
hsa-miR-21/29a/29b/29c/155	HMGCR	↑β cell mass and insulin secretion	Inhibits TAP/TAC activation	[80]
hsa-miR-142/155	NNT	↑insulin secretion	Increases the NADH/NAD + ratio under high glucose conditions and regulates the effects of Ca^2+^ on exocytosis	[64]
hsa-miR-21/29a/29b/29c/146a/155	CDK6	↑β cell proliferation	Regulates the G1/S phase of the growth cycle in β cells	[77]
hsa-miR-21/29a/29b/29c/146a/155	MTPN	Regulates insulin secretion	Controls F-actin formation and fuses the cyst with the plasma membrane	[65]
hsa-miR-21/29a/29c/146a/142	FBXO28	↑β cell survival under diabetic conditions	Protects β cells against apoptosis	[81]
hsa-miR-21/29a/29c/146a	GIT2	Affects insulin secretion and β cell mass	Controls insulin receptor-related signals	[66]
hsa-miR-21/29a/29c/146a	ZDHHC17	↑ β cell survival and glucose-induced insulin secretion	Inhibits the IL-1β-induced NF-KB signaling pathway and protects islet β cells against IL-1β-mediated β cell apoptosis	[67]
hsa-miR-21/29a/29c/146a	STAT3	↓β cell apoptosis and ↑β cell proliferation	Inhibits PTEN expression and promotes the phosphorylation of downstream AKTs, thereby triggering the transcription of β cell-related genes	[60]
hsa-miR-21/29a/29c/146a	ONECUT2	↑insulin secretion	Inhibits the expression of granulocyte proteins, thereby blocking the bioregulatory process of granulocyte protein-mediated insulin secretion inhibition	[25]
hsa-miR-21/29a/29c/146a	ARFRP1	↑insulin secretion	ARFRP1 associates with the Golgi-associated PDZ, coiled-coil motif-containing protein (GOPC), to regulate insulin secretion by controlling the localization of the SNAP25 protein on the plasma membrane	[68]
hsa-miR-21/29a/29c/146a	OPA1	↑insulin secretion	Maintains the integrity of the electron transport chain in the mitochondrion, thereby ensuring the production of ATP and providing energy for insulin secretion	[69]
hsa-miR-142-3p/155	CDC42	Regulates insulin secretion and expression	Promotes insulin secretion by inducing actin remodeling, and promotes insulin expression by phosphorylating ERK1/2 and NeuroD1	[70]
hsa-miR-142/155	SLC30A7	Regulates insulin expression	Regulates insulin expression by regulating Mtf1 transcriptional activity	[82]
hsa-miR-142/155	GNPNAT1	↑insulin secretion and may protect β cells	GNPNAT1 methylation may have a protective effect on beta cells and increases insulin secretion to meet up with increasing insulin demand	[71]
hsa-miR-142	SMAD2	Maintains β cell identity	Maintains β cell function and identity through the Tgfb2 pathway	[83]
hsa-miR-142	RICTOR	↑ β cell proliferation	RICTOR/mTORC2 downregulates FOXO1 and P27 expression by acting on PAKT-S437	[78]
hsa-miR-21/142	SIK2	↑insulin secretion	SIK2 is activated at high glucose levels, and phosphorylates P35	[72]
hsa-miR-21/142	SOX5	↑insulin secretion	Regulates the opening of calcium channels on the mitochondrial membrane and produces ATP synthase for the provision of energy for insulin secretion	[73]
hsa-miR-21/142	TSHZ1	Regulates β cell maturation and insulin secretion	Directly regulates CLEC16A or indirectly regulates CLE16A through PDX1	[74]
hsa-miR-21/142	ABCA1	Regulates insulin secretion	Maintains normal cholesterol levels, thereby preventing lipid toxicity-induced dysregulation of normal pancreatic β cell function, thus ensuring normal insulin secretion	[76]
hsa-miR-21/142	IDS	↑GSIS	Promotes insulin secretion by Phosphorylating PKCα and MARCKS	[75]

### 3.3 Construction of the diagram showing islet β cell apoptosis-promoting immune cell-derived extracellular vesicular miRNAs

**Figure 5:**
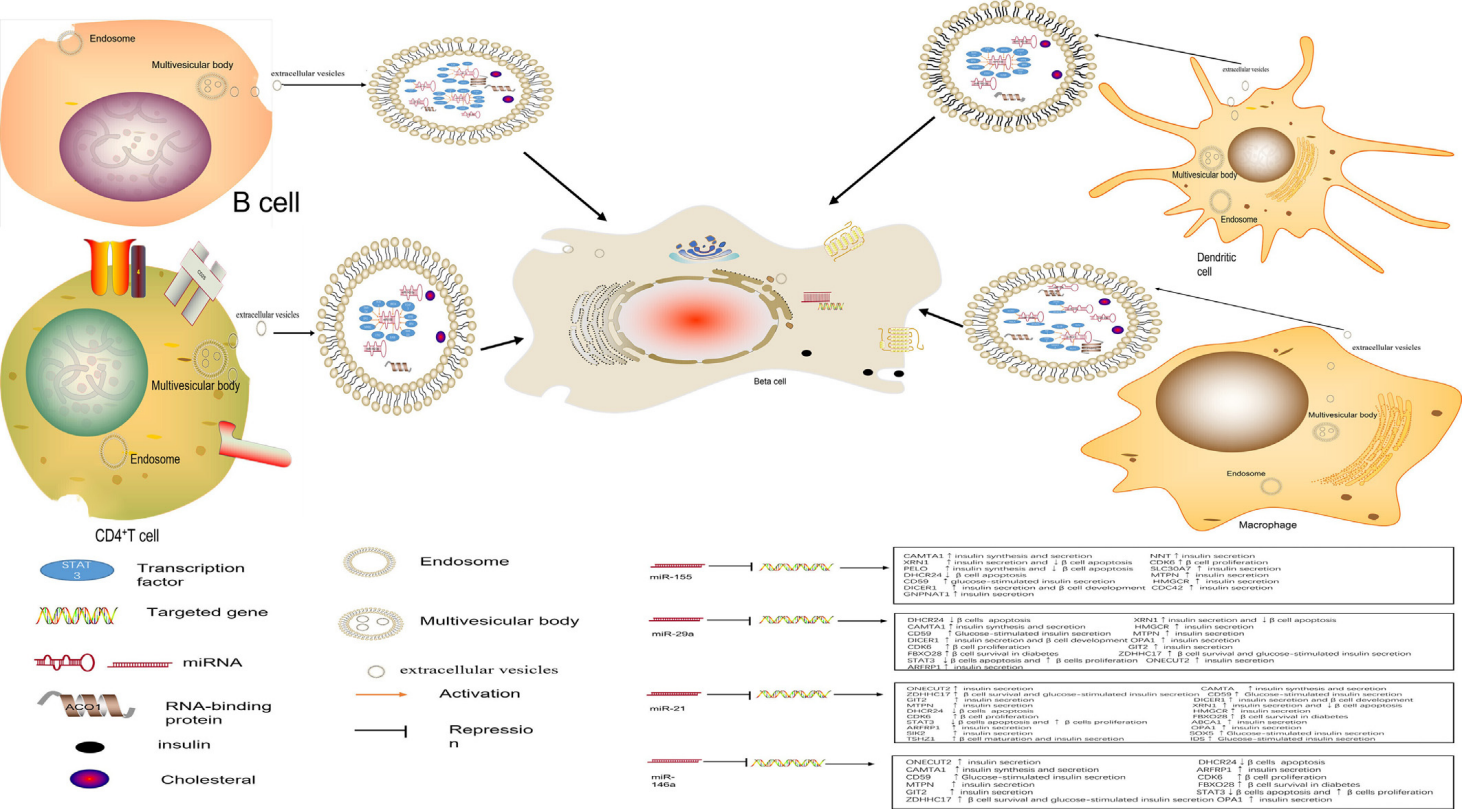
Immune cell-derived extracellular vesicular MicroRNAs induce pancreatic beta cell apoptosis.

The immune cells included in this study were CD4^+^ T lymphocytes, B lymphocytes, dendritic cells, and pro-inflammatory macrophages, all of which release cytokines and extracellular vesicles that contain specific miRNAs. In response to stimuli, cytokines are released by these immune cells and bind to cytokine receptors on islet β cells, thereby activating downstream transcription factors (e.g., NF-κB and STAT-1) that promote chemokine release. Immune cells are recruited to the islet β cell environment, thereby exposing islet β cells to a long-term inflammatory environment. miRNAs contained in extracellular vesicles can be positively regulated by upstream transcription factors, thereby increasing their expression. miR-155, miR-21, miR-146a, and miR-29a are transported by extracellular vesicles and transferred to β cells in their active form, where they bind to candidate genes that regulate cell function. Figure 5 illustrated the process of miRNA binding and their candidate genes in the cytoplasm of β cells. These candidate genes promote insulin secretion and islet β cell proliferation, and inhibit apoptosis. After miRNAs bind to their candidate genes, expression is inhibited, thereby causing islet β cell dysfunction, which decreases islet β cell count and decreases insulin secretion, and ultimately T1DM. In addition, in extracellular vesicles derived from B lymphocytes, miR-29b binds to the ACO1 protein, while in those derived from pro-inflammatory macrophages, miR-29c binds to the ELAVL1 protein. These miRNAs can exist stably in extracellular vesicles. Our results indicate that miR-155, miR-21, miR-146a, and miR-29a can regulate transcription factors, along with some target genes, which may play a role in T1DM progression through TFs-miRNA gene regulatory network.

## 4 Discussion

T1DM is a chronic disease prevalent worldwide, with significant deleterious effects on human health. Although the pathogenesis and treatment for T1DM have been extensively studied, the specific mechanism underlying islet β cell loss in T1DM remains unclear. Studies have reported that pattern recognition receptors (PRRs) (e.g., TLR3, TLR4, RIG, and MAD5) and cytokine receptors (IL-1R and TNFR, among others) are expressed on the surface of islet β cells [3]. PRRs can detect rotavirus nucleic acids [1, 2]. In addition, some pro-inflammatory cytokines (IL-β, TNF-α, and IFN-γ) bind to cytokine receptors, thereby activating transcription factors, such as NF-κB and STAT-1 [3], which induce the release of chemokines, such as CXCL10 and CCL2 [21,22], which in turn induce the recruitment of more immune cells to the islet, ultimately aggravating islet β cell apoptosis. Furthermore, MHC class I molecules expressed in islet β cells present autoantigens such as proinsulin, GAD65, and IA-2, which are recognized by CD8^+^ T cells. This process initiates islet β cell attack, which leads to progressive β cell loss [1]. Exogenous insulin supplementation is the conventional approach for T1DM treatment; however, miRNAs present in immune cell-derived extracellular vesicles can promote islet β cell apoptosis and serves as biomarkers for T1DM prevention and treatment.

miRNAs expressed in extracellular vesicles released by Jurakt cells, Raji cells, dendritic cells, and pro-inflammatory macrophages were analyzed, and the following eight miRNAs were found to promote islet β cell apoptosis: hsa-miR-142-3p, hsa-miR-142-5p, hsa-miR-155-5p, hsa-miR-21-5p, hsa-miR-29a-3p, hsa-miR-29b-3p, and hsa-miR-29c-3p. Guay et al. found that T lymphocyte-derived extracellular vesicles specifically contain miR-142-3p/-5p and miR-155: miR-142-3p is overexpressed in activated T lymphocytes and transferred to β cells where it promotes the expression of *CCL2*, *CCL7*, and *CXCL10,* which are involved in the chemokine signaling pathway [8]. In addition, miR-142-3p can regulate the expression of cytokine-encoding genes [8] which induces the formation an inflammatory environment by producing pro-inflammatory cytokines and facilitating immune cell recruitment. This leads to the impairment of normal islet β cell function and a reduction in islet β cell count. miR-21 can inhibit the transcription of the anti-apoptotic BCL2-encoding gene and increase caspase3 production, thereby promoting islet β cell apoptosis [23, 24]. During the early stages of diabetes, islet β cells are exposed to the inflammatory environment formed by pro-inflammatory cytokines; under these conditions, miR-29 overexpression inhibits the expression of the transcription factor, Onecut2, which is associated with defective insulin secretion. Furthermore, miR-29 overexpression-induced decrease in the levels of the antiapoptotic protein Mcl1 impairs mitochondrial function, cytochrome c release, and caspase3 activation, thereby accelerating islet β cell apoptosis [25].

Roggli et al. confirmed that following human islet β cell exposure to an inflammatory environment, miR-146a overexpression promoted islet β cell apoptosis and decreased GSIS levels; however, the specific mechanism underlying this process remains poorly understood [13].

TFs can regulate miRNA expression. Thus, we predicted a series of TFs that could positively regulate candidate miRNAs in the TransmiR V2.0 database. In addition, to confirm the accuracy of our data, we analyzed studies published in PubMed to verify these TFs. AP-1, JUN, KLF4, SMAD3, and TCF4 were found to promote miR-21 expression [26, 27, 28, 29, 30]. Based on the data obtained from our datasets, miR-21 was overexpressed in B lymphocytes, dendritic cells, and pro-inflammatory macrophages. By analyzing related literature, we confirmed that these miR-21-regulating TFs were expressed in at least one of these immune cells. AP-1, JUN, KLF4, and SMAD3 play a role in macrophage polarization [31, 32, 33, 34], while TCF4 plays a role in dendritic cell development and functional maintenance [35]. TFs, STAT1 and STAT3 can promote miR-29a expression [36, 37] in extracellular vesicles secreted by B lymphocytes and pro-inflammatory macrophages. STAT3 is required for B cell development [38], whereas STAT1 plays an indirect role in pro-inflammatory macrophage polymerization as METTL3 regulates pro-inflammatory macrophage polarization [39]; miR-146a is significantly overexpressed in extracellular vesicles derived from pro-inflammatory macrophages, while the TFs, EGR3, RELA, and STAT3 can upregulate miR-146a levels [40, 41, 42]. Both EGR3 and RELA are expressed in pro-inflammatory macrophages [38, 43, 44], while STAT3 is expressed in B lymphocytes [38]. Several upstream TFs, including JUNB, FOSB, BRD4, FOXP3, SMAD3, ETS2, IRF4, NFKB1, and SMAD4, regulate miR-155 expression [45, 46, 47, 48, 49, 50, 51]. Data obtained from our datasets indicate that miR-155 is overexpressed in extracellular vesicles derived from T and B lymphocytes. Literature was retrieved for these nine miR-155-regulating TFs to determine whether they were expressed in T or B cells. We concluded that the TFs JUNB, FOSB, BRD4, FOXP3, SMAD3, and SMAD4 are likely expressed in CD4^+^T cells [34, 52, 53, 54, 55], while ETS2 and NFKB1 are likely expressed in B cells [48, 56]. The complex between IRF4, SPI1, and BATF plays an important role in T and B cell activation [50]. By analyzing TFs, we confirmed that IRF4 is expressed in immune cells and positively regulates miRNAs promoting β cell apoptosis. T lymphocytes, B lymphocytes, dendritic cells, and pro-inflammatory macrophages can release extracellular vesicles containing specific TFs and deliver their regulated miRNAs to islet β cells following stimulation by pro-inflammatory cytokines. These miRNAs enter β cells and modulate the expression of target genes that regulate cell biological functions, such as insulin secretion and cell development, thereby dysregulating normal biological functions in islet β cells and causing metabolic dysfunction.

Specific miRNAs present in extracellular vesicles derived from T lymphocytes, B lymphocytes, dendritic cells, and pro-inflammatory macrophages were selected based on their fold-change values; these included 10 miRNAs, such as hsa-miR-142 and hsa-miR-155. We predicted the candidate target genes of these miRNAs, and an intersection analysis of the obtained candidate genes and their screening based on their roles in islet β cells led to the identification of the appropriate downstream targeting genes for miR-155, miR-21, miR-29a, and miR-146a. Among other functions, these genes regulate insulin secretion and islet β cell proliferation and protect islet β cells against apoptosis, thereby maintaining normal β cell function [8, 24, 25, 57]. miRNAs can interfere with islet β cell function by acting on different genes; however, some genes are not targeted by only one miRNA. For example, *DHCR24* can be targeted by miR-21, miR-29a, and miR-155 [58]. Apoptosis-induced loss of β cell mass is the main mechanism underlying T1DM, and ultimately leads to insufficient insulin secretion [5, 8]. The candidate genes, *DHCR24*, *XRN1*, *PELO*, and *STAT3,* can decrease islet β cell apoptosis by inhibiting reactive oxygen species generation and degrading pro-apoptotic proteins [58, 59, 60]. *XRN1 and PELO* protect islet β cells against cytokine-induced apoptosis [59]. Several candidate genes, including *CAMTA1* and *CD59*, play important roles in the biological process underlying insulin secretion [25, 61, 62, 63, 64, 65, 66, 67, 68, 69, 70, 71, 72, 73, 74, 75, 76]; *CDK6, STAT3,* and *RICTOR* participate in islet β cell proliferation [60, 77, 78]. Some candidate genes can regulate multiple biological processes in islet β cells; for instance, *STAT3* can promote islet β cell proliferation and protect them against apoptosis [60]. This increases diversity and complexity of islet β cell regulation. Targeting multiple regulatory target genes may interrupt biological processes in islet β cells, affect islet β cell function, and alter the course of the disease. Therefore, these predicted candidate genes should be further evaluated to identify key genes that play a critical role in maintaining normal islet β cell function.

In this study, by integrating the datasets and microarray analysis, we determined the miRNA expression in immune cell-derived extracellular vesicles. We focused on immune cell-derived extracellular vesicular miRNAs capable of inducing islet β cell apoptosis. Among these, miR-155, miR-21, miR-146a, and miR-29a were found to regulate transcription factors as well as some target genes, which can form a TFs-miRNA gene regulatory network; thus, they may play a key role in the progression of T1DM. Immune cells are speculated to secrete extracellular vesicles containing specific miRNAs, which can be transferred to islet β cells, thereby inhibiting normal islet β cell biological function via inhibiting the expression of candidate genes, leading to disease aggravation.

This study has some limitations. First, some miRNAs that regulate islet β cell apoptosis during T1DM may have been omitted due to different retrieval methods, whereas for some other miRNAs, it remains to be determined whether the experimental results obtained in mice comparable to those in humans; therefore, these miRNAs warrant further investigated. Second, based on the results obtained from the database analyzed in this study, binding proteins were not identified for some immune cell-derived extracellular vesicular miRNAs that affect islet β cell apoptosis; however, this does not indicate that these miRNAs could not exist stably in immune cell-derived extracellular vesicles. Third, we found no key genes nor TFs that promoted islet beta cell apoptosis, thus could not construct a specific TF-microRNA gene regulatory network.

TFs and candidate genes identified in this study remain to be assessed to confirm the molecules involved in islet β cell apoptosis. Finally, the current data was obtained from cell lines, including B cells, macrophages, dendritic cell, CD 4^+^ T cells, but not CD 8^+^ T cells, hence further studies are warranted prior to clinical applications. Despite these limitations, this study provides a theoretical basis and research direction for the construction of the TF-miRNA gene regulatory network, along with novel insights into the diagnosis, treatment, and prevention of T1DM.

## Declarations

### Author contribution statement

Yueyang Yu: Performed the experiments; Analyzed and interpreted the data; Wrote the paper.

Mengyin Li: Contributed reagents, materials, analysis tools or data; Wrote the paper.

Yuxuan Zhao: Contributed to reagents, materials, analysis tools or data.

Fangzhou Fan and Wenxiang Wu: Contributed to reagents, materials, analysis tools or data; Analyzed and interpreted the data.

Chunyu Bai and Yuhua Gao: Conceived and designed the experiments; Analyzed and interpreted the data; Wrote the paper.

### Funding statement

Chunyu Bai was supported by 10.13039/501100001809National Natural Science Foundation of China, China [31972755], Shandong Provincial Natural Science Foundation, China [ZR2020KH031], Project of Shandong Province Higher Educational Youth Innovation Science and Technology Program, China [2019KJK010], Shandong Provincial Natural Science Foundation, China (Grant No. ZR2020KH031), Project of Shandong Province Higher Educational Youth Innovation Science and Technology Program (2019KJK010).

### Data availability statement

Data included in article/supp. material/referenced in article.

### Declaration of interest's statement

The authors declare no conflict of interest.

### Additional information

No additional information is available for this paper.

## Appendix A Supplementary data

Supplemental.docx Supplemental.docx
