# Supplementary material for: Immune cell-derived extracellular vesicular microRNAs induce pancreatic beta cell apoptosis
Source: Heliyon. 2022 Dec 2;8(12):e11995. doi: 10.1016/j.heliyon.2022.e11995 (PMC9763775; doi:10.1016/j.heliyon.2022.e11995)
Supplement: Supplemental.docx [file mmc1.docx]

Supplemental information

Immune cell-derived extracellular vesicles MicroRNAs induce pancreatic beta cell apoptosis

| Table S1 | microRNA expression in extracellular vesicles of T lymphocyte in GSE27997 |
| --- | --- |
| Table S2 | microRNA expression in extracellular vesicles of dendritic cell in GSE27997 |
| Table S3 | microRNA expression in extracellular vesicles of B lymphocyte in GSE27997 |
| Table S4 | microRNA expression in extracellular vesicles of pro-inflammatory macrophage in GSE137637 |
| Table S5 | The microRNA can promote islet β cell apoptosis in T lymphocyte extracellular vesicles in GSE27997 |
| Table S6 | The microRNA can promote islet β cell apoptosis in B lymphocyte extracellular vesicles in GSE27997 |
| Table S7 | The microRNA can promote islet β cell apoptosis in dendritic cells extracellular vesicles in GSE27997 |
| Table S8 | The microRNA can promote islet β cell apoptosis in pro-inflammatory macrophage extracellular vesicles in GSE137637 |

TableS1microRNA expression in extracellular vesicles of T lymphocyte in GSE27997

| GSM692621 |  | GSM692622 |  | GSM692630 |  |
| --- | --- | --- | --- | --- | --- |
| hsa-miR-483-5p | 7.858503217 | hsa-miR-874 | 11.20179589 | hsa-miR-575 | 12.48838398 |
| hsa-miR-193b* | 7.645927783 | hsa-miR-575 | 11.16757392 | hsa-miR-630 | 11.7237884 |
| hsa-miR-575 | 7.448936238 | hsa-miR-10a* | 10.85987265 | hsa-miR-638 | 11.09488362 |
| hsa-miR-10a* | 7.213702629 | hsa-miR-193b* | 10.55243158 | hsa-miR-1225-5p | 10.30861721 |
| hsa-miR-451 | 7.084784317 | hsa-miR-188-5p | 10.53680538 | hsa-miR-125a-3p | 10.19844323 |
| hsa-miR-188-5p | 7.044174506 | hsa-miR-16 | 9.836403734 | hsa-miR-494 | 9.250920955 |
| hsa-miR-638 | 6.927753629 | hsa-miR-638 | 9.692384061 | hsa-miR-939 | 8.561126147 |
| hsa-miR-630 | 6.882817681 | hsa-let-7a | 9.637434815 | hsa-miR-513a-5p | 8.550087251 |
| hsa-miR-1225-5p | 6.778271644 | hsa-miR-103 | 9.597764458 | hsa-miR-19b | 7.66343049 |
| hsa-miR-874 | 6.725572886 | hsa-miR-20a | 9.571964205 | hsa-miR-150* | 7.566058359 |
| hsa-miR-92a | 6.528306421 | hsa-miR-142-3p | 9.497821839 | hsa-miR-92a | 7.513439446 |
| hsa-miR-20a | 6.124115339 | hsa-miR-19b | 9.413164875 | hsa-miR-483-5p | 7.352934123 |
| hsa-miR-16 | 5.93580214 | hsa-miR-483-5p | 9.314450037 | hsa-miR-663 | 7.329417088 |
| hsa-miR-193a-5p | 5.76209782 | hsa-miR-193a-5p | 9.283108705 | hsa-miR-181a | 7.267412172 |
| hsa-miR-654-5p | 5.658371979 | hsa-let-7b | 9.058015551 | hsa-miR-16 | 7.186956882 |
| hsa-miR-103 | 5.542209098 | hsa-miR-17 | 8.850253671 | hsa-miR-671-5p | 6.784734771 |
| hsa-let-7a | 5.465186578 | hsa-miR-92a | 8.7686279 | hsa-miR-342-3p | 6.778570228 |
| hsa-miR-223 | 5.271281022 | hsa-miR-1225-5p | 8.732000553 | hsa-miR-134 | 6.77082071 |
| hsa-miR-25 | 5.268843117 | hsa-let-7f | 8.614597818 | hsa-miR-940 | 6.712909113 |
| hsa-miR-940 | 5.262493457 | hsa-miR-20b | 8.522533831 | hsa-miR-29c | 6.682470992 |
| hsa-miR-17 | 5.186301702 | hsa-miR-29c | 8.378573333 | hsa-miR-188-5p | 6.673456795 |
| hsa-miR-198 | 5.162021069 | hsa-miR-21 | 8.378165264 | hsa-miR-20a | 6.534041626 |
| hsa-miR-939 | 5.144717878 | hsa-miR-181a | 8.374592773 | hsa-miR-1224-5p | 6.506818155 |
| hsa-miR-19b | 5.123709645 | hsa-miR-363 | 8.363238179 | hsa-miR-135a* | 6.360125424 |
| hsa-miR-20b | 5.086301398 | hsa-miR-422a | 8.229345335 | hsa-miR-142-3p | 6.210976205 |
| hsa-miR-1228 | 5.07523737 | hsa-miR-25 | 8.154451935 | hsa-miR-17 | 6.166633928 |
| hsa-miR-424* | 5.058871984 | hsa-miR-494 | 8.134673264 | hsa-miR-765 | 6.12786066 |
| hsa-miR-584 | 4.945736181 | hsa-miR-630 | 8.127171966 | hsa-miR-654-5p | 6.060610277 |
| hsa-miR-1234 | 4.914973075 | hsa-miR-7 | 8.125362755 | hsa-miR-19a | 6.011099124 |
| hsa-let-7b | 4.885636053 | hsa-miR-15b | 8.116335804 | hsa-miR-1228 | 6.007027315 |
| hsa-miR-671-5p | 4.883771937 | hsa-miR-19a | 8.096652684 | hsa-miR-324-3p | 5.978994899 |
| hsa-miR-363 | 4.842498325 | hsa-miR-452 | 8.043612629 | hsa-miR-1234 | 5.890805735 |
| hsa-miR-1226* | 4.834107427 | hsa-miR-342-3p | 8.036415209 | hsa-miR-25 | 5.845388302 |
| hsa-miR-1238 | 4.78925318 | hsa-miR-198 | 8.021580656 | hsa-miR-125b-1* | 5.826233569 |
| hsa-miR-125a-3p | 4.775465643 | hsa-miR-654-5p | 7.930223356 | hsa-miR-513b | 5.780262926 |
| hsa-miR-181a | 4.766490671 | hsa-miR-658 | 7.847222476 | hsa-miR-371-5p | 5.708487332 |
| hsa-miR-513a-5p | 4.699945705 | hsa-miR-128 | 7.75375645 | hsa-miR-1238 | 5.690827517 |
| hsa-miR-658 | 4.675057947 | hsa-miR-106b | 7.567985468 | hsa-miR-191* | 5.623882524 |
| hsa-miR-760 | 4.673613304 | hsa-miR-223 | 7.531244678 | hsa-miR-20b | 5.587825576 |
| hsa-miR-21 | 4.646875662 | hsa-miR-451 | 7.508639175 | hsa-miR-21 | 5.556236501 |
| hsa-miR-15b | 4.622513453 | hsa-miR-130b | 7.443799643 | hsa-let-7a | 5.554113511 |
| hsa-miR-1225-3p | 4.592380296 | hsa-miR-125a-3p | 7.426170596 | hsa-miR-363 | 5.519626375 |
| hsa-let-7f | 4.575373584 | hsa-miR-423-5p | 7.414294446 | hsa-let-7b | 5.518481975 |
| hsa-miR-1224-5p | 4.564324376 | hsa-miR-107 | 7.382240373 | hsa-miR-572 | 5.495355033 |
| hsa-miR-142-3p | 4.500539674 | hsa-miR-15a | 7.326682004 | hsa-miR-451 | 5.434328179 |
| hsa-miR-766 | 4.474703342 | hsa-miR-892b | 7.30683914 | hsa-miR-181b | 5.407023137 |
| hsa-miR-191* | 4.459060466 | hsa-miR-135a* | 7.302391358 | hsa-miR-210 | 5.366375434 |
| hsa-miR-29c | 4.40948989 | hsa-miR-500 | 7.302297663 | hsa-miR-877 | 5.358648068 |
| hsa-miR-500 | 4.406523929 | hsa-let-7g | 7.286288959 | hsa-miR-425* | 5.336815659 |
| hsa-miR-892b | 4.372637871 | hsa-let-7i | 7.280584448 | hsa-miR-30d | 5.318083295 |
| hsa-miR-500* | 4.295795005 | hsa-miR-513a-5p | 7.170677873 | hsa-miR-223 | 5.316388735 |
| hsa-miR-425* | 4.25405707 | hsa-miR-921 | 7.154254275 | hsa-miR-1225-3p | 5.300306454 |
| hsa-miR-342-3p | 4.214961399 | hsa-let-7d | 7.107988822 | hsa-miR-7 | 5.28983048 |
| hsa-let-7b* | 4.210750705 | hsa-miR-939 | 7.061741047 | hsa-miR-107 | 5.245652455 |
| hsa-miR-149 | 4.191081002 | hsa-miR-1226* | 7.054588251 | hsa-miR-128 | 5.227661392 |
| hsa-miR-662 | 4.187063215 | hsa-miR-148a | 6.944316726 | hsa-miR-106b | 5.194664356 |
| hsa-miR-324-3p | 4.16120457 | hsa-miR-210 | 6.812551725 | hsa-let-7f | 5.172364143 |
| hsa-miR-494 | 4.151033138 | hsa-miR-324-3p | 6.778629492 | hsa-miR-33b* | 5.163395916 |
| hsa-let-7f-1* | 4.141624069 | hsa-miR-1224-5p | 6.762287489 | hsa-let-7b* | 5.143884124 |
| hsa-miR-197 | 4.135045276 | hsa-miR-150* | 6.735221642 | hsa-miR-564 | 5.142408231 |
| hsa-miR-33b* | 4.134731894 | hsa-miR-501-5p | 6.700274697 | hsa-miR-550 | 5.142103782 |
| hsa-miR-1237 | 4.120389214 | hsa-miR-628-3p | 6.682936627 | hsa-miR-142-5p | 5.13602866 |
| hsa-miR-602 | 4.035139722 | hsa-miR-155 | 6.604461812 | hsa-miR-149 | 5.13302264 |
| hsa-miR-422a | 3.996088303 | hsa-miR-520b | 6.556517407 | hsa-miR-423-5p | 5.094744938 |
| hsa-miR-513b | 3.944635889 | hsa-miR-23a | 6.548869729 | hsa-let-7f-1* | 5.059284466 |
| hsa-miR-452 | 3.942636538 | hsa-miR-29a | 6.540670162 | hsa-miR-93 | 5.039395704 |
| hsa-miR-550 | 3.921804762 | hsa-miR-574-5p | 6.519797999 | hsa-miR-766 | 4.987575741 |
| hsa-miR-19a | 3.91512645 | hsa-miR-29b | 6.486188033 | hsa-miR-130b | 4.98220411 |
| hsa-miR-150* | 3.857467247 | hsa-miR-424* | 6.454113683 | hsa-miR-1237 | 4.92954547 |
| hsa-miR-29a | 3.853615518 | hsa-miR-601 | 6.394923218 | hsa-miR-602 | 4.89462007 |
| hsa-miR-128 | 3.823998465 | hsa-miR-142-5p | 6.383760521 | hsa-miR-15a | 4.854158938 |
| hsa-miR-93 | 3.792505705 | hsa-miR-513b | 6.344219989 | hsa-miR-563 | 4.852743481 |
| hsa-let-7i | 3.698328917 | hsa-miR-425 | 6.309572935 | hsa-miR-15b | 4.822330654 |
| hsa-miR-130b | 3.689426421 | hsa-miR-134 | 6.300967167 | hsa-miR-574-5p | 4.718916898 |
| hsa-miR-563 | 3.684308399 | hsa-miR-671-5p | 6.26460392 | hsa-let-7i | 4.709269738 |
| hsa-miR-634 | 3.621366544 | hsa-miR-662 | 6.247190864 | hsa-miR-23a | 4.655045014 |
| hsa-miR-601 | 3.600479564 | hsa-miR-30d | 6.242088052 | hsa-miR-148a | 4.646408524 |
| hsa-miR-501-5p | 3.594754897 | hsa-miR-93 | 6.214345517 | hsa-let-7g | 4.623495258 |
| hsa-miR-1229 | 3.578561378 | hsa-miR-27a | 6.143980495 | hsa-miR-636 | 4.540844684 |
| hsa-miR-30d | 3.57605779 | hsa-miR-487b | 6.093394284 | hsa-miR-103 | 4.521560061 |
| hsa-let-7g | 3.533233507 | hsa-miR-212 | 6.058254336 | hsa-miR-516a-5p | 4.505063075 |
| hsa-miR-148a | 3.530918888 | hsa-miR-22 | 6.053561969 | hsa-miR-155 | 4.496757547 |
| hsa-miR-574-5p | 3.523327745 | hsa-miR-760 | 6.047434109 | hsa-miR-197 | 4.480871463 |
| hsa-miR-150 | 3.511237849 | hsa-miR-181b | 5.960983764 | hsa-miR-933 | 4.469436947 |
| hsa-miR-765 | 3.508859958 | hsa-miR-500* | 5.900866956 | hsa-miR-26a | 4.463114594 |
| hsa-miR-23a | 3.507953064 | hsa-miR-18a | 5.872396576 | hsa-miR-29b | 4.449093944 |
| hsa-miR-502-3p | 3.493925797 | hsa-miR-331-3p | 5.856791905 | hsa-miR-513c | 4.448976409 |
| hsa-miR-181b | 3.478053937 | hsa-miR-520e | 5.839282332 | hsa-miR-557 | 4.44209865 |
| hsa-miR-92b | 3.474342269 | hsa-miR-887 | 5.839249456 | hsa-miR-623 | 4.411159016 |
| hsa-miR-296-5p | 3.470929734 | hsa-miR-584 | 5.779378806 | hsa-miR-659 | 4.386186296 |
| hsa-miR-933 | 3.459114735 | hsa-let-7c | 5.758438812 | hsa-miR-296-5p | 4.357905142 |
| hsa-miR-107 | 3.437063793 | hsa-miR-610 | 5.693138867 | hsa-miR-874 | 4.348280094 |
| hsa-miR-155 | 3.405051861 | hsa-miR-202 | 5.640503165 | hsa-miR-1229 | 4.340864573 |
| hsa-miR-636 | 3.401165155 | hsa-miR-490-5p | 5.598060244 | hsa-miR-92b | 4.33245542 |
| hsa-let-7d | 3.378711519 | hsa-miR-30e | 5.586685844 | hsa-miR-583 | 4.245969049 |
| hsa-miR-15a | 3.366069079 | hsa-miR-150 | 5.576917589 | hsa-miR-129-3p | 4.211008421 |
| hsa-miR-877* | 3.364942835 | hsa-miR-338-5p | 5.559642425 | hsa-miR-877* | 4.160892168 |
| hsa-miR-921 | 3.348351448 | hsa-miR-24 | 5.52086483 | hsa-miR-365 | 4.147095334 |
| hsa-miR-129-3p | 3.334641487 | hsa-miR-30b | 5.508620573 | hsa-miR-1227 | 4.13491971 |
| hsa-miR-331-3p | 3.323140068 | hsa-miR-1228 | 5.486556115 | hsa-miR-422a | 4.094246558 |
| hsa-miR-200a* | 3.322058833 | hsa-miR-542-5p | 5.461088127 | hsa-miR-29a | 4.089844211 |
| hsa-miR-210 | 3.314858964 | hsa-miR-513c | 5.453293883 | hsa-miR-129* | 4.048928925 |
| hsa-miR-1227 | 3.295421481 | hsa-miR-18b | 5.348692655 | hsa-miR-601 | 4.028883398 |
| hsa-miR-135a* | 3.289974902 | hsa-miR-26a | 5.33474261 | hsa-miR-22 | 4.026729611 |
| hsa-miR-887 | 3.283353977 | hsa-miR-200a* | 5.33129403 | hsa-miR-760 | 4.017616769 |
| hsa-miR-365 | 3.273236129 | hsa-miR-378 | 5.29602557 | hsa-miR-30e | 3.981777797 |
| hsa-let-7d* | 3.223930824 | hsa-miR-26b | 5.259820797 | hsa-miR-484 | 3.978229992 |
| hsa-miR-129* | 3.217755307 | hsa-miR-330-3p | 5.240687673 | hsa-miR-18a | 3.964382631 |
| hsa-miR-513c | 3.191516625 | hsa-miR-502-3p | 5.219003677 | hsa-miR-198 | 3.954338778 |
| hsa-miR-27a | 3.131743885 | hsa-miR-27b | 5.217179774 | hsa-miR-634 | 3.939757657 |
| hsa-miR-423-5p | 3.12110596 | hsa-miR-371-5p | 5.171506694 | hsa-miR-936 | 3.908352638 |
| hsa-miR-520b | 3.114286325 | hsa-miR-301a | 5.130106397 | hsa-miR-125b-2* | 3.908096231 |
| hsa-miR-484 | 3.021130197 | hsa-miR-9* | 5.113465742 | hsa-miR-425 | 3.886697544 |
| hsa-miR-610 | 3.01823724 | hsa-miR-339-3p | 5.087637322 | hsa-miR-617 | 3.880862515 |
| hsa-miR-126 | 2.997431251 | hsa-miR-373* | 5.06612935 | hsa-miR-584 | 3.839646171 |
| hsa-miR-382 | 2.908235471 | hsa-miR-126 | 5.034997789 | hsa-miR-24 | 3.837415029 |
| hsa-miR-30b | 2.871084515 | hsa-miR-940 | 5.029282971 | hsa-miR-708 | 3.833628787 |
| hsa-miR-510 | 2.864363483 | hsa-miR-183* | 5.024380049 | hsa-miR-331-3p | 3.815789273 |
| hsa-miR-623 | 2.859550322 | hsa-miR-342-5p | 5.010309 | hsa-miR-370 | 3.809339731 |
| hsa-miR-617 | 2.835053998 | hsa-miR-149* | 5.007089528 | hsa-miR-342-5p | 3.798775812 |
| hsa-miR-106b | 2.81861557 | hsa-miR-361-5p | 4.9484159 | hsa-let-7c | 3.754162156 |
| hsa-miR-134 | 2.81575496 | hsa-miR-877 | 4.940982191 | hsa-miR-149* | 3.72955164 |
| hsa-miR-483-3p | 2.747047045 | hsa-miR-519e* | 4.889724124 | hsa-miR-767-3p | 3.727385859 |
| hsa-miR-29b | 2.736928387 | hsa-miR-765 | 4.876083363 | hsa-miR-1226* | 3.703025371 |
| hsa-miR-22 | 2.731601159 | hsa-miR-96 | 4.779629373 | hsa-miR-665 | 3.699634966 |
| hsa-miR-632 | 2.722727848 | hsa-miR-623 | 4.753255688 | hsa-miR-518c* | 3.631682524 |
| hsa-miR-133a | 2.699535976 | hsa-miR-145 | 4.724378785 | hsa-miR-625* | 3.617418578 |
| hsa-miR-425 | 2.680236528 | hsa-miR-936 | 4.710942805 | hsa-miR-486-5p | 3.598056478 |
| hsa-miR-767-3p | 2.665893886 | hsa-miR-1234 | 4.707000729 | hsa-miR-202 | 3.587542494 |
| hsa-miR-572 | 2.66213035 | hsa-miR-140-5p | 4.706897793 | hsa-miR-631 | 3.56965835 |
| hsa-miR-490-5p | 2.652770507 | hsa-miR-590-5p | 4.693733683 | hsa-miR-18b | 3.558244996 |
| hsa-miR-631 | 2.652135452 | hsa-miR-602 | 4.66876884 | hsa-miR-610 | 3.555091755 |
| hsa-miR-371-5p | 2.616323019 | hsa-miR-146a | 4.635571428 | hsa-miR-378 | 3.551193167 |
| hsa-miR-27b | 2.528679651 | hsa-miR-185 | 4.632556162 | hsa-miR-18b* | 3.532648372 |
| hsa-miR-520e | 2.489143872 | hsa-miR-296-5p | 4.618687389 | hsa-miR-99b* | 3.527857644 |
| hsa-miR-498 | 2.470100331 | hsa-miR-140-3p | 4.603943552 | hsa-miR-181a* | 3.523027275 |
| hsa-miR-24 | 2.468844608 | hsa-miR-98 | 4.588393965 | hsa-miR-483-3p | 3.498101098 |
| hsa-miR-338-5p | 2.460543844 | hsa-miR-148b* | 4.552367258 | hsa-let-7d | 3.481744015 |
| hsa-miR-136 | 2.449334709 | hsa-miR-28-3p | 4.55095732 | hsa-miR-186 | 3.479696006 |
| hsa-miR-622 | 2.43755911 | hsa-miR-766 | 4.550142825 | hsa-miR-301a | 3.408496542 |
| hsa-miR-337-3p | 2.428984842 | hsa-miR-151-5p | 4.54238212 | hsa-miR-622 | 3.327303461 |
| hsa-miR-373* | 2.423141007 | hsa-miR-424 | 4.5305663 | hsa-miR-150 | 3.262660358 |
| hsa-miR-542-5p | 2.396113481 | hsa-miR-99b* | 4.524201388 | hsa-miR-409-3p | 3.262022304 |
| hsa-miR-671-3p | 2.388448573 | hsa-miR-617 | 4.511899394 | hsa-miR-133a | 3.23941792 |
| hsa-miR-660 | 2.337400674 | hsa-miR-197 | 4.500465875 | hsa-miR-26b | 3.201322605 |
| hsa-miR-663 | 2.308876956 | hsa-miR-30c | 4.494783535 | hsa-miR-671-3p | 3.155307259 |
| hsa-miR-30c | 2.293435455 | hsa-miR-663 | 4.467289744 | hsa-miR-30b | 3.148818009 |
| hsa-miR-625* | 2.264340578 | hsa-miR-632 | 4.466335423 | hsa-miR-361-5p | 3.136278321 |
| hsa-miR-299-5p | 2.264149634 | hsa-miR-186 | 4.461487223 | hsa-miR-1236 | 3.121610994 |
| hsa-miR-26a-2* | 2.262375392 | hsa-miR-151-3p | 4.458586362 | hsa-miR-27a | 3.036292506 |
| hsa-miR-211 | 2.241274739 | hsa-miR-622 | 4.454355936 | hsa-miR-520b | 3.015074763 |
| hsa-let-7c | 2.24041546 | hsa-miR-518e* | 4.44181242 | hsa-miR-212 | 3.001011711 |
| hsa-miR-335* | 2.236756479 | hsa-miR-1225-3p | 4.435384627 | hsa-miR-126 | 2.997608382 |
| hsa-miR-186 | 2.210951403 | hsa-miR-510 | 4.418896632 | hsa-miR-887 | 2.908125221 |
| hsa-miR-7 | 2.208244156 | hsa-miR-513a-3p | 4.40538028 | hsa-miR-18a* | 2.83286543 |
| hsa-miR-361-5p | 2.204202275 | hsa-miR-509-5p | 4.404009595 | hsa-miR-892b | 2.812983637 |
| hsa-miR-28-3p | 2.197724463 | hsa-miR-181a* | 4.372008846 | hsa-miR-498 | 2.789898255 |
| hsa-miR-202 | 2.187490737 | hsa-miR-627 | 4.357932667 | hsa-miR-32* | 2.745719467 |
| hsa-miR-330-3p | 2.141966772 | hsa-miR-660 | 4.355820511 | hsa-miR-30c | 2.724856609 |
| hsa-miR-936 | 2.140506754 | hsa-miR-769-3p | 4.325009458 | hsa-miR-424 | 2.705280173 |
| hsa-miR-18b* | 2.126125668 | hsa-miR-557 | 4.313888584 | hsa-miR-423-3p | 2.686964944 |
| hsa-miR-505 | 2.112249633 | hsa-miR-23b | 4.257095261 | hsa-miR-133b | 2.686917735 |
| hsa-miR-125b-2* | 2.095793782 | hsa-miR-532-5p | 4.254999125 | hsa-miR-30c-1* | 2.650268633 |
| hsa-miR-29c* | 2.092895032 | hsa-miR-183 | 4.242975654 | hsa-miR-140-3p | 2.630433089 |
| hsa-miR-140-5p | 2.087111039 | hsa-miR-564 | 4.207653353 | hsa-miR-513a-3p | 2.591947772 |
| hsa-miR-192 | 2.048899988 | hsa-miR-125b-2* | 4.18784246 | hsa-miR-146a | 2.571573626 |
| hsa-miR-212 | 2.037954247 | hsa-miR-631 | 4.120247919 | hsa-miR-29c* | 2.571299206 |
| hsa-miR-151-3p | 2.036099657 | hsa-miR-1238 | 4.110703621 | hsa-miR-23b | 2.530061344 |
| hsa-miR-133b | 2.034274642 | hsa-miR-17* | 4.100524046 | hsa-miR-30c-2* | 2.527420971 |
| hsa-miR-10b | 2.008885655 | hsa-miR-23a* | 4.087097 | hsa-miR-299-5p | 2.524761902 |
| hsa-miR-885-5p | 1.97495318 | hsa-miR-641 | 4.043892654 | hsa-miR-221 | 2.488186529 |
| hsa-miR-487b | 1.967012915 | hsa-miR-10b* | 4.010913233 | hsa-miR-625 | 2.422206729 |
| hsa-miR-145 | 1.955893896 | hsa-miR-382 | 3.937290637 | hsa-miR-520e | 2.403261187 |
| hsa-miR-193b | 1.948337086 | hsa-miR-365 | 3.881464987 | hsa-miR-10b* | 2.383398565 |
| hsa-miR-181a* | 1.947612453 | hsa-miR-324-5p | 3.868947722 | hsa-miR-196b | 2.37803694 |
| hsa-miR-518d-3p | 1.94130593 | hsa-miR-625 | 3.858203094 | hsa-miR-27b | 2.374397102 |
| hsa-miR-149* | 1.922559829 | hsa-miR-191* | 3.853246986 | hsa-let-7d* | 2.364462136 |
| hsa-miR-328 | 1.880212417 | hsa-miR-659 | 3.851954119 | hsa-miR-181d | 2.33296103 |
| hsa-miR-454* | 1.877593129 | hsa-miR-29c* | 3.829692512 | hsa-miR-516b | 2.298471198 |
| hsa-miR-518a-3p | 1.876022158 | hsa-miR-33b* | 3.793077088 | hsa-miR-324-5p | 2.295059534 |
| hsa-miR-126* | 1.866446383 | hsa-miR-1237 | 3.781574912 | hsa-miR-632 | 2.283147287 |
| hsa-miR-302a | 1.852362362 | hsa-miR-550 | 3.776446362 | hsa-miR-595 | 2.282599475 |
| hsa-miR-520d-3p | 1.848251341 | hsa-miR-374a | 3.765255182 | hsa-miR-629* | 2.280024959 |
| hsa-miR-23b | 1.832797824 | hsa-miR-340 | 3.757656546 | hsa-miR-769-3p | 2.260443277 |
| hsa-miR-551b | 1.823798047 | hsa-miR-518c* | 3.752910692 | hsa-miR-532-5p | 2.253096291 |
| hsa-miR-557 | 1.795502667 | hsa-miR-193b | 3.726023627 | hsa-miR-328 | 2.251304031 |
| hsa-miR-135a | 1.790528722 | hsa-miR-572 | 3.691968418 | hsa-miR-345 | 2.246875928 |
| hsa-miR-151-5p | 1.788763869 | hsa-miR-200c | 3.675471147 | hsa-miR-1233 | 2.231337148 |
| hsa-miR-769-3p | 1.760850432 | hsa-miR-650 | 3.659974153 | hsa-miR-566 | 2.222806422 |
| hsa-miR-369-5p | 1.75231199 | hsa-miR-374b | 3.606340802 | hsa-let-7i* | 2.208258175 |
| hsa-miR-1236 | 1.746258163 | hsa-miR-770-5p | 3.605259488 | hsa-miR-297 | 2.182153828 |
| hsa-miR-486-5p | 1.715916983 | hsa-miR-629 | 3.598632593 | hsa-miR-489 | 2.172435098 |
| hsa-miR-204 | 1.715749681 | hsa-miR-425* | 3.585879969 | hsa-miR-193a-5p | 2.171567995 |
| hsa-miR-346 | 1.708518572 | hsa-miR-634 | 3.51533014 | hsa-miR-505 | 2.153704905 |
| hsa-miR-215 | 1.703209275 | hsa-let-7c* | 3.490186581 | hsa-miR-374a | 2.145781199 |
| hsa-miR-183* | 1.680219804 | hsa-let-7d* | 3.480267799 | hsa-miR-432 | 2.141483384 |
| hsa-miR-519e* | 1.675491518 | hsa-miR-629* | 3.471055859 | hsa-miR-181c | 2.136955405 |
| hsa-miR-576-5p | 1.674603076 | hsa-miR-628-5p | 3.460614151 | hsa-miR-101 | 2.132682425 |
| hsa-miR-668 | 1.662629741 | hsa-miR-196a | 3.457597691 | hsa-miR-335* | 2.121368056 |
| hsa-miR-937 | 1.6469371 | hsa-miR-498 | 3.419420165 | hsa-miR-96 | 2.10730434 |
| hsa-miR-556-3p | 1.637056916 | hsa-miR-192 | 3.416454796 | hsa-miR-122 | 2.100176833 |
| hsa-miR-520a-3p | 1.626320463 | hsa-miR-101 | 3.411137106 | hsa-miR-184 | 2.091923204 |
| hsa-miR-770-5p | 1.617662732 | hsa-miR-7-1* | 3.398992107 | hsa-miR-662 | 2.054766183 |
| hsa-miR-1224-3p | 1.609579581 | hsa-let-7b* | 3.391691668 | hsa-miR-1226 | 2.036290107 |
| hsa-miR-142-5p | 1.603136114 | hsa-miR-148b | 3.378542496 | hsa-miR-574-3p | 2.022870362 |
| hsa-miR-200b* | 1.601548059 | hsa-miR-181d | 3.375504951 | hsa-miR-192 | 2.002221287 |
| hsa-miR-125a-5p | 1.598904095 | hsa-miR-92b | 3.369982275 | hsa-miR-500* | 2.000698215 |
| hsa-miR-453 | 1.559665564 | hsa-miR-181c* | 3.365220537 | hsa-miR-501-5p | 1.995532658 |
| hsa-miR-127-3p | 1.552813603 | hsa-miR-99b | 3.326950274 | hsa-miR-186* | 1.98289705 |
| hsa-miR-598 | 1.543464644 | hsa-miR-518a-3p | 3.322896818 | hsa-miR-185 | 1.97971198 |
| hsa-miR-513a-3p | 1.53829303 | hsa-miR-491-5p | 3.312773331 | hsa-miR-340* | 1.968354889 |
| hsa-miR-219-5p | 1.530126098 | hsa-miR-508-3p | 3.299517429 | hsa-miR-539 | 1.959886384 |
| hsa-miR-548c-3p | 1.524583989 | hsa-miR-370 | 3.295145527 | hsa-miR-629 | 1.948324683 |
| hsa-miR-302b | 1.504673684 | hsa-miR-149 | 3.292889538 | hsa-miR-660 | 1.932754908 |
| hsa-miR-26a | 1.504236608 | hsa-miR-636 | 3.282638631 | hsa-miR-301b | 1.907704934 |
| hsa-miR-564 | 1.500418169 | hsa-miR-583 | 3.28134524 | hsa-miR-187* | 1.870406587 |
| hsa-let-7f-2* | 1.500342067 | hsa-miR-196b | 3.270754571 | hsa-miR-196a | 1.841190982 |
| hsa-miR-28-5p | -1.514148647 | hsa-miR-432 | 3.27066832 | hsa-miR-526b | 1.830860631 |
| hsa-miR-516b | -1.514222481 | hsa-miR-1226 | 3.250284814 | hsa-miR-937 | 1.829117674 |
| hsa-miR-520g | -1.517862399 | hsa-miR-492 | 3.249809249 | hsa-miR-605 | 1.818927106 |
| hsa-miR-581 | -1.539113917 | hsa-miR-200b* | 3.243370227 | hsa-miR-126* | 1.787304445 |
| hsa-miR-509-5p | -1.562205134 | hsa-miR-194 | 3.243149048 | hsa-miR-520d-3p | 1.760222353 |
| hsa-miR-92a-1* | -1.563163817 | hsa-miR-340* | 3.242447009 | hsa-miR-374b | 1.759688074 |
| hsa-miR-612 | -1.565884814 | hsa-miR-221 | 3.240083101 | hsa-miR-140-5p | 1.756741381 |
| hsa-miR-383 | -1.581438203 | hsa-miR-1229 | 3.23704197 | hsa-miR-424* | 1.7450185 |
| hsa-miR-302b* | -1.614750161 | hsa-miR-1228* | 3.219302755 | hsa-miR-98 | 1.738936561 |
| hsa-let-7e | -1.623401785 | hsa-miR-516a-5p | 3.213221363 | hsa-miR-302a | 1.719296537 |
| hsa-miR-586 | -1.658740838 | hsa-miR-933 | 3.202654838 | hsa-miR-7-1* | 1.678715359 |
| hsa-miR-944 | -1.671042011 | hsa-miR-516b | 3.188947575 | hsa-miR-628-5p | 1.636087454 |
| hsa-miR-593 | -1.686822957 | hsa-miR-181c | 3.186233537 | hsa-miR-222 | 1.62705474 |
| hsa-miR-146a* | -1.707617703 | hsa-miR-301b | 3.179227522 | hsa-miR-371-3p | 1.620513005 |
| hsa-miR-143 | -1.728698321 | hsa-miR-130a | 3.126718696 | hsa-miR-361-3p | 1.596620323 |
| hsa-miR-615-5p | -1.737032105 | hsa-miR-514 | 3.125650288 | hsa-miR-129-5p | 1.596185849 |
| hsa-miR-217 | -1.756255117 | hsa-miR-362-5p | 3.103406027 | hsa-miR-421 | 1.570995216 |
| hsa-miR-1 | -1.778224696 | hsa-miR-92a-1* | 3.102270256 | hsa-miR-613 | 1.565398757 |
| hsa-miR-373 | -1.779809288 | hsa-miR-505 | 3.098395374 | hsa-miR-450a | 1.531359035 |
| hsa-miR-517c | -1.791187723 | hsa-miR-125b-1* | 3.095951814 | hsa-miR-500 | 1.524375243 |
| hsa-miR-384 | -1.806894392 | hsa-let-7f-1* | 3.080405329 | hsa-miR-556-3p | 1.522890942 |
| hsa-miR-523 | -1.828987385 | hsa-miR-215 | 3.02753439 | hsa-miR-326 | 1.518881432 |
| hsa-miR-101 | -1.831547066 | hsa-miR-129-3p | 3.023892885 | hsa-miR-373* | 1.51535317 |
| hsa-miR-526b | -1.832688347 | hsa-miR-421 | 3.014300521 | hsa-miR-542-5p | 1.505463816 |
| hsa-miR-185 | -1.844035652 | hsa-miR-129-5p | 2.97246676 | hsa-miR-337-3p | 1.501122413 |
| hsa-miR-553 | -1.870848297 | hsa-miR-886-3p | 2.96868739 | hsa-miR-650 | 1.500832163 |
| hsa-miR-218-2* | -1.929930196 | hsa-miR-125b | 2.945324593 | hsa-miR-518e | -1.535841184 |
| hsa-miR-454 | -1.935900736 | hsa-miR-506 | 2.912070039 | hsa-let-7e | -1.540493 |
| hsa-miR-412 | -1.977513343 | hsa-miR-184 | 2.910399153 | hsa-miR-300 | -1.54325686 |
| hsa-miR-600 | -1.991674314 | hsa-miR-28-5p | 2.856032096 | hsa-miR-132 | -1.546919215 |
| hsa-miR-25* | -1.992018532 | hsa-miR-608 | 2.854395456 | hsa-miR-943 | -1.594059884 |
| hsa-miR-606 | -2.020798293 | hsa-miR-563 | 2.851763322 | hsa-miR-410 | -1.661037177 |
| hsa-miR-300 | -2.044759044 | hsa-miR-129* | 2.848376103 | hsa-miR-653 | -1.662845243 |
| hsa-miR-21* | -2.074654336 | hsa-miR-345 | 2.838626109 | hsa-miR-507 | -1.684163344 |
| hsa-miR-512-5p | -2.078483559 | hsa-miR-136 | 2.808940116 | hsa-miR-146b-3p | -1.71976646 |
| hsa-miR-9* | -2.088994378 | hsa-miR-423-3p | 2.787404116 | hsa-miR-639 | -1.721408838 |
| hsa-miR-30d* | -2.093462697 | hsa-miR-877* | 2.763829081 | hsa-miR-493* | -1.732497988 |
| hsa-miR-144 | -2.108069841 | hsa-miR-1227 | 2.758144518 | hsa-miR-23a* | -1.767618974 |
| hsa-miR-139-3p | -2.115428486 | hsa-miR-141 | 2.742837828 | hsa-miR-591 | -1.792226117 |
| hsa-miR-597 | -2.116155764 | hsa-miR-525-5p | 2.721570937 | hsa-miR-147 | -1.806154489 |
| hsa-miR-136* | -2.137639388 | hsa-miR-625* | 2.710482605 | hsa-miR-588 | -1.819265192 |
| hsa-miR-520a-5p | -2.157102932 | hsa-miR-484 | 2.699490507 | hsa-miR-642 | -1.834687115 |
| hsa-miR-499-3p | -2.160115429 | hsa-miR-518f | 2.685064972 | hsa-miR-105 | -1.83549543 |
| hsa-miR-591 | -2.16341406 | hsa-miR-152 | 2.682430282 | hsa-miR-147b | -1.839317084 |
| hsa-miR-619 | -2.168891191 | hsa-miR-362-3p | 2.647486737 | hsa-miR-888* | -1.844096191 |
| hsa-miR-802 | -2.169799292 | hsa-miR-30b* | 2.629241875 | hsa-miR-302c* | -1.844270697 |
| hsa-miR-1228* | -2.188178383 | hsa-miR-518d-3p | 2.626673067 | hsa-miR-493 | -1.848718882 |
| hsa-miR-106b* | -2.209137589 | hsa-miR-509-3-5p | 2.615875714 | hsa-miR-99a* | -1.867737729 |
| hsa-miR-203 | -2.211885853 | hsa-miR-18b* | 2.612571281 | hsa-miR-222* | -1.896036165 |
| hsa-miR-508-5p | -2.215252435 | hsa-miR-454 | 2.598742704 | hsa-miR-153 | -1.901436024 |
| hsa-miR-567 | -2.230177901 | hsa-miR-217 | 2.584745497 | hsa-miR-335 | -1.913058821 |
| hsa-miR-220b | -2.285288239 | hsa-miR-942 | 2.517512519 | hsa-miR-561 | -1.918569745 |
| hsa-miR-376a | -2.318973189 | hsa-miR-453 | 2.507158607 | hsa-miR-130a* | -1.924053062 |
| hsa-miR-558 | -2.325114436 | hsa-miR-520d-3p | 2.49868891 | hsa-miR-640 | -1.957505235 |
| hsa-miR-452* | -2.334002822 | hsa-miR-141* | 2.497541653 | hsa-miR-337-5p | -1.977669006 |
| hsa-miR-32 | -2.340173132 | hsa-miR-30a | 2.49152509 | hsa-miR-637 | -1.987833143 |
| hsa-miR-345 | -2.361674509 | hsa-miR-455-3p | 2.456648159 | hsa-miR-200b* | -1.995583408 |
| hsa-miR-935 | -2.376407009 | hsa-miR-124 | 2.449898693 | hsa-miR-145* | -1.996873754 |
| hsa-miR-708* | -2.387310297 | hsa-miR-551b* | 2.444687487 | hsa-miR-138-2* | -2.016981453 |
| hsa-miR-554 | -2.397892631 | hsa-miR-934 | 2.428319376 | hsa-miR-592 | -2.02595919 |
| hsa-miR-433 | -2.427807459 | hsa-miR-195 | 2.412695453 | hsa-miR-21* | -2.034686494 |
| hsa-miR-589 | -2.446674862 | hsa-miR-132 | 2.409502076 | hsa-miR-367* | -2.045673229 |
| hsa-miR-29b-2* | -2.450545162 | hsa-miR-550* | 2.406251182 | hsa-miR-376a | -2.061065265 |
| hsa-miR-377 | -2.454888274 | hsa-miR-367 | 2.37580868 | hsa-miR-16-1* | -2.081884849 |
| hsa-miR-105 | -2.457968737 | hsa-miR-302a | 2.360272455 | hsa-miR-218-2* | -2.088957281 |
| hsa-miR-649 | -2.459014618 | hsa-miR-526b | 2.357768703 | hsa-miR-220c | -2.108715884 |
| hsa-miR-34c-3p | -2.469984357 | hsa-miR-486-5p | 2.352158784 | hsa-miR-767-5p | -2.11612546 |
| hsa-miR-876-3p | -2.479927612 | hsa-miR-29b-1* | 2.320894683 | hsa-miR-541* | -2.13927874 |
| hsa-miR-486-3p | -2.502070301 | hsa-miR-18a* | 2.31630675 | hsa-miR-218-1* | -2.165348737 |
| hsa-miR-206 | -2.528859403 | hsa-miR-497* | 2.313529821 | hsa-miR-19a* | -2.185233927 |
| hsa-miR-19a* | -2.556835556 | hsa-miR-648 | 2.299758142 | hsa-miR-597 | -2.218083161 |
| hsa-miR-503 | -2.559843607 | hsa-miR-9 | 2.282772224 | hsa-miR-578 | -2.218560163 |
| hsa-miR-611 | -2.568302162 | hsa-miR-133a | 2.248603106 | hsa-miR-486-3p | -2.255969533 |
| hsa-miR-367* | -2.570126911 | hsa-miR-33a | 2.226235753 | hsa-miR-367 | -2.264652282 |
| hsa-miR-137 | -2.595096995 | hsa-miR-652 | 2.217413258 | hsa-miR-499-3p | -2.280306058 |
| hsa-miR-579 | -2.597998252 | hsa-miR-769-5p | 2.209904196 | hsa-miR-921 | -2.281201345 |
| hsa-miR-616 | -2.617727393 | hsa-miR-483-3p | 2.201223372 | hsa-miR-590-3p | -2.298274893 |
| hsa-miR-708 | -2.627970578 | hsa-miR-361-3p | 2.189909797 | hsa-miR-511 | -2.303550783 |
| hsa-miR-30a* | -2.680724504 | hsa-miR-10b | 2.186129811 | hsa-miR-125a-5p | -2.356389609 |
| hsa-miR-130a | -2.690079924 | hsa-miR-649 | 2.171169388 | hsa-miR-620 | -2.393551253 |
| hsa-miR-549 | -2.696195318 | hsa-miR-219-5p | 2.157165778 | hsa-miR-455-3p | -2.412695088 |
| hsa-miR-222 | -2.712354313 | hsa-miR-639 | 2.089460335 | hsa-miR-485-3p | -2.447229337 |
| hsa-miR-421 | -2.714062065 | hsa-miR-376a | 2.075604534 | hsa-miR-208b | -2.450536153 |
| hsa-miR-379 | -2.71475544 | hsa-let-7e | 2.058873655 | hsa-miR-200c | -2.46633286 |
| hsa-miR-588 | -2.757297472 | hsa-miR-1236 | 2.048361264 | hsa-miR-194* | -2.485899958 |
| hsa-miR-888 | -2.772701862 | hsa-miR-30e* | 1.994369168 | hsa-miR-497* | -2.488597629 |
| hsa-miR-214* | -2.812231876 | hsa-miR-767-3p | 1.984056227 | hsa-miR-504 | -2.501343418 |
| hsa-miR-188-3p | -2.84447609 | hsa-miR-885-5p | 1.978730333 | hsa-miR-299-3p | -2.525304335 |
| hsa-miR-497* | -2.86638253 | hsa-miR-133b | 1.973996131 | hsa-miR-875-3p | -2.535863832 |
| hsa-miR-92b* | -2.887775716 | hsa-miR-30c-1* | 1.94636584 | hsa-miR-628-3p | -2.539472718 |
| hsa-miR-33b | -2.890878892 | hsa-miR-200b | 1.943824854 | hsa-miR-376c | -2.54581423 |
| hsa-miR-16-1* | -2.89331882 | hsa-miR-503 | 1.935997755 | hsa-miR-363* | -2.558479993 |
| hsa-miR-577 | -2.917187493 | hsa-miR-542-3p | 1.921413897 | hsa-miR-509-3-5p | -2.589891202 |
| hsa-miR-335 | -2.923078279 | hsa-miR-518f* | 1.919900322 | hsa-miR-143 | -2.608242609 |
| hsa-miR-448 | -2.930410171 | hsa-miR-125a-5p | 1.904290241 | hsa-miR-589* | -2.61533 |
| hsa-miR-216a | -2.935067693 | hsa-miR-509-3p | 1.875386857 | hsa-miR-495 | -2.62268291 |
| hsa-miR-493 | -2.951551068 | hsa-miR-611 | 1.870467145 | hsa-miR-541 | -2.631244367 |
| hsa-miR-138 | -2.973835892 | hsa-let-7i* | 1.867824146 | hsa-miR-127-5p | -2.633995186 |
| hsa-miR-24-1* | -2.978223819 | hsa-miR-516a-3p | 1.854647887 | hsa-miR-379 | -2.640192887 |
| hsa-miR-340 | -2.987687888 | hsa-miR-299-5p | 1.831108019 | hsa-miR-101* | -2.641756363 |
| hsa-miR-551b* | -3.022997411 | hsa-miR-138-2* | 1.812294569 | hsa-miR-200b | -2.688798014 |
| hsa-miR-363* | -3.029176031 | hsa-miR-526b* | 1.81110431 | hsa-miR-554 | -2.701452077 |
| hsa-miR-573 | -3.080687532 | hsa-miR-135a | 1.81018632 | hsa-miR-208a | -2.711576041 |
| hsa-miR-138-1* | -3.12214958 | hsa-miR-545* | 1.789882915 | hsa-miR-10a* | -2.752686173 |
| hsa-miR-644 | -3.140724 | hsa-miR-302c* | 1.78880531 | hsa-miR-375 | -2.770843959 |
| hsa-miR-208b | -3.152703077 | hsa-miR-222 | 1.776767395 | hsa-miR-323-3p | -2.792001692 |
| hsa-miR-767-5p | -3.15676255 | hsa-miR-92a-2* | 1.774397735 | hsa-miR-100 | -2.831666042 |
| hsa-miR-561 | -3.162539576 | hsa-miR-645 | 1.752941086 | hsa-miR-649 | -2.862930118 |
| hsa-miR-34c-5p | -3.208983772 | hsa-miR-501-3p | 1.711042245 | hsa-miR-1 | -2.892244172 |
| hsa-miR-585 | -3.241893636 | hsa-miR-450a | 1.694989872 | hsa-miR-148a* | -2.893402768 |
| hsa-miR-541 | -3.242034398 | hsa-miR-99a | 1.690560575 | hsa-miR-922 | -2.896592723 |
| hsa-miR-499-5p | -3.25995742 | hsa-miR-299-3p | 1.686741067 | hsa-miR-431* | -2.965537217 |
| hsa-miR-138-2* | -3.294118771 | hsa-miR-186* | 1.670187561 | hsa-miR-485-5p | -2.969521134 |
| hsa-miR-191 | -3.297664918 | hsa-miR-20a* | 1.646925909 | hsa-miR-196a* | -2.978485895 |
| hsa-miR-516a-5p | -3.386621773 | hsa-miR-193a-3p | 1.584370018 | hsa-miR-876-3p | -2.985216595 |
| hsa-miR-490-3p | -3.38959134 | hsa-miR-570 | -1.512669188 | hsa-miR-412 | -2.990428493 |
| hsa-miR-23a* | -3.425719991 | hsa-miR-520h | -1.533293375 | hsa-miR-200c* | -2.994908315 |
| hsa-miR-580 | -3.436664597 | hsa-miR-302a* | -1.539464655 | hsa-miR-615-5p | -3.012064638 |
| hsa-miR-218-1* | -3.437703166 | hsa-miR-92b* | -1.539792208 | hsa-miR-187 | -3.025661566 |
| hsa-miR-222* | -3.442975443 | hsa-miR-331-5p | -1.553804528 | hsa-miR-214 | -3.030401581 |
| hsa-miR-339-5p | -3.479991443 | hsa-miR-218-1* | -1.556856518 | hsa-let-7c* | -3.032567114 |
| hsa-miR-376b | -3.494840086 | hsa-miR-196a* | -1.562459159 | hsa-miR-323-5p | -3.045287585 |
| hsa-miR-449b | -3.501051991 | hsa-miR-214* | -1.57429271 | hsa-miR-377* | -3.047544892 |
| hsa-miR-196a* | -3.525241291 | hsa-miR-377* | -1.587971382 | hsa-miR-380* | -3.056450926 |
| hsa-miR-298 | -3.53850715 | hsa-miR-148a* | -1.595078821 | hsa-miR-616* | -3.109405733 |
| hsa-miR-331-5p | -3.552316253 | hsa-miR-329 | -1.597697192 | hsa-miR-651 | -3.113562082 |
| hsa-miR-885-3p | -3.567232423 | hsa-miR-523 | -1.60119592 | hsa-miR-217 | -3.125528149 |
| hsa-miR-216b | -3.574408703 | hsa-miR-548a-5p | -1.601453181 | hsa-miR-621 | -3.128039832 |
| hsa-miR-487a | -3.589481639 | hsa-miR-637 | -1.607074438 | hsa-miR-496 | -3.139831039 |
| hsa-miR-589* | -3.613018905 | hsa-miR-330-5p | -1.615499123 | hsa-miR-325 | -3.145393109 |
| hsa-miR-593* | -3.615058467 | hsa-miR-511 | -1.617311491 | hsa-miR-555 | -3.154804695 |
| hsa-miR-380* | -3.615956977 | hsa-miR-200a | -1.619275594 | hsa-miR-600 | -3.198333665 |
| hsa-miR-362-5p | -3.636278799 | hsa-miR-31 | -1.63584873 | hsa-miR-888 | -3.232594138 |
| hsa-miR-153 | -3.638779213 | hsa-miR-372 | -1.646412429 | hsa-miR-214* | -3.232828076 |
| hsa-let-7g* | -3.63917449 | hsa-miR-34a* | -1.660820626 | hsa-miR-383 | -3.252159263 |
| hsa-miR-556-5p | -3.722376235 | hsa-miR-448 | -1.664242422 | hsa-miR-27a* | -3.278027589 |
| hsa-miR-608 | -3.731858869 | hsa-miR-105 | -1.672801864 | hsa-miR-508-3p | -3.285195326 |
| hsa-miR-33a | -3.764123689 | hsa-miR-548a-3p | -1.682157452 | hsa-miR-593* | -3.294056339 |
| hsa-miR-127-5p | -3.764780974 | hsa-miR-518e | -1.700489745 | hsa-miR-96* | -3.301216131 |
| hsa-miR-890 | -3.768908277 | hsa-miR-606 | -1.701458581 | hsa-miR-517* | -3.308404726 |
| hsa-miR-296-3p | -3.786260782 | hsa-miR-30d* | -1.710545996 | hsa-miR-509-3p | -3.348629782 |
| hsa-miR-297 | -3.812090202 | hsa-let-7a* | -1.71071351 | hsa-miR-188-3p | -3.375125151 |
| hsa-miR-587 | -3.818693077 | hsa-miR-661 | -1.73042141 | hsa-miR-579 | -3.380956428 |
| hsa-miR-576-3p | -3.8547174 | hsa-miR-576-3p | -1.733596579 | hsa-miR-339-5p | -3.395132818 |
| hsa-miR-187* | -3.887525289 | hsa-miR-556-3p | -1.790644235 | hsa-miR-381 | -3.40638091 |
| hsa-miR-542-3p | -3.916357597 | hsa-miR-562 | -1.810754954 | hsa-miR-1231 | -3.423270788 |
| hsa-miR-920 | -3.922057482 | hsa-miR-888 | -1.829283681 | hsa-miR-27b* | -3.443904468 |
| hsa-miR-27a* | -3.923231873 | hsa-miR-619 | -1.840721932 | hsa-miR-569 | -3.506021708 |
| hsa-miR-147b | -3.926473306 | hsa-miR-597 | -1.85345215 | hsa-miR-603 | -3.506042621 |
| hsa-miR-675 | -4.016197142 | hsa-miR-147b | -1.860162208 | hsa-miR-616 | -3.510146895 |
| hsa-miR-938 | -4.07239335 | hsa-miR-363* | -1.86666415 | hsa-miR-216a | -3.538416144 |
| hsa-miR-96* | -4.12100346 | hsa-miR-589 | -1.92562206 | hsa-miR-891a | -3.545412277 |
| hsa-miR-620 | -4.135241506 | hsa-miR-449b | -1.934684445 | hsa-miR-675 | -3.550293682 |
| hsa-miR-219-1-3p | -4.147502901 | hsa-miR-599 | -1.94867576 | hsa-miR-619 | -3.558697098 |
| hsa-miR-875-3p | -4.201351565 | hsa-miR-325 | -1.956496226 | hsa-miR-429 | -3.574943016 |
| hsa-miR-208a | -4.289914796 | hsa-miR-616* | -1.959123925 | hsa-miR-941 | -3.622845621 |
| hsa-miR-214 | -4.356551366 | hsa-miR-626 | -1.978258121 | hsa-miR-137 | -3.662823902 |
| hsa-miR-323-5p | -4.373265281 | hsa-miR-33b | -1.980677457 | hsa-miR-220b | -3.717378749 |
| hsa-miR-744 | -4.384635994 | hsa-miR-491-3p | -1.985476584 | hsa-miR-302d* | -3.729458043 |
| hsa-miR-302d* | -4.411935417 | hsa-miR-135b* | -1.994615738 | hsa-miR-372 | -3.786310801 |
| hsa-miR-24-2* | -4.431198684 | hsa-miR-647 | -2.003770726 | hsa-miR-216b | -3.787810151 |
| hsa-miR-187 | -4.487075818 | hsa-miR-298 | -2.007466034 | hsa-miR-938 | -3.827553725 |
| hsa-miR-941 | -4.524418478 | hsa-miR-222* | -2.012252681 | hsa-miR-487a | -3.880652377 |
| hsa-miR-135b* | -4.605597703 | hsa-miR-651 | -2.034311799 | hsa-miR-219-1-3p | -3.93374168 |
| hsa-miR-922 | -4.623939013 | hsa-miR-380 | -2.042765224 | hsa-miR-200a | -3.950287956 |
| hsa-miR-1231 | -4.858795426 | hsa-miR-220b | -2.044306632 | hsa-miR-920 | -3.955016745 |
|  | -5.056822806 | hsa-miR-554 | -2.052144376 | hsa-miR-452 | -3.962983368 |
|  | -5.097705914 | hsa-miR-376b | -2.091248338 | hsa-miR-433 | -3.984316447 |
|  |  | hsa-miR-302b* | -2.091438589 | hsa-miR-92b* | -4.069489036 |
|  |  | hsa-miR-323-3p | -2.093133117 | hsa-miR-452* | -4.422944811 |
|  |  | hsa-miR-590-3p | -2.103025056 |  |  |
|  |  | hsa-miR-551a | -2.103507735 |  |  |
|  |  | hsa-miR-593 | -2.133408122 |  |  |
|  |  | hsa-miR-573 | -2.137285544 |  |  |
|  |  | hsa-miR-384 | -2.157896151 |  |  |
|  |  | hsa-miR-485-5p | -2.1807394 |  |  |
|  |  | hsa-miR-412 | -2.191655988 |  |  |
|  |  | hsa-miR-569 | -2.204191524 |  |  |
|  |  | hsa-miR-549 | -2.206014069 |  |  |
|  |  | hsa-miR-875-3p | -2.210140126 |  |  |
|  |  | hsa-miR-653 | -2.236039712 |  |  |
|  |  | hsa-miR-146a* | -2.23699965 |  |  |
|  |  | hsa-miR-541* | -2.241827803 |  |  |
|  |  | hsa-miR-586 | -2.2469451 |  |  |
|  |  | hsa-miR-496 | -2.261062756 |  |  |
|  |  | hsa-miR-138-1* | -2.321294819 |  |  |
|  |  | hsa-miR-582-5p | -2.39754465 |  |  |
|  |  | hsa-miR-137 | -2.401836299 |  |  |
|  |  | hsa-miR-708* | -2.405171035 |  |  |
|  |  | hsa-miR-767-5p | -2.417614465 |  |  |
|  |  | hsa-miR-124* | -2.422367575 |  |  |
|  |  | hsa-miR-544 | -2.455496381 |  |  |
|  |  | hsa-miR-581 | -2.468147945 |  |  |
|  |  | hsa-miR-577 | -2.468436446 |  |  |
|  |  | hsa-miR-486-3p | -2.472335457 |  |  |
|  |  | hsa-miR-146b-3p | -2.494607338 |  |  |
|  |  | hsa-miR-208a | -2.535540367 |  |  |
|  |  | hsa-miR-621 | -2.548904621 |  |  |
|  |  | hsa-miR-96* | -2.56035251 |  |  |
|  |  | hsa-miR-374a* | -2.569053696 |  |  |
|  |  | hsa-miR-612 | -2.590798375 |  |  |
|  |  | hsa-miR-100* | -2.59082719 |  |  |
|  |  | hsa-miR-935 | -2.593303862 |  |  |
|  |  | hsa-miR-31* | -2.606762187 |  |  |
|  |  | hsa-miR-591 | -2.625195162 |  |  |
|  |  | hsa-miR-219-2-3p | -2.633718198 |  |  |
|  |  | hsa-miR-300 | -2.670857357 |  |  |
|  |  | hsa-miR-876-3p | -2.671579474 |  |  |
|  |  | hsa-miR-587 | -2.783745751 |  |  |
|  |  | hsa-miR-512-5p | -2.795653311 |  |  |
|  |  | hsa-miR-487a | -2.797156399 |  |  |
|  |  | hsa-miR-24-1* | -2.810438909 |  |  |
|  |  | hsa-miR-335 | -2.853129384 |  |  |
|  |  | hsa-miR-1 | -2.86010236 |  |  |
|  |  | hsa-miR-499-3p | -2.860595861 |  |  |
|  |  | hsa-miR-579 | -2.871924543 |  |  |
|  |  | hsa-miR-493 | -2.885599475 |  |  |
|  |  | hsa-miR-187 | -2.896112292 |  |  |
|  |  | hsa-miR-541 | -2.90934553 |  |  |
|  |  | hsa-miR-556-5p | -2.946772987 |  |  |
|  |  | hsa-miR-561 | -2.981322733 |  |  |
|  |  | hsa-miR-433 | -2.990868346 |  |  |
|  |  | hsa-miR-555 | -3.032911104 |  |  |
|  |  | hsa-miR-154* | -3.036244774 |  |  |
|  |  | hsa-miR-27a* | -3.036924602 |  |  |
|  |  | hsa-miR-452* | -3.059342755 |  |  |
|  |  | hsa-miR-588 | -3.123851756 |  |  |
|  |  | hsa-miR-296-3p | -3.162289019 |  |  |
|  |  | hsa-miR-593* | -3.166762075 |  |  |
|  |  | hsa-miR-297 | -3.189296021 |  |  |
|  |  | hsa-miR-380* | -3.191726787 |  |  |
|  |  | hsa-miR-624 | -3.285816725 |  |  |
|  |  | hsa-miR-191 | -3.375359006 |  |  |
|  |  | hsa-miR-600 | -3.415594827 |  |  |
|  |  | hsa-miR-938 | -3.451193993 |  |  |
|  |  | hsa-miR-24-2* | -3.544583342 |  |  |
|  |  | hsa-miR-323-5p | -3.610670231 |  |  |
|  |  | hsa-miR-429 | -3.64551897 |  |  |
|  |  | hsa-miR-1231 | -3.683180581 |  |  |
|  |  | hsa-miR-302d* | -3.687458439 |  |  |
|  |  | hsa-miR-922 | -3.702084873 |  |  |
|  |  | hsa-miR-208b | -3.953185184 |  |  |
|  |  | hsa-miR-517c | -3.965995152 |  |  |
|  |  | hsa-miR-620 | -4.046208821 |  |  |
|  |  | hsa-miR-941 | -4.210181152 |  |  |

Table S2 microRNA expression in extracellular vesicles of dendritic cell in GSE27997

| GSM692625 |  | GSM692626 |  |
| --- | --- | --- | --- |
| hsa-miR-335 | 9.973972523 | hsa-miR-335 | 10.99405471 |
| hsa-miR-21 | 9.195723059 | hsa-miR-21 | 9.586470134 |
| hsa-miR-630 | 9.045761996 | hsa-miR-630 | 8.145334218 |
| hsa-miR-638 | 7.370446463 | hsa-miR-223 | 6.875361069 |
| hsa-miR-223 | 6.762059365 | hsa-miR-575 | 6.769494321 |
| hsa-miR-342-3p | 6.760673958 | hsa-miR-342-3p | 6.734250684 |
| hsa-miR-575 | 6.644871407 | hsa-miR-940 | 6.496610714 |
| hsa-miR-1225-5p | 6.435425255 | hsa-miR-638 | 6.410640959 |
| hsa-miR-23a | 6.364290478 | hsa-miR-1234 | 6.347518386 |
| hsa-miR-940 | 6.226423905 | hsa-miR-1238 | 6.206478796 |
| hsa-miR-1234 | 5.945660579 | hsa-miR-23a | 6.189930696 |
| hsa-miR-1238 | 5.781138772 | hsa-miR-191* | 6.15221226 |
| hsa-miR-1228 | 5.75759534 | hsa-miR-1228 | 6.109222268 |
| hsa-miR-191* | 5.633293274 | hsa-miR-425* | 5.864416767 |
| hsa-miR-425* | 5.403015778 | hsa-miR-550 | 5.695503753 |
| hsa-miR-16 | 5.259364088 | hsa-miR-149 | 5.691997494 |
| hsa-miR-22 | 5.253002289 | hsa-let-7b* | 5.687142338 |
| hsa-miR-550 | 5.25270961 | hsa-miR-33b* | 5.660918968 |
| hsa-miR-33b* | 5.230599668 | hsa-miR-1225-3p | 5.613222244 |
| hsa-let-7b* | 5.229164335 | hsa-let-7f-1* | 5.611717685 |
| hsa-miR-24 | 5.227675949 | hsa-miR-563 | 5.527102642 |
| hsa-miR-149 | 5.226448364 | hsa-miR-766 | 5.45215743 |
| hsa-let-7f-1* | 5.204954187 | hsa-miR-1237 | 5.38582653 |
| hsa-miR-1225-3p | 5.186726422 | hsa-miR-1225-5p | 5.360742638 |
| hsa-miR-766 | 5.062426075 | hsa-miR-602 | 5.322126807 |
| hsa-miR-563 | 5.015361087 | hsa-miR-197 | 5.077012539 |
| hsa-miR-1237 | 4.986096811 | hsa-miR-16 | 5.065388122 |
| hsa-miR-574-5p | 4.89685951 | hsa-miR-24 | 5.020983613 |
| hsa-miR-602 | 4.830506774 | hsa-miR-22 | 5.02021811 |
| hsa-miR-197 | 4.814500508 | hsa-miR-27a | 4.999871115 |
| hsa-miR-939 | 4.769719847 | hsa-miR-933 | 4.952294688 |
| hsa-miR-107 | 4.765174795 | hsa-miR-142-3p | 4.916091737 |
| hsa-miR-150* | 4.757494415 | hsa-miR-92b | 4.822531061 |
| hsa-miR-27a | 4.72610457 | hsa-miR-1229 | 4.781759753 |
| hsa-miR-103 | 4.687836033 | hsa-miR-451 | 4.722902302 |
| hsa-miR-933 | 4.562179727 | hsa-miR-636 | 4.70712065 |
| hsa-miR-142-3p | 4.532555636 | hsa-miR-1227 | 4.6446046 |
| hsa-miR-92a | 4.504881737 | hsa-miR-129-3p | 4.581751423 |
| hsa-miR-30d | 4.428993286 | hsa-miR-30d | 4.574580814 |
| hsa-miR-92b | 4.403533853 | hsa-miR-129* | 4.544851479 |
| hsa-miR-1229 | 4.366019941 | hsa-miR-877* | 4.513945041 |
| hsa-miR-760 | 4.344124875 | hsa-miR-202 | 4.4409857 |
| hsa-miR-572 | 4.331057267 | hsa-miR-103 | 4.430095603 |
| hsa-miR-451 | 4.320739196 | hsa-miR-365 | 4.294022072 |
| hsa-let-7a | 4.318660739 | hsa-miR-634 | 4.292322916 |
| hsa-let-7f | 4.31105899 | hsa-miR-107 | 4.271463705 |
| hsa-miR-636 | 4.299690314 | hsa-miR-939 | 4.187708175 |
| hsa-miR-129-3p | 4.238882082 | hsa-let-7f | 4.16870544 |
| hsa-miR-1227 | 4.220127906 | hsa-miR-150* | 4.150498735 |
| hsa-miR-365 | 4.214858418 | hsa-miR-92a | 4.1088843 |
| hsa-miR-378 | 4.202161715 | hsa-miR-484 | 4.079465696 |
| hsa-miR-129* | 4.137184079 | hsa-miR-574-5p | 4.048036665 |
| hsa-miR-877* | 4.128433539 | hsa-let-7a | 4.045577327 |
| hsa-miR-634 | 3.92656531 | hsa-miR-665 | 4.023206447 |
| hsa-miR-188-5p | 3.875405443 | hsa-miR-767-3p | 3.999295608 |
| hsa-let-7b | 3.87328596 | hsa-miR-625* | 3.96020005 |
| hsa-miR-654-5p | 3.769314305 | hsa-miR-483-3p | 3.919021846 |
| hsa-miR-483-5p | 3.765689006 | hsa-miR-221 | 3.910763528 |
| hsa-miR-15a | 3.745286234 | hsa-miR-378 | 3.862397653 |
| hsa-miR-625* | 3.715643277 | hsa-miR-296-5p | 3.844381581 |
| hsa-miR-146b-5p | 3.668685712 | hsa-miR-18b* | 3.787078653 |
| hsa-miR-18b* | 3.664235704 | hsa-miR-142-5p | 3.776092927 |
| hsa-miR-409-3p | 3.632731883 | hsa-miR-409-3p | 3.76849235 |
| hsa-miR-767-3p | 3.608995329 | hsa-miR-671-3p | 3.749014686 |
| hsa-miR-484 | 3.583734383 | hsa-miR-671-5p | 3.692577971 |
| hsa-miR-221 | 3.583337513 | hsa-miR-15a | 3.668594764 |
| hsa-miR-483-3p | 3.580009441 | hsa-miR-324-3p | 3.667737952 |
| hsa-miR-296-5p | 3.546109402 | hsa-miR-654-5p | 3.60703206 |
| hsa-miR-665 | 3.544084305 | hsa-miR-423-5p | 3.585285941 |
| hsa-miR-32* | 3.454166301 | hsa-miR-146b-5p | 3.575323687 |
| hsa-miR-324-3p | 3.444763514 | hsa-miR-133a | 3.54808793 |
| hsa-miR-125a-3p | 3.436288226 | hsa-miR-19b | 3.52277435 |
| hsa-miR-671-3p | 3.375695606 | hsa-miR-26a | 3.463805329 |
| hsa-miR-26a | 3.368304931 | hsa-miR-26b | 3.43775293 |
| hsa-miR-425 | 3.281225941 | hsa-let-7b | 3.377676034 |
| hsa-miR-19b | 3.259570579 | hsa-miR-155 | 3.230384078 |
| hsa-let-7i | 3.17974504 | hsa-miR-572 | 3.214320338 |
| hsa-miR-133a | 3.174249839 | hsa-miR-299-5p | 3.196873378 |
| hsa-miR-29a | 3.172499153 | hsa-let-7i | 3.150645997 |
| hsa-miR-132 | 3.172073926 | hsa-miR-425 | 3.030918729 |
| hsa-miR-423-5p | 3.102094056 | hsa-let-7g | 3.029925189 |
| hsa-miR-15b | 3.091751209 | hsa-miR-133b | 2.994728929 |
| hsa-miR-513a-5p | 3.044612861 | hsa-miR-29b | 2.978517359 |
| hsa-miR-26b | 3.023696246 | hsa-miR-15b | 2.970046935 |
| hsa-miR-1236 | 3.022550692 | hsa-miR-29a | 2.952378496 |
| hsa-miR-671-5p | 3.020796806 | hsa-miR-371-5p | 2.916200156 |
| hsa-let-7g | 2.991889396 | hsa-miR-1233 | 2.915545523 |
| hsa-miR-299-5p | 2.981022776 | hsa-miR-146a | 2.838158811 |
| hsa-miR-146a | 2.956541876 | hsa-miR-20a | 2.808284892 |
| hsa-miR-921 | 2.91976497 | hsa-miR-513a-5p | 2.786691485 |
| hsa-miR-134 | 2.849730478 | hsa-miR-188-5p | 2.783823364 |
| hsa-miR-155 | 2.84372139 | hsa-miR-125a-3p | 2.783607958 |
| hsa-miR-142-5p | 2.826347079 | hsa-miR-760 | 2.769953215 |
| hsa-miR-181a | 2.792360847 | hsa-miR-32* | 2.669814685 |
| hsa-miR-181a-2* | 2.692827476 | hsa-miR-424 | 2.657323896 |
| hsa-miR-494 | 2.68818973 | hsa-miR-19a | 2.553027248 |
| hsa-miR-25 | 2.660667187 | hsa-miR-328 | 2.5297459 |
| hsa-miR-561 | 2.605378495 | hsa-miR-30b | 2.505639917 |
| hsa-miR-27b | 2.577735573 | hsa-miR-518b | 2.490590493 |
| hsa-miR-922 | 2.537937193 | hsa-miR-937 | 2.44956257 |
| hsa-miR-125b | 2.514151435 | hsa-miR-181a | 2.434379798 |
| hsa-miR-93 | 2.509514878 | hsa-miR-483-5p | 2.409042497 |
| hsa-let-7c | 2.496892415 | hsa-miR-30e | 2.375755274 |
| hsa-miR-361-5p | 2.494769212 | hsa-let-7d | 2.366192491 |
| hsa-let-7d | 2.490703362 | hsa-miR-25 | 2.353941162 |
| hsa-miR-767-5p | 2.490505573 | hsa-miR-1236 | 2.332200974 |
| hsa-miR-20a | 2.457132183 | hsa-miR-132 | 2.322818834 |
| hsa-miR-29b | 2.450543565 | hsa-miR-331-3p | 2.299008291 |
| hsa-miR-133b | 2.432439698 | hsa-miR-126 | 2.255134044 |
| hsa-miR-331-3p | 2.407469023 | hsa-miR-584 | 2.239155412 |
| hsa-miR-30b | 2.399219732 | hsa-miR-335* | 2.209195287 |
| hsa-miR-371-5p | 2.385788539 | hsa-miR-498 | 2.159793454 |
| hsa-miR-126 | 2.371433974 | hsa-miR-30c | 2.15736325 |
| hsa-miR-30c | 2.359443617 | hsa-miR-519d | 2.150920442 |
| hsa-miR-29a* | 2.339373307 | hsa-miR-361-5p | 2.150520245 |
| hsa-miR-140-3p | 2.333571418 | hsa-miR-29c | 2.133497168 |
| hsa-miR-601 | 2.328050086 | hsa-let-7d* | 2.122578519 |
| hsa-miR-886-5p | 2.325384954 | hsa-miR-10b* | 2.083368602 |
| hsa-miR-1233 | 2.283164694 | hsa-miR-17 | 2.044891625 |
| hsa-miR-424 | 2.270643302 | hsa-miR-574-3p | 2.03827849 |
| hsa-miR-99b* | 2.257100524 | hsa-miR-212 | 2.03413748 |
| hsa-miR-328 | 2.218653783 | hsa-miR-423-3p | 2.032179067 |
| hsa-miR-610 | 2.203429611 | hsa-miR-548c-5p | 1.974420105 |
| hsa-miR-937 | 2.20315659 | hsa-miR-204 | 1.969579626 |
| hsa-miR-23b | 2.196972654 | hsa-miR-140-3p | 1.952817299 |
| hsa-miR-574-3p | 2.179346586 | hsa-miR-595 | 1.93786 |
| hsa-miR-448 | 2.144667576 | hsa-miR-99b | 1.88982844 |
| hsa-miR-582-3p | 2.110949983 | hsa-miR-548c-3p | 1.859024822 |
| hsa-miR-212 | 2.104537664 | hsa-miR-551b | 1.856298037 |
| hsa-miR-889 | 2.104145842 | hsa-miR-337-3p | 1.850715537 |
| hsa-miR-595 | 2.091107928 | hsa-miR-886-3p | 1.843871091 |
| hsa-miR-26a-2* | 2.069754822 | hsa-miR-582-3p | 1.829325636 |
| hsa-miR-326 | 2.061177971 | hsa-miR-885-5p | 1.826611517 |
| hsa-miR-548c-3p | 2.055577727 | hsa-miR-211 | 1.822196304 |
| hsa-miR-548d-5p | 2.051018609 | hsa-miR-192 | 1.821352803 |
| hsa-miR-493 | 2.048128451 | hsa-miR-505 | 1.80862601 |
| hsa-miR-329 | 2.040529793 | hsa-miR-338-3p | 1.788230117 |
| hsa-miR-887 | 2.014087697 | hsa-miR-23b | 1.771682615 |
| hsa-miR-17 | 2.00159094 | hsa-miR-186 | 1.768570449 |
| hsa-let-7d* | 1.995091306 | hsa-miR-448 | 1.768029244 |
| hsa-miR-335* | 1.974527912 | hsa-miR-140-5p | 1.764755922 |
| hsa-miR-632 | 1.942410479 | hsa-miR-556-3p | 1.763686962 |
| hsa-miR-885-5p | 1.93382514 | hsa-miR-329 | 1.762293409 |
| hsa-miR-135a* | 1.931962857 | hsa-let-7c | 1.757956203 |
| hsa-miR-421 | 1.891011824 | hsa-let-7f-2* | 1.722498643 |
| hsa-miR-628-5p | 1.884046331 | hsa-miR-195* | 1.685365248 |
| hsa-miR-486-5p | 1.863079278 | hsa-miR-487b | 1.652848018 |
| hsa-miR-29c | 1.858087274 | hsa-miR-454* | 1.649166786 |
| hsa-miR-187* | 1.851535487 | hsa-miR-548a-5p | 1.625247988 |
| hsa-miR-186 | 1.822098497 | hsa-miR-326 | 1.623217111 |
| hsa-miR-423-3p | 1.79074228 | hsa-miR-758 | 1.606289777 |
| hsa-miR-30e | 1.786202964 | hsa-miR-29c* | 1.586696708 |
| hsa-miR-765 | 1.75695496 | hsa-miR-15b* | 1.583949881 |
| hsa-miR-454* | 1.7497921 | hsa-miR-26a-2* | 1.580918766 |
| hsa-miR-26a-1* | 1.709693041 | hsa-miR-613 | 1.577750871 |
| hsa-miR-28-5p | 1.682664664 | hsa-miR-106b | 1.568832124 |
| hsa-miR-301b | 1.68021288 | hsa-miR-34a | 1.564747827 |
| hsa-miR-450b-5p | 1.678133701 | hsa-miR-371-3p | 1.528272389 |
| hsa-miR-646 | 1.666703538 | hsa-miR-126* | 1.521545624 |
| hsa-miR-520g | 1.660348656 | hsa-miR-421 | 1.510799807 |
| hsa-miR-631 | 1.613664627 | hsa-miR-888* | 1.504483427 |
| hsa-miR-607 | 1.610682432 | hsa-miR-624* | -1.50302322 |
| hsa-miR-567 | 1.600949874 | hsa-miR-578 | -1.50715644 |
| hsa-miR-551b | 1.597486138 | hsa-miR-802 | -1.513613039 |
| hsa-miR-519d | 1.595666255 | hsa-miR-297 | -1.532522884 |
| hsa-miR-186* | 1.587612612 | hsa-miR-502-3p | -1.543004359 |
| hsa-miR-519e | 1.587505195 | hsa-miR-124* | -1.553696293 |
| hsa-miR-34a | 1.581492089 | hsa-miR-552 | -1.563399404 |
| hsa-miR-802 | 1.574720878 | hsa-miR-516a-3p | -1.569696823 |
| hsa-miR-566 | 1.574454903 | hsa-miR-642 | -1.589279022 |
| hsa-miR-548b-3p | 1.518843137 | hsa-miR-652 | -1.593107025 |
| hsa-miR-492 | 1.511342946 | hsa-miR-649 | -1.603821366 |
| hsa-miR-557 | 1.504656856 | hsa-miR-139-5p | -1.644487361 |
| hsa-miR-106b* | -1.513240171 | hsa-miR-31* | -1.650453961 |
| hsa-miR-203 | -1.525309236 | hsa-miR-597 | -1.659092169 |
| hsa-miR-193a-3p | -1.543184565 | hsa-miR-890 | -1.687526625 |
| hsa-miR-151-5p | -1.545186661 | hsa-miR-626 | -1.722760615 |
| hsa-miR-181d | -1.564036974 | hsa-miR-876-3p | -1.733532565 |
| hsa-miR-520a-5p | -1.572571797 | hsa-miR-10b | -1.73910442 |
| hsa-miR-518d-3p | -1.579422474 | hsa-miR-509-3-5p | -1.741146157 |
| hsa-miR-141* | -1.57999882 | hsa-miR-198 | -1.761812367 |
| hsa-miR-571 | -1.645470493 | hsa-miR-9 | -1.764177185 |
| hsa-miR-542-3p | -1.647117968 | hsa-miR-604 | -1.766551622 |
| hsa-miR-508-3p | -1.647977888 | hsa-miR-541 | -1.793416806 |
| hsa-miR-429 | -1.648763163 | hsa-miR-629* | -1.804982831 |
| hsa-miR-24-1* | -1.655076753 | hsa-miR-377 | -1.823534203 |
| hsa-miR-210 | -1.671475117 | hsa-miR-302d | -1.827733227 |
| hsa-miR-31 | -1.677921287 | hsa-miR-20b* | -1.835748635 |
| hsa-miR-130a* | -1.698715578 | hsa-miR-195 | -1.848109897 |
| hsa-miR-34c-3p | -1.701551339 | hsa-miR-542-3p | -1.854453266 |
| hsa-miR-10b* | -1.718992825 | hsa-miR-181c | -1.854734238 |
| hsa-miR-206 | -1.728500944 | hsa-miR-708* | -1.864528995 |
| hsa-miR-564 | -1.731138431 | hsa-miR-148b | -1.867279607 |
| hsa-miR-181c* | -1.74504616 | hsa-miR-579 | -1.922340561 |
| hsa-miR-526b* | -1.790294706 | hsa-miR-587 | -1.943732205 |
| hsa-miR-28-3p | -1.791567326 | hsa-miR-589* | -1.960101008 |
| hsa-miR-558 | -1.801316251 | hsa-miR-300 | -1.973802948 |
| hsa-miR-199a-5p | -1.837762011 | hsa-miR-623 | -1.978616725 |
| hsa-miR-944 | -1.854654577 | hsa-let-7i* | -1.987778439 |
| hsa-miR-138 | -1.896805737 | hsa-miR-92a-1* | -1.997702199 |
| hsa-miR-544 | -1.905573484 | hsa-miR-411 | -2.00071498 |
| hsa-miR-181b | -1.924127841 | hsa-miR-508-5p | -2.00408476 |
| hsa-miR-30e* | -1.932177501 | hsa-miR-612 | -2.008258538 |
| hsa-miR-545* | -1.932205352 | hsa-miR-382 | -2.02262411 |
| hsa-miR-596 | -1.933098486 | hsa-miR-380* | -2.051836411 |
| hsa-miR-412 | -1.934641686 | hsa-miR-146b-3p | -2.056090382 |
| hsa-miR-506 | -1.937738515 | hsa-miR-518e* | -2.082286847 |
| hsa-miR-621 | -1.94652306 | hsa-miR-181d | -2.093060665 |
| hsa-miR-593* | -1.949513003 | hsa-miR-378* | -2.142915441 |
| hsa-miR-454 | -1.954087378 | hsa-miR-372 | -2.146774729 |
| hsa-miR-96* | -1.961641274 | hsa-miR-301b | -2.14719903 |
| hsa-miR-769-3p | -1.963119722 | hsa-miR-101* | -2.174185622 |
| hsa-miR-100 | -1.964807093 | hsa-miR-936 | -2.174710432 |
| hsa-miR-376a | -1.966217362 | hsa-miR-19b-1* | -2.176818273 |
| hsa-miR-378* | -1.979447034 | hsa-miR-614 | -2.190285829 |
| hsa-miR-302d | -1.980978635 | hsa-miR-130a* | -2.206953406 |
| hsa-miR-214* | -1.98764015 | hsa-miR-596 | -2.211478063 |
| hsa-miR-491-5p | -1.998129776 | hsa-miR-148a* | -2.214157018 |
| hsa-miR-410 | -2.009244662 | hsa-miR-373 | -2.229208781 |
| hsa-miR-373 | -2.013522563 | hsa-miR-363* | -2.231266441 |
| hsa-let-7e | -2.020355244 | hsa-miR-181c* | -2.233731012 |
| hsa-miR-9 | -2.020961651 | hsa-miR-616* | -2.238538342 |
| hsa-miR-589 | -2.022519178 | hsa-miR-499-5p | -2.240267941 |
| hsa-miR-649 | -2.040836679 | hsa-miR-431 | -2.242634976 |
| hsa-miR-324-5p | -2.049847604 | hsa-miR-367* | -2.244801385 |
| hsa-miR-516b | -2.050782384 | hsa-miR-33a | -2.246425742 |
| hsa-miR-517* | -2.053194739 | hsa-miR-523 | -2.255188449 |
| hsa-miR-876-3p | -2.067812413 | hsa-miR-511 | -2.259156469 |
| hsa-miR-526b | -2.067976164 | hsa-miR-592 | -2.282968603 |
| hsa-miR-514 | -2.082669186 | hsa-miR-376c | -2.28541772 |
| hsa-miR-512-5p | -2.094126115 | hsa-miR-934 | -2.295220524 |
| hsa-miR-379* | -2.104921474 | hsa-miR-643 | -2.307012631 |
| hsa-miR-518e | -2.115245044 | hsa-miR-138 | -2.308830771 |
| hsa-miR-500 | -2.126414244 | hsa-miR-580 | -2.318397182 |
| hsa-miR-618 | -2.136192585 | hsa-miR-223* | -2.329432284 |
| hsa-miR-323-3p | -2.177574106 | hsa-miR-139-3p | -2.341475613 |
| hsa-miR-549 | -2.182218997 | hsa-miR-564 | -2.343733256 |
| hsa-miR-105* | -2.188639908 | hsa-miR-877 | -2.354975461 |
| hsa-miR-101 | -2.195151911 | hsa-miR-510 | -2.381989836 |
| hsa-miR-512-3p | -2.197801948 | hsa-miR-203 | -2.411944608 |
| hsa-miR-589* | -2.19792623 | hsa-miR-323-5p | -2.41247088 |
| hsa-miR-183 | -2.19821373 | hsa-miR-554 | -2.437896904 |
| hsa-miR-551b* | -2.210664081 | hsa-miR-944 | -2.467447886 |
| hsa-miR-382 | -2.218280783 | hsa-miR-941 | -2.469123396 |
| hsa-miR-938 | -2.246710427 | hsa-miR-200a | -2.474768681 |
| hsa-miR-661 | -2.250532773 | hsa-miR-553 | -2.485532297 |
| hsa-miR-507 | -2.252512259 | hsa-miR-514 | -2.492270215 |
| hsa-miR-383 | -2.26030978 | hsa-miR-490-3p | -2.514434265 |
| hsa-miR-658 | -2.260526034 | hsa-miR-410 | -2.517971079 |
| hsa-miR-576-3p | -2.295104183 | hsa-let-7c* | -2.616513052 |
| hsa-miR-590-3p | -2.315205591 | hsa-miR-769-3p | -2.627797538 |
| hsa-miR-523 | -2.31941687 | hsa-miR-218-1* | -2.628462094 |
| hsa-miR-339-3p | -2.319609111 | hsa-miR-412 | -2.62964391 |
| hsa-miR-200a | -2.369991939 | hsa-miR-208a | -2.644384487 |
| hsa-miR-592 | -2.374640369 | hsa-miR-182* | -2.66949419 |
| hsa-miR-637 | -2.400706868 | hsa-miR-499-3p | -2.672747646 |
| hsa-miR-936 | -2.420544895 | hsa-miR-147 | -2.684972137 |
| hsa-miR-651 | -2.423188811 | hsa-miR-424* | -2.688782596 |
| hsa-miR-182 | -2.428160906 | hsa-miR-943 | -2.723921086 |
| hsa-miR-17* | -2.453434011 | hsa-miR-182 | -2.747396907 |
| hsa-miR-146b-3p | -2.462915002 | hsa-miR-518e | -2.754991767 |
| hsa-miR-493* | -2.494763508 | hsa-miR-302c* | -2.767627649 |
| hsa-miR-890 | -2.496448504 | hsa-miR-637 | -2.779046665 |
| hsa-miR-129-5p | -2.512518814 | hsa-miR-338-5p | -2.781917183 |
| hsa-miR-218-1* | -2.522576751 | hsa-miR-505* | -2.783000866 |
| hsa-miR-224 | -2.548348628 | hsa-miR-449b | -2.787864727 |
| hsa-miR-30a | -2.555457327 | hsa-miR-935 | -2.805675246 |
| hsa-miR-34b* | -2.557799892 | hsa-miR-194 | -2.810549851 |
| hsa-miR-124* | -2.60035475 | hsa-miR-99a* | -2.811910571 |
| hsa-miR-1226 | -2.609240036 | hsa-miR-588 | -2.850560138 |
| hsa-miR-569 | -2.615541263 | hsa-miR-569 | -2.858965448 |
| hsa-miR-614 | -2.677826479 | hsa-miR-206 | -2.863322638 |
| hsa-miR-591 | -2.681883167 | hsa-miR-129-5p | -2.872606591 |
| hsa-miR-541* | -2.693824367 | hsa-miR-508-3p | -2.875158061 |
| hsa-miR-648 | -2.713004571 | hsa-miR-429 | -2.881039999 |
| hsa-miR-340 | -2.734308392 | hsa-miR-194* | -2.914494961 |
| hsa-miR-524-5p | -2.735277661 | hsa-miR-558 | -2.930381678 |
| hsa-miR-642 | -2.737974305 | hsa-miR-590-3p | -2.931969885 |
| hsa-miR-92a-2* | -2.763630504 | hsa-miR-509-5p | -2.938734688 |
| hsa-miR-24-2* | -2.76474766 | hsa-miR-153 | -2.94533974 |
| hsa-miR-194 | -2.773487279 | hsa-miR-370 | -2.946661364 |
| hsa-miR-624 | -2.780833938 | hsa-miR-337-5p | -2.972432468 |
| hsa-miR-219-1-3p | -2.84128886 | hsa-miR-493 | -2.972966999 |
| hsa-miR-453 | -2.885116894 | hsa-miR-99b* | -2.977337713 |
| hsa-miR-1231 | -2.885130722 | hsa-miR-513a-3p | -2.983442837 |
| hsa-miR-325 | -2.895432435 | hsa-miR-141 | -3.008499942 |
| hsa-miR-363* | -2.911198109 | hsa-miR-571 | -3.025379965 |
| hsa-miR-92a-1* | -2.914441916 | hsa-miR-645 | -3.039642877 |
| hsa-miR-99a* | -2.92523742 | hsa-miR-345 | -3.041526546 |
| hsa-miR-181c | -2.93172586 | hsa-miR-561 | -3.0548336 |
| hsa-miR-141 | -2.931982238 | hsa-miR-216b | -3.058822701 |
| hsa-miR-188-3p | -2.946190377 | hsa-miR-190 | -3.061243786 |
| hsa-miR-148a* | -2.958337242 | hsa-miR-185* | -3.075370933 |
| hsa-miR-517a | -2.967746863 | hsa-miR-9* | -3.08378494 |
| hsa-miR-875-3p | -2.969268651 | hsa-miR-628-3p | -3.12585546 |
| hsa-miR-143 | -3.025304921 | hsa-miR-137 | -3.149192029 |
| hsa-miR-374a* | -3.027690208 | hsa-miR-562 | -3.154351416 |
| hsa-miR-338-5p | -3.036766822 | hsa-miR-149* | -3.179089465 |
| hsa-miR-554 | -3.045570062 | hsa-miR-891a | -3.199320265 |
| hsa-miR-516a-3p | -3.09720058 | hsa-miR-744 | -3.23306245 |
| hsa-miR-30a* | -3.140993489 | hsa-miR-938 | -3.254045841 |
| hsa-miR-302c* | -3.146747662 | hsa-miR-379 | -3.261490368 |
| hsa-miR-620 | -3.153343625 | hsa-miR-452* | -3.268690521 |
| hsa-miR-337-5p | -3.155719774 | hsa-miR-200c* | -3.271686366 |
| hsa-miR-299-3p | -3.158819462 | hsa-miR-125b-1* | -3.280249176 |
| hsa-miR-487a | -3.175263267 | hsa-miR-376a | -3.284906832 |
| hsa-miR-643 | -3.178917514 | hsa-miR-921 | -3.28858746 |
| hsa-miR-373* | -3.179882846 | hsa-miR-431* | -3.307508016 |
| hsa-miR-616 | -3.191074608 | hsa-miR-589 | -3.320234081 |
| hsa-miR-380* | -3.19117985 | hsa-miR-433 | -3.33540977 |
| hsa-miR-510 | -3.197855518 | hsa-miR-1228* | -3.357389676 |
| hsa-miR-296-3p | -3.229344806 | hsa-miR-217 | -3.360125406 |
| hsa-miR-217 | -3.241006941 | hsa-miR-219-1-3p | -3.371569558 |
| hsa-miR-599 | -3.245398202 | hsa-miR-504 | -3.37901879 |
| hsa-miR-214 | -3.255229241 | hsa-miR-487a | -3.386118317 |
| hsa-miR-508-5p | -3.255938961 | hsa-miR-453 | -3.396966793 |
| hsa-miR-208a | -3.26032594 | hsa-miR-452 | -3.420069992 |
| hsa-miR-193a-5p | -3.271802494 | hsa-miR-302d* | -3.423556227 |
| hsa-miR-16-1* | -3.279240568 | hsa-miR-920 | -3.441248029 |
| hsa-miR-139-3p | -3.284044682 | hsa-miR-17* | -3.486321266 |
| hsa-miR-381 | -3.303389518 | hsa-miR-593* | -3.486420585 |
| hsa-miR-216b | -3.349324532 | hsa-miR-375 | -3.507524965 |
| hsa-miR-604 | -3.356334587 | hsa-miR-600 | -3.517799003 |
| hsa-miR-509-5p | -3.359749204 | hsa-miR-200a* | -3.532443806 |
| hsa-miR-205 | -3.361009368 | hsa-miR-135b* | -3.533903459 |
| hsa-miR-424* | -3.3706673 | hsa-miR-325 | -3.553189267 |
| hsa-miR-379 | -3.371393346 | hsa-miR-130a | -3.573907235 |
| hsa-miR-9* | -3.381504075 | hsa-miR-216a | -3.578417968 |
| hsa-miR-330-3p | -3.429002956 | hsa-miR-500 | -3.584471128 |
| hsa-miR-568 | -3.436394193 | hsa-miR-187* | -3.60553143 |
| hsa-miR-151-3p | -3.438779892 | hsa-miR-373* | -3.633509557 |
| hsa-miR-497 | -3.475579548 | hsa-miR-509-3p | -3.641040383 |
| hsa-miR-300 | -3.477761118 | hsa-miR-298 | -3.650062124 |
| hsa-miR-675 | -3.499758953 | hsa-miR-551b* | -3.660393334 |
| hsa-miR-490-3p | -3.501811305 | hsa-miR-96* | -3.667909422 |
| hsa-miR-891a | -3.514910574 | hsa-miR-454 | -3.695400719 |
| hsa-miR-920 | -3.522990431 | hsa-miR-330-3p | -3.719763044 |
| hsa-miR-376c | -3.550287617 | hsa-miR-639 | -3.724029869 |
| hsa-miR-200c | -3.570334424 | hsa-miR-28-3p | -3.758467473 |
| hsa-miR-148b | -3.589463028 | hsa-miR-183* | -3.813407776 |
| hsa-miR-198 | -3.589858941 | hsa-miR-620 | -3.818597621 |
| hsa-miR-1 | -3.61417155 | hsa-miR-196a* | -3.824192181 |
| hsa-miR-200b | -3.640691357 | hsa-miR-24-2* | -3.83131727 |
| hsa-miR-23a* | -3.679901593 | hsa-miR-576-3p | -3.856567331 |
| hsa-miR-452 | -3.698560767 | hsa-let-7g* | -3.857078798 |
| hsa-miR-639 | -3.720645005 | hsa-miR-573 | -3.890025718 |
| hsa-miR-152 | -3.754888091 | hsa-miR-885-3p | -3.902816164 |
| hsa-miR-208b | -3.784766266 | hsa-miR-152 | -3.932773851 |
| hsa-miR-137 | -3.787182519 | hsa-miR-675 | -3.941645267 |
| hsa-miR-509-3p | -3.797157806 | hsa-miR-193b* | -3.949298935 |
| hsa-miR-573 | -3.84312327 | hsa-miR-490-5p | -3.970243912 |
| hsa-miR-490-5p | -3.879977694 | hsa-miR-619 | -3.973813583 |
| hsa-miR-193b* | -3.939098704 | hsa-miR-339-5p | -3.988994449 |
| hsa-miR-934 | -3.948330464 | hsa-miR-214 | -4.019355691 |
| hsa-miR-153 | -3.968800606 | hsa-miR-27a* | -4.034988491 |
| hsa-miR-497* | -3.979049386 | hsa-miR-16-1* | -4.040332954 |
| hsa-miR-375 | -4.025142441 | hsa-miR-485-5p | -4.050185535 |
| hsa-miR-885-3p | -4.203867937 | hsa-miR-127-5p | -4.066383598 |
| hsa-miR-370 | -4.257133412 | hsa-miR-497* | -4.06832238 |
| hsa-miR-433 | -4.309342179 | hsa-miR-339-3p | -4.119973607 |
| hsa-miR-92b* | -4.35936623 | hsa-miR-200b* | -4.129210635 |
| hsa-miR-452* | -4.391167329 | hsa-miR-23a* | -4.157486881 |
|  |  | hsa-miR-516b | -4.184125569 |
|  |  | hsa-miR-106b* | -4.194827833 |
|  |  | hsa-miR-648 | -4.210459474 |
|  |  | hsa-miR-616 | -4.283787482 |
|  |  | hsa-miR-92b* | -4.298475895 |
|  |  | hsa-miR-875-3p | -4.31680951 |
|  |  | hsa-miR-1226* | -4.317245905 |
|  |  | hsa-miR-138-2* | -4.489533113 |
|  |  | hsa-miR-1231 | -4.637526158 |
|  |  |  |  |

Table S3microRNA expression in extracellular vesicles of B lymphocyte in GSE27997

| GSM692617 |  | GSM692618 |  | GSM692629 |  |
| --- | --- | --- | --- | --- | --- |
| hsa-miR-21 | 9.8955658 | hsa-miR-575 | 14.1108365 | hsa-miR-575 | 13.62146757 |
| hsa-miR-575 | 9.800984911 | hsa-miR-21 | 11.99070527 | hsa-miR-630 | 11.56514234 |
| hsa-miR-451 | 9.747651939 | hsa-miR-1225-5p | 11.32300496 | hsa-miR-1225-5p | 10.62007906 |
| hsa-miR-483-5p | 9.736539108 | hsa-miR-630 | 10.83335965 | hsa-miR-125a-3p | 10.22255088 |
| hsa-miR-638 | 9.681348527 | hsa-miR-19b | 10.78634869 | hsa-miR-638 | 10.13628219 |
| hsa-miR-1225-5p | 9.54743667 | hsa-miR-20a | 10.70894194 | hsa-miR-21 | 9.58249374 |
| hsa-miR-193b* | 9.389173868 | hsa-miR-16 | 10.41108585 | hsa-miR-939 | 9.015754839 |
| hsa-miR-630 | 9.282273561 | hsa-miR-125a-3p | 10.34147785 | hsa-miR-513a-5p | 8.290288055 |
| hsa-miR-10a* | 9.143351611 | hsa-miR-638 | 10.23737666 | hsa-miR-16 | 8.180193836 |
| hsa-miR-20a | 9.120027977 | hsa-miR-939 | 9.803212208 | hsa-miR-19b | 7.90097669 |
| hsa-miR-188-5p | 8.915735686 | hsa-miR-574-5p | 9.767344166 | hsa-miR-20a | 7.670070009 |
| hsa-miR-16 | 8.211747975 | hsa-miR-17 | 9.638728769 | hsa-miR-574-5p | 7.626036591 |
| hsa-miR-17 | 8.186912731 | hsa-let-7f | 9.511379413 | hsa-miR-92a | 7.306941231 |
| hsa-miR-103 | 8.087761449 | hsa-miR-106b | 9.505996867 | hsa-miR-483-5p | 7.284091654 |
| hsa-miR-939 | 8.044614448 | hsa-miR-19a | 9.42359061 | hsa-miR-155 | 7.09736436 |
| hsa-miR-19b | 7.993744524 | hsa-miR-155 | 9.376834142 | hsa-let-7f | 7.094202975 |
| hsa-miR-874 | 7.970321484 | hsa-miR-198 | 9.367422834 | hsa-miR-17 | 6.984898243 |
| hsa-miR-15b | 7.85426951 | hsa-miR-92a | 9.14210443 | hsa-miR-150* | 6.913880633 |
| hsa-miR-92a | 7.810532219 | hsa-miR-29a | 9.130349647 | hsa-miR-19a | 6.865032803 |
| hsa-miR-193a-5p | 7.78058258 | hsa-miR-25 | 9.106992734 | hsa-miR-106b | 6.798239765 |
| hsa-let-7f | 7.716378821 | hsa-let-7a | 9.07961263 | hsa-miR-107 | 6.788590504 |
| hsa-miR-29a | 7.713224779 | hsa-miR-15b | 9.061831974 | hsa-miR-663 | 6.614558626 |
| hsa-let-7a | 7.706177029 | hsa-miR-658 | 8.850674789 | hsa-miR-134 | 6.605351947 |
| hsa-miR-25 | 7.638352923 | hsa-miR-107 | 8.746889287 | hsa-miR-654-5p | 6.557674049 |
| hsa-miR-654-5p | 7.597349355 | hsa-let-7g | 8.690859758 | hsa-miR-15b | 6.4738555 |
| hsa-miR-125a-3p | 7.589369536 | hsa-miR-93 | 8.616824071 | hsa-miR-29a | 6.455721755 |
| hsa-miR-155 | 7.401324408 | hsa-miR-142-3p | 8.596823753 | hsa-miR-25 | 6.424615693 |
| hsa-miR-106b | 7.352381136 | hsa-miR-324-3p | 8.564493077 | hsa-miR-940 | 6.393259351 |
| hsa-miR-19a | 7.319020347 | hsa-miR-146a | 8.463204808 | hsa-let-7a | 6.36639179 |
| hsa-miR-494 | 7.217377382 | hsa-miR-103 | 8.340361558 | hsa-miR-1224-5p | 6.344809533 |
| hsa-miR-424* | 7.130880902 | hsa-miR-451 | 8.313734993 | hsa-miR-188-5p | 6.342040841 |
| hsa-miR-142-3p | 6.999211812 | hsa-miR-188-5p | 8.202810466 | hsa-miR-146a | 6.338376418 |
| hsa-miR-107 | 6.750144861 | hsa-miR-513a-5p | 7.991346415 | hsa-miR-142-3p | 6.24595696 |
| hsa-let-7g | 6.738518004 | hsa-miR-425 | 7.981805022 | hsa-miR-494 | 6.192436356 |
| hsa-miR-93 | 6.68211387 | hsa-let-7d | 7.861189158 | hsa-miR-93 | 6.172545895 |
| hsa-miR-1224-5p | 6.614961575 | hsa-miR-20b | 7.837444481 | hsa-miR-32* | 6.136320015 |
| hsa-miR-20b | 6.613102809 | hsa-miR-30e | 7.824386843 | hsa-miR-451 | 6.073418918 |
| hsa-miR-223 | 6.61134148 | hsa-miR-193b | 7.818791528 | hsa-let-7g | 6.070809658 |
| hsa-miR-324-3p | 6.468952534 | hsa-miR-30d | 7.591672603 | hsa-miR-324-3p | 6.001541459 |
| hsa-miR-671-5p | 6.441919161 | hsa-miR-15a | 7.585507965 | hsa-miR-125b-1* | 5.993787366 |
| hsa-let-7d | 6.422900511 | hsa-miR-210 | 7.58257536 | hsa-miR-20b | 5.966100009 |
| hsa-miR-1226* | 6.363528921 | hsa-let-7i | 7.572758832 | hsa-miR-765 | 5.8421023 |
| hsa-miR-452 | 6.321739313 | hsa-miR-487b | 7.538371965 | hsa-miR-103 | 5.825640669 |
| hsa-miR-513a-5p | 6.314854413 | hsa-miR-422a | 7.492191643 | hsa-miR-15a | 5.787751151 |
| hsa-miR-134 | 6.291146092 | hsa-miR-134 | 7.439212057 | hsa-miR-1228 | 5.7497436 |
| hsa-miR-29b | 6.126816927 | hsa-miR-30b | 7.392876467 | hsa-miR-365 | 5.724344736 |
| hsa-miR-198 | 6.096821052 | hsa-miR-494 | 7.293025323 | hsa-miR-210 | 5.674641566 |
| hsa-miR-425 | 6.024227991 | hsa-miR-516a-5p | 7.267384143 | hsa-miR-671-5p | 5.647418082 |
| hsa-miR-572 | 5.989721141 | hsa-miR-365 | 7.207201856 | hsa-miR-30d | 5.632158877 |
| hsa-miR-193b | 5.975553307 | hsa-miR-654-5p | 7.204609464 | hsa-miR-513b | 5.527323201 |
| hsa-let-7i | 5.928036525 | hsa-miR-29b | 7.169402685 | hsa-miR-135a* | 5.523871957 |
| hsa-miR-15a | 5.916317477 | hsa-miR-423-5p | 7.139650942 | hsa-miR-1234 | 5.513939296 |
| hsa-miR-210 | 5.911167717 | hsa-miR-483-5p | 7.118782469 | hsa-miR-26b | 5.496630347 |
| hsa-miR-135a* | 5.897963669 | hsa-miR-18a | 7.058052761 | hsa-miR-30e | 5.370983232 |
| hsa-miR-146a | 5.798617026 | hsa-miR-125b-1* | 7.014638816 | hsa-miR-1238 | 5.365167527 |
| hsa-miR-150* | 5.766310378 | hsa-miR-135a* | 6.956917988 | hsa-miR-877 | 5.358279797 |
| hsa-miR-658 | 5.71575948 | hsa-miR-671-5p | 6.949501361 | hsa-miR-425 | 5.331028751 |
| hsa-miR-30b | 5.681513092 | hsa-miR-9* | 6.923416669 | hsa-miR-595 | 5.281412936 |
| hsa-miR-892b | 5.654881802 | hsa-miR-29c | 6.907274575 | hsa-miR-423-5p | 5.264389882 |
| hsa-miR-513b | 5.652876156 | hsa-miR-1224-5p | 6.890140858 | hsa-miR-30b | 5.188999934 |
| hsa-miR-423-5p | 5.648542207 | hsa-miR-150* | 6.807666897 | hsa-let-7d | 5.187542603 |
| hsa-miR-500 | 5.628140649 | hsa-miR-148a | 6.770653573 | hsa-miR-191* | 5.171891545 |
| hsa-miR-365 | 5.623031046 | hsa-miR-331-3p | 6.770016108 | hsa-miR-193b | 5.168498029 |
| hsa-miR-760 | 5.616209106 | hsa-miR-223 | 6.752419058 | hsa-miR-378 | 5.144903436 |
| hsa-miR-422a | 5.603259428 | hsa-miR-26b | 6.711779741 | hsa-miR-371-5p | 5.063823407 |
| hsa-miR-617 | 5.43240052 | hsa-miR-513b | 6.677565711 | hsa-let-7i | 5.028477124 |
| hsa-miR-148a | 5.391857479 | hsa-miR-26a | 6.649552606 | hsa-miR-29b | 5.004304188 |
| hsa-miR-30d | 5.376875923 | hsa-miR-212 | 6.621618504 | hsa-miR-26a | 4.96042002 |
| hsa-miR-378 | 5.308218048 | hsa-miR-22 | 6.573627745 | hsa-miR-1225-3p | 4.94881417 |
| hsa-miR-520b | 5.280304047 | hsa-miR-181a | 6.572455818 | hsa-miR-142-5p | 4.947706291 |
| hsa-miR-601 | 5.257262386 | hsa-miR-130b | 6.544258701 | hsa-let-7b | 4.931210861 |
| hsa-miR-331-3p | 5.257105342 | hsa-let-7b | 6.543962999 | hsa-miR-425* | 4.914396302 |
| hsa-miR-584 | 5.238766221 | hsa-miR-10b* | 6.472776822 | hsa-miR-564 | 4.876704958 |
| hsa-miR-126 | 5.219934203 | hsa-miR-142-5p | 6.365604825 | hsa-miR-550 | 4.82301096 |
| hsa-miR-26b | 5.219460743 | hsa-miR-30c | 6.35244865 | hsa-miR-33b* | 4.788206654 |
| hsa-miR-29c | 5.175764189 | hsa-miR-378 | 6.263776304 | hsa-miR-223 | 4.783547571 |
| hsa-miR-1228 | 5.175437023 | hsa-let-7c | 6.086351726 | hsa-miR-766 | 4.751775938 |
| hsa-miR-940 | 5.111666937 | hsa-miR-99a | 6.006380228 | hsa-miR-181a | 4.722682085 |
| hsa-miR-26a | 5.099800717 | hsa-miR-1228 | 5.939063909 | hsa-miR-572 | 4.70934978 |
| hsa-miR-662 | 5.020164606 | hsa-miR-940 | 5.937079679 | hsa-let-7b* | 4.69363399 |
| hsa-let-7b | 5.01495564 | hsa-miR-760 | 5.862839397 | hsa-let-7f-1* | 4.670769583 |
| hsa-miR-30e | 5.007914867 | hsa-miR-617 | 5.815476754 | hsa-miR-29c | 4.662006654 |
| hsa-miR-510 | 4.949193217 | hsa-miR-874 | 5.784727524 | hsa-miR-149 | 4.645329838 |
| hsa-miR-501-5p | 4.915286663 | hsa-miR-765 | 5.765598129 | hsa-miR-18a | 4.634671205 |
| hsa-miR-921 | 4.907046924 | hsa-miR-770-5p | 5.763996184 | hsa-miR-1237 | 4.609903222 |
| hsa-miR-200a* | 4.884146778 | hsa-miR-101 | 5.750476743 | hsa-miR-1228* | 4.577117311 |
| hsa-miR-22 | 4.869563971 | hsa-miR-513c | 5.692940167 | hsa-miR-563 | 4.54937278 |
| hsa-miR-18a | 4.841173593 | hsa-miR-125b | 5.626886527 | hsa-miR-422a | 4.509312702 |
| hsa-miR-513c | 4.83953579 | hsa-miR-126 | 5.556358517 | hsa-miR-516a-5p | 4.48529953 |
| hsa-miR-371-5p | 4.809806593 | hsa-miR-590-5p | 5.467809391 | hsa-miR-101 | 4.457907916 |
| hsa-miR-181a | 4.758367499 | hsa-miR-557 | 5.465728679 | hsa-miR-602 | 4.44524196 |
| hsa-miR-130b | 4.748787431 | hsa-miR-151-5p | 5.38034866 | hsa-miR-331-3p | 4.405645201 |
| hsa-miR-564 | 4.741179779 | hsa-miR-18b | 5.375874483 | hsa-miR-22 | 4.401376804 |
| hsa-miR-30c | 4.728981534 | hsa-miR-371-5p | 5.314365585 | hsa-miR-148a | 4.389593736 |
| hsa-miR-766 | 4.705372342 | hsa-miR-140-3p | 5.295758112 | hsa-miR-557 | 4.362502654 |
| hsa-miR-23a | 4.698110228 | hsa-miR-1226* | 5.212707711 | hsa-miR-296-5p | 4.329180316 |
| hsa-miR-373* | 4.686197081 | hsa-miR-23a | 5.16763882 | hsa-miR-636 | 4.299265998 |
| hsa-miR-27a | 4.679064708 | hsa-miR-202 | 5.15725799 | hsa-miR-617 | 4.194979417 |
| hsa-miR-765 | 4.637965796 | hsa-miR-877 | 5.157244542 | hsa-miR-30c | 4.149970125 |
| hsa-miR-1234 | 4.600373553 | hsa-miR-21* | 5.121723332 | hsa-miR-206 | 4.149024925 |
| hsa-miR-887 | 4.589766812 | hsa-miR-24 | 5.107147313 | hsa-miR-9* | 4.134533422 |
| hsa-miR-520e | 4.561513708 | hsa-miR-296-5p | 5.095078438 | hsa-miR-197 | 4.106312041 |
| hsa-miR-519e* | 4.500699936 | hsa-miR-601 | 5.092900694 | hsa-miR-933 | 4.08470674 |
| hsa-miR-663 | 4.492953391 | hsa-miR-650 | 5.080754107 | hsa-miR-130b | 4.069884625 |
| hsa-miR-1225-3p | 4.420035814 | hsa-miR-564 | 5.080013041 | hsa-miR-198 | 4.038710033 |
| hsa-miR-382 | 4.395253455 | hsa-miR-27b | 5.079413528 | hsa-miR-659 | 4.016652394 |
| hsa-miR-610 | 4.385221938 | hsa-miR-140-5p | 5.079044473 | hsa-miR-513c | 3.956813128 |
| hsa-miR-27b | 4.37338293 | hsa-miR-186 | 5.065856422 | hsa-miR-610 | 3.918675807 |
| hsa-miR-623 | 4.368385625 | hsa-miR-572 | 5.061957915 | hsa-miR-186 | 3.870623679 |
| hsa-miR-1238 | 4.322051124 | hsa-miR-663 | 5.042604305 | hsa-miR-1229 | 3.860852436 |
| hsa-miR-296-5p | 4.29825199 | hsa-miR-17* | 4.956025638 | hsa-miR-92b | 3.852518778 |
| hsa-miR-149* | 4.276776703 | hsa-miR-1225-3p | 4.943538431 | hsa-miR-877* | 3.81649902 |
| hsa-miR-125b-1* | 4.249689968 | hsa-miR-892b | 4.934724408 | hsa-miR-874 | 3.812028413 |
| hsa-miR-602 | 4.237018733 | hsa-miR-30e* | 4.907299201 | hsa-miR-760 | 3.790677754 |
| hsa-miR-490-5p | 4.215767512 | hsa-miR-623 | 4.900928958 | hsa-miR-125b-2* | 3.785097431 |
| hsa-miR-151-5p | 4.194875168 | hsa-miR-370 | 4.893126254 | hsa-miR-539 | 3.763634847 |
| hsa-miR-24 | 4.182724256 | hsa-miR-1234 | 4.878882043 | hsa-miR-129-3p | 3.756350873 |
| hsa-miR-500* | 4.146768897 | hsa-miR-193b* | 4.843578096 | hsa-miR-623 | 3.721946287 |
| hsa-let-7c | 4.123145856 | hsa-miR-361-5p | 4.782202089 | hsa-miR-1227 | 3.650911101 |
| hsa-miR-338-5p | 4.121680755 | hsa-miR-23b | 4.772368271 | hsa-let-7c | 3.630279417 |
| hsa-miR-28-3p | 4.113379229 | hsa-miR-513a-3p | 4.74827227 | hsa-miR-583 | 3.587002186 |
| hsa-miR-99b* | 4.078532977 | hsa-miR-500 | 4.665000043 | hsa-miR-122 | 3.586044887 |
| hsa-miR-197 | 4.069643722 | hsa-miR-324-5p | 4.6516278 | hsa-miR-601 | 3.580898618 |
| hsa-miR-191* | 4.015846692 | hsa-miR-610 | 4.635386869 | hsa-miR-129* | 3.563141116 |
| hsa-miR-202 | 4.014477491 | hsa-miR-625 | 4.625346831 | hsa-miR-634 | 3.533487962 |
| hsa-miR-1237 | 4.010978978 | hsa-miR-181b | 4.58191121 | hsa-miR-1226* | 3.462401348 |
| hsa-miR-9* | 4.009861388 | hsa-miR-766 | 4.579670623 | hsa-miR-518c* | 3.461279788 |
| hsa-miR-23b | 3.991377756 | hsa-miR-128 | 4.574272938 | hsa-miR-181b | 3.404610639 |
| hsa-miR-557 | 3.969340529 | hsa-miR-27a | 4.566442159 | hsa-miR-483-3p | 3.397500802 |
| hsa-miR-590-5p | 3.95246185 | hsa-miR-301a | 4.558064638 | hsa-miR-484 | 3.377334938 |
| hsa-miR-212 | 3.940720119 | hsa-miR-125b-2* | 4.532076492 | hsa-miR-625* | 3.372467106 |
| hsa-miR-425* | 3.911558459 | hsa-miR-98 | 4.513589703 | hsa-miR-665 | 3.329923918 |
| hsa-miR-1229 | 3.823694285 | hsa-miR-520b | 4.500946907 | hsa-miR-370 | 3.325815333 |
| hsa-miR-632 | 3.809796474 | hsa-miR-374a | 4.499229465 | hsa-miR-149* | 3.273126176 |
| hsa-miR-33b* | 3.788139188 | hsa-miR-636 | 4.496474114 | hsa-miR-338-5p | 3.265840524 |
| hsa-miR-877 | 3.752434476 | hsa-miR-192 | 4.451520741 | hsa-miR-18b* | 3.214759209 |
| hsa-miR-150 | 3.741314344 | hsa-miR-662 | 4.4453126 | hsa-miR-21* | 3.171150787 |
| hsa-miR-125b | 3.732902547 | hsa-miR-7 | 4.402907042 | hsa-miR-125b | 3.165080915 |
| hsa-miR-125b-2* | 3.73254417 | hsa-miR-148b* | 4.395705339 | hsa-miR-18b | 3.103833057 |
| hsa-miR-10b* | 3.715784981 | hsa-miR-598 | 4.377799065 | hsa-miR-133a | 3.082334282 |
| hsa-let-7b* | 3.714185216 | hsa-miR-542-5p | 4.358469713 | hsa-miR-7 | 3.078736855 |
| hsa-miR-361-5p | 3.710672378 | hsa-miR-500* | 4.355759937 | hsa-miR-151-5p | 3.056077188 |
| hsa-miR-542-5p | 3.703376216 | hsa-miR-1238 | 4.337698058 | hsa-miR-212 | 3.053073777 |
| hsa-miR-574-5p | 3.70105614 | hsa-miR-431 | 4.248707484 | hsa-miR-202 | 2.984254616 |
| hsa-miR-142-5p | 3.665924528 | hsa-miR-181a* | 4.246655581 | hsa-miR-671-3p | 2.969447411 |
| hsa-miR-339-3p | 3.653216799 | hsa-miR-659 | 4.212963102 | hsa-miR-23a | 2.942798199 |
| hsa-miR-634 | 3.652823188 | hsa-miR-197 | 4.206936029 | hsa-miR-513a-3p | 2.937727351 |
| hsa-miR-487b | 3.62531367 | hsa-miR-28-5p | 4.158664901 | hsa-miR-140-3p | 2.926484906 |
| hsa-miR-629* | 3.600475939 | hsa-miR-193a-5p | 4.140351425 | hsa-miR-548c-3p | 2.925390325 |
| hsa-miR-140-3p | 3.591469393 | hsa-miR-148b | 4.130750884 | hsa-miR-584 | 2.915872948 |
| hsa-miR-550 | 3.586909868 | hsa-miR-200a* | 4.069761844 | hsa-miR-99a | 2.833895874 |
| hsa-miR-149 | 3.564751607 | hsa-miR-1237 | 4.069397581 | hsa-miR-767-3p | 2.819327194 |
| hsa-let-7f-1* | 3.554183301 | hsa-miR-505 | 4.045499412 | hsa-miR-301a | 2.814808347 |
| hsa-miR-628-3p | 3.519279603 | hsa-miR-138 | 4.042358593 | hsa-miR-297 | 2.813555865 |
| hsa-miR-513a-3p | 3.492175367 | hsa-miR-1228* | 4.024960908 | hsa-miR-195* | 2.813108615 |
| hsa-miR-622 | 3.481557786 | hsa-miR-205 | 4.016153182 | hsa-miR-382 | 2.809395546 |
| hsa-miR-330-3p | 3.468957781 | hsa-miR-33b* | 4.015930817 | hsa-miR-409-3p | 2.806487554 |
| hsa-miR-936 | 3.444720764 | hsa-miR-185 | 3.985812236 | hsa-miR-30c-2* | 2.790694001 |
| hsa-miR-498 | 3.414199233 | hsa-miR-492 | 3.974978342 | hsa-miR-486-5p | 2.761705599 |
| hsa-miR-502-3p | 3.404142662 | hsa-miR-99b* | 3.972711652 | hsa-miR-127-3p | 2.750868116 |
| hsa-miR-486-5p | 3.392152454 | hsa-miR-191* | 3.953737092 | hsa-miR-30b* | 2.739072113 |
| hsa-miR-636 | 3.382920673 | hsa-miR-486-5p | 3.910293753 | hsa-miR-181d | 2.727492023 |
| hsa-miR-99a | 3.368334434 | hsa-miR-30c-1* | 3.909775267 | hsa-miR-29b-1* | 2.709176942 |
| hsa-miR-129-3p | 3.366239663 | hsa-miR-7-1* | 3.909559841 | hsa-miR-23b | 2.708515842 |
| hsa-miR-151-3p | 3.361200161 | hsa-miR-583 | 3.907979068 | hsa-miR-98 | 2.683891082 |
| hsa-miR-101 | 3.357129531 | hsa-miR-649 | 3.903779228 | hsa-miR-432 | 2.675020562 |
| hsa-miR-627 | 3.261002172 | hsa-miR-622 | 3.879331177 | hsa-miR-936 | 2.654065716 |
| hsa-miR-7 | 3.251450207 | hsa-miR-632 | 3.865988345 | hsa-miR-27b | 2.633277599 |
| hsa-miR-770-5p | 3.242939961 | hsa-miR-520e | 3.852554312 | hsa-miR-140-5p | 2.623306894 |
| hsa-miR-17* | 3.201050673 | hsa-miR-936 | 3.850760279 | hsa-miR-625 | 2.604861086 |
| hsa-miR-769-3p | 3.191907684 | hsa-miR-374b | 3.834739831 | hsa-miR-520b | 2.60299544 |
| hsa-miR-18b | 3.187557198 | hsa-miR-339-3p | 3.826894857 | hsa-miR-221 | 2.573997727 |
| hsa-miR-563 | 3.180016667 | hsa-miR-887 | 3.816787994 | hsa-miR-34a | 2.57115984 |
| hsa-miR-933 | 3.164894021 | hsa-miR-151-3p | 3.776889575 | hsa-miR-126 | 2.54780237 |
| hsa-miR-181b | 3.128383179 | hsa-miR-215 | 3.7668659 | hsa-miR-887 | 2.538421141 |
| hsa-miR-342-3p | 3.100243946 | hsa-miR-200b* | 3.75879304 | hsa-miR-590-5p | 2.515676295 |
| hsa-miR-877* | 3.091958576 | hsa-miR-629* | 3.757109196 | hsa-miR-30c-1* | 2.489501663 |
| hsa-miR-625 | 3.069691354 | hsa-miR-769-3p | 3.719968741 | hsa-miR-361-5p | 2.469177694 |
| hsa-miR-186 | 3.044156036 | hsa-miR-342-3p | 3.717847253 | hsa-miR-505 | 2.430074258 |
| hsa-miR-144 | 3.040107356 | hsa-miR-886-3p | 3.710576005 | hsa-miR-885-3p | 2.407795438 |
| hsa-miR-129* | 3.032807109 | hsa-miR-184 | 3.679595209 | hsa-miR-24 | 2.401860546 |
| hsa-miR-140-5p | 3.013348982 | hsa-miR-584 | 3.662456261 | hsa-miR-17* | 2.362791938 |
| hsa-miR-641 | 2.97355419 | hsa-miR-425* | 3.658056477 | hsa-miR-10b* | 2.355491092 |
| hsa-miR-492 | 2.949958409 | hsa-miR-502-3p | 3.655727899 | hsa-miR-133b | 2.31181703 |
| hsa-miR-505 | 2.920233267 | hsa-miR-150 | 3.650200346 | hsa-miR-770-5p | 2.307313853 |
| hsa-miR-345 | 2.904356114 | hsa-miR-518c* | 3.630859882 | hsa-miR-769-5p | 2.305009397 |
| hsa-miR-650 | 2.903993354 | hsa-miR-501-5p | 3.597497937 | hsa-miR-622 | 2.297120759 |
| hsa-miR-192 | 2.885758875 | hsa-miR-769-5p | 3.590320149 | hsa-miR-345 | 2.234330521 |
| hsa-miR-148b | 2.882339303 | hsa-miR-361-3p | 3.569711616 | hsa-miR-629* | 2.215076461 |
| hsa-miR-518a-3p | 2.863203375 | hsa-miR-378* | 3.565293174 | hsa-miR-328 | 2.209875748 |
| hsa-miR-128 | 2.844134841 | hsa-miR-490-5p | 3.55925542 | hsa-miR-892b | 2.178477126 |
| hsa-miR-98 | 2.838614502 | hsa-miR-602 | 3.530042366 | hsa-miR-192 | 2.172250302 |
| hsa-miR-10b | 2.835178841 | hsa-miR-194 | 3.522677197 | hsa-miR-708 | 2.144731718 |
| hsa-miR-518c* | 2.833165027 | hsa-miR-338-5p | 3.519545007 | hsa-miR-324-5p | 2.139744501 |
| hsa-miR-183* | 2.784398928 | hsa-miR-20a* | 3.500830358 | hsa-miR-299-5p | 2.135866454 |
| hsa-miR-145 | 2.747879723 | hsa-miR-519e* | 3.472733414 | hsa-miR-148b | 2.134966501 |
| hsa-miR-509-5p | 2.741540487 | hsa-miR-550 | 3.463886104 | hsa-miR-383 | 2.134331336 |
| hsa-miR-136 | 2.738323988 | hsa-miR-921 | 3.44657714 | hsa-miR-30e* | 2.132394936 |
| hsa-miR-886-3p | 2.737740444 | hsa-miR-509-5p | 3.444597035 | hsa-miR-361-3p | 2.112078155 |
| hsa-miR-92b | 2.702440792 | hsa-miR-28-3p | 3.420532275 | hsa-miR-650 | 2.097211289 |
| hsa-miR-453 | 2.701097471 | hsa-miR-498 | 3.414509177 | hsa-miR-454 | 2.09440482 |
| hsa-miR-1227 | 2.695801265 | hsa-miR-627 | 3.379458595 | hsa-miR-498 | 2.089483643 |
| hsa-miR-370 | 2.690108752 | hsa-miR-9 | 3.359667055 | hsa-miR-453 | 2.048195962 |
| hsa-miR-194 | 2.676228478 | hsa-let-7b* | 3.321519557 | hsa-miR-662 | 2.029436341 |
| hsa-miR-708 | 2.647776771 | hsa-miR-631 | 3.314322031 | hsa-miR-520e | 2.02867518 |
| hsa-miR-769-5p | 2.629565045 | hsa-miR-634 | 3.31102977 | hsa-miR-629 | 2.018108957 |
| hsa-miR-299-5p | 2.624188998 | hsa-miR-138-2* | 3.291080548 | hsa-miR-520d-3p | 1.997531103 |
| hsa-miR-625* | 2.6151122 | hsa-miR-29b-1* | 3.266301925 | hsa-miR-374a | 1.98269578 |
| hsa-miR-598 | 2.593220113 | hsa-miR-149* | 3.232946275 | hsa-miR-605 | 1.96748244 |
| hsa-miR-23a* | 2.571037154 | hsa-miR-149 | 3.222125442 | hsa-miR-421 | 1.942579496 |
| hsa-miR-484 | 2.567993537 | hsa-miR-30a | 3.185130918 | hsa-miR-215 | 1.940383166 |
| hsa-miR-628-5p | 2.441682277 | hsa-miR-330-3p | 3.172510928 | hsa-miR-501-5p | 1.932872532 |
| hsa-miR-483-3p | 2.438996396 | hsa-miR-505* | 3.084213208 | hsa-miR-542-5p | 1.887320879 |
| hsa-miR-148b* | 2.424082554 | hsa-miR-933 | 3.074838368 | hsa-miR-339-3p | 1.884483288 |
| hsa-miR-21* | 2.42354621 | hsa-miR-100 | 3.071713653 | hsa-miR-1233 | 1.869725761 |
| hsa-miR-200b* | 2.385570277 | hsa-let-7f-1* | 3.059744534 | hsa-miR-28-5p | 1.850556444 |
| hsa-miR-18b* | 2.353914859 | hsa-miR-1229 | 3.058932813 | hsa-miR-128 | 1.839654728 |
| hsa-miR-215 | 2.351464607 | hsa-miR-454 | 3.053129778 | hsa-miR-769-3p | 1.839327348 |
| hsa-miR-301a | 2.317178525 | hsa-miR-345 | 3.018009716 | hsa-miR-1236 | 1.836816008 |
| hsa-miR-631 | 2.311505316 | hsa-miR-421 | 2.97087637 | hsa-miR-136 | 1.818620642 |
| hsa-miR-30e* | 2.304007365 | hsa-miR-708 | 2.926768589 | hsa-miR-545 | 1.807743529 |
| hsa-miR-221 | 2.30114588 | hsa-miR-16-2* | 2.920808393 | hsa-miR-598 | 1.791300367 |
| hsa-miR-374b | 2.289326455 | hsa-miR-129-3p | 2.901746511 | hsa-miR-335* | 1.789608046 |
| hsa-miR-9 | 2.274036981 | hsa-miR-186* | 2.879848888 | hsa-miR-187* | 1.754937449 |
| hsa-miR-583 | 2.251844742 | hsa-miR-155* | 2.867607575 | hsa-miR-423-3p | 1.738254718 |
| hsa-miR-133a | 2.228087349 | hsa-miR-625* | 2.838801061 | hsa-miR-9 | 1.734248601 |
| hsa-miR-30a | 2.204009736 | hsa-miR-10b | 2.816975591 | hsa-miR-16-2* | 1.694944366 |
| hsa-miR-423-3p | 2.202401165 | hsa-miR-877* | 2.790696359 | hsa-miR-645 | 1.682139259 |
| hsa-miR-374a | 2.168574634 | hsa-miR-195 | 2.772171934 | hsa-miR-632 | 1.676220243 |
| hsa-miR-185 | 2.167316936 | hsa-miR-18b* | 2.714842354 | hsa-miR-548d-5p | 1.670604519 |
| hsa-miR-200c | 2.137726855 | hsa-miR-942 | 2.711407247 | hsa-miR-500 | 1.653523674 |
| hsa-miR-181d | 2.12784121 | hsa-miR-146b-5p | 2.704806135 | hsa-miR-607 | 1.642735702 |
| hsa-miR-491-5p | 2.116947266 | hsa-miR-196a | 2.703726894 | hsa-miR-942 | 1.642290162 |
| hsa-miR-324-5p | 2.108195783 | hsa-miR-92b | 2.699383473 | hsa-miR-194 | 1.618161206 |
| hsa-miR-28-5p | 2.105815648 | hsa-miR-221 | 2.697572023 | hsa-miR-342-3p | 1.617587161 |
| hsa-miR-181a* | 2.089995736 | hsa-miR-484 | 2.694997461 | hsa-miR-631 | 1.584168314 |
| hsa-miR-7-1* | 2.087419127 | hsa-miR-563 | 2.682452629 | hsa-miR-181c* | 1.579902937 |
| hsa-miR-516a-5p | 2.036024216 | hsa-miR-34a | 2.682422817 | hsa-miR-186* | 1.569521436 |
| hsa-miR-525-5p | 1.978840344 | hsa-miR-30c-2* | 2.652225891 | hsa-miR-99b* | 1.555911743 |
| hsa-miR-29b-1* | 1.970764926 | hsa-miR-23a* | 2.651323857 | hsa-miR-516b | 1.548846133 |
| hsa-miR-302c* | 1.963022039 | hsa-miR-145 | 2.648610574 | hsa-miR-497* | 1.542143487 |
| hsa-miR-302a | 1.962496749 | hsa-miR-33a | 2.63471922 | hsa-miR-485-3p | -1.515651322 |
| hsa-miR-520d-3p | 1.954974051 | hsa-miR-10a* | 2.631694951 | hsa-miR-517a | -1.536307157 |
| hsa-miR-378* | 1.945761312 | hsa-miR-129* | 2.621861696 | hsa-miR-34a* | -1.546413162 |
| hsa-miR-518e* | 1.916307852 | hsa-miR-30b* | 2.603914037 | hsa-miR-182* | -1.564388557 |
| hsa-miR-526b | 1.914260932 | hsa-miR-29c* | 2.591646704 | hsa-miR-24-2* | -1.579945545 |
| hsa-miR-942 | 1.895694168 | hsa-miR-92a-1* | 2.564236199 | hsa-miR-148a* | -1.587253996 |
| hsa-miR-184 | 1.880646746 | hsa-miR-139-3p | 2.556185609 | hsa-miR-624 | -1.589148366 |
| hsa-miR-141* | 1.876753105 | hsa-miR-595 | 2.536078247 | hsa-miR-551a | -1.594123997 |
| hsa-miR-518f | 1.844078305 | hsa-miR-132 | 2.527670763 | hsa-miR-944 | -1.605753772 |
| hsa-miR-885-5p | 1.834005473 | hsa-miR-219-5p | 2.523344775 | hsa-miR-626 | -1.614193334 |
| hsa-miR-409-3p | 1.817414207 | hsa-miR-181d | 2.523301118 | hsa-miR-26a-1* | -1.615319287 |
| hsa-miR-138-2* | 1.795072625 | hsa-miR-32* | 2.495166314 | hsa-miR-220c | -1.626679414 |
| hsa-miR-361-3p | 1.792394032 | hsa-miR-665 | 2.488736148 | hsa-miR-431* | -1.627714461 |
| hsa-miR-122 | 1.785859306 | hsa-miR-152 | 2.455513831 | hsa-miR-200c | -1.63446297 |
| hsa-miR-217 | 1.780641933 | hsa-miR-432 | 2.449983893 | hsa-miR-548a-5p | -1.658236028 |
| hsa-miR-100 | 1.778240166 | hsa-miR-200c | 2.383216673 | hsa-miR-640 | -1.676002152 |
| hsa-miR-671-3p | 1.745206106 | hsa-miR-130a | 2.381833418 | hsa-miR-214* | -1.678826018 |
| hsa-miR-33a* | 1.74172514 | hsa-miR-582-5p | 2.371084561 | hsa-miR-34c-5p | -1.689696199 |
| hsa-miR-506 | 1.711160715 | hsa-miR-181c* | 2.3669979 | hsa-miR-591 | -1.695559585 |
| hsa-miR-421 | 1.687902886 | hsa-miR-516b | 2.340347123 | hsa-miR-585 | -1.70805062 |
| hsa-miR-10a | 1.683043101 | hsa-miR-628-3p | 2.336028974 | hsa-miR-367 | -1.716254788 |
| hsa-miR-508-3p | 1.670278828 | hsa-miR-298 | 2.325411906 | hsa-miR-571 | -1.718665514 |
| hsa-miR-99b | 1.608868217 | hsa-miR-423-3p | 2.310336487 | hsa-miR-299-3p | -1.726116815 |
| hsa-miR-335* | 1.596963943 | hsa-miR-133b | 2.290388019 | hsa-miR-362-3p | -1.735254953 |
| hsa-miR-376c | 1.592747462 | hsa-miR-196b | 2.288337279 | hsa-miR-615-3p | -1.751411725 |
| hsa-let-7d* | 1.591243055 | hsa-miR-526b | 2.287080998 | hsa-miR-124* | -1.753530926 |
| hsa-miR-30c-2* | 1.581628034 | hsa-miR-299-3p | 2.282336976 | hsa-miR-154 | -1.773272275 |
| hsa-miR-888* | 1.579319801 | hsa-miR-133a | 2.269734835 | hsa-miR-651 | -1.77557747 |
| hsa-miR-432 | 1.553752571 | hsa-miR-409-3p | 2.265882002 | hsa-miR-580 | -1.788759068 |
| hsa-miR-659 | 1.552983694 | hsa-miR-1227 | 2.262280136 | hsa-miR-455-3p | -1.793786083 |
| hsa-miR-16-2* | 1.539584418 | hsa-miR-509-3-5p | 2.261130636 | hsa-miR-409-5p | -1.839252826 |
| hsa-miR-328 | 1.526775638 | hsa-miR-331-5p | 2.254557134 | hsa-miR-886-5p | -1.84834737 |
| hsa-miR-514 | 1.519030643 | hsa-miR-525-5p | 2.2496583 | hsa-miR-363 | -1.852962381 |
| hsa-miR-371-3p | 1.502845032 | hsa-let-7e | 2.238287209 | hsa-miR-106a* | -1.872025589 |
| hsa-miR-603 | -1.151964997 | hsa-miR-222 | 2.216546594 | hsa-miR-541 | -1.88469816 |
| hsa-miR-431 | -1.176813908 | hsa-miR-136 | 2.195313388 | hsa-miR-490-3p | -1.920530266 |
| hsa-miR-455-5p | -1.184691891 | hsa-miR-124 | 2.186459419 | hsa-miR-342-5p | -1.936274818 |
| hsa-miR-493 | -1.202906798 | hsa-miR-301b | 2.176338502 | hsa-miR-216b | -1.954515983 |
| hsa-miR-767-5p | -1.207670916 | hsa-miR-744 | 2.153297392 | hsa-miR-549 | -1.972029364 |
| hsa-miR-517c | -1.217166767 | hsa-miR-22* | 2.148164482 | hsa-miR-105 | -1.979266107 |
| hsa-miR-148a* | -1.223636569 | hsa-miR-629 | 2.142317027 | hsa-miR-24-1* | -1.982638234 |
| hsa-miR-147 | -1.229976978 | hsa-miR-373* | 2.049904439 | hsa-miR-510 | -1.991873273 |
| hsa-miR-485-5p | -1.284473932 | hsa-miR-605 | 2.045572323 | hsa-miR-323-3p | -1.997659909 |
| hsa-miR-124* | -1.308659361 | hsa-miR-641 | 2.045338647 | hsa-miR-506 | -2.018160949 |
| hsa-miR-146a* | -1.320820818 | hsa-miR-483-3p | 2.024800394 | hsa-miR-99a* | -2.018541731 |
| hsa-miR-646 | -1.32758434 | hsa-miR-604 | 1.994194479 | hsa-miR-31* | -2.054328401 |
| hsa-miR-203 | -1.350188666 | hsa-miR-424 | 1.936622372 | hsa-miR-384 | -2.062467615 |
| hsa-miR-586 | -1.359661849 | hsa-miR-518a-3p | 1.926076082 | hsa-miR-23b* | -2.077179891 |
| hsa-miR-582-3p | -1.368023855 | hsa-miR-19b-1* | 1.921440115 | hsa-miR-188-3p | -2.082343973 |
| hsa-miR-182 | -1.37192453 | hsa-miR-489 | 1.916754822 | hsa-miR-200b | -2.082746246 |
| hsa-miR-590-3p | -1.378254409 | hsa-miR-141* | 1.897151154 | hsa-miR-217 | -2.082815599 |
| hsa-miR-24-1* | -1.391117109 | hsa-miR-671-3p | 1.886949873 | hsa-miR-410 | -2.085450048 |
| hsa-miR-499-3p | -1.40081839 | hsa-miR-508-5p | 1.880171896 | hsa-miR-621 | -2.130148386 |
| hsa-miR-372 | -1.41709541 | hsa-miR-516a-3p | 1.866283541 | hsa-miR-18a* | -2.151733413 |
| hsa-miR-302d | -1.429738757 | hsa-miR-648 | 1.856550976 | hsa-miR-106b* | -2.182975511 |
| hsa-miR-553 | -1.434797898 | hsa-miR-890 | 1.83955171 | hsa-miR-154* | -2.193801968 |
| hsa-miR-647 | -1.44495746 | hsa-miR-206 | 1.778201511 | hsa-miR-520g | -2.199355549 |
| hsa-miR-548b-5p | -1.475775253 | hsa-miR-122 | 1.720436776 | hsa-miR-30d* | -2.200601751 |
| hsa-miR-612 | -1.506542749 | hsa-miR-25* | 1.717738922 | hsa-miR-509-3-5p | -2.203574562 |
| hsa-miR-32 | -1.506653198 | hsa-miR-376c | 1.671998125 | hsa-miR-220b | -2.216904001 |
| hsa-miR-384 | -1.514957518 | hsa-miR-767-3p | 1.64778683 | hsa-miR-379* | -2.218636093 |
| hsa-miR-19b-2* | -1.517163617 | hsa-miR-934 | 1.626654728 | hsa-miR-920 | -2.219395242 |
| hsa-miR-559 | -1.532759111 | hsa-miR-217 | 1.614546897 | hsa-miR-199a-5p | -2.220220771 |
| hsa-miR-138-1* | -1.537477267 | hsa-miR-512-3p | 1.595436276 | hsa-miR-555 | -2.245701918 |
| hsa-miR-92a-2* | -1.540549693 | hsa-miR-199a-5p | 1.541986761 | hsa-miR-125a-5p | -2.246691891 |
| hsa-miR-377* | -1.553724797 | hsa-miR-141 | 1.537429339 | hsa-miR-143* | -2.26093733 |
| hsa-miR-1226 | -1.56961724 | hsa-miR-520c-3p | 1.536105437 | hsa-miR-208b | -2.262578643 |
| hsa-miR-618 | -1.590122275 | hsa-miR-520d-3p | 1.532012222 | hsa-miR-873 | -2.283988424 |
| hsa-miR-541* | -1.603317853 | hsa-miR-299-5p | 1.530065731 | hsa-miR-127-5p | -2.296107014 |
| hsa-miR-542-3p | -1.606974329 | hsa-miR-135b* | 1.52326988 | hsa-miR-141 | -2.349732775 |
| hsa-miR-517a | -1.638971642 | hsa-miR-144 | 1.512874744 | hsa-miR-376a | -2.376764685 |
| hsa-miR-449b | -1.640158974 | hsa-miR-185* | -1.501909683 | hsa-miR-597 | -2.378414364 |
| hsa-miR-448 | -1.655587192 | hsa-miR-548d-3p | -1.508169377 | hsa-miR-875-5p | -2.39687991 |
| hsa-miR-589 | -1.663966354 | hsa-miR-194* | -1.508641696 | hsa-miR-452* | -2.402823061 |
| hsa-miR-624 | -1.664404865 | hsa-miR-23b* | -1.535119939 | hsa-miR-203 | -2.404543511 |
| hsa-miR-214* | -1.669394373 | hsa-miR-302a* | -1.538177598 | hsa-miR-300 | -2.411886053 |
| hsa-miR-298 | -1.670607499 | hsa-miR-219-2-3p | -1.54119303 | hsa-miR-518c | -2.41791848 |
| hsa-miR-635 | -1.680930705 | hsa-miR-183 | -1.567624764 | hsa-miR-603 | -2.41868702 |
| hsa-miR-33a | -1.701510558 | hsa-miR-589 | -1.567752322 | hsa-miR-888 | -2.442862713 |
| hsa-miR-532-5p | -1.701523563 | hsa-miR-363* | -1.584689662 | hsa-miR-136* | -2.44792718 |
| hsa-miR-96* | -1.71822853 | hsa-miR-892a | -1.608921475 | hsa-miR-485-5p | -2.490419604 |
| hsa-miR-580 | -1.736511929 | hsa-miR-20b* | -1.61932175 | hsa-miR-648 | -2.492645656 |
| hsa-miR-591 | -1.737548269 | hsa-miR-369-3p | -1.627814722 | hsa-miR-452 | -2.49814774 |
| hsa-miR-488 | -1.749578419 | hsa-miR-599 | -1.630235384 | hsa-miR-507 | -2.515447764 |
| hsa-miR-545 | -1.774308395 | hsa-miR-569 | -1.634705935 | hsa-miR-542-3p | -2.534725534 |
| hsa-miR-340 | -1.854128466 | hsa-miR-556-5p | -1.637981436 | hsa-miR-200c* | -2.541202578 |
| hsa-miR-518e | -1.874847752 | hsa-miR-377 | -1.638391028 | hsa-miR-182 | -2.556374137 |
| hsa-miR-376b | -1.87966694 | hsa-miR-380 | -1.641653952 | hsa-miR-497 | -2.599351172 |
| hsa-miR-548a-5p | -1.938126842 | hsa-miR-589* | -1.645444103 | hsa-miR-152 | -2.613784663 |
| hsa-miR-935 | -1.990512763 | hsa-miR-517a | -1.646297907 | hsa-miR-642 | -2.629529454 |
| hsa-miR-374b* | -2.011606572 | hsa-miR-504 | -1.647910124 | hsa-miR-379 | -2.63908017 |
| hsa-miR-577 | -2.013644581 | hsa-miR-615-5p | -1.650459189 | hsa-miR-137 | -2.678185094 |
| hsa-miR-429 | -2.065557351 | hsa-miR-16-1* | -1.671819806 | hsa-miR-518f | -2.729311687 |
| hsa-miR-644 | -2.081881182 | hsa-miR-541 | -1.672213277 | hsa-let-7e | -2.732044308 |
| hsa-miR-302b* | -2.084233587 | hsa-miR-143* | -1.680118106 | hsa-miR-214 | -2.732529969 |
| hsa-miR-549 | -2.085259269 | hsa-miR-519b-3p | -1.693241615 | hsa-miR-523 | -2.737294181 |
| hsa-miR-330-5p | -2.08809775 | hsa-miR-567 | -1.695825387 | hsa-miR-767-5p | -2.758139333 |
| hsa-miR-607 | -2.089279399 | hsa-miR-124* | -1.706854974 | hsa-miR-146a* | -2.763429214 |
| hsa-miR-606 | -2.120695645 | hsa-miR-31 | -1.707301944 | hsa-miR-302c* | -2.76728418 |
| hsa-miR-517* | -2.142958587 | hsa-miR-1224-3p | -1.708627776 | hsa-miR-589 | -2.768535737 |
| hsa-miR-195* | -2.195070042 | hsa-miR-548a-3p | -1.710320516 | hsa-miR-578 | -2.789925429 |
| hsa-miR-32* | -2.243078326 | hsa-miR-372 | -1.716640241 | hsa-miR-424* | -2.801443367 |
| hsa-miR-651 | -2.301147402 | hsa-miR-607 | -1.760135376 | hsa-miR-147 | -2.80683949 |
| hsa-miR-20b* | -2.303295905 | hsa-miR-873 | -1.763080676 | hsa-miR-554 | -2.816045769 |
| hsa-miR-27b* | -2.320094899 | hsa-miR-581 | -1.779020367 | hsa-miR-218-1* | -2.819067165 |
| hsa-miR-541 | -2.34072334 | hsa-miR-432* | -1.791134273 | hsa-miR-222* | -2.864548172 |
| hsa-miR-224 | -2.343813998 | hsa-miR-571 | -1.79223749 | hsa-miR-139-5p | -2.872804609 |
| hsa-miR-34a* | -2.356354024 | hsa-miR-106a* | -1.797583367 | hsa-miR-576-3p | -2.894879723 |
| hsa-miR-515-5p | -2.361209507 | hsa-miR-562 | -1.799537929 | hsa-miR-147b | -2.90291847 |
| hsa-miR-152 | -2.37139841 | hsa-miR-222* | -1.826666045 | hsa-miR-200a | -2.928817976 |
| hsa-miR-554 | -2.373443817 | hsa-miR-586 | -1.833441046 | hsa-miR-146b-3p | -2.937259763 |
| hsa-miR-222* | -2.389690875 | hsa-miR-34a* | -1.840146542 | hsa-miR-620 | -2.941088037 |
| hsa-miR-380* | -2.451876412 | hsa-miR-92b* | -1.841047225 | hsa-miR-660 | -2.957565414 |
| hsa-miR-558 | -2.457035706 | hsa-miR-182 | -1.846940725 | hsa-miR-335 | -2.963933882 |
| hsa-miR-548c-3p | -2.474473257 | hsa-miR-323-3p | -1.847558436 | hsa-miR-19b-2* | -2.964930894 |
| hsa-miR-183 | -2.474494402 | hsa-miR-591 | -1.870549217 | hsa-miR-661 | -2.967210517 |
| hsa-miR-331-5p | -2.478593139 | hsa-miR-29b-2* | -1.884970336 | hsa-miR-514 | -2.989126352 |
| hsa-miR-520a-5p | -2.488641484 | hsa-miR-496 | -1.88548351 | hsa-miR-891a | -3.072181844 |
| hsa-miR-27a* | -2.504982524 | hsa-miR-374a* | -1.885729558 | hsa-miR-502-3p | -3.086937698 |
| hsa-miR-218-2* | -2.530172942 | hsa-miR-146b-3p | -1.894668551 | hsa-miR-644 | -3.096639632 |
| hsa-miR-615-5p | -2.564372796 | hsa-miR-549 | -1.897683065 | hsa-miR-744 | -3.101759976 |
| hsa-miR-127-5p | -2.593892614 | hsa-miR-553 | -1.913672768 | hsa-miR-208a | -3.117465412 |
| hsa-miR-615-3p | -2.60139251 | hsa-miR-379* | -1.923879215 | hsa-miR-377* | -3.141392587 |
| hsa-miR-101* | -2.636782176 | hsa-miR-558 | -1.925090493 | hsa-miR-340 | -3.171944741 |
| hsa-miR-496 | -2.678843464 | hsa-miR-647 | -1.954494413 | hsa-miR-616 | -3.180529738 |
| hsa-miR-501-3p | -2.686807143 | hsa-miR-33b | -1.976523047 | hsa-miR-600 | -3.185268775 |
| hsa-miR-569 | -2.695162764 | hsa-let-7i* | -1.993762881 | hsa-miR-337-5p | -3.232490354 |
| hsa-miR-33b | -2.713524155 | hsa-miR-376b | -2.002085306 | hsa-miR-649 | -3.255967783 |
| hsa-miR-220c | -2.738983886 | hsa-miR-188-3p | -2.016597718 | hsa-miR-503 | -3.299320996 |
| hsa-miR-194* | -2.749300942 | hsa-miR-889 | -2.019721787 | hsa-miR-302d* | -3.307547884 |
| hsa-miR-25* | -2.757614093 | hsa-miR-220b | -2.033306906 | hsa-miR-30a* | -3.338999081 |
| hsa-miR-614 | -2.762110693 | hsa-miR-223* | -2.039196617 | hsa-miR-643 | -3.355202212 |
| hsa-miR-105* | -2.773345812 | hsa-miR-153 | -2.042417292 | hsa-miR-512-5p | -3.362075848 |
| hsa-miR-223* | -2.778544314 | hsa-miR-517c | -2.04504162 | hsa-miR-10a* | -3.365292234 |
| hsa-miR-302a* | -2.783031541 | hsa-miR-618 | -2.056178828 | hsa-miR-216a | -3.378217442 |
| hsa-miR-31 | -2.784922747 | hsa-miR-31* | -2.07896961 | hsa-miR-675 | -3.406915518 |
| hsa-miR-885-3p | -2.799697206 | hsa-miR-651 | -2.090657265 | hsa-miR-1231 | -3.422924893 |
| hsa-miR-181c | -2.824356937 | hsa-miR-597 | -2.099895513 | hsa-miR-1 | -3.423706206 |
| hsa-miR-29b-2* | -2.828078098 | hsa-miR-518e | -2.104884053 | hsa-miR-619 | -3.439211577 |
| hsa-miR-191 | -2.832258281 | hsa-miR-644 | -2.114852486 | hsa-miR-532-3p | -3.44562333 |
| hsa-miR-220b | -2.86061961 | hsa-miR-384 | -2.12802963 | hsa-miR-429 | -3.465126913 |
| hsa-miR-675 | -2.863320346 | hsa-miR-191 | -2.154243863 | hsa-miR-589* | -3.491870014 |
| hsa-miR-486-3p | -2.870960903 | hsa-miR-532-3p | -2.156121411 | hsa-miR-608 | -3.52765732 |
| hsa-miR-802 | -2.891844288 | hsa-miR-888 | -2.161643345 | hsa-miR-218 | -3.53566711 |
| hsa-miR-581 | -2.898022094 | hsa-miR-485-5p | -2.161799121 | hsa-miR-487a | -3.564201763 |
| hsa-miR-367* | -2.898992075 | hsa-miR-24-1* | -2.173426701 | hsa-miR-219-1-3p | -3.583578941 |
| hsa-let-7g* | -2.912675193 | hsa-miR-935 | -2.178394507 | hsa-miR-941 | -3.596487996 |
| hsa-miR-137 | -2.914153236 | hsa-miR-196a* | -2.186475141 | hsa-miR-323-5p | -3.684123816 |
| hsa-miR-708* | -2.916505428 | hsa-miR-579 | -2.251172911 | hsa-miR-363* | -3.697088098 |
| hsa-miR-588 | -2.920927226 | hsa-miR-325 | -2.263766865 | hsa-miR-153 | -3.779002026 |
| hsa-miR-621 | -2.924313735 | hsa-miR-643 | -2.271927646 | hsa-miR-193a-3p | -3.789739878 |
| hsa-miR-616 | -2.968962941 | hsa-miR-626 | -2.297051082 | hsa-miR-221* | -3.84919886 |
| hsa-miR-573 | -3.001238889 | hsa-miR-96* | -2.318395387 | hsa-miR-561 | -3.859606368 |
| hsa-miR-578 | -3.006446727 | hsa-miR-296-3p | -2.336925494 | hsa-miR-196a* | -3.908808113 |
| hsa-miR-218-1* | -3.037467826 | hsa-miR-375 | -2.338450962 | hsa-miR-433 | -3.926596424 |
| hsa-miR-363* | -3.040070802 | hsa-miR-330-5p | -2.339277494 | hsa-miR-92b* | -3.950521789 |
| hsa-miR-153 | -3.043743981 | hsa-miR-105* | -2.341883697 | hsa-miR-593* | -3.967362069 |
| hsa-miR-890 | -3.081924465 | hsa-miR-655 | -2.348395357 | hsa-miR-938 | -4.064020844 |
| hsa-miR-597 | -3.082049973 | hsa-miR-34c-5p | -2.387764896 | hsa-miR-96* | -4.546637361 |
| hsa-miR-24-2* | -3.086875404 | hsa-miR-431* | -2.401370268 |  |  |
| hsa-miR-214 | -3.117589267 | hsa-miR-302b* | -2.405986738 |  |  |
| hsa-miR-938 | -3.137455358 | hsa-miR-616* | -2.429605263 |  |  |
| hsa-miR-503 | -3.181208084 | hsa-miR-802 | -2.433030113 |  |  |
| hsa-miR-300 | -3.191767582 | hsa-miR-19a* | -2.438631812 |  |  |
| hsa-miR-561 | -3.198654383 | hsa-miR-646 | -2.441263562 |  |  |
| hsa-miR-888 | -3.203625925 | hsa-miR-603 | -2.446354671 |  |  |
| hsa-miR-147b | -3.24752461 | hsa-miR-433 | -2.447256842 |  |  |
| hsa-miR-593* | -3.281324918 | hsa-miR-587 | -2.448896313 |  |  |
| hsa-miR-568 | -3.305328545 | hsa-miR-145* | -2.475169431 |  |  |
| hsa-miR-876-3p | -3.311500246 | hsa-miR-1 | -2.48331696 |  |  |
| hsa-miR-339-5p | -3.33483884 | hsa-miR-943 | -2.503699271 |  |  |
| hsa-miR-337-5p | -3.358807926 | hsa-miR-27b* | -2.506288206 |  |  |
| hsa-miR-556-5p | -3.371275761 | hsa-miR-147b | -2.515925074 |  |  |
| hsa-miR-219-1-3p | -3.388005753 | hsa-miR-522 | -2.552642416 |  |  |
| hsa-miR-187 | -3.433288164 | hsa-miR-578 | -2.562310737 |  |  |
| hsa-miR-335 | -3.465356967 | hsa-miR-653 | -2.566798254 |  |  |
| hsa-miR-362-5p | -3.488117205 | hsa-miR-554 | -2.577493452 |  |  |
| hsa-miR-297 | -3.508258254 | hsa-miR-216b | -2.589849599 |  |  |
| hsa-miR-31* | -3.524021991 | hsa-miR-606 | -2.593563765 |  |  |
| hsa-miR-943 | -3.52590776 | hsa-miR-561 | -2.616545839 |  |  |
| hsa-miR-146b-3p | -3.529033792 | hsa-miR-590-3p | -2.664151458 |  |  |
| hsa-miR-92b* | -3.534150372 | hsa-miR-503 | -2.666338634 |  |  |
| hsa-miR-208a | -3.549130034 | hsa-miR-367* | -2.681290478 |  |  |
| hsa-miR-600 | -3.611802361 | hsa-miR-105 | -2.70263127 |  |  |
| hsa-miR-587 | -3.754187909 | hsa-miR-187 | -2.740190595 |  |  |
| hsa-miR-487a | -3.792811136 | hsa-miR-518c | -2.749080855 |  |  |
| hsa-miR-920 | -3.802731286 | hsa-miR-377* | -2.755708466 |  |  |
| hsa-miR-323-5p | -3.827159716 | hsa-miR-340 | -2.807882466 |  |  |
| hsa-miR-1 | -3.835304463 | hsa-miR-127-5p | -2.824293889 |  |  |
| hsa-miR-620 | -3.842328315 | hsa-miR-380* | -2.829921628 |  |  |
| hsa-miR-433 | -3.85890852 | hsa-miR-593* | -2.85175139 |  |  |
| hsa-miR-302d* | -3.952564124 | hsa-miR-588 | -2.892637379 |  |  |
| hsa-miR-296-3p | -3.973739585 | hsa-miR-27a* | -3.044541896 |  |  |
| hsa-miR-325 | -4.037523395 | hsa-miR-208a | -3.047252872 |  |  |
| hsa-miR-875-3p | -4.089458076 | hsa-miR-621 | -3.052183628 |  |  |
| hsa-miR-208b | -4.097202355 | hsa-miR-568 | -3.060463221 |  |  |
| hsa-miR-452* | -4.200094871 | hsa-miR-875-3p | -3.118921069 |  |  |
| hsa-miR-135b* | -4.266939505 | hsa-miR-499-3p | -3.121963536 |  |  |
| hsa-miR-576-3p | -4.329769967 | hsa-miR-137 | -3.125255303 |  |  |
| hsa-miR-941 | -4.349804144 | hsa-miR-335 | -3.13836159 |  |  |
| hsa-miR-1231 | -4.479351407 | hsa-miR-512-5p | -3.245830671 |  |  |
| hsa-miR-922 | -4.485758329 | hsa-miR-323-5p | -3.25830865 |  |  |
|  |  | hsa-miR-708* | -3.272163258 |  |  |
|  |  | hsa-miR-600 | -3.299025949 |  |  |
|  |  | hsa-miR-576-3p | -3.417090699 |  |  |
|  |  | hsa-miR-302d* | -3.458343011 |  |  |
|  |  | hsa-miR-24-2* | -3.468412596 |  |  |
|  |  | hsa-miR-219-1-3p | -3.494378433 |  |  |
|  |  | hsa-miR-620 | -3.504910072 |  |  |
|  |  | hsa-miR-452* | -3.600873215 |  |  |
|  |  | hsa-miR-573 | -3.64765031 |  |  |
|  |  | hsa-miR-922 | -3.689395485 |  |  |
|  |  | hsa-miR-208b | -3.748765093 |  |  |
|  |  | hsa-miR-941 | -3.902632608 |  |  |
|  |  | hsa-miR-1231 | -3.98300282 |  |  |
|  |  |  |  |  |  |

Table S4 microRNA expression in extracellular vesicles of pro-inflammatory macrophage in GSE137637

| microRNA | GSM4083583 | GSM4083584 | GSM4083585 | GSM4083590 | GSM4083591 | GSM4083592 |
| --- | --- | --- | --- | --- | --- | --- |
| hsa-let-7a-3p | 1.986254968 | 1.69516346 | 1.791634272 | 1.791730514 | 2.137908674 | 1.797699372 |
| hsa-let-7a-5p | 9.681646128 | 8.18167813 | 8.805853907 | 7.762896219 | 3.221774675 | 8.234527917 |
| hsa-let-7b-3p | 2.210442278 | 1.884478759 | 2.039057525 | 2.0413136 | 1.979451413 | 2.11694836 |
| hsa-let-7b-5p | 11.07716436 | 11.64595136 | 11.89959757 | 9.544050086 | 2.91484113 | 10.61185722 |
| hsa-let-7c-3p | 1.754223691 | 1.783514136 | 1.765891476 | 1.65648484 | 1.826986787 | 1.766773849 |
| hsa-let-7c-5p | 9.399543141 | 10.0017553 | 10.47782717 | 8.668949773 | 1.896432683 | 8.366855966 |
| hsa-let-7d-3p | 4.560814636 | 2.352877714 | 3.546576435 | 5.644252274 | 2.309708715 | 7.190316193 |
| hsa-let-7d-5p | 7.566526672 | 3.394075504 | 3.533630158 | 3.938879498 | 2.143477413 | 3.818583015 |
| hsa-let-7e-3p | 1.724785015 | 1.835273949 | 1.71147283 | 1.689398343 | 1.669786316 | 1.61895655 |
| hsa-let-7e-5p | 7.841216047 | 2.054605802 | 2.089061672 | 3.463296543 | 2.590288279 | 3.607118157 |
| hsa-let-7f-1-3p | 1.916177477 | 2.116854319 | 1.871923895 | 2.106876937 | 2.412139062 | 1.895089634 |
| hsa-let-7f-2-3p | 1.715444477 | 2.55191715 | 1.864639092 | 1.695576158 | 2.37812167 | 1.904745264 |
| hsa-let-7f-5p | 8.740896687 | 4.546239892 | 4.918230112 | 5.972885406 | 2.084265968 | 5.693750984 |
| hsa-let-7g-3p | 1.667147732 | 1.738196278 | 1.702275079 | 1.60694491 | 1.874028381 | 1.724057411 |
| hsa-let-7g-5p | 7.100122749 | 2.550472993 | 4.352949724 | 6.911701467 | 3.203136007 | 5.022398318 |
| hsa-let-7i-3p | 1.771438276 | 1.814807613 | 1.71133781 | 1.658738632 | 2.20914212 | 1.74386169 |
| hsa-let-7i-5p | 7.807906514 | 3.776115203 | 7.284730734 | 8.003495961 | 3.266495691 | 8.777155881 |
| hsa-miR-1-3p | 1.75372443 | 1.685684863 | 2.07595064 | 3.328759724 | 2.202471088 | 2.031157499 |
| hsa-miR-1-5p | 2.142432082 | 1.958759063 | 1.82109045 | 1.756354132 | 2.098125945 | 1.764915596 |
| hsa-miR-100-3p | 1.951114054 | 1.806765883 | 1.731713683 | 1.788438469 | 1.867521264 | 1.708589925 |
| hsa-miR-100-5p | 7.839602653 | 1.733709121 | 1.914614029 | 5.183378245 | 2.491122342 | 5.501736981 |
| hsa-miR-101-3p | 2.020894096 | 1.706236964 | 1.891839624 | 3.428532381 | 2.031770053 | 3.992370718 |
| hsa-miR-101-5p | 1.733009155 | 1.902624783 | 1.736717406 | 1.628306151 | 1.879502088 | 1.825739133 |
| hsa-miR-103a-2-5p | 1.644786525 | 1.878844748 | 1.781749274 | 1.622474935 | 1.839348071 | 1.709792406 |
| hsa-miR-103a-3p | 7.73213307 | 2.432052701 | 2.277148677 | 4.039742808 | 1.90923501 | 3.825760991 |
| hsa-miR-103b | 1.74763843 | 1.740456808 | 1.833194154 | 1.707608628 | 1.879736761 | 1.680650638 |
| hsa-miR-105-3p | 1.693683609 | 1.803679559 | 1.748952444 | 1.730766342 | 2.724663162 | 2.022340489 |
| hsa-miR-105-5p | 1.708776643 | 1.671278872 | 1.805157438 | 1.667277088 | 1.751864787 | 1.895676132 |
| hsa-miR-106a-3p | 1.68141024 | 1.604501767 | 1.707748758 | 1.69414113 | 1.713900771 | 1.65910557 |
| hsa-miR-106b-3p | 1.715265504 | 1.721520828 | 1.674420078 | 1.657294235 | 1.660622596 | 1.803626983 |
| hsa-miR-106b-5p | 5.425004927 | 1.821515554 | 1.961443973 | 4.774630314 | 2.110265051 | 5.457046593 |
| hsa-miR-107 | 7.647490652 | 3.005822479 | 4.428001059 | 6.996209402 | 2.659769869 | 9.261643169 |
| hsa-miR-10a-3p | 1.694607346 | 1.805357955 | 1.690385496 | 1.669673861 | 1.777388 | 1.709166284 |
| hsa-miR-10a-5p | 6.00474693 | 1.784766972 | 1.858906505 | 5.063371202 | 1.798394445 | 4.299951008 |
| hsa-miR-10b-3p | 1.75597864 | 1.803169104 | 1.95862438 | 2.039351748 | 2.071767462 | 2.469814941 |
| hsa-miR-10b-5p | 4.640780828 | 1.838937433 | 1.864829317 | 7.798274113 | 3.099681763 | 10.18451818 |
| hsa-miR-1178-3p | 1.687108723 | 2.390047029 | 1.884814386 | 1.689554287 | 2.445835037 | 1.935428792 |
| hsa-miR-1178-5p | 1.726076571 | 1.672439061 | 1.659567285 | 1.643584328 | 1.952440294 | 1.645821498 |
| hsa-miR-1179 | 1.729757303 | 2.197124112 | 2.071777817 | 1.746254368 | 2.079241739 | 1.807710937 |
| hsa-miR-1180-3p | 1.99493714 | 1.786678767 | 1.791351131 | 2.857633117 | 1.644314362 | 1.836267158 |
| hsa-miR-1180-5p | 1.579010615 | 1.692623143 | 1.793818211 | 1.606824751 | 1.744141439 | 1.685905043 |
| hsa-miR-1181 | 1.950843873 | 2.353483745 | 1.916953898 | 2.38299071 | 1.976383584 | 2.123043645 |
| hsa-miR-1182 | 2.211355991 | 1.797470818 | 1.903521006 | 6.028782305 | 1.776034783 | 1.7651209 |
| hsa-miR-1183 | 2.933752532 | 2.514165107 | 1.980228081 | 6.300404868 | 2.092013829 | 3.567068041 |
| hsa-miR-1184 | 1.805645988 | 1.946797469 | 1.677043001 | 1.713968983 | 1.704230683 | 1.719658628 |
| hsa-miR-1185-1-3p | 2.746208606 | 2.110713694 | 1.938479136 | 5.763451332 | 1.798270338 | 1.838527675 |
| hsa-miR-1185-2-3p | 1.917119041 | 2.123423463 | 1.822505425 | 4.493806188 | 1.799221105 | 1.818383992 |
| hsa-miR-1185-5p | 1.710141768 | 1.80677075 | 1.890916597 | 1.693459625 | 1.795076806 | 1.751303142 |
| hsa-miR-1193 | 1.70014859 | 1.945175727 | 1.793603699 | 1.651343457 | 1.798288576 | 1.763417695 |
| hsa-miR-1197 | 1.870240116 | 1.892332187 | 1.704707615 | 1.685670379 | 1.802516062 | 1.69350554 |
| hsa-miR-1199-3p | 1.639767714 | 1.795498202 | 1.878789672 | 1.64940367 | 1.747048464 | 1.742012514 |
| hsa-miR-1199-5p | 1.690215264 | 1.78360083 | 1.632775792 | 1.764507387 | 1.685287236 | 1.606144103 |
| hsa-miR-1200 | 1.715424358 | 1.678227469 | 1.7545805 | 1.589643351 | 1.759576001 | 1.686520491 |
| hsa-miR-1202 | 8.566944915 | 9.779897091 | 9.491488958 | 7.913172841 | 5.828117023 | 9.017226879 |
| hsa-miR-1203 | 1.664087888 | 1.661226599 | 1.773841794 | 1.668943426 | 1.809024808 | 1.719317958 |
| hsa-miR-1204 | 1.870731613 | 1.753233456 | 1.745475955 | 1.706754477 | 1.644808001 | 1.718469227 |
| hsa-miR-1205 | 1.706445793 | 1.735380925 | 1.689585031 | 1.841486326 | 1.716591756 | 1.680524724 |
| hsa-miR-1206 | 1.81309849 | 1.671391219 | 1.772542333 | 1.667178584 | 2.414084152 | 1.701991123 |
| hsa-miR-1207-3p | 1.774324484 | 1.689154438 | 1.65403828 | 1.994698381 | 1.610058189 | 1.680921315 |
| hsa-miR-1207-5p | 7.956219907 | 7.343320189 | 7.412986884 | 10.65992136 | 10.67491627 | 7.051482098 |
| hsa-miR-1208 | 1.981178758 | 1.78076615 | 1.915994139 | 2.795664483 | 1.827578024 | 2.030903852 |
| hsa-miR-122-3p | 1.898297578 | 1.78062622 | 1.693022301 | 1.674933067 | 1.804762922 | 2.067699494 |
| hsa-miR-122-5p | 1.817157094 | 1.689859083 | 1.703622443 | 3.3578763 | 2.037471276 | 1.799958498 |
| hsa-miR-1224-3p | 1.681198563 | 1.875027037 | 1.736729748 | 1.640416316 | 1.678783689 | 1.641337233 |
| hsa-miR-1224-5p | 4.587692064 | 2.81680856 | 2.286998736 | 4.871841277 | 1.785317161 | 2.025288055 |
| hsa-miR-1225-5p | 7.323941714 | 8.007417359 | 7.988159917 | 7.345638832 | 3.143671549 | 8.961229586 |
| hsa-miR-1226-3p | 1.820294892 | 1.715069514 | 1.65193652 | 1.689168616 | 1.683985171 | 1.686463587 |
| hsa-miR-1226-5p | 2.107116187 | 1.814470727 | 2.205412114 | 4.607226837 | 1.826214619 | 2.638411625 |
| hsa-miR-1227-3p | 2.028115286 | 2.526142019 | 1.73816962 | 1.884094832 | 1.845895645 | 1.91021754 |
| hsa-miR-1227-5p | 6.206429161 | 3.654384978 | 1.923761058 | 2.584434775 | 1.992663169 | 1.755798622 |
| hsa-miR-1228-3p | 6.640416737 | 8.444837626 | 8.620714276 | 4.334261447 | 11.07966949 | 7.968926517 |
| hsa-miR-1228-5p | 1.63837757 | 1.758598696 | 1.804145905 | 2.270672649 | 1.812035632 | 1.74232124 |
| hsa-miR-1229-3p | 3.050468218 | 3.057997936 | 3.73146839 | 2.600757766 | 2.921019371 | 3.752226477 |
| hsa-miR-1229-5p | 6.923533469 | 6.281157332 | 4.181469545 | 7.342079356 | 1.765224702 | 5.236720727 |
| hsa-miR-1231 | 1.643268048 | 1.880751929 | 1.700723481 | 1.606769318 | 1.701191854 | 1.628719778 |
| hsa-miR-1233-3p | 1.850948296 | 1.802027964 | 1.708515817 | 1.830200646 | 1.717983895 | 1.685971029 |
| hsa-miR-1233-5p | 2.355946075 | 1.952920195 | 1.863711282 | 4.008900155 | 1.662960135 | 1.757824947 |
| hsa-miR-1234-3p | 6.767505941 | 8.451144836 | 8.840325057 | 4.487845053 | 11.34854317 | 8.181825009 |
| hsa-miR-1236-3p | 1.59453081 | 1.618347817 | 1.61142494 | 1.676157154 | 1.742517314 | 1.684894013 |
| hsa-miR-1236-5p | 7.085033448 | 3.186428044 | 3.985413371 | 5.988906011 | 1.722451958 | 6.923525446 |
| hsa-miR-1237-3p | 4.515749307 | 4.878596474 | 6.186964516 | 2.962682588 | 8.343676085 | 5.909162992 |
| hsa-miR-1237-5p | 1.678511969 | 1.799644914 | 1.849075302 | 1.753970141 | 1.826108321 | 1.793940838 |
| hsa-miR-1238-3p | 5.83763902 | 7.203218944 | 8.254873441 | 3.712829174 | 10.80302633 | 7.588838565 |
| hsa-miR-1238-5p | 2.353646739 | 2.867359219 | 2.542959292 | 2.077887488 | 2.343325936 | 2.129127499 |
| hsa-miR-124-3p | 3.90697573 | 2.50836558 | 2.403664134 | 2.550681127 | 1.881739582 | 2.454420795 |
| hsa-miR-124-5p | 1.682971393 | 1.667657934 | 1.798698109 | 1.651659939 | 1.828606991 | 1.733139765 |
| hsa-miR-1243 | 1.841478283 | 1.743973444 | 1.750421612 | 1.655820108 | 2.455768775 | 1.991484548 |
| hsa-miR-1244 | 1.795235885 | 1.777797992 | 1.891176604 | 1.851813785 | 1.851694603 | 1.763685785 |
| hsa-miR-1245a | 1.794400617 | 1.757371047 | 1.774136506 | 1.678045664 | 2.044540776 | 2.150000555 |
| hsa-miR-1245b-3p | 1.936844764 | 1.709919674 | 1.879550981 | 1.736222227 | 1.802426301 | 1.753739232 |
| hsa-miR-1245b-5p | 1.793692221 | 1.769163449 | 1.766960683 | 1.7036918 | 1.855378459 | 1.657438791 |
| hsa-miR-1246 | 11.68435607 | 12.7831161 | 13.03727114 | 10.34486981 | 10.43046564 | 12.90195045 |
| hsa-miR-1247-3p | 2.904254965 | 1.778325901 | 1.740828851 | 2.000099613 | 1.687261076 | 1.756232056 |
| hsa-miR-1247-5p | 1.679672209 | 1.749559863 | 1.760271083 | 1.726741563 | 1.959163056 | 1.744122432 |
| hsa-miR-1248 | 1.699643647 | 1.678181547 | 2.017761515 | 1.668868805 | 1.883726725 | 1.727462523 |
| hsa-miR-1249-3p | 2.580168387 | 2.531095267 | 2.87352882 | 3.161491675 | 3.523578165 | 3.096501268 |
| hsa-miR-1249-5p | 1.963452502 | 1.751873675 | 2.196702849 | 4.292900365 | 1.792195512 | 1.900604582 |
| hsa-miR-1250-3p | 1.683563671 | 2.048558137 | 2.556321886 | 1.720878875 | 1.877815151 | 1.89259967 |
| hsa-miR-1250-5p | 1.747770587 | 1.759321368 | 1.683149529 | 1.731832081 | 1.722715759 | 1.847115356 |
| hsa-miR-1251-3p | 1.649714859 | 1.717968263 | 1.73463225 | 1.746257715 | 1.836522722 | 1.682412498 |
| hsa-miR-1251-5p | 1.614286912 | 1.650371389 | 1.75733448 | 1.634370385 | 1.727460247 | 1.719427781 |
| hsa-miR-1252-3p | 1.998712444 | 2.018752725 | 2.365267255 | 1.932408684 | 2.323151603 | 1.806739257 |
| hsa-miR-1252-5p | 1.770274963 | 1.930350852 | 2.078339185 | 1.975868276 | 4.016745255 | 1.826404407 |
| hsa-miR-1253 | 1.743183867 | 1.642758959 | 1.675035894 | 1.62195127 | 1.703198827 | 1.723137389 |
| hsa-miR-1254 | 1.685801729 | 1.74681785 | 1.610160571 | 1.726833096 | 1.667730534 | 1.719842435 |
| hsa-miR-1255a | 1.764223517 | 1.744911147 | 1.722756022 | 1.597617491 | 1.747455434 | 1.770783556 |
| hsa-miR-1255b-2-3p | 1.738679863 | 1.698097072 | 1.800287721 | 1.757503126 | 1.918317841 | 1.782290723 |
| hsa-miR-1255b-5p | 1.641597918 | 1.741252501 | 1.727082125 | 1.600395059 | 1.835988819 | 1.689485575 |
| hsa-miR-1256 | 1.824889611 | 1.826609491 | 1.925227913 | 1.671868089 | 1.967897502 | 1.78717029 |
| hsa-miR-1257 | 1.64202079 | 1.684641716 | 2.163754089 | 1.674501738 | 1.96092683 | 1.727689476 |
| hsa-miR-1258 | 1.863727609 | 1.974474238 | 1.772220286 | 1.733537713 | 1.771320571 | 1.864374108 |
| hsa-miR-125a-3p | 4.152871752 | 1.992325486 | 1.817192269 | 6.813363554 | 1.819580781 | 2.422825029 |
| hsa-miR-125a-5p | 7.086045038 | 3.58561381 | 2.246973305 | 5.7670703 | 1.767819049 | 2.909963138 |
| hsa-miR-125b-1-3p | 1.768493405 | 1.864807113 | 1.700664245 | 1.629465788 | 1.622844155 | 1.64531161 |
| hsa-miR-125b-2-3p | 5.076623922 | 2.043526929 | 2.046560005 | 3.628015084 | 1.725886452 | 2.307747131 |
| hsa-miR-125b-5p | 6.579487611 | 1.917608902 | 1.882658445 | 9.83798682 | 4.779783453 | 9.410973094 |
| hsa-miR-126-3p | 2.05111908 | 1.835275048 | 1.921654864 | 3.601632902 | 1.965505823 | 4.366396771 |
| hsa-miR-126-5p | 1.763774493 | 1.827623733 | 2.363220263 | 1.84484991 | 3.695256049 | 2.227094418 |
| hsa-miR-1260a | 4.40382075 | 6.473873954 | 4.461986061 | 6.178943489 | 6.244731377 | 8.544029449 |
| hsa-miR-1260b | 3.488759013 | 4.980776721 | 2.200952294 | 4.191536389 | 1.761412087 | 5.016623959 |
| hsa-miR-1261 | 1.683837632 | 1.658799147 | 1.698201977 | 1.622138603 | 1.884728013 | 1.837666226 |
| hsa-miR-1262 | 1.678866979 | 1.695678952 | 1.778269717 | 1.769585855 | 2.13245862 | 1.965053523 |
| hsa-miR-1263 | 1.647316587 | 1.643608205 | 1.730574186 | 1.656389905 | 1.804261821 | 1.677626644 |
| hsa-miR-1264 | 1.807298993 | 2.02281417 | 2.2101006 | 1.712147491 | 2.107090076 | 1.987832652 |
| hsa-miR-1265 | 1.789583133 | 1.846531502 | 1.745738249 | 1.741717484 | 2.104325662 | 1.721362914 |
| hsa-miR-1266-3p | 2.029725985 | 1.882712099 | 1.776129607 | 1.87341865 | 1.808317587 | 2.454669199 |
| hsa-miR-1266-5p | 1.713664567 | 1.764327414 | 1.735630968 | 1.647281925 | 1.655363499 | 1.716696373 |
| hsa-miR-1267 | 1.789922701 | 1.770251453 | 1.95484439 | 1.723963001 | 1.871408501 | 1.752154525 |
| hsa-miR-1268a | 9.64763435 | 11.40636584 | 11.19497322 | 10.96690073 | 8.140553104 | 10.66558127 |
| hsa-miR-1268b | 7.508979249 | 6.496395222 | 7.865067208 | 6.771111112 | 1.77364495 | 6.782331571 |
| hsa-miR-1269a | 1.754827837 | 1.646828697 | 1.786555392 | 1.632207997 | 1.705621335 | 1.764933776 |
| hsa-miR-1269b | 1.645542719 | 1.762282845 | 1.743783384 | 1.652046026 | 1.695414757 | 1.835856674 |
| hsa-miR-127-3p | 1.777612807 | 1.729423396 | 1.694013421 | 3.382408153 | 1.89291455 | 4.10149151 |
| hsa-miR-127-5p | 1.734280192 | 1.823759593 | 1.673268169 | 1.793565867 | 1.949966346 | 1.677839758 |
| hsa-miR-1270 | 1.725939693 | 2.433041854 | 1.798722613 | 1.653076863 | 1.835817752 | 1.735993916 |
| hsa-miR-1271-3p | 1.601738408 | 1.760946479 | 1.819024675 | 1.676578961 | 1.774002165 | 1.744741579 |
| hsa-miR-1271-5p | 1.76973365 | 1.896413519 | 1.92861742 | 1.668234065 | 1.962056981 | 1.793231387 |
| hsa-miR-1272 | 1.753621048 | 1.703793064 | 1.717392701 | 1.719137744 | 1.773384993 | 1.72518265 |
| hsa-miR-1273a | 1.741201866 | 1.778019046 | 1.746634762 | 1.726837185 | 2.331321282 | 1.738259889 |
| hsa-miR-1273c | 1.656196114 | 1.74126377 | 1.662442685 | 1.701972754 | 1.837849783 | 1.682333057 |
| hsa-miR-1273d | 1.822828142 | 1.937344194 | 2.344196876 | 1.702967091 | 1.679237689 | 1.833536947 |
| hsa-miR-1273e | 2.17675668 | 1.946060848 | 2.43155223 | 1.752759293 | 1.923489041 | 4.225547764 |
| hsa-miR-1273f | 5.764602347 | 7.890126584 | 5.311036948 | 1.938841733 | 1.526606119 | 1.777799983 |
| hsa-miR-1273g-3p | 9.272938955 | 8.932463028 | 6.487350259 | 4.527467105 | 1.8153508 | 4.324465475 |
| hsa-miR-1273g-5p | 1.76620432 | 1.820374949 | 1.763771006 | 1.680786944 | 1.667463362 | 1.658664706 |
| hsa-miR-1273h-3p | 1.713984343 | 1.673815297 | 1.885328473 | 1.715563608 | 1.983675172 | 1.860506 |
| hsa-miR-1273h-5p | 1.806975443 | 1.67145305 | 1.714373722 | 1.619541012 | 1.669213148 | 1.67213335 |
| hsa-miR-1275 | 6.41087585 | 3.968682681 | 5.757592172 | 8.119896887 | 1.804727101 | 3.577142052 |
| hsa-miR-1276 | 1.689288784 | 1.674541178 | 1.769142963 | 1.759197122 | 1.807547352 | 1.761864082 |
| hsa-miR-1277-3p | 1.896745981 | 2.231487712 | 1.894982651 | 2.022911715 | 3.480890474 | 1.848860005 |
| hsa-miR-1278 | 1.967879303 | 1.766509679 | 2.112191253 | 1.766325667 | 2.279562331 | 2.447248128 |
| hsa-miR-1279 | 1.936793372 | 1.888977598 | 1.780621081 | 1.760257353 | 2.187044013 | 1.682183546 |
| hsa-miR-128-1-5p | 1.737975166 | 1.805094009 | 1.67604195 | 1.63523348 | 1.732284863 | 1.607389384 |
| hsa-miR-128-2-5p | 1.636114015 | 1.765986727 | 1.783683691 | 1.660689522 | 1.790980486 | 1.787419859 |
| hsa-miR-128-3p | 3.591673432 | 1.713087796 | 1.948702955 | 5.695925309 | 1.993784144 | 7.672648995 |
| hsa-miR-1281 | 5.339772458 | 7.466050124 | 7.563768261 | 3.244780387 | 9.740405657 | 7.039739896 |
| hsa-miR-1282 | 1.791047084 | 1.780126846 | 1.701601799 | 1.728761669 | 1.753158268 | 1.754629374 |
| hsa-miR-1283 | 1.660663634 | 1.836155875 | 1.72227217 | 1.64144497 | 1.686544126 | 1.697168669 |
| hsa-miR-1284 | 1.654377858 | 1.700934748 | 1.733630463 | 1.619748669 | 1.767115911 | 1.802556788 |
| hsa-miR-1285-3p | 1.76031251 | 2.492620689 | 1.89964599 | 1.723211554 | 1.743831728 | 1.792675411 |
| hsa-miR-1285-5p | 1.865574548 | 2.779652289 | 2.735105652 | 1.661958225 | 1.860035449 | 1.9368142 |
| hsa-miR-1286 | 2.111989582 | 1.654381026 | 1.680845557 | 1.758845403 | 1.749754687 | 1.742069739 |
| hsa-miR-1287-3p | 1.644412676 | 1.820717277 | 1.77834499 | 1.625530445 | 1.790237898 | 1.804764209 |
| hsa-miR-1287-5p | 6.074172141 | 2.427592614 | 3.668759817 | 3.164613586 | 1.736839022 | 2.348400183 |
| hsa-miR-1288-3p | 1.739139092 | 1.887077209 | 1.82925467 | 1.726136583 | 1.748941824 | 1.961480942 |
| hsa-miR-1288-5p | 1.639856046 | 1.77002501 | 1.782219079 | 1.612029404 | 1.827346943 | 1.756489669 |
| hsa-miR-1289 | 1.854674646 | 1.744982119 | 1.614885455 | 1.75093359 | 1.755647923 | 1.675295101 |
| hsa-miR-129-1-3p | 2.42539161 | 2.121242663 | 2.626557857 | 2.528520471 | 3.555831257 | 3.026121387 |
| hsa-miR-129-2-3p | 3.241060183 | 2.1987006 | 3.448222395 | 4.602664796 | 3.492869046 | 5.43991984 |
| hsa-miR-129-5p | 1.810973745 | 1.711837586 | 1.826693843 | 1.961284039 | 1.762472731 | 1.792839897 |
| hsa-miR-1290 | 10.68909594 | 12.16383557 | 12.50114007 | 7.781826992 | 7.030778215 | 11.21433967 |
| hsa-miR-1291 | 2.678389069 | 1.675149418 | 1.717878379 | 1.721865573 | 1.841432379 | 1.672957591 |
| hsa-miR-1292-3p | 1.680914417 | 1.849940781 | 1.692222791 | 1.651859026 | 2.119809104 | 1.768436626 |
| hsa-miR-1292-5p | 1.648085341 | 1.758205054 | 1.715517768 | 1.601612083 | 1.959396011 | 1.833979677 |
| hsa-miR-1293 | 1.816210735 | 1.850548564 | 1.843146038 | 1.60142666 | 1.814266922 | 1.881857401 |
| hsa-miR-1294 | 1.722924561 | 1.91093298 | 1.990965536 | 1.738902276 | 1.984369862 | 1.801000225 |
| hsa-miR-1295a | 1.806124946 | 2.316126517 | 2.103703703 | 1.695145955 | 2.47133643 | 2.224266928 |
| hsa-miR-1295b-3p | 1.760663818 | 1.801427932 | 1.791700638 | 2.248445325 | 1.794472429 | 1.831832877 |
| hsa-miR-1295b-5p | 1.767676904 | 1.700599632 | 1.668691701 | 1.684775984 | 1.691436818 | 1.61948495 |
| hsa-miR-1296-3p | 1.758719629 | 1.810512744 | 1.638711154 | 1.694853865 | 1.775146707 | 1.64501981 |
| hsa-miR-1296-5p | 1.718368281 | 1.938199529 | 1.713315185 | 2.565869977 | 1.904513612 | 1.807982815 |
| hsa-miR-1297 | 1.635474832 | 1.842393151 | 1.948688699 | 1.68703823 | 1.867205367 | 1.814093035 |
| hsa-miR-1298-3p | 1.798178295 | 2.041238493 | 1.897524264 | 1.742019158 | 2.20899511 | 2.251672374 |
| hsa-miR-1298-5p | 1.642822898 | 1.839609913 | 1.788634087 | 1.656146326 | 1.920559012 | 1.703642935 |
| hsa-miR-1299 | 5.928565659 | 3.6488655 | 4.506122678 | 3.580250227 | 1.70844095 | 6.85642569 |
| hsa-miR-1301-3p | 1.76577123 | 1.930588848 | 1.715138016 | 1.678357532 | 1.684341646 | 1.718800329 |
| hsa-miR-1301-5p | 1.784242259 | 1.788390075 | 1.729072336 | 1.894537695 | 1.859355991 | 1.90368144 |
| hsa-miR-1302 | 1.769334903 | 1.816129915 | 1.9734739 | 1.633442556 | 2.736647793 | 1.867584523 |
| hsa-miR-1303 | 1.755431185 | 1.834552033 | 1.68281065 | 1.785672406 | 1.651001211 | 1.699902208 |
| hsa-miR-1304-3p | 5.65441346 | 7.462355843 | 7.680515138 | 3.709537988 | 10.4809044 | 7.451594357 |
| hsa-miR-1304-5p | 1.703870928 | 1.785135517 | 1.697264533 | 1.69518064 | 1.799236048 | 1.699649101 |
| hsa-miR-1305 | 2.773978531 | 1.810722137 | 1.817576108 | 5.481302639 | 1.809261762 | 2.11882237 |
| hsa-miR-1306-3p | 1.983918726 | 1.969679366 | 2.087420374 | 2.025034716 | 1.724700218 | 1.889175592 |
| hsa-miR-1306-5p | 1.913268374 | 1.751060826 | 1.670832007 | 2.563194216 | 1.617088404 | 1.852808014 |
| hsa-miR-1307-3p | 1.868860236 | 1.702763035 | 1.669037354 | 1.791050283 | 1.923034497 | 1.656145437 |
| hsa-miR-1307-5p | 1.971697805 | 1.809143813 | 1.671430128 | 2.535567047 | 1.673631177 | 1.664035105 |
| hsa-miR-130a-3p | 7.714689386 | 1.736025151 | 1.751468249 | 4.240347709 | 1.82732986 | 5.931755707 |
| hsa-miR-130a-5p | 1.688145541 | 1.724137627 | 1.723230378 | 1.714658574 | 1.789894305 | 1.705592002 |
| hsa-miR-130b-3p | 5.579468843 | 3.00515466 | 4.24246759 | 4.89580224 | 2.509250203 | 6.978601564 |
| hsa-miR-130b-5p | 1.784077008 | 1.761712752 | 1.76555024 | 1.825218487 | 1.892770877 | 1.669257308 |
| hsa-miR-132-3p | 5.433941312 | 1.686982898 | 1.747714906 | 5.605104723 | 1.818627162 | 8.187582656 |
| hsa-miR-132-5p | 1.856577489 | 1.849086956 | 1.755152121 | 1.701525525 | 1.950474051 | 1.732165344 |
| hsa-miR-1321 | 1.739084651 | 1.764716137 | 1.957271825 | 2.865000775 | 1.767303225 | 1.768504122 |
| hsa-miR-1322 | 1.694232948 | 1.78014906 | 1.715768893 | 1.655227791 | 1.691548724 | 1.72503628 |
| hsa-miR-1323 | 1.906730364 | 1.717560631 | 1.698922118 | 1.811939048 | 1.937257536 | 1.70470819 |
| hsa-miR-1324 | 1.95082162 | 1.692093597 | 1.756528921 | 1.696987804 | 1.819767468 | 1.747672518 |
| hsa-miR-133a-3p | 1.977267531 | 2.12156733 | 1.96525546 | 3.353906437 | 3.600538695 | 3.184519475 |
| hsa-miR-133a-5p | 1.694335085 | 1.664444379 | 1.6403389 | 1.664762359 | 1.839715962 | 1.709629935 |
| hsa-miR-133b | 3.234846319 | 2.152053866 | 1.917873769 | 5.312585731 | 2.137453989 | 5.583638981 |
| hsa-miR-134-3p | 1.682970915 | 1.722149631 | 1.70508685 | 1.68747698 | 1.675666246 | 1.654530592 |
| hsa-miR-134-5p | 2.882324994 | 1.979970663 | 2.226210241 | 5.239617264 | 3.000852671 | 5.916885355 |
| hsa-miR-1343-3p | 1.674090503 | 1.901761293 | 1.844460002 | 2.551989689 | 2.010998759 | 2.032529325 |
| hsa-miR-1343-5p | 2.500104803 | 1.787274838 | 2.156214948 | 4.852439308 | 1.751016542 | 4.698230666 |
| hsa-miR-135a-3p | 2.4949892 | 1.998423592 | 1.894522264 | 3.508199125 | 1.96051118 | 1.776216498 |
| hsa-miR-135a-5p | 2.045030685 | 2.27791388 | 1.87265256 | 1.826295002 | 1.851918557 | 1.874215921 |
| hsa-miR-135b-3p | 1.630519594 | 1.815736257 | 1.940681741 | 2.907082091 | 2.089405874 | 2.952622178 |
| hsa-miR-135b-5p | 1.776744377 | 1.980145987 | 2.880397638 | 2.996306526 | 4.405084636 | 2.597147178 |
| hsa-miR-136-3p | 1.891590312 | 1.755543269 | 2.086298463 | 1.76418459 | 3.014329773 | 2.284198326 |
| hsa-miR-136-5p | 2.225007777 | 2.277762872 | 2.823067055 | 1.902634663 | 2.115762316 | 2.201328636 |
| hsa-miR-137 | 3.803146413 | 1.747076573 | 1.729996618 | 1.72210184 | 1.792370803 | 1.703975123 |
| hsa-miR-138-1-3p | 1.671268095 | 1.785552623 | 1.714200121 | 1.654598967 | 1.715608499 | 1.72774141 |
| hsa-miR-138-2-3p | 1.660414502 | 1.983689922 | 1.686642951 | 2.214282877 | 1.726689158 | 1.803222826 |
| hsa-miR-138-5p | 1.809865919 | 1.85463805 | 1.708470147 | 2.213364464 | 1.805241105 | 1.761197467 |
| hsa-miR-139-3p | 1.693474804 | 1.672581184 | 1.933340307 | 1.676384669 | 1.781875908 | 1.845412067 |
| hsa-miR-139-5p | 1.683492159 | 1.72013166 | 1.766016145 | 1.957332506 | 1.799595078 | 1.688183004 |
| hsa-miR-140-3p | 4.123254684 | 2.739694009 | 2.160917766 | 7.696162735 | 1.848855694 | 9.560251028 |
| hsa-miR-140-5p | 1.957098367 | 2.082642459 | 2.138388242 | 6.211607821 | 2.531437822 | 8.051167176 |
| hsa-miR-141-3p | 1.76462193 | 1.873757831 | 1.678555581 | 2.871846348 | 1.684254003 | 1.871984104 |
| hsa-miR-141-5p | 1.727410599 | 1.67141042 | 1.729533512 | 1.718202746 | 1.6659761 | 1.824214448 |
| hsa-miR-142-3p | 2.268129977 | 2.927775719 | 2.254799714 | 3.1438144 | 2.401104373 | 5.247530853 |
| hsa-miR-142-5p | 1.646601946 | 1.780373367 | 1.760231608 | 3.184070149 | 1.992686124 | 1.921273445 |
| hsa-miR-143-3p | 1.717827335 | 1.75130355 | 1.648987242 | 5.458258258 | 2.045452296 | 5.655027361 |
| hsa-miR-143-5p | 1.69177458 | 1.949110559 | 1.745608026 | 1.795861733 | 1.878594156 | 1.732449975 |
| hsa-miR-144-3p | 2.462186074 | 2.056800571 | 1.858024107 | 2.547709703 | 2.021992107 | 2.10989035 |
| hsa-miR-144-5p | 1.809631499 | 1.792988455 | 2.080542641 | 1.79620816 | 1.876330378 | 2.106611433 |
| hsa-miR-145-3p | 1.805385053 | 1.737335137 | 1.870746087 | 3.397917211 | 1.953796725 | 2.048063918 |
| hsa-miR-145-5p | 1.71895324 | 1.731023029 | 2.031058689 | 9.942654058 | 5.150277521 | 10.15944508 |
| hsa-miR-1468-3p | 1.760264617 | 1.661092111 | 1.80632019 | 1.622058444 | 1.964219009 | 1.864576991 |
| hsa-miR-1468-5p | 1.698115631 | 1.796578539 | 1.70211951 | 1.682520424 | 1.669970508 | 1.624278862 |
| hsa-miR-1469 | 1.720701946 | 1.675542668 | 1.691131098 | 2.229894054 | 1.754244375 | 1.663628555 |
| hsa-miR-146a-3p | 1.746982389 | 1.853804656 | 1.892177549 | 1.670528419 | 2.92468105 | 1.809164798 |
| hsa-miR-146a-5p | 2.705359347 | 5.301722062 | 6.721904321 | 9.565863076 | 4.76892167 | 10.88900798 |
| hsa-miR-146b-3p | 1.689238585 | 1.672987278 | 1.68548916 | 1.706821789 | 1.812812322 | 1.756341811 |
| hsa-miR-146b-5p | 1.777774758 | 1.980999574 | 1.771369733 | 2.723745777 | 1.930962526 | 1.761623454 |
| hsa-miR-1470 | 1.860106266 | 2.190578068 | 1.969470185 | 1.721244002 | 1.878264866 | 1.95128442 |
| hsa-miR-1471 | 1.926032091 | 1.793242573 | 1.701415977 | 5.286644989 | 1.771959303 | 1.749956856 |
| hsa-miR-147a | 1.700071036 | 1.723561521 | 1.828509239 | 1.642435908 | 1.83214267 | 1.733992902 |
| hsa-miR-147b | 1.621814963 | 1.807195465 | 1.704218938 | 1.570930683 | 1.75945086 | 1.82391979 |
| hsa-miR-148a-3p | 2.501475835 | 1.925625762 | 2.201588116 | 6.879292102 | 3.655559341 | 9.421963478 |
| hsa-miR-148a-5p | 1.749507445 | 1.7156096 | 1.695176489 | 1.686530391 | 1.774975219 | 1.77813266 |
| hsa-miR-148b-3p | 1.845094678 | 1.758478447 | 1.948962338 | 3.678667063 | 1.80204542 | 3.796369388 |
| hsa-miR-148b-5p | 1.704536138 | 1.762354427 | 1.927989433 | 1.66280877 | 2.417805561 | 1.724239927 |
| hsa-miR-149-3p | 1.77454321 | 1.816732566 | 1.688552991 | 2.649988346 | 1.758891514 | 1.619731213 |
| hsa-miR-149-5p | 5.431335292 | 2.681428649 | 1.952249061 | 2.622238537 | 2.860795315 | 2.47176326 |
| hsa-miR-150-3p | 6.828796978 | 4.392839486 | 4.748602465 | 5.223074146 | 1.764945569 | 2.796380057 |
| hsa-miR-150-5p | 2.102770982 | 1.751296523 | 1.88929611 | 4.438534172 | 2.127716867 | 2.04388716 |
| hsa-miR-151a-3p | 4.426938925 | 1.691054129 | 1.682142106 | 3.525745524 | 1.801402101 | 3.106743457 |
| hsa-miR-151a-5p | 5.881178023 | 1.836990597 | 1.883684259 | 3.385663844 | 1.81220519 | 2.098669499 |
| hsa-miR-151b | 5.665418785 | 3.150296126 | 4.523333722 | 6.443366579 | 2.041234285 | 7.856680798 |
| hsa-miR-152-3p | 1.683389596 | 1.737254262 | 1.833327402 | 2.641011983 | 1.722410022 | 3.518104043 |
| hsa-miR-152-5p | 1.700947155 | 1.790448091 | 1.865607897 | 1.719526051 | 2.139099109 | 1.819812888 |
| hsa-miR-153-3p | 1.730355111 | 1.6936318 | 1.86331253 | 1.709298786 | 1.719676706 | 1.895138003 |
| hsa-miR-153-5p | 1.656498016 | 1.923214086 | 1.75663988 | 1.6401236 | 2.092379489 | 1.687635733 |
| hsa-miR-1537-3p | 1.711027457 | 1.975971035 | 1.857060689 | 1.622041875 | 1.944085224 | 1.798472024 |
| hsa-miR-1537-5p | 1.924665264 | 1.808519037 | 1.986553419 | 1.722336388 | 2.202754909 | 1.977016365 |
| hsa-miR-1538 | 1.727043216 | 2.21853224 | 1.767320036 | 1.701908109 | 1.855835297 | 1.887550241 |
| hsa-miR-1539 | 2.15591326 | 2.454828587 | 2.437000113 | 2.260479588 | 3.014070221 | 2.393128499 |
| hsa-miR-154-3p | 1.87619484 | 1.655803216 | 1.826480422 | 1.778085047 | 1.747881563 | 1.798793417 |
| hsa-miR-154-5p | 2.0214485 | 1.809024318 | 1.812700393 | 2.100614452 | 2.281258339 | 2.574300382 |
| hsa-miR-155-3p | 1.858753753 | 1.845914373 | 1.74035548 | 1.735645594 | 1.753281915 | 1.740325612 |
| hsa-miR-155-5p | 6.931215413 | 6.459838957 | 5.853746214 | 5.877624308 | 1.853742345 | 2.666609126 |
| hsa-miR-1587 | 5.364684427 | 4.775305117 | 4.396388466 | 6.316393655 | 1.823797097 | 5.731166323 |
| hsa-miR-15a-3p | 1.717919447 | 1.669626339 | 1.776803724 | 1.640219377 | 1.892322181 | 1.769807512 |
| hsa-miR-15a-5p | 5.466454734 | 4.205433465 | 6.464737818 | 6.32465739 | 2.457052333 | 8.860776086 |
| hsa-miR-15b-3p | 2.392616364 | 2.21991492 | 1.915994642 | 1.78244392 | 1.792293501 | 1.735734127 |
| hsa-miR-15b-5p | 8.850971512 | 5.718481062 | 7.712470883 | 7.784900454 | 2.046577585 | 8.447933638 |
| hsa-miR-16-1-3p | 1.632702257 | 1.742267485 | 1.7010619 | 1.790785085 | 1.744622763 | 1.691692896 |
| hsa-miR-16-2-3p | 2.004287778 | 1.814213645 | 1.809883324 | 1.796194499 | 2.108741646 | 1.717998971 |
| hsa-miR-16-5p | 9.77632047 | 10.09984157 | 10.88446382 | 9.681779055 | 5.185852563 | 11.09749895 |
| hsa-miR-17-3p | 1.595556291 | 1.927253676 | 1.767026169 | 1.724853375 | 1.723178863 | 1.919935195 |
| hsa-miR-17-5p | 7.352120754 | 8.812637628 | 9.762961693 | 7.13252396 | 1.667954611 | 8.933180715 |
| hsa-miR-181a-2-3p | 1.883527854 | 1.649504617 | 1.714290057 | 1.581048599 | 1.744073695 | 1.789267114 |
| hsa-miR-181a-3p | 2.121777044 | 1.731201142 | 1.763830112 | 1.645510512 | 1.836240074 | 1.703698261 |
| hsa-miR-181a-5p | 6.760916258 | 1.832021032 | 1.980350756 | 2.880496896 | 1.962889464 | 1.878115679 |
| hsa-miR-181b-2-3p | 1.89871871 | 1.892640742 | 1.67204174 | 1.797032606 | 1.76761017 | 1.702111856 |
| hsa-miR-181b-3p | 1.738098898 | 1.912841372 | 1.742477755 | 1.711395477 | 1.91451868 | 1.729760814 |
| hsa-miR-181b-5p | 6.787240272 | 1.745834596 | 2.123225302 | 3.432508787 | 1.716817934 | 1.782007648 |
| hsa-miR-181c-3p | 1.723975875 | 1.948796255 | 1.738815826 | 1.781144008 | 1.728520839 | 1.711508097 |
| hsa-miR-181c-5p | 1.836892412 | 2.018853606 | 1.828018694 | 2.238748917 | 1.941041531 | 1.818651654 |
| hsa-miR-181d-3p | 1.718602032 | 1.759416614 | 1.886730818 | 1.678432743 | 1.826931295 | 1.786118934 |
| hsa-miR-181d-5p | 2.716347258 | 1.782096016 | 1.896127762 | 3.433424282 | 1.842456794 | 1.74428342 |
| hsa-miR-182-3p | 1.812217269 | 1.860516598 | 1.743069231 | 1.770604812 | 1.873044421 | 1.684595862 |
| hsa-miR-182-5p | 2.037761026 | 1.780545794 | 1.685246409 | 1.630484943 | 1.652660782 | 1.608852655 |
| hsa-miR-1825 | 5.264034466 | 6.981232179 | 7.276563797 | 3.198131744 | 9.382741994 | 6.70872794 |
| hsa-miR-1827 | 1.694596958 | 1.783414714 | 2.203439712 | 1.615161988 | 2.070218239 | 1.817221757 |
| hsa-miR-183-3p | 2.140058846 | 1.766251581 | 1.71933746 | 1.698260568 | 1.673074371 | 1.691817779 |
| hsa-miR-183-5p | 5.067398164 | 1.910308736 | 1.810370056 | 2.291064836 | 1.843566009 | 1.7687335 |
| hsa-miR-184 | 1.999053761 | 1.818323969 | 2.26504694 | 2.026581712 | 2.11859282 | 2.253901774 |
| hsa-miR-185-3p | 1.646869551 | 2.019383567 | 1.890267918 | 1.653062673 | 1.720152648 | 1.854948688 |
| hsa-miR-185-5p | 2.165759827 | 1.779280016 | 1.80589944 | 5.500514969 | 1.729663341 | 8.24292401 |
| hsa-miR-186-3p | 1.998344445 | 2.333824576 | 2.343810372 | 4.274221039 | 2.57693163 | 5.754079658 |
| hsa-miR-186-5p | 3.150630468 | 2.690873108 | 2.4576291 | 5.242870006 | 2.370736619 | 6.149566415 |
| hsa-miR-187-3p | 1.774130883 | 1.641238109 | 1.627892927 | 1.641131432 | 1.763779437 | 1.630084666 |
| hsa-miR-187-5p | 1.617726516 | 1.785129485 | 1.709218994 | 1.803730349 | 1.66200641 | 1.645095645 |
| hsa-miR-188-3p | 1.640782624 | 1.707565502 | 1.954603857 | 1.643182806 | 1.941772797 | 1.741460404 |
| hsa-miR-188-5p | 6.102482282 | 4.836994599 | 5.753737533 | 6.194582849 | 1.896683571 | 7.012412284 |
| hsa-miR-18a-3p | 1.720384535 | 1.867801898 | 1.764857702 | 1.733960445 | 1.814725824 | 1.740750692 |
| hsa-miR-18a-5p | 2.315142488 | 2.302840564 | 2.535153521 | 3.005447611 | 1.787812461 | 2.48267038 |
| hsa-miR-18b-3p | 2.030832524 | 1.95540027 | 2.296902587 | 2.083415663 | 2.773543301 | 1.921025191 |
| hsa-miR-18b-5p | 1.929913056 | 1.789271701 | 1.916888344 | 2.236397064 | 1.868558281 | 1.791707217 |
| hsa-miR-1908-3p | 5.470394992 | 5.848907082 | 5.820244239 | 7.685842567 | 6.459644269 | 5.830996561 |
| hsa-miR-1908-5p | 1.626769774 | 1.754061102 | 1.82492325 | 1.63342818 | 1.660536113 | 1.761047394 |
| hsa-miR-1909-3p | 1.70419515 | 1.737215951 | 1.796059519 | 1.737544275 | 2.016107071 | 1.770222047 |
| hsa-miR-1909-5p | 1.888956849 | 1.774823095 | 2.052252314 | 1.803425457 | 1.910520135 | 1.697862223 |
| hsa-miR-190a-3p | 1.884608626 | 1.868046054 | 1.813789067 | 1.856930453 | 1.937385991 | 1.764134194 |
| hsa-miR-190a-5p | 2.160010786 | 2.012472261 | 1.879604664 | 1.828234349 | 2.791091678 | 1.943969331 |
| hsa-miR-190b | 1.806796625 | 1.850451641 | 2.005937453 | 1.872787631 | 2.044662392 | 1.868870533 |
| hsa-miR-191-3p | 5.701315709 | 6.920525286 | 7.897812773 | 3.572932823 | 10.37863367 | 7.314453359 |
| hsa-miR-191-5p | 1.76630782 | 1.644904631 | 1.627992389 | 1.948323405 | 1.713283237 | 1.623416124 |
| hsa-miR-1910-3p | 1.794627714 | 1.762067409 | 1.744499761 | 1.923908948 | 1.843969324 | 2.041757639 |
| hsa-miR-1910-5p | 1.725405175 | 1.920766056 | 1.690295526 | 2.191475797 | 2.110012059 | 1.672325858 |
| hsa-miR-1911-3p | 1.678865382 | 1.687496143 | 1.719045877 | 1.661174875 | 1.800299701 | 1.797872822 |
| hsa-miR-1911-5p | 1.996122416 | 2.236024731 | 2.043006104 | 4.202817123 | 2.101420923 | 4.476620861 |
| hsa-miR-1912 | 1.778839392 | 1.700373282 | 1.792650672 | 1.702669829 | 1.892758581 | 1.687324617 |
| hsa-miR-1913 | 1.687599316 | 1.743313696 | 1.917774132 | 1.648189221 | 1.748989442 | 1.895348766 |
| hsa-miR-1914-3p | 8.816419216 | 2.312720035 | 2.311047448 | 4.53839055 | 1.621616218 | 6.56233703 |
| hsa-miR-1914-5p | 1.686764571 | 1.685799057 | 1.830144852 | 1.700451842 | 1.900522758 | 1.862534102 |
| hsa-miR-1915-3p | 9.180298482 | 9.330454288 | 8.72381439 | 8.952897366 | 3.080348028 | 7.138391249 |
| hsa-miR-1915-5p | 1.65733809 | 1.790593004 | 1.694099122 | 2.281952571 | 1.725455707 | 1.718045134 |
| hsa-miR-192-3p | 1.703680159 | 1.891906974 | 1.722173703 | 1.677940188 | 1.781782397 | 1.854570762 |
| hsa-miR-192-5p | 1.889674175 | 1.764708348 | 1.817806378 | 2.79186105 | 1.794519528 | 1.81955204 |
| hsa-miR-193a-3p | 1.621639608 | 1.987275519 | 2.27240611 | 2.085247366 | 2.128230232 | 3.552494155 |
| hsa-miR-193a-5p | 3.276330923 | 3.47138743 | 2.843172155 | 3.887964563 | 1.764267099 | 4.461528275 |
| hsa-miR-193b-3p | 5.86513698 | 1.656768577 | 1.712629857 | 2.450319706 | 1.762944334 | 1.788401842 |
| hsa-miR-193b-5p | 3.070775919 | 2.48420133 | 1.911641379 | 3.463101286 | 1.82067019 | 1.965542857 |
| hsa-miR-194-3p | 2.11974361 | 1.997104862 | 1.886137119 | 1.724040905 | 2.259790803 | 1.754690907 |
| hsa-miR-194-5p | 1.746719277 | 1.644166159 | 1.712195849 | 1.706931964 | 1.651427667 | 1.688471692 |
| hsa-miR-195-3p | 1.770463651 | 1.772638581 | 1.810981742 | 1.881950131 | 1.841341399 | 1.652649793 |
| hsa-miR-195-5p | 1.966870732 | 1.69156618 | 1.792044512 | 5.431093966 | 1.838182034 | 5.813572653 |
| hsa-miR-196a-3p | 1.654388834 | 1.71538204 | 1.722173328 | 1.669361474 | 1.676173502 | 1.778228391 |
| hsa-miR-196a-5p | 4.065878208 | 2.090533548 | 2.48402641 | 4.178837453 | 2.506183997 | 5.869628065 |
| hsa-miR-196b-3p | 1.659491422 | 1.724068409 | 1.736138771 | 1.64257167 | 1.731534796 | 1.809167102 |
| hsa-miR-196b-5p | 4.6620058 | 2.355394859 | 2.36582972 | 3.759558781 | 2.338396682 | 4.340183859 |
| hsa-miR-197-3p | 8.211543308 | 7.869654099 | 8.35203728 | 4.565799468 | 2.331711594 | 4.920910498 |
| hsa-miR-197-5p | 9.736769659 | 11.2715321 | 11.60842354 | 10.88885844 | 12.10447412 | 13.51853548 |
| hsa-miR-1972 | 2.580378927 | 5.169803392 | 4.480721825 | 1.712170675 | 1.744109465 | 1.770102519 |
| hsa-miR-1973 | 1.704755538 | 1.715874214 | 1.679266184 | 1.725906356 | 1.629317101 | 1.674486671 |
| hsa-miR-1976 | 1.76648388 | 1.903018118 | 1.746310329 | 1.731874713 | 2.074822354 | 1.726768077 |
| hsa-miR-198 | 1.904187532 | 1.693959231 | 1.659904835 | 2.957946367 | 1.74211192 | 1.694634411 |
| hsa-miR-199a-3p | 1.725194264 | 1.995546393 | 1.818491889 | 7.307553174 | 2.507461541 | 8.789991313 |
| hsa-miR-199a-5p | 1.699182956 | 1.875492907 | 1.806028453 | 5.433936227 | 2.055772341 | 3.488927004 |
| hsa-miR-199b-5p | 1.982301765 | 1.966026539 | 1.923582891 | 3.847725192 | 2.178088231 | 3.149343933 |
| hsa-miR-19a-3p | 4.310788667 | 5.944459453 | 8.38542576 | 4.36060266 | 4.045041422 | 8.541865307 |
| hsa-miR-19a-5p | 1.758463005 | 1.984809023 | 1.830871023 | 1.617108029 | 2.025907066 | 1.839685952 |
| hsa-miR-19b-1-5p | 1.647138065 | 1.866163748 | 1.733222204 | 1.617720273 | 1.789217322 | 1.916831578 |
| hsa-miR-19b-2-5p | 1.861311949 | 1.683959124 | 1.878650073 | 1.768649872 | 3.095436344 | 1.779452997 |
| hsa-miR-19b-3p | 7.546128641 | 9.213033621 | 9.890142894 | 7.140561249 | 3.479242633 | 10.75337213 |
| hsa-miR-200a-3p | 2.826308431 | 1.758078016 | 1.84331157 | 2.778922269 | 1.708887154 | 1.768226937 |
| hsa-miR-200a-5p | 2.063259782 | 1.721695261 | 1.68727801 | 4.146530922 | 1.621078027 | 1.643796772 |
| hsa-miR-200b-3p | 6.626576769 | 1.786141968 | 1.723043856 | 2.423741677 | 1.731266539 | 1.770925911 |
| hsa-miR-200b-5p | 1.657015691 | 1.832801336 | 1.77170048 | 1.794603241 | 1.841850184 | 1.818398879 |
| hsa-miR-200c-3p | 1.710113138 | 1.738501792 | 1.763638716 | 2.809170236 | 1.854696645 | 1.753025056 |
| hsa-miR-200c-5p | 1.650505574 | 1.618847773 | 1.814415818 | 1.747258069 | 1.881845511 | 1.733395602 |
| hsa-miR-202-3p | 4.558270092 | 1.91643765 | 1.850908023 | 3.203725969 | 1.743386183 | 1.817842441 |
| hsa-miR-202-5p | 1.773383023 | 1.973699671 | 1.943651352 | 1.800411088 | 1.990718031 | 1.990412503 |
| hsa-miR-203a-3p | 1.684549444 | 1.962052513 | 1.809896074 | 2.512186752 | 1.929344271 | 1.797265933 |
| hsa-miR-203a-5p | 1.799364852 | 1.694667608 | 1.869281558 | 1.721029012 | 2.211983891 | 1.817654614 |
| hsa-miR-203b-3p | 1.664413055 | 1.767314302 | 1.829923367 | 1.651334159 | 1.725082887 | 2.168075507 |
| hsa-miR-203b-5p | 1.688650169 | 1.865042123 | 1.818955355 | 1.634530745 | 1.822100306 | 1.895551893 |
| hsa-miR-204-3p | 1.879088735 | 1.741431247 | 1.65291678 | 1.753220952 | 2.163739109 | 1.679879421 |
| hsa-miR-204-5p | 1.905249945 | 1.707810592 | 1.710872486 | 4.312362681 | 1.816448518 | 1.931746218 |
| hsa-miR-205-3p | 1.786246592 | 1.73319669 | 1.687000458 | 1.698999524 | 1.74537264 | 1.688862392 |
| hsa-miR-205-5p | 1.740798875 | 1.689287558 | 1.645827034 | 3.391192072 | 1.735088285 | 1.76609151 |
| hsa-miR-2052 | 2.243347691 | 1.784017152 | 1.719218759 | 1.827869735 | 1.844141999 | 1.768415763 |
| hsa-miR-2053 | 1.958277796 | 2.600748304 | 2.191146185 | 1.721878327 | 2.448647675 | 2.438162465 |
| hsa-miR-206 | 1.784936301 | 1.804581445 | 1.874244721 | 2.051853349 | 1.750064867 | 1.755405426 |
| hsa-miR-208a-3p | 1.643685544 | 1.647012126 | 1.737328485 | 1.64134992 | 1.833286722 | 1.729538745 |
| hsa-miR-208a-5p | 1.748572579 | 1.715390593 | 1.879538835 | 3.612874168 | 1.806863869 | 1.721688667 |
| hsa-miR-208b-3p | 1.733967596 | 1.623196771 | 1.784420533 | 1.707674963 | 2.252419122 | 1.751368122 |
| hsa-miR-208b-5p | 1.816088268 | 1.913879684 | 2.047425638 | 1.726875723 | 2.104906562 | 1.869349128 |
| hsa-miR-20a-3p | 1.800954141 | 1.701615781 | 1.802225525 | 1.72842026 | 1.87900565 | 1.713797368 |
| hsa-miR-20a-5p | 8.061552659 | 9.327761865 | 10.66046752 | 8.091334702 | 2.267611233 | 10.49257291 |
| hsa-miR-20b-3p | 1.68016206 | 1.720958742 | 1.749996853 | 1.66025801 | 1.662967031 | 1.703876168 |
| hsa-miR-20b-5p | 5.306781588 | 5.577422952 | 8.198555829 | 6.793858807 | 2.254503111 | 8.28067242 |
| hsa-miR-21-3p | 1.881091039 | 1.862036481 | 1.700532991 | 1.930767637 | 1.713691266 | 1.780897714 |
| hsa-miR-21-5p | 10.80543694 | 7.71589535 | 8.875328017 | 11.51434162 | 9.499387474 | 12.13710393 |
| hsa-miR-210-3p | 4.282191563 | 1.95349675 | 1.944056884 | 3.6941767 | 1.91870594 | 8.385942725 |
| hsa-miR-210-5p | 1.878652589 | 1.795558655 | 1.771854795 | 1.933096045 | 2.207853148 | 1.810627741 |
| hsa-miR-211-3p | 4.863291134 | 2.569300039 | 2.447497612 | 2.515906547 | 1.996579024 | 2.127677386 |
| hsa-miR-211-5p | 1.888828967 | 1.86727978 | 1.860844594 | 1.895379791 | 2.45830313 | 1.785416184 |
| hsa-miR-2110 | 1.567961776 | 1.738845647 | 1.709070408 | 1.633822531 | 1.787599302 | 1.725120553 |
| hsa-miR-2113 | 1.694623572 | 1.79788268 | 1.815846655 | 1.661195652 | 1.921241323 | 1.813634705 |
| hsa-miR-2114-3p | 1.713825338 | 1.969670334 | 1.959065054 | 1.69552743 | 1.972639931 | 1.990401038 |
| hsa-miR-2114-5p | 1.65201059 | 1.727235941 | 1.987643585 | 1.546264996 | 1.987593989 | 1.69304685 |
| hsa-miR-2115-3p | 1.759927075 | 1.655911342 | 1.773422458 | 1.655479993 | 1.91855707 | 1.773802128 |
| hsa-miR-2115-5p | 1.632693818 | 1.891336105 | 1.800862127 | 1.692842951 | 1.858980458 | 1.780089484 |
| hsa-miR-2116-3p | 1.936958986 | 2.646220551 | 1.923125095 | 1.836777276 | 2.088120894 | 1.935662403 |
| hsa-miR-2116-5p | 1.816402965 | 2.051633419 | 1.744520275 | 1.777172635 | 1.831648495 | 1.785161138 |
| hsa-miR-2117 | 1.728040696 | 1.693398281 | 1.730889475 | 1.755106163 | 1.862574429 | 1.704449932 |
| hsa-miR-212-3p | 1.831839209 | 1.887874457 | 1.729783058 | 2.287235739 | 1.820387699 | 2.129363143 |
| hsa-miR-212-5p | 1.725404962 | 1.775997357 | 1.687053911 | 1.86524266 | 1.949725254 | 1.692485558 |
| hsa-miR-214-3p | 1.66658414 | 1.793437238 | 1.711984901 | 6.639956496 | 1.799606458 | 6.495450613 |
| hsa-miR-214-5p | 1.67081936 | 1.934513076 | 1.721501851 | 2.153050092 | 2.143746026 | 1.729452923 |
| hsa-miR-215-3p | 1.763278726 | 2.023636942 | 1.831924764 | 1.804641093 | 1.864114396 | 1.731267876 |
| hsa-miR-215-5p | 2.338709673 | 1.794735056 | 1.66318467 | 2.551107191 | 1.939754283 | 1.838208202 |
| hsa-miR-216a-3p | 1.766211377 | 1.777807705 | 1.809582652 | 1.755881261 | 1.792572514 | 1.757770448 |
| hsa-miR-216a-5p | 1.627844159 | 1.819309339 | 1.732610707 | 1.65245726 | 1.698557969 | 1.726040454 |
| hsa-miR-216b-3p | 1.675243932 | 1.832616114 | 1.736213797 | 1.662211463 | 1.886853932 | 1.886666248 |
| hsa-miR-216b-5p | 1.652439356 | 1.758269532 | 1.770438808 | 1.859938111 | 1.861169116 | 1.748970677 |
| hsa-miR-217 | 1.885930523 | 1.836225988 | 1.703287794 | 1.785881191 | 1.695088549 | 1.693262398 |
| hsa-miR-218-1-3p | 1.679253712 | 1.809434818 | 1.716405263 | 1.629238602 | 1.848993887 | 1.659254857 |
| hsa-miR-218-2-3p | 1.664935906 | 1.78361524 | 1.614413163 | 1.626180637 | 1.740401958 | 1.655126046 |
| hsa-miR-218-5p | 1.859588446 | 1.769845928 | 1.808134272 | 2.206068176 | 1.984031527 | 2.180167827 |
| hsa-miR-219a-1-3p | 1.655643385 | 1.790105216 | 1.755229515 | 1.619356397 | 1.935298202 | 1.656003638 |
| hsa-miR-219a-2-3p | 1.667070291 | 1.733402435 | 1.76331034 | 1.671934923 | 1.806086634 | 1.674596135 |
| hsa-miR-219a-5p | 1.732579715 | 1.818924897 | 2.039535183 | 1.73992715 | 2.423849654 | 2.918175449 |
| hsa-miR-219b-3p | 1.625588253 | 1.749636764 | 1.921175839 | 1.637093879 | 1.805304448 | 1.877400604 |
| hsa-miR-219b-5p | 1.658920486 | 1.66588089 | 1.726698885 | 1.680375352 | 1.782845457 | 1.706665247 |
| hsa-miR-22-3p | 7.004873922 | 1.915800771 | 2.079264943 | 9.42720719 | 9.208444025 | 11.780823 |
| hsa-miR-22-5p | 2.004897389 | 1.84639217 | 2.122107943 | 2.497879951 | 1.85889083 | 2.017336155 |
| hsa-miR-221-3p | 8.052464635 | 5.465076689 | 5.427990523 | 7.5780127 | 2.045321013 | 8.696544331 |
| hsa-miR-221-5p | 1.873427647 | 1.718628283 | 1.785427174 | 1.718077457 | 1.774007332 | 1.70323056 |
| hsa-miR-222-3p | 5.047034915 | 3.345604435 | 1.870533183 | 3.904623288 | 1.682556032 | 3.96075037 |
| hsa-miR-222-5p | 1.710620384 | 1.677366645 | 1.665152754 | 1.623702592 | 1.753250921 | 1.712078382 |
| hsa-miR-223-3p | 6.128094551 | 8.305487797 | 9.491018823 | 10.39583553 | 6.51202464 | 11.38962046 |
| hsa-miR-223-5p | 1.733981127 | 1.731078987 | 1.849048872 | 1.715305672 | 2.678057175 | 1.773230066 |
| hsa-miR-224-3p | 1.696094415 | 1.983327893 | 1.886707952 | 1.628089836 | 2.146334559 | 1.851265903 |
| hsa-miR-224-5p | 2.197224526 | 1.759411785 | 1.701335931 | 2.799650183 | 1.713018594 | 2.261139927 |
| hsa-miR-2276-3p | 4.911508374 | 2.817948884 | 2.07908439 | 4.749654534 | 1.760170908 | 2.786351152 |
| hsa-miR-2276-5p | 1.67408217 | 1.831009558 | 1.808501912 | 1.636641721 | 1.780538334 | 1.767756146 |
| hsa-miR-2277-3p | 1.944467969 | 1.726728638 | 1.803655056 | 1.810983324 | 1.769271096 | 1.63013807 |
| hsa-miR-2277-5p | 1.653885132 | 1.859867388 | 1.743938176 | 1.70356203 | 1.753826255 | 1.705476003 |
| hsa-miR-2278 | 1.787889973 | 1.853215117 | 2.630523007 | 1.815181864 | 1.882179412 | 1.840968956 |
| hsa-miR-2355-3p | 1.843028449 | 1.888027754 | 1.79462866 | 1.673936074 | 1.717332209 | 1.73401471 |
| hsa-miR-2355-5p | 1.916559056 | 1.716500103 | 1.997276185 | 1.617061948 | 1.770804642 | 1.708423822 |
| hsa-miR-2392 | 10.28573028 | 2.606371086 | 5.810808545 | 6.649749666 | 1.759809772 | 3.710214938 |
| hsa-miR-23a-3p | 9.833960414 | 9.588909734 | 10.01609133 | 6.552147994 | 2.977881691 | 9.570450415 |
| hsa-miR-23a-5p | 1.938566077 | 1.818801529 | 1.841428557 | 2.081375186 | 1.845609929 | 1.641989264 |
| hsa-miR-23b-3p | 4.850576302 | 1.877769402 | 1.699401001 | 5.145962436 | 1.790467894 | 4.427140023 |
| hsa-miR-23b-5p | 1.778113518 | 1.627850138 | 1.827504239 | 1.740040851 | 1.828425741 | 1.694903225 |
| hsa-miR-23c | 1.953258718 | 2.060793849 | 1.922481796 | 1.919733185 | 2.224248819 | 1.820265741 |
| hsa-miR-24-1-5p | 1.749022403 | 1.828746193 | 1.733274409 | 1.770300788 | 1.885937474 | 1.762108785 |
| hsa-miR-24-2-5p | 1.650546653 | 1.722728206 | 1.772345263 | 1.69008782 | 1.701304975 | 1.754696654 |
| hsa-miR-24-3p | 8.56736008 | 7.207942682 | 8.183314069 | 8.951247602 | 7.676099726 | 11.30614459 |
| hsa-miR-2467-3p | 1.939309613 | 1.872085443 | 1.840684094 | 2.711007138 | 1.684606394 | 1.781653279 |
| hsa-miR-2467-5p | 1.654895769 | 1.757109576 | 1.713228076 | 1.655941906 | 1.798281304 | 1.747934549 |
| hsa-miR-25-3p | 8.244111632 | 5.632525004 | 7.728961899 | 8.089064997 | 3.664225962 | 9.775506382 |
| hsa-miR-25-5p | 1.768530925 | 1.702013938 | 1.809141881 | 1.661360826 | 1.752886334 | 1.687155601 |
| hsa-miR-2681-3p | 1.706414909 | 2.676371898 | 1.710466861 | 1.766430967 | 1.720082303 | 1.769811354 |
| hsa-miR-2681-5p | 1.739081912 | 1.810294676 | 1.691737306 | 1.694369003 | 1.653996976 | 1.718955333 |
| hsa-miR-2682-3p | 1.692976216 | 1.966482428 | 1.734779308 | 1.682417526 | 1.701670179 | 1.658566477 |
| hsa-miR-2682-5p | 1.716934257 | 1.664534758 | 1.702410249 | 1.674980711 | 1.808643237 | 1.689995705 |
| hsa-miR-26a-1-3p | 1.837190351 | 1.809168676 | 2.129593915 | 1.664465116 | 2.986657491 | 2.194829785 |
| hsa-miR-26a-2-3p | 1.831388286 | 2.098844413 | 2.327794256 | 1.796687109 | 3.456837867 | 2.088065439 |
| hsa-miR-26a-5p | 4.783689695 | 1.709214278 | 1.702592565 | 4.482645459 | 1.698720337 | 2.83206379 |
| hsa-miR-26b-3p | 1.782407214 | 1.779120892 | 1.872130395 | 1.790014563 | 2.650300705 | 1.891443192 |
| hsa-miR-26b-5p | 5.219882356 | 1.984990461 | 2.731746524 | 4.627133302 | 2.577181029 | 3.337842347 |
| hsa-miR-27a-3p | 5.972084303 | 3.043859096 | 5.065067688 | 7.552680467 | 2.886059068 | 11.14378093 |
| hsa-miR-27a-5p | 1.777201474 | 1.753286081 | 1.750145288 | 1.66571245 | 1.757026508 | 1.652365325 |
| hsa-miR-27b-3p | 3.062681185 | 1.784945207 | 1.766870007 | 6.971649978 | 1.947002446 | 10.3848401 |
| hsa-miR-27b-5p | 1.689061338 | 1.693672842 | 1.811027119 | 1.607548553 | 1.854618194 | 1.750800755 |
| hsa-miR-28-3p | 1.725965622 | 1.768992486 | 1.887613644 | 1.944529075 | 1.866390687 | 1.771920542 |
| hsa-miR-28-5p | 2.119811906 | 1.615074975 | 1.665041221 | 2.583282642 | 1.789630288 | 1.756399775 |
| hsa-miR-2861 | 9.879182953 | 9.452560241 | 5.634332051 | 4.873059351 | 1.859377721 | 4.265899176 |
| hsa-miR-2909 | 1.636857528 | 1.88064208 | 1.869360439 | 1.681806801 | 2.018437854 | 1.753190856 |
| hsa-miR-296-3p | 1.662383379 | 1.630706722 | 1.836512198 | 1.755585121 | 1.759833723 | 1.716624274 |
| hsa-miR-296-5p | 2.281416209 | 2.564240333 | 2.201249037 | 2.803630643 | 2.015574868 | 2.179476096 |
| hsa-miR-297 | 1.723131375 | 1.856595809 | 1.748138974 | 1.617276187 | 1.778486971 | 1.646550182 |
| hsa-miR-298 | 1.809862016 | 1.761194332 | 1.669623017 | 1.900514646 | 1.719406046 | 1.629528914 |
| hsa-miR-299-3p | 1.749934245 | 1.815293478 | 1.770313075 | 1.748124472 | 1.952439673 | 1.874890446 |
| hsa-miR-299-5p | 1.761552262 | 2.250164175 | 1.713028815 | 1.726987187 | 2.623621745 | 1.924502138 |
| hsa-miR-29a-3p | 9.172587026 | 3.055462357 | 3.759248881 | 8.712283336 | 5.658593322 | 10.32793131 |
| hsa-miR-29a-5p | 1.882510978 | 2.07701127 | 1.842480796 | 1.89134084 | 4.974544712 | 2.645898274 |
| hsa-miR-29b-1-5p | 2.063190293 | 1.85670284 | 1.833886021 | 1.914384435 | 2.101059496 | 2.002790322 |
| hsa-miR-29b-2-5p | 1.716987979 | 1.716906103 | 1.738053183 | 1.660341622 | 2.198349763 | 1.809335772 |
| hsa-miR-29b-3p | 5.667269201 | 1.876210597 | 2.77192366 | 4.209595993 | 2.310570658 | 5.48998278 |
| hsa-miR-29c-3p | 6.053975381 | 1.895553335 | 2.652474097 | 7.743111576 | 3.007891189 | 10.31517934 |
| hsa-miR-29c-5p | 1.870162369 | 1.702099483 | 1.837463553 | 3.781673638 | 1.941577245 | 2.22322194 |
| hsa-miR-300 | 1.784576501 | 1.843366131 | 1.810655359 | 1.625810584 | 1.699584247 | 1.757429272 |
| hsa-miR-301a-3p | 3.007464979 | 1.590495554 | 1.772989753 | 3.024526748 | 2.280130321 | 2.281209145 |
| hsa-miR-301a-5p | 1.78387003 | 1.990468893 | 1.786861709 | 1.745706577 | 2.201630332 | 1.987168943 |
| hsa-miR-301b-3p | 1.741585844 | 1.723670584 | 1.780105141 | 1.712695878 | 1.885182798 | 1.743589373 |
| hsa-miR-301b-5p | 1.668738126 | 1.786789322 | 1.825948912 | 1.688299727 | 1.71494475 | 1.827024542 |
| hsa-miR-302a-3p | 1.80987111 | 1.934348717 | 2.13570525 | 1.880845805 | 2.354416959 | 1.783389915 |
| hsa-miR-302a-5p | 1.722462411 | 1.705553283 | 2.049799997 | 1.648619449 | 2.184788105 | 2.141676994 |
| hsa-miR-302b-3p | 1.878082329 | 1.851907088 | 1.758306638 | 1.735391098 | 2.118750356 | 1.966691861 |
| hsa-miR-302b-5p | 1.94866566 | 1.75054828 | 1.734372966 | 1.736784305 | 1.700804658 | 1.792538876 |
| hsa-miR-302c-3p | 1.647720746 | 1.732537389 | 1.791122016 | 1.840433363 | 2.380076518 | 1.739668738 |
| hsa-miR-302c-5p | 1.805080153 | 1.719602693 | 1.694103944 | 1.718914235 | 1.643820697 | 1.725679874 |
| hsa-miR-302d-3p | 1.716839908 | 1.794411742 | 1.752921458 | 1.774322394 | 1.983616736 | 1.812522638 |
| hsa-miR-302d-5p | 1.669806071 | 1.712021252 | 1.789705183 | 1.60487229 | 1.936944406 | 1.731325337 |
| hsa-miR-302e | 1.748922597 | 1.683998618 | 1.623767632 | 1.675896278 | 1.815301221 | 1.681611984 |
| hsa-miR-302f | 1.761113536 | 1.644584994 | 1.717932733 | 1.735281959 | 2.065996878 | 1.761316026 |
| hsa-miR-3064-3p | 1.70260103 | 1.692460312 | 1.705216804 | 1.617193056 | 2.321430197 | 1.738391865 |
| hsa-miR-3064-5p | 1.726315732 | 1.916732011 | 1.851422591 | 1.674422252 | 1.842058413 | 1.750586856 |
| hsa-miR-3065-3p | 1.72475697 | 1.767334399 | 1.671533673 | 1.967297783 | 1.819237337 | 1.743380676 |
| hsa-miR-3065-5p | 1.82763054 | 1.759069192 | 1.839023348 | 1.735597939 | 1.735342069 | 1.725828613 |
| hsa-miR-3074-3p | 1.665141129 | 1.812803546 | 1.752686762 | 1.77582401 | 1.726473762 | 1.697873997 |
| hsa-miR-3074-5p | 1.714362823 | 1.936346975 | 1.787840676 | 1.636315203 | 1.795047786 | 1.701157758 |
| hsa-miR-30a-3p | 3.287435154 | 1.685350708 | 1.813278683 | 2.497362368 | 1.767860772 | 1.827535931 |
| hsa-miR-30a-5p | 8.436607255 | 1.71478538 | 1.805442325 | 6.708446533 | 1.849986762 | 7.670160384 |
| hsa-miR-30b-3p | 2.269644983 | 1.747763023 | 1.673175674 | 2.486022559 | 2.304486285 | 1.677609965 |
| hsa-miR-30b-5p | 4.058589013 | 1.683395662 | 2.622838919 | 5.703327503 | 1.565760114 | 2.631656555 |
| hsa-miR-30c-1-3p | 1.769775785 | 1.770829758 | 1.875168427 | 3.887639007 | 1.9611292 | 1.888912843 |
| hsa-miR-30c-2-3p | 3.403001515 | 2.459812885 | 1.977321728 | 4.619434753 | 2.594603399 | 2.929726699 |
| hsa-miR-30c-5p | 6.215163658 | 5.329001222 | 6.102324917 | 4.479104363 | 1.698950382 | 4.665027712 |
| hsa-miR-30d-3p | 1.758369681 | 1.694809893 | 1.738318341 | 1.818887861 | 1.984300215 | 1.676512817 |
| hsa-miR-30d-5p | 7.35307243 | 6.776258787 | 7.488078814 | 9.196383423 | 7.640105283 | 10.22959971 |
| hsa-miR-30e-3p | 2.280700036 | 2.14744317 | 2.00631877 | 2.315041486 | 1.795821288 | 1.790678362 |
| hsa-miR-30e-5p | 6.377235909 | 8.515341671 | 9.86902651 | 6.407837492 | 1.984094929 | 9.745657058 |
| hsa-miR-31-3p | 1.832296123 | 1.706114825 | 1.70635775 | 1.905904077 | 1.932849208 | 1.833243776 |
| hsa-miR-31-5p | 2.199284726 | 1.800735901 | 1.768283226 | 1.828542719 | 1.920333354 | 1.763836282 |
| hsa-miR-3115 | 1.686809572 | 1.847241421 | 1.676311775 | 1.756989517 | 2.839142607 | 1.753914615 |
| hsa-miR-3116 | 1.74170083 | 1.739184719 | 1.715461992 | 1.721204779 | 1.990889367 | 1.801780224 |
| hsa-miR-3117-3p | 1.675611587 | 1.701570386 | 1.812596027 | 1.679551812 | 1.755675517 | 1.708648894 |
| hsa-miR-3117-5p | 1.728474526 | 1.833720227 | 2.084445923 | 1.677757052 | 2.103684875 | 1.988643377 |
| hsa-miR-3118 | 1.753053293 | 1.94733274 | 2.139733943 | 1.701585288 | 2.139610068 | 1.823913447 |
| hsa-miR-3119 | 1.75557979 | 1.78396624 | 1.892888873 | 1.742453975 | 2.355106253 | 1.780278252 |
| hsa-miR-3120-3p | 1.680164925 | 1.786577633 | 1.719296854 | 1.720367983 | 1.711900939 | 1.749698599 |
| hsa-miR-3120-5p | 1.702636896 | 1.801817212 | 1.783927165 | 1.73246462 | 1.850077464 | 1.727935489 |
| hsa-miR-3121-3p | 1.801118414 | 1.954527755 | 1.748399389 | 1.792338879 | 1.675909144 | 1.713330114 |
| hsa-miR-3121-5p | 1.927819393 | 2.110029762 | 1.983041409 | 1.743338422 | 2.329150455 | 2.156439503 |
| hsa-miR-3122 | 1.884971514 | 1.717245584 | 1.694154451 | 1.795913188 | 1.815456668 | 1.759747871 |
| hsa-miR-3123 | 1.709059955 | 1.82660964 | 1.768800893 | 1.644681614 | 1.856275694 | 1.77019427 |
| hsa-miR-3124-3p | 1.61834376 | 1.683418348 | 1.675577968 | 1.608610295 | 1.686025705 | 1.752590443 |
| hsa-miR-3124-5p | 3.33636808 | 4.176123662 | 4.033452871 | 2.560701733 | 2.029271037 | 4.176479702 |
| hsa-miR-3125 | 1.846649854 | 1.765114266 | 1.779708261 | 2.99409895 | 1.820622024 | 1.819674318 |
| hsa-miR-3126-3p | 1.629322274 | 1.766483864 | 1.727422542 | 1.661575599 | 1.843437262 | 1.821603055 |
| hsa-miR-3126-5p | 1.745006161 | 1.782747168 | 1.725037405 | 1.683335321 | 1.812421306 | 1.920213254 |
| hsa-miR-3127-3p | 1.658904703 | 1.821742837 | 1.718733035 | 1.674705813 | 1.908519841 | 1.729430507 |
| hsa-miR-3127-5p | 5.602830246 | 2.190782638 | 1.741177896 | 4.034744466 | 1.886166542 | 1.733264905 |
| hsa-miR-3128 | 1.783097646 | 1.840125203 | 1.728671223 | 1.727404192 | 2.238515879 | 1.788875483 |
| hsa-miR-3129-3p | 1.839597798 | 1.799653936 | 1.729265281 | 1.665495912 | 1.782414502 | 1.753602414 |
| hsa-miR-3129-5p | 1.759936356 | 2.174544451 | 1.768030137 | 1.769791254 | 2.157299926 | 1.931099704 |
| hsa-miR-3130-3p | 1.658644824 | 1.778460053 | 1.719449352 | 1.743055424 | 1.739686094 | 1.722040166 |
| hsa-miR-3130-5p | 1.768512747 | 1.698534406 | 1.627638207 | 1.792849171 | 1.7089774 | 1.640329673 |
| hsa-miR-3131 | 2.216684891 | 1.960526336 | 2.027895117 | 2.118313003 | 1.68916306 | 1.99938061 |
| hsa-miR-3132 | 1.820954478 | 1.689344255 | 1.737979852 | 1.968727304 | 2.05040134 | 1.936087318 |
| hsa-miR-3133 | 1.764350626 | 1.748830675 | 1.943396134 | 1.67889023 | 1.903308214 | 1.814246498 |
| hsa-miR-3134 | 1.694373979 | 1.800475976 | 2.239943263 | 1.747319645 | 2.82384844 | 1.880271873 |
| hsa-miR-3135a | 1.631453983 | 1.699941142 | 1.651247443 | 1.682055157 | 1.763099065 | 1.714276661 |
| hsa-miR-3135b | 8.655369556 | 10.89845251 | 10.59655008 | 4.856402589 | 1.688349794 | 5.168882296 |
| hsa-miR-3136-3p | 1.813561331 | 1.802486022 | 2.186774249 | 1.718759298 | 2.76660601 | 2.390601901 |
| hsa-miR-3136-5p | 1.846262356 | 1.735284657 | 2.000013433 | 3.883836805 | 1.79784559 | 1.700163322 |
| hsa-miR-3137 | 2.881127589 | 2.488821195 | 2.477832803 | 3.260642606 | 1.894131998 | 2.225189863 |
| hsa-miR-3138 | 5.589205421 | 3.249885022 | 6.164024673 | 7.719357733 | 1.951155128 | 5.817745693 |
| hsa-miR-3139 | 1.703741062 | 1.878260708 | 1.949387289 | 1.771522705 | 1.905176878 | 1.749591637 |
| hsa-miR-3140-3p | 1.802719026 | 1.756394879 | 1.718155833 | 1.599424317 | 2.053219048 | 1.718529093 |
| hsa-miR-3140-5p | 1.84839631 | 1.66421118 | 1.787662664 | 1.70576067 | 1.778162957 | 1.698120797 |
| hsa-miR-3141 | 7.392363437 | 7.933688684 | 8.631766067 | 9.937102836 | 2.129403187 | 8.247343492 |
| hsa-miR-3142 | 1.689179889 | 1.692259031 | 1.903024595 | 1.659443245 | 2.478232503 | 1.860066089 |
| hsa-miR-3143 | 1.668385172 | 2.152172524 | 1.6737845 | 1.733852405 | 1.996735263 | 1.670637686 |
| hsa-miR-3144-3p | 1.733174537 | 1.633512439 | 1.750552222 | 1.69152628 | 1.964233934 | 1.708351137 |
| hsa-miR-3144-5p | 1.795072039 | 1.718753853 | 1.658374071 | 1.731453321 | 1.892916407 | 1.650853016 |
| hsa-miR-3145-3p | 1.739218326 | 1.832671401 | 1.807433674 | 1.724547438 | 2.43211555 | 1.797826664 |
| hsa-miR-3145-5p | 1.767135108 | 2.032960637 | 1.748242394 | 1.700100885 | 1.784908569 | 1.876478783 |
| hsa-miR-3146 | 1.76472287 | 1.740435225 | 1.734659497 | 1.670491284 | 1.781812758 | 1.790224142 |
| hsa-miR-3147 | 3.390438675 | 2.134406084 | 2.181742817 | 5.230455041 | 1.633805122 | 2.089599856 |
| hsa-miR-3148 | 1.757807539 | 2.521443408 | 1.867375732 | 1.720842781 | 2.227300473 | 1.742368227 |
| hsa-miR-3149 | 2.111067788 | 1.850578218 | 2.976327296 | 1.80678484 | 2.276548797 | 2.468119625 |
| hsa-miR-3150a-3p | 1.726662307 | 1.832103999 | 1.830737415 | 1.765913108 | 1.915955823 | 1.956214765 |
| hsa-miR-3150a-5p | 1.614882053 | 1.860344131 | 1.83097577 | 1.624317436 | 1.759361799 | 1.876961242 |
| hsa-miR-3150b-3p | 1.720253472 | 1.729699288 | 1.698687783 | 1.783994736 | 1.790685024 | 1.65860501 |
| hsa-miR-3150b-5p | 2.040236067 | 1.826447742 | 1.976623366 | 1.933215314 | 1.704419589 | 1.649703511 |
| hsa-miR-3151-3p | 3.028524152 | 3.787318134 | 3.607045414 | 2.584730353 | 3.308466292 | 2.649500638 |
| hsa-miR-3151-5p | 1.762503629 | 1.680796743 | 1.720286991 | 1.802473283 | 1.740281235 | 1.708186422 |
| hsa-miR-3152-3p | 3.570327145 | 2.900994069 | 2.583472571 | 2.230880656 | 1.997742296 | 2.028431373 |
| hsa-miR-3152-5p | 1.69909155 | 1.730105035 | 1.756538796 | 1.667563301 | 1.772160004 | 1.833792283 |
| hsa-miR-3153 | 1.710837175 | 1.691759056 | 1.711001026 | 1.794688557 | 1.705166176 | 1.738323302 |
| hsa-miR-3154 | 1.886013329 | 1.75785706 | 2.093143565 | 2.92950104 | 1.641200717 | 2.138432642 |
| hsa-miR-3155a | 1.664839517 | 2.057029368 | 1.861838839 | 1.666156814 | 2.089571765 | 1.817550724 |
| hsa-miR-3155b | 3.150028033 | 1.931559281 | 2.009568325 | 2.041765341 | 1.817657076 | 2.43145732 |
| hsa-miR-3156-3p | 1.684736924 | 1.807180806 | 2.087318532 | 1.634284823 | 2.297724752 | 2.04733382 |
| hsa-miR-3156-5p | 5.524882875 | 5.553682582 | 5.054515276 | 6.301543198 | 1.704534862 | 9.602500745 |
| hsa-miR-3157-3p | 1.662702891 | 1.664006797 | 1.940326568 | 1.612514978 | 2.18401057 | 2.097806415 |
| hsa-miR-3157-5p | 1.623626303 | 2.147405118 | 1.906994672 | 1.669949106 | 1.715189796 | 1.723951459 |
| hsa-miR-3158-3p | 1.789526873 | 1.716098569 | 1.718167302 | 1.6650248 | 1.679572476 | 1.735792107 |
| hsa-miR-3158-5p | 1.895314085 | 1.863132292 | 1.997359149 | 2.268796689 | 1.898510925 | 1.756235686 |
| hsa-miR-3159 | 1.660784785 | 1.660040666 | 1.680229816 | 1.668111486 | 1.874781401 | 1.724382603 |
| hsa-miR-3160-3p | 1.676758804 | 1.912912264 | 1.786607301 | 1.672606416 | 1.69741599 | 1.781403588 |
| hsa-miR-3160-5p | 1.634749467 | 1.762071426 | 1.822718914 | 1.734538427 | 1.79295826 | 1.749284755 |
| hsa-miR-3161 | 1.659833052 | 1.786396352 | 1.65376266 | 1.85114224 | 1.781003914 | 1.673435926 |
| hsa-miR-3162-3p | 6.386257945 | 8.44444476 | 8.49321097 | 4.137455996 | 11.06497157 | 7.92441891 |
| hsa-miR-3162-5p | 8.613454184 | 9.571746133 | 9.969176128 | 7.394458441 | 7.456146122 | 8.738535631 |
| hsa-miR-3163 | 1.679456425 | 1.74374215 | 1.754458968 | 2.273407174 | 1.881511494 | 1.730895023 |
| hsa-miR-3164 | 1.695072479 | 1.910516546 | 1.846116106 | 1.758755413 | 2.158566585 | 1.849880212 |
| hsa-miR-3165 | 1.72069406 | 1.843453963 | 1.819301919 | 1.677397577 | 1.930140652 | 1.723282541 |
| hsa-miR-3166 | 1.652285558 | 2.099391236 | 1.70569738 | 1.60488239 | 1.71296957 | 1.738069363 |
| hsa-miR-3167 | 1.812000977 | 1.843691346 | 1.741506722 | 1.666861935 | 1.687891731 | 1.720191238 |
| hsa-miR-3168 | 1.710167007 | 1.612759647 | 1.783255113 | 1.64911488 | 1.745542371 | 1.673042952 |
| hsa-miR-3169 | 1.670515532 | 1.758953682 | 1.641009895 | 1.64334968 | 1.634130927 | 1.652519901 |
| hsa-miR-3170 | 1.728056998 | 1.795991985 | 1.730007305 | 1.729317368 | 1.637773556 | 1.685438976 |
| hsa-miR-3171 | 1.940465783 | 1.82498475 | 2.140121989 | 1.752987039 | 2.178610566 | 1.856727192 |
| hsa-miR-3173-3p | 1.661671428 | 1.745945936 | 1.719950501 | 1.74951363 | 1.836187528 | 1.733834641 |
| hsa-miR-3173-5p | 1.810338597 | 1.780010812 | 1.789848699 | 1.763896874 | 1.695074706 | 1.709618354 |
| hsa-miR-3174 | 1.745164244 | 1.863195192 | 1.825949193 | 2.001796727 | 1.977099898 | 1.703679191 |
| hsa-miR-3175 | 1.561821594 | 1.776109174 | 1.757578074 | 1.598841897 | 1.887475935 | 1.756616508 |
| hsa-miR-3176 | 1.623832764 | 1.792056581 | 1.815568058 | 1.661158575 | 2.283375942 | 1.733409182 |
| hsa-miR-3177-3p | 1.737030194 | 1.77215399 | 1.817731548 | 2.672903877 | 1.763209921 | 1.692561097 |
| hsa-miR-3177-5p | 1.626298107 | 1.699640808 | 1.73758175 | 1.677278924 | 1.656369162 | 1.791389218 |
| hsa-miR-3178 | 1.678569947 | 1.733345497 | 1.648727796 | 1.584743598 | 1.813194508 | 1.685420021 |
| hsa-miR-3179 | 1.85213343 | 1.688243312 | 1.773113103 | 1.682743174 | 1.708955416 | 1.6720135 |
| hsa-miR-3180 | 1.654216266 | 1.747557572 | 1.678840902 | 1.636130786 | 1.724954134 | 1.709760951 |
| hsa-miR-3180-3p | 1.842898986 | 1.680406529 | 1.792386092 | 3.456537547 | 1.699508244 | 5.074600061 |
| hsa-miR-3180-5p | 3.330698833 | 2.054941435 | 4.145987957 | 2.921612384 | 3.594932704 | 2.780660107 |
| hsa-miR-3181 | 1.760682136 | 1.677593443 | 1.730473756 | 2.410277902 | 1.697495443 | 1.635031613 |
| hsa-miR-3182 | 1.765653241 | 1.780615136 | 1.918212621 | 1.728433671 | 2.016955144 | 1.794643791 |
| hsa-miR-3183 | 1.733522503 | 1.67354643 | 1.758947689 | 1.749240683 | 1.661174931 | 1.664804133 |
| hsa-miR-3184-3p | 1.7409472 | 1.925558948 | 2.612321972 | 1.745332964 | 1.857948329 | 1.918792406 |
| hsa-miR-3184-5p | 1.710030182 | 1.950857988 | 1.817333053 | 1.684777687 | 1.986357403 | 1.749088764 |
| hsa-miR-3185 | 1.782815259 | 1.847496129 | 1.751336983 | 1.701504714 | 1.776338228 | 1.697558085 |
| hsa-miR-3186-3p | 2.731133339 | 1.899480749 | 1.867282439 | 3.178529071 | 2.073694348 | 2.14856961 |
| hsa-miR-3186-5p | 1.752249403 | 1.864685654 | 1.843596228 | 1.71787511 | 1.645262743 | 1.75173047 |
| hsa-miR-3187-3p | 1.847170949 | 1.688010657 | 1.694166664 | 1.891571022 | 1.695838574 | 1.630200372 |
| hsa-miR-3187-5p | 1.696903036 | 1.692501695 | 1.683780543 | 1.754862127 | 1.74867169 | 1.718880787 |
| hsa-miR-3188 | 3.001881535 | 2.590930281 | 1.948064259 | 4.304573082 | 1.734263093 | 4.048461095 |
| hsa-miR-3189-3p | 1.928288792 | 1.720998279 | 2.005460923 | 2.785550069 | 1.733435281 | 1.900275167 |
| hsa-miR-3189-5p | 1.975832703 | 1.840375168 | 1.804992303 | 1.92704823 | 1.730414822 | 1.894501063 |
| hsa-miR-3190-3p | 1.824289326 | 2.056005885 | 1.770522348 | 2.962423502 | 1.680995401 | 1.712244844 |
| hsa-miR-3190-5p | 1.756310988 | 1.72182611 | 1.840020806 | 1.793873949 | 2.006523443 | 1.798097948 |
| hsa-miR-3191-3p | 1.726603156 | 1.683426468 | 1.632581896 | 1.638295452 | 1.635445285 | 1.606594074 |
| hsa-miR-3191-5p | 1.76270632 | 2.264182718 | 1.809641568 | 1.709689122 | 1.867764146 | 1.731337005 |
| hsa-miR-3192-3p | 1.705997725 | 1.964324907 | 1.726293194 | 1.736287465 | 1.69942436 | 1.715989987 |
| hsa-miR-3192-5p | 1.926373834 | 1.7776946 | 1.81338464 | 1.889664411 | 2.083561037 | 1.726571949 |
| hsa-miR-3193 | 1.835437828 | 1.707763207 | 1.687317344 | 1.731982423 | 1.880564596 | 1.734366648 |
| hsa-miR-3194-3p | 1.703637525 | 1.903909422 | 1.792212637 | 1.731064029 | 1.894723453 | 1.823966393 |
| hsa-miR-3194-5p | 1.983936982 | 2.421539987 | 1.720125665 | 2.609130023 | 1.774970392 | 1.843024783 |
| hsa-miR-3195 | 5.11796856 | 4.727694816 | 1.990389071 | 8.01962254 | 1.781358887 | 2.882423147 |
| hsa-miR-3196 | 4.095057734 | 1.992651527 | 1.73903403 | 4.090678478 | 1.796825707 | 1.954130285 |
| hsa-miR-3197 | 1.67269795 | 1.754778073 | 1.776972456 | 1.713547115 | 1.764353361 | 1.75047604 |
| hsa-miR-3198 | 2.604818523 | 1.868998707 | 1.787138907 | 2.818330535 | 1.690866678 | 1.985626795 |
| hsa-miR-3199 | 1.67054302 | 1.852687557 | 1.857133352 | 1.650270594 | 1.878512904 | 1.770196516 |
| hsa-miR-32-3p | 1.874097484 | 2.159813603 | 3.458620726 | 1.926440667 | 3.402498845 | 1.982525967 |
| hsa-miR-32-5p | 1.95896149 | 1.724303359 | 1.890937042 | 1.635240569 | 1.987919428 | 1.705766889 |
| hsa-miR-3200-3p | 1.739568056 | 1.678867942 | 1.733558729 | 1.677922437 | 1.814574995 | 1.769185899 |
| hsa-miR-3200-5p | 1.86648126 | 1.697070704 | 1.815244751 | 1.952825916 | 1.627829707 | 1.702045871 |
| hsa-miR-3201 | 1.724051496 | 1.666937584 | 1.600898611 | 1.653130655 | 1.650156433 | 1.688740769 |
| hsa-miR-3202 | 5.302677605 | 3.735900302 | 4.944304209 | 7.195225597 | 1.999590751 | 2.967258855 |
| hsa-miR-320a | 5.917201301 | 3.403274114 | 4.167674517 | 5.913637166 | 1.8313408 | 6.676197852 |
| hsa-miR-320b | 6.751775247 | 5.121024805 | 5.051944805 | 6.169924539 | 1.843824632 | 6.933820224 |
| hsa-miR-320c | 7.371214072 | 6.115266821 | 5.665920699 | 6.904714869 | 1.72827147 | 6.328120147 |
| hsa-miR-320d | 8.333340152 | 8.557194897 | 9.220845884 | 6.850124931 | 2.18989662 | 9.170260072 |
| hsa-miR-320e | 7.334517238 | 6.675545689 | 6.635460368 | 6.340761772 | 1.750573295 | 7.672714582 |
| hsa-miR-323a-3p | 1.771843895 | 1.790760041 | 1.950704826 | 1.864827122 | 2.019045489 | 2.643857939 |
| hsa-miR-323a-5p | 1.608335524 | 1.708115986 | 1.723076819 | 1.669434883 | 1.665182646 | 1.680734789 |
| hsa-miR-323b-3p | 1.61953118 | 1.784194513 | 1.707194972 | 1.693479851 | 1.944328918 | 1.682934861 |
| hsa-miR-323b-5p | 1.712646953 | 1.834978964 | 1.678180912 | 1.633318799 | 1.911631132 | 1.784107908 |
| hsa-miR-324-3p | 6.785115244 | 3.205664477 | 4.266142052 | 5.187174382 | 2.003740735 | 6.174652082 |
| hsa-miR-324-5p | 5.037661939 | 1.718330485 | 1.648800959 | 3.97175326 | 1.71741979 | 2.274545733 |
| hsa-miR-325 | 1.721928235 | 1.737997017 | 1.748168722 | 1.710159718 | 1.738072364 | 1.711379252 |
| hsa-miR-326 | 1.867779283 | 1.805077064 | 1.703851768 | 2.858707518 | 1.855584307 | 1.910323715 |
| hsa-miR-328-3p | 2.884816514 | 3.152770849 | 3.582783147 | 7.39296136 | 2.381841434 | 8.849160906 |
| hsa-miR-328-5p | 6.917614234 | 3.755698076 | 2.029627244 | 5.122970641 | 2.167905701 | 1.73746266 |
| hsa-miR-329-3p | 1.708028251 | 1.943186708 | 2.112285086 | 1.624399339 | 1.867502773 | 2.220661264 |
| hsa-miR-329-5p | 2.017164473 | 1.957408226 | 1.827508526 | 1.778694534 | 1.891864128 | 1.720133591 |
| hsa-miR-330-3p | 2.028949025 | 1.88461304 | 1.765722842 | 2.716925537 | 1.745372989 | 1.937532624 |
| hsa-miR-330-5p | 1.651217774 | 1.7010765 | 2.19294292 | 1.679072783 | 1.940707734 | 1.684072194 |
| hsa-miR-331-3p | 7.207081971 | 1.876382875 | 1.751429811 | 5.205816059 | 1.970154197 | 2.781892473 |
| hsa-miR-331-5p | 1.61768177 | 1.860486002 | 1.78063055 | 1.616946578 | 2.315933012 | 1.804702708 |
| hsa-miR-335-3p | 1.806861717 | 2.052708034 | 1.914679818 | 1.812117234 | 2.490634171 | 2.019189577 |
| hsa-miR-335-5p | 1.647743689 | 1.982603625 | 1.958588989 | 1.665203678 | 1.934249718 | 1.819944215 |
| hsa-miR-337-3p | 1.831306518 | 2.16907382 | 2.058097903 | 1.864794095 | 2.72508516 | 2.018536398 |
| hsa-miR-337-5p | 1.637190251 | 1.945135642 | 1.687216323 | 1.744537092 | 1.754298429 | 2.174105085 |
| hsa-miR-338-3p | 1.840209176 | 1.854314277 | 2.206978534 | 1.696330936 | 3.115741906 | 2.352598374 |
| hsa-miR-338-5p | 1.753289485 | 1.75792266 | 1.851765178 | 1.941125154 | 1.942922797 | 1.740551248 |
| hsa-miR-339-3p | 3.286879613 | 1.70339782 | 1.719449992 | 2.650894952 | 1.700531291 | 1.703146693 |
| hsa-miR-339-5p | 1.722047312 | 1.742051513 | 1.72819119 | 1.76106401 | 1.806417717 | 1.634499327 |
| hsa-miR-33a-3p | 1.700212176 | 1.813445183 | 1.753748061 | 1.645003398 | 1.989204683 | 1.969245842 |
| hsa-miR-33a-5p | 1.929568365 | 1.933422134 | 2.009369174 | 1.743800088 | 2.329176496 | 1.811548069 |
| hsa-miR-33b-3p | 2.096352391 | 2.143569631 | 2.324272357 | 2.212672046 | 2.274397029 | 2.30896115 |
| hsa-miR-33b-5p | 1.722030043 | 1.801138547 | 1.720103303 | 1.631152057 | 1.89061175 | 1.840921232 |
| hsa-miR-340-3p | 1.895268498 | 1.667326011 | 1.718070128 | 1.700987666 | 1.904936967 | 1.799765652 |
| hsa-miR-340-5p | 1.958968432 | 1.732129149 | 1.715833208 | 1.829418501 | 1.919835277 | 1.956767069 |
| hsa-miR-342-3p | 5.682728535 | 6.517785187 | 6.862266127 | 2.0452573 | 1.946055738 | 3.890353952 |
| hsa-miR-342-5p | 1.884506737 | 2.213932788 | 2.194902274 | 1.662973877 | 2.117371955 | 1.879843931 |
| hsa-miR-345-3p | 3.950950708 | 2.387637678 | 2.887262313 | 4.432805129 | 1.82720639 | 2.921809318 |
| hsa-miR-345-5p | 2.087651543 | 1.716034139 | 1.864672068 | 2.051279496 | 1.804862623 | 1.745238921 |
| hsa-miR-346 | 1.759518012 | 2.029898753 | 1.688922291 | 1.724927323 | 1.641196363 | 1.622185555 |
| hsa-miR-34a-3p | 1.744428438 | 1.735022905 | 1.631768394 | 2.251117868 | 1.810670586 | 1.653554793 |
| hsa-miR-34a-5p | 2.581060699 | 1.721929728 | 1.825174662 | 8.897247424 | 2.116640871 | 10.82385506 |
| hsa-miR-34b-3p | 1.756578615 | 1.742632764 | 1.660630915 | 6.866913349 | 2.379530551 | 5.963013464 |
| hsa-miR-34b-5p | 1.815508669 | 1.715173386 | 1.831912209 | 6.725443422 | 2.574234681 | 8.121017371 |
| hsa-miR-34c-3p | 1.758906986 | 2.060096396 | 1.940380724 | 1.982978441 | 1.809632416 | 1.772984256 |
| hsa-miR-34c-5p | 1.695399285 | 1.756336187 | 1.867101804 | 6.858289591 | 2.49948546 | 7.708529657 |
| hsa-miR-3529-3p | 1.698916715 | 1.925644952 | 2.053564087 | 1.728501773 | 2.739731328 | 1.796914915 |
| hsa-miR-3529-5p | 1.720373466 | 1.902739035 | 2.062600197 | 1.716110588 | 2.840716131 | 2.150875878 |
| hsa-miR-3591-3p | 1.680261106 | 1.82713817 | 1.744729254 | 1.668168444 | 1.91756263 | 2.066748551 |
| hsa-miR-3591-5p | 1.707314386 | 1.831955023 | 1.725733458 | 1.624992326 | 1.940421037 | 1.77964598 |
| hsa-miR-3605-3p | 1.673092844 | 1.924470226 | 2.379541681 | 1.686038919 | 2.217186369 | 2.028106528 |
| hsa-miR-3605-5p | 1.838358038 | 2.469174495 | 2.085365155 | 4.068907679 | 1.785820626 | 1.888025159 |
| hsa-miR-3606-3p | 1.870478896 | 1.820374353 | 2.05163563 | 1.797501118 | 4.027700614 | 2.018379095 |
| hsa-miR-3606-5p | 1.888431693 | 2.045956498 | 2.632873284 | 1.749350637 | 2.443425207 | 2.02275224 |
| hsa-miR-3607-3p | 1.636924556 | 1.790804914 | 1.687750094 | 1.680196756 | 1.740859594 | 1.682449195 |
| hsa-miR-3607-5p | 1.75075698 | 1.996266864 | 1.829928169 | 1.653462931 | 1.975721284 | 1.734832893 |
| hsa-miR-3609 | 1.962962864 | 1.685194345 | 1.684599708 | 1.685197699 | 1.776873474 | 1.669738633 |
| hsa-miR-361-3p | 2.130181604 | 1.942894548 | 2.047429808 | 2.409760534 | 2.060210845 | 2.515291624 |
| hsa-miR-361-5p | 5.337644806 | 3.987141778 | 4.800512377 | 5.474267063 | 1.807038368 | 5.632526043 |
| hsa-miR-3610 | 2.059907844 | 1.788517552 | 1.688340441 | 3.678958753 | 1.71896211 | 1.665971645 |
| hsa-miR-3611 | 1.748394361 | 1.882039918 | 1.950042274 | 1.70772748 | 1.932911795 | 2.060136955 |
| hsa-miR-3612 | 1.694532099 | 1.752763363 | 1.675521388 | 1.678075653 | 1.662426486 | 1.724057981 |
| hsa-miR-3613-3p | 1.991663083 | 1.993069889 | 1.81939155 | 1.913394987 | 2.00019603 | 2.124401306 |
| hsa-miR-3613-5p | 1.922084923 | 2.151150469 | 1.905916873 | 1.909125331 | 1.97598765 | 1.937788361 |
| hsa-miR-3614-3p | 1.6430797 | 2.027842171 | 1.976271623 | 1.69436691 | 2.650098756 | 1.909553706 |
| hsa-miR-3614-5p | 1.726491403 | 2.077022373 | 1.937723344 | 1.766137993 | 1.766222912 | 1.723189027 |
| hsa-miR-3615 | 1.736413255 | 1.814678795 | 1.698962485 | 1.653715933 | 1.826514944 | 1.817677363 |
| hsa-miR-3616-3p | 1.738409451 | 2.182689547 | 1.9076025 | 1.704134348 | 1.942634081 | 1.783161622 |
| hsa-miR-3616-5p | 1.663947396 | 1.715090304 | 1.839333619 | 1.741105831 | 2.07736345 | 1.936367628 |
| hsa-miR-3617-3p | 1.818111388 | 1.791691709 | 1.787413496 | 1.826452668 | 1.756648738 | 1.87881295 |
| hsa-miR-3617-5p | 1.983111345 | 3.880181737 | 2.335526503 | 4.513121801 | 1.848903077 | 6.022995293 |
| hsa-miR-3618 | 1.814073976 | 1.697088673 | 1.732502856 | 1.70589607 | 1.970269192 | 1.68308254 |
| hsa-miR-3619-3p | 1.720998167 | 1.867128699 | 1.819729028 | 1.633322886 | 1.856556464 | 1.752898747 |
| hsa-miR-3619-5p | 1.714127629 | 2.059802717 | 1.775184823 | 1.659862652 | 1.890960881 | 1.719390649 |
| hsa-miR-362-3p | 1.723790333 | 1.665581445 | 1.683566824 | 2.046502791 | 1.81400903 | 2.31865118 |
| hsa-miR-362-5p | 1.77833508 | 1.69338172 | 1.694137036 | 1.66574684 | 1.927956426 | 1.745576789 |
| hsa-miR-3620-3p | 2.81412636 | 2.966127878 | 2.642105148 | 2.372086868 | 2.588742609 | 1.958674281 |
| hsa-miR-3620-5p | 1.686580863 | 1.789713728 | 1.887220774 | 2.181480667 | 1.747754621 | 1.701220015 |
| hsa-miR-3621 | 1.784882335 | 1.816441462 | 1.746186681 | 2.955270503 | 1.775468116 | 1.820715667 |
| hsa-miR-3622a-3p | 1.700885363 | 1.855163139 | 1.774731837 | 1.760231759 | 1.891061075 | 1.782572327 |
| hsa-miR-3622a-5p | 1.725462526 | 1.634476273 | 1.690160772 | 1.84287891 | 1.76481667 | 1.750685375 |
| hsa-miR-3622b-3p | 1.682146466 | 1.751744757 | 1.788958465 | 1.722244382 | 1.737289257 | 1.69232388 |
| hsa-miR-3622b-5p | 1.841568154 | 1.856550522 | 1.717581362 | 2.812314173 | 1.833037766 | 1.9562049 |
| hsa-miR-363-3p | 1.856157699 | 1.841542668 | 1.801778551 | 5.471571103 | 1.864931219 | 5.959541781 |
| hsa-miR-363-5p | 1.636962149 | 1.794519729 | 1.688230119 | 1.672671782 | 1.747585308 | 1.676471821 |
| hsa-miR-3646 | 2.927442323 | 4.000470368 | 2.778658045 | 4.631886716 | 1.739535308 | 2.866322841 |
| hsa-miR-3648 | 2.252748346 | 2.279197362 | 1.935761534 | 4.426819993 | 1.703898645 | 1.960452723 |
| hsa-miR-3649 | 1.723232597 | 1.622646176 | 1.639658984 | 1.797095965 | 1.731692521 | 1.691890842 |
| hsa-miR-3650 | 1.675087419 | 1.85147523 | 1.822608433 | 1.686840433 | 1.715886553 | 1.778995986 |
| hsa-miR-3651 | 7.753694611 | 2.571391624 | 1.915013407 | 1.713447857 | 1.858338358 | 1.876570452 |
| hsa-miR-3652 | 5.631244196 | 4.689581702 | 3.17101386 | 4.138180633 | 1.834567511 | 6.111600701 |
| hsa-miR-3653-3p | 1.687162242 | 1.742050022 | 1.983228958 | 2.773143382 | 2.898205665 | 1.749948798 |
| hsa-miR-3653-5p | 1.921534182 | 1.85251873 | 1.75121685 | 1.742535456 | 1.941407528 | 1.809747913 |
| hsa-miR-3654 | 1.756765653 | 1.723241955 | 1.800426913 | 1.76891405 | 1.734456508 | 1.758846924 |
| hsa-miR-3655 | 1.74726581 | 1.697376732 | 1.756487854 | 1.681000049 | 2.072174586 | 1.719286742 |
| hsa-miR-3656 | 7.2709709 | 5.039161843 | 6.527325797 | 7.610371348 | 2.225535664 | 4.569667915 |
| hsa-miR-3657 | 1.714266402 | 2.088094121 | 1.779444413 | 1.765507927 | 1.958013949 | 1.775162793 |
| hsa-miR-3658 | 1.719781956 | 1.681731046 | 1.775720923 | 1.622309066 | 1.782254806 | 1.659173248 |
| hsa-miR-3659 | 1.75707598 | 1.732706837 | 1.787286713 | 2.191688189 | 1.777152894 | 1.691977783 |
| hsa-miR-365a-3p | 6.395435216 | 4.654013936 | 5.233610799 | 6.729346511 | 6.080914268 | 7.954855418 |
| hsa-miR-365a-5p | 2.021647323 | 1.77193349 | 1.911092859 | 2.945610408 | 1.673460071 | 1.758433377 |
| hsa-miR-365b-5p | 1.740889568 | 1.718022201 | 1.698263969 | 1.708105138 | 1.858421391 | 1.733412568 |
| hsa-miR-3660 | 1.755481013 | 1.714824293 | 1.744217179 | 2.265190211 | 1.817344546 | 1.81544497 |
| hsa-miR-3661 | 1.680759613 | 1.654986041 | 1.697148814 | 1.679533104 | 1.835204093 | 1.652521307 |
| hsa-miR-3662 | 1.676223909 | 1.81221694 | 1.788663352 | 1.648469981 | 2.340744202 | 2.166229717 |
| hsa-miR-3663-3p | 5.806422262 | 6.356341372 | 2.296783196 | 6.614989948 | 1.766857606 | 1.727671028 |
| hsa-miR-3663-5p | 1.634579452 | 1.719141964 | 1.794799521 | 1.820728188 | 1.99189834 | 1.89467845 |
| hsa-miR-3664-3p | 1.642730572 | 1.680017581 | 1.690368686 | 1.689119623 | 1.820212487 | 1.687769983 |
| hsa-miR-3664-5p | 1.778246914 | 1.753941694 | 1.752570045 | 1.692919375 | 2.105648294 | 1.696908885 |
| hsa-miR-3665 | 7.956442949 | 8.042689313 | 3.873927804 | 4.591450026 | 2.047473299 | 2.259737577 |
| hsa-miR-3666 | 1.676580981 | 1.755091268 | 1.787012549 | 1.787375 | 2.043219222 | 1.730780066 |
| hsa-miR-3667-3p | 1.668160229 | 1.923969771 | 1.799619716 | 1.666720679 | 1.94184257 | 1.802367007 |
| hsa-miR-3667-5p | 5.818852869 | 1.860063227 | 2.657696851 | 7.149876784 | 2.071363928 | 5.178531871 |
| hsa-miR-3668 | 1.890751004 | 1.872476678 | 1.689144086 | 1.795221558 | 2.887057939 | 1.77538522 |
| hsa-miR-367-3p | 1.658265718 | 1.848571195 | 1.743813118 | 1.73779446 | 2.165583936 | 2.154095535 |
| hsa-miR-367-5p | 1.730387711 | 2.204948901 | 1.862745963 | 1.679647075 | 2.782778081 | 1.896422909 |
| hsa-miR-3670 | 1.714086439 | 2.126924087 | 1.749319546 | 1.695503053 | 2.359518991 | 1.791702443 |
| hsa-miR-3671 | 1.725005523 | 1.739513826 | 1.841204923 | 1.658004155 | 2.12796599 | 1.847596245 |
| hsa-miR-3672 | 1.701865161 | 1.891622099 | 2.091352559 | 1.726240908 | 1.940858704 | 1.896733821 |
| hsa-miR-3674 | 1.686787565 | 1.678200948 | 1.791592196 | 1.683317876 | 1.735188152 | 1.75162849 |
| hsa-miR-3675-3p | 2.936035641 | 2.911502883 | 2.717169394 | 2.203992496 | 4.093480815 | 3.52389146 |
| hsa-miR-3675-5p | 1.793613187 | 1.767434707 | 1.91861306 | 1.661424206 | 2.06474494 | 1.768232265 |
| hsa-miR-3677-3p | 1.677466149 | 1.66743462 | 1.665340031 | 1.722463505 | 1.716477138 | 1.666054108 |
| hsa-miR-3677-5p | 1.71548862 | 1.816246587 | 1.68755578 | 1.594314983 | 1.636465391 | 1.7118899 |
| hsa-miR-3678-3p | 1.820537105 | 1.968963738 | 1.713600464 | 1.771800911 | 1.652683312 | 1.629222697 |
| hsa-miR-3678-5p | 1.768728881 | 1.690952408 | 1.800580344 | 1.71696668 | 1.696709001 | 1.726057875 |
| hsa-miR-3679-3p | 2.734434517 | 2.163669368 | 3.959959135 | 2.946813604 | 5.425287907 | 2.712091059 |
| hsa-miR-3679-5p | 11.00262059 | 9.499893055 | 9.378015713 | 10.35423109 | 2.80289959 | 8.309683725 |
| hsa-miR-3680-3p | 1.971794036 | 1.703674849 | 1.708806217 | 2.48204734 | 1.656472045 | 1.663237428 |
| hsa-miR-3680-5p | 1.595716982 | 1.766448718 | 1.674855438 | 1.636878161 | 2.213883208 | 1.743876602 |
| hsa-miR-3681-3p | 1.659740928 | 1.840362732 | 1.777081821 | 1.693891943 | 1.914535867 | 1.86670577 |
| hsa-miR-3681-5p | 1.6671717 | 1.77123022 | 1.727037781 | 1.629155834 | 1.771011205 | 1.670320136 |
| hsa-miR-3682-3p | 3.796829563 | 2.206209112 | 2.786335451 | 4.235038798 | 1.974326697 | 2.875391424 |
| hsa-miR-3682-5p | 1.895435705 | 1.765954758 | 1.918437087 | 1.776392023 | 1.890185054 | 1.766886034 |
| hsa-miR-3683 | 1.76317675 | 1.870350702 | 1.711377549 | 1.833366685 | 1.686657368 | 1.711747384 |
| hsa-miR-3684 | 1.695807997 | 1.672224746 | 1.677698558 | 1.695825646 | 1.793581574 | 1.677994779 |
| hsa-miR-3685 | 1.653887606 | 1.684621894 | 1.645612071 | 1.719369969 | 1.962161945 | 1.684081595 |
| hsa-miR-3686 | 1.728487105 | 1.582053115 | 1.756708427 | 1.663682125 | 1.695516172 | 1.624612355 |
| hsa-miR-3687 | 1.814947571 | 1.688888555 | 1.76102906 | 1.694537384 | 1.771469143 | 1.643503498 |
| hsa-miR-3688-3p | 1.688805777 | 1.732711789 | 1.708385235 | 1.685591053 | 2.449961108 | 1.642976721 |
| hsa-miR-3688-5p | 1.792909828 | 1.934385668 | 1.704403716 | 1.765709884 | 1.986472222 | 1.912022923 |
| hsa-miR-3689a-3p | 1.716972779 | 1.977217679 | 1.901142158 | 1.742459085 | 1.942658795 | 1.801124 |
| hsa-miR-3689a-5p | 3.787042381 | 2.155087822 | 1.775428563 | 2.504429803 | 1.885347546 | 1.902537196 |
| hsa-miR-3689b-3p | 2.096812012 | 1.925530639 | 1.79129786 | 1.688531907 | 1.895464157 | 1.755810371 |
| hsa-miR-3689d | 1.712309587 | 1.708163577 | 1.72855053 | 1.662643329 | 1.837399834 | 1.73310087 |
| hsa-miR-3689f | 4.765922843 | 3.319472491 | 1.779588421 | 2.871074972 | 1.743410172 | 2.267694993 |
| hsa-miR-369-3p | 1.895955629 | 1.787217938 | 2.204535775 | 1.743697435 | 2.507814189 | 2.336229084 |
| hsa-miR-369-5p | 1.761721619 | 1.998307096 | 1.970445162 | 1.675520652 | 2.88568 | 2.096432125 |
| hsa-miR-3690 | 1.65458584 | 1.704079458 | 1.674215244 | 1.696383039 | 1.695653589 | 1.71017614 |
| hsa-miR-3691-3p | 1.682065858 | 1.716667642 | 1.641043342 | 1.64135244 | 1.672615557 | 1.671290531 |
| hsa-miR-3691-5p | 1.746536921 | 1.725796625 | 1.621845393 | 1.757065925 | 1.870373577 | 1.673860031 |
| hsa-miR-3692-3p | 1.721334596 | 1.703357833 | 1.662346218 | 1.682775064 | 1.743658569 | 1.679695957 |
| hsa-miR-3692-5p | 2.047175991 | 1.758475594 | 1.739949141 | 1.727728279 | 1.71495739 | 1.648237776 |
| hsa-miR-370-3p | 1.776535106 | 1.684379343 | 1.654574252 | 2.798509296 | 1.710981415 | 1.67376635 |
| hsa-miR-370-5p | 1.742027272 | 1.966479414 | 1.824232759 | 1.662716487 | 1.819541379 | 1.777432501 |
| hsa-miR-3713 | 1.83448309 | 1.877024523 | 2.012076968 | 2.341425905 | 2.051642186 | 1.851069547 |
| hsa-miR-3714 | 1.887678825 | 1.768537573 | 1.736783138 | 1.741762531 | 1.732297392 | 1.767603821 |
| hsa-miR-371a-3p | 1.698641376 | 2.068830733 | 2.054235504 | 1.811498205 | 3.520696308 | 1.980192467 |
| hsa-miR-371a-5p | 4.605756597 | 5.682740612 | 3.134222875 | 6.401858686 | 1.932476927 | 2.799445186 |
| hsa-miR-371b-3p | 1.683389942 | 1.626624993 | 1.803978334 | 1.616777448 | 2.06044596 | 1.704425917 |
| hsa-miR-371b-5p | 7.710051784 | 4.923792435 | 2.155490119 | 2.029690241 | 1.749436545 | 1.909546507 |
| hsa-miR-372-3p | 1.702389019 | 1.810084981 | 1.731509996 | 1.625593794 | 1.799172325 | 1.688842102 |
| hsa-miR-372-5p | 1.70077776 | 2.493143771 | 2.022939281 | 1.737593883 | 4.306039697 | 2.215070555 |
| hsa-miR-373-3p | 1.855671148 | 1.676471556 | 1.736672146 | 1.813421967 | 2.340233343 | 1.855821396 |
| hsa-miR-373-5p | 1.759614807 | 1.690086784 | 1.744709551 | 1.968864581 | 1.751014652 | 1.7515205 |
| hsa-miR-374a-3p | 2.071891911 | 1.686993982 | 1.951035991 | 1.776666154 | 2.099936709 | 1.737420312 |
| hsa-miR-374a-5p | 2.086137252 | 1.925474559 | 2.128590902 | 1.747657139 | 1.998941091 | 2.08162105 |
| hsa-miR-374b-3p | 1.75087443 | 1.990221467 | 1.921347929 | 1.838294113 | 2.411387827 | 2.091239056 |
| hsa-miR-374b-5p | 2.271166704 | 1.728021118 | 1.705262389 | 1.813352152 | 1.793948162 | 1.709745945 |
| hsa-miR-374c-3p | 1.917764931 | 1.760981269 | 1.71960196 | 1.924369158 | 2.41619249 | 1.787322699 |
| hsa-miR-374c-5p | 1.663326951 | 1.931915152 | 1.691668752 | 1.676310538 | 1.734867675 | 1.893752352 |
| hsa-miR-375 | 1.776854729 | 1.790141197 | 1.826249777 | 2.086924568 | 1.814140254 | 1.873024397 |
| hsa-miR-376a-2-5p | 1.716256202 | 2.003820604 | 1.790072362 | 1.683488391 | 2.522995867 | 1.82966522 |
| hsa-miR-376a-3p | 1.693465577 | 1.713837707 | 2.266571593 | 1.935709942 | 2.903821799 | 3.255989699 |
| hsa-miR-376a-5p | 1.971018257 | 2.159550296 | 1.750570605 | 1.713164066 | 2.140850497 | 1.84876301 |
| hsa-miR-376b-3p | 1.748634389 | 1.787619925 | 2.474201681 | 1.686867526 | 2.340412706 | 1.873742518 |
| hsa-miR-376b-5p | 1.977869289 | 2.458113902 | 1.736678836 | 1.731546028 | 1.920473048 | 1.93205805 |
| hsa-miR-376c-3p | 1.663265525 | 1.698075612 | 1.732167208 | 1.663687629 | 1.860411013 | 1.988915907 |
| hsa-miR-376c-5p | 1.785776463 | 1.858708601 | 1.976468159 | 1.816715844 | 2.529944839 | 2.183598959 |
| hsa-miR-377-3p | 1.721978716 | 2.084685313 | 2.022182166 | 2.645427648 | 3.501093224 | 7.575422869 |
| hsa-miR-377-5p | 1.791326895 | 1.647147395 | 1.743773288 | 1.722967042 | 1.818821501 | 1.625197352 |
| hsa-miR-378a-3p | 3.92837632 | 3.456340334 | 3.058951327 | 2.918018803 | 1.659716286 | 3.561773351 |
| hsa-miR-378a-5p | 1.752227621 | 1.696702491 | 1.783224468 | 1.752662657 | 2.102408098 | 1.932604558 |
| hsa-miR-378b | 1.800989458 | 1.698264411 | 1.752659577 | 1.784470395 | 1.630831481 | 1.654824249 |
| hsa-miR-378c | 1.979796173 | 1.866563162 | 1.925438842 | 1.968536266 | 1.79344179 | 2.617367489 |
| hsa-miR-378d | 1.697109873 | 2.320649198 | 1.753623148 | 1.665316968 | 1.913254452 | 1.78574773 |
| hsa-miR-378e | 1.788706303 | 1.743103268 | 1.71477405 | 1.751029893 | 1.691514216 | 1.770020147 |
| hsa-miR-378f | 1.963917986 | 1.760465114 | 1.979360184 | 5.387020838 | 1.726955307 | 2.371393432 |
| hsa-miR-378g | 1.669610935 | 1.824003518 | 1.660248616 | 2.956140862 | 1.769078421 | 1.706228524 |
| hsa-miR-378h | 1.699996868 | 1.772128994 | 1.707976296 | 1.753313228 | 1.982412094 | 1.691280665 |
| hsa-miR-378i | 2.575523217 | 3.726448755 | 3.580716649 | 3.621806234 | 1.945205117 | 4.67897165 |
| hsa-miR-378j | 1.744184833 | 1.722021724 | 1.68226853 | 6.376949888 | 1.754234961 | 7.020810309 |
| hsa-miR-379-3p | 1.687436695 | 1.654921725 | 1.742124513 | 1.696353915 | 1.901310009 | 1.7184876 |
| hsa-miR-379-5p | 1.610337564 | 1.754221886 | 1.733756313 | 1.782698904 | 1.780538345 | 1.774721455 |
| hsa-miR-380-3p | 1.873255267 | 1.759052504 | 1.769273695 | 1.771771523 | 1.948675859 | 2.170082774 |
| hsa-miR-380-5p | 1.610509987 | 1.873209729 | 1.786921181 | 1.580753384 | 2.265192874 | 1.784668773 |
| hsa-miR-381-3p | 1.723768807 | 1.726960377 | 1.753351247 | 2.138480059 | 1.695224229 | 3.144557163 |
| hsa-miR-381-5p | 1.714586487 | 2.243753108 | 1.921687627 | 1.703259504 | 2.020466593 | 1.963903499 |
| hsa-miR-382-3p | 1.757860301 | 1.738264104 | 1.615594686 | 1.581317839 | 1.618273275 | 1.606965991 |
| hsa-miR-382-5p | 1.690629709 | 1.894354754 | 1.762499693 | 1.896924495 | 1.919357625 | 1.765207181 |
| hsa-miR-383-3p | 1.700814806 | 1.782624556 | 1.730631921 | 1.648155743 | 1.866002879 | 1.735734447 |
| hsa-miR-383-5p | 1.692705013 | 1.622336741 | 1.737192408 | 1.656255099 | 1.741473554 | 1.723233355 |
| hsa-miR-384 | 1.846991117 | 1.999017188 | 1.917993041 | 1.840109498 | 2.352655426 | 1.785943135 |
| hsa-miR-3907 | 1.769709722 | 1.817669133 | 1.884684696 | 1.674071419 | 1.830456266 | 1.827663756 |
| hsa-miR-3908 | 1.669247301 | 1.761596024 | 1.867626027 | 1.69499174 | 1.874892937 | 1.769659728 |
| hsa-miR-3909 | 1.695278601 | 1.636629332 | 1.719892571 | 1.646329051 | 1.777254361 | 1.745427971 |
| hsa-miR-3910 | 1.674933782 | 1.67023932 | 1.768013619 | 1.598925792 | 1.749497877 | 1.613167711 |
| hsa-miR-3911 | 4.292177163 | 2.200622964 | 2.950753094 | 7.774712206 | 1.833300698 | 4.346865153 |
| hsa-miR-3912-3p | 1.801763694 | 1.692515953 | 1.721190452 | 1.680186644 | 1.750802769 | 1.799220433 |
| hsa-miR-3912-5p | 1.812362891 | 1.836362215 | 1.814755364 | 5.50707358 | 2.352161014 | 1.914646985 |
| hsa-miR-3913-3p | 1.810042508 | 1.736459826 | 1.642751784 | 1.651818069 | 1.825609056 | 1.697483338 |
| hsa-miR-3913-5p | 1.651919106 | 1.798062578 | 1.787967164 | 1.679637792 | 1.986790517 | 1.944508403 |
| hsa-miR-3914 | 1.698706124 | 1.649267598 | 1.644472642 | 2.63782931 | 1.723614841 | 1.676299624 |
| hsa-miR-3915 | 1.930509009 | 1.755130495 | 1.979615066 | 2.145335651 | 1.956874282 | 1.84772889 |
| hsa-miR-3916 | 1.771612856 | 1.9867464 | 1.783362796 | 1.627239704 | 1.686353382 | 1.780656103 |
| hsa-miR-3917 | 3.970713388 | 3.264147926 | 3.717647541 | 5.338430736 | 1.758267013 | 4.746383202 |
| hsa-miR-3918 | 1.671565487 | 1.782221763 | 1.784555343 | 1.680039892 | 1.763946451 | 1.753678189 |
| hsa-miR-3919 | 1.873807022 | 1.72506327 | 1.703129017 | 1.64645897 | 1.772455753 | 1.711880859 |
| hsa-miR-3920 | 1.815631175 | 1.78058217 | 1.835454817 | 1.786995246 | 3.484863394 | 1.827739887 |
| hsa-miR-3921 | 1.718358685 | 1.82075321 | 1.86641501 | 1.754221705 | 2.559222737 | 1.851946161 |
| hsa-miR-3922-3p | 1.749807183 | 1.749135567 | 2.3180634 | 1.716794036 | 1.796707053 | 1.856890225 |
| hsa-miR-3922-5p | 1.882472275 | 1.749518681 | 1.824378246 | 1.755261545 | 1.724956911 | 1.726741338 |
| hsa-miR-3923 | 2.246512932 | 2.536628139 | 2.227608243 | 5.757523263 | 2.016257426 | 5.597809271 |
| hsa-miR-3924 | 1.907978057 | 1.729608303 | 1.871604694 | 1.663677407 | 2.67729567 | 1.822472935 |
| hsa-miR-3925-3p | 1.741262111 | 1.715776743 | 1.772484076 | 1.795310796 | 2.325518389 | 1.848021701 |
| hsa-miR-3925-5p | 1.772610996 | 2.009657232 | 1.764409228 | 1.944303994 | 1.656625285 | 1.777999925 |
| hsa-miR-3926 | 2.091029877 | 2.155151734 | 1.967467864 | 2.737228401 | 2.276972928 | 1.890752183 |
| hsa-miR-3927-3p | 1.940723648 | 1.723567455 | 1.965744626 | 1.736159703 | 2.099627627 | 1.835621112 |
| hsa-miR-3927-5p | 1.72100063 | 1.84316496 | 2.284539558 | 1.743417045 | 2.053037829 | 1.756178789 |
| hsa-miR-3928-3p | 1.70209251 | 1.724307084 | 1.68287649 | 1.749546207 | 1.837758536 | 1.743334858 |
| hsa-miR-3928-5p | 1.790177825 | 1.755760713 | 1.657517922 | 1.633905276 | 1.707195414 | 1.658546184 |
| hsa-miR-3929 | 1.683718361 | 1.895481923 | 1.834233228 | 1.800792569 | 1.809219045 | 1.813314795 |
| hsa-miR-3934-3p | 3.078609479 | 2.040236956 | 1.851620222 | 2.267885321 | 1.754131425 | 1.96412638 |
| hsa-miR-3934-5p | 1.980046261 | 1.836236362 | 2.020608182 | 2.382475669 | 1.848105375 | 1.723364951 |
| hsa-miR-3935 | 1.783256294 | 2.023202873 | 1.71680444 | 2.145969066 | 1.753887348 | 1.783684454 |
| hsa-miR-3936 | 1.710466551 | 1.905665044 | 1.877106403 | 1.651805914 | 1.912883218 | 1.890068633 |
| hsa-miR-3937 | 5.020026123 | 4.886883235 | 4.630100371 | 4.991896099 | 1.859846254 | 5.349949706 |
| hsa-miR-3938 | 1.750448142 | 1.680465068 | 1.714465081 | 1.697547446 | 1.72187559 | 1.827979916 |
| hsa-miR-3939 | 1.635430167 | 1.720312982 | 2.052448181 | 1.660048414 | 2.040023327 | 1.849726218 |
| hsa-miR-3940-3p | 1.946452218 | 1.921692539 | 1.860016204 | 1.912426825 | 1.907881879 | 1.771727619 |
| hsa-miR-3940-5p | 4.256957852 | 2.025522408 | 1.72263552 | 3.541916991 | 1.836368888 | 1.737653468 |
| hsa-miR-3941 | 1.730038981 | 1.613103187 | 1.708921834 | 1.616960121 | 1.7534444 | 1.687096273 |
| hsa-miR-3942-3p | 1.767430112 | 1.712679888 | 1.984494015 | 1.802249481 | 1.893496595 | 2.376982286 |
| hsa-miR-3942-5p | 1.685184084 | 1.781616617 | 1.849077197 | 1.731306785 | 2.464259895 | 1.744379977 |
| hsa-miR-3943 | 1.671545599 | 1.744502169 | 1.771106466 | 1.589979758 | 1.766353111 | 1.770912108 |
| hsa-miR-3944-3p | 1.661018874 | 1.796461041 | 1.768402515 | 1.55073417 | 1.729118718 | 1.911857698 |
| hsa-miR-3944-5p | 2.932919487 | 3.217106749 | 2.698464308 | 2.449402799 | 2.392516036 | 3.062961311 |
| hsa-miR-3945 | 2.002806525 | 1.788775052 | 1.930576004 | 5.856367272 | 2.546057902 | 2.132968774 |
| hsa-miR-3960 | 10.00343408 | 11.16027016 | 10.04263327 | 10.33882627 | 11.86096943 | 8.107473224 |
| hsa-miR-3972 | 1.674722351 | 1.823268209 | 1.711947367 | 1.779543645 | 1.74575247 | 1.677421704 |
| hsa-miR-3973 | 1.651444022 | 1.7418378 | 1.912237987 | 1.690191168 | 1.889218237 | 1.860253214 |
| hsa-miR-3974 | 1.655647656 | 1.753025823 | 1.803180194 | 1.650554159 | 1.903615974 | 1.853180639 |
| hsa-miR-3975 | 1.771106582 | 1.80537491 | 1.955526273 | 1.662763618 | 1.934552227 | 1.812569242 |
| hsa-miR-3976 | 3.494527787 | 1.71353606 | 1.759317379 | 1.790369992 | 1.813756435 | 1.688379159 |
| hsa-miR-3977 | 1.659261728 | 1.801669289 | 1.785216089 | 1.642721644 | 2.213098247 | 1.854973742 |
| hsa-miR-3978 | 1.718984471 | 1.765835867 | 1.72439161 | 1.726910245 | 1.740298181 | 1.728204876 |
| hsa-miR-409-3p | 1.705680649 | 1.675135153 | 1.685561429 | 2.374238153 | 1.605889549 | 1.665837612 |
| hsa-miR-409-5p | 1.685197338 | 1.812103138 | 1.8742936 | 1.596728225 | 1.68281464 | 1.719086355 |
| hsa-miR-410-3p | 1.70709542 | 1.695074275 | 2.032348111 | 1.880196142 | 2.185889354 | 3.300535563 |
| hsa-miR-410-5p | 1.792463916 | 1.709031653 | 1.794686125 | 1.782500117 | 1.774296372 | 1.743579519 |
| hsa-miR-411-3p | 1.715117685 | 1.7676604 | 1.653496064 | 1.63046256 | 2.110890941 | 1.74021871 |
| hsa-miR-411-5p | 1.656580589 | 1.811151475 | 1.754970114 | 1.631987446 | 1.698517701 | 1.740506404 |
| hsa-miR-412-3p | 1.656065272 | 1.736106436 | 1.804176252 | 1.58910185 | 1.716636885 | 1.684208511 |
| hsa-miR-412-5p | 1.68875462 | 1.873932705 | 1.747864865 | 1.706280856 | 1.711520272 | 1.868559152 |
| hsa-miR-421 | 1.817666315 | 1.657999572 | 1.702451074 | 1.792884521 | 1.689750802 | 1.70354359 |
| hsa-miR-422a | 1.959018742 | 1.668012459 | 1.655218551 | 2.066862935 | 1.738435846 | 1.668537152 |
| hsa-miR-423-3p | 1.756887595 | 1.829963341 | 1.720532942 | 2.15223486 | 1.767352841 | 1.791040741 |
| hsa-miR-423-5p | 6.248924337 | 5.506281791 | 5.230872947 | 8.742530539 | 2.039534975 | 6.370289512 |
| hsa-miR-424-3p | 1.718760984 | 1.651374003 | 1.764842239 | 2.072596407 | 2.065622256 | 1.712293624 |
| hsa-miR-424-5p | 2.300515735 | 1.830196198 | 1.91716553 | 5.259009637 | 2.828418838 | 8.208727758 |
| hsa-miR-425-3p | 4.711629411 | 4.738605011 | 5.785303114 | 3.405482253 | 8.218575683 | 5.95689001 |
| hsa-miR-425-5p | 7.167056981 | 5.770820347 | 8.486693432 | 6.734456151 | 2.029698541 | 6.883982299 |
| hsa-miR-4251 | 1.690303883 | 1.702054624 | 1.760222241 | 1.671646335 | 1.801719193 | 1.812048061 |
| hsa-miR-4252 | 1.678985665 | 1.799668354 | 1.783782713 | 1.633767712 | 1.8379799 | 1.968679669 |
| hsa-miR-4253 | 5.449893223 | 1.76541522 | 2.164887878 | 3.145415311 | 1.707663238 | 2.743057738 |
| hsa-miR-4254 | 1.730832004 | 1.73674275 | 1.852201834 | 1.668456821 | 1.65977548 | 1.738172611 |
| hsa-miR-4255 | 1.672324423 | 1.760190708 | 1.781735501 | 1.710751797 | 1.858844206 | 1.957134125 |
| hsa-miR-4256 | 1.688889731 | 1.735641233 | 1.626614122 | 1.684190456 | 1.996821109 | 1.742192847 |
| hsa-miR-4257 | 5.51468546 | 5.766836829 | 4.047676699 | 4.999899718 | 1.871991901 | 4.703884194 |
| hsa-miR-4258 | 1.647889568 | 1.908509195 | 1.714442755 | 1.860017308 | 1.691380497 | 1.683434991 |
| hsa-miR-4259 | 2.623078315 | 2.15910843 | 2.109037991 | 4.006336194 | 1.742430032 | 2.291289157 |
| hsa-miR-4260 | 1.609366201 | 1.716376839 | 1.742806126 | 1.686050457 | 1.796588846 | 1.763561276 |
| hsa-miR-4261 | 1.82365516 | 1.757122608 | 1.816157639 | 1.832082039 | 1.640836748 | 1.692423185 |
| hsa-miR-4262 | 1.678340941 | 1.674597974 | 1.839199784 | 1.589997679 | 1.81619717 | 1.696708191 |
| hsa-miR-4263 | 1.683560621 | 1.709008907 | 1.889774349 | 1.63079726 | 1.823727485 | 1.809913682 |
| hsa-miR-4264 | 1.867512888 | 1.7438705 | 1.74348328 | 1.709530112 | 1.739310647 | 1.708435374 |
| hsa-miR-4265 | 1.795780773 | 1.764812923 | 1.799279169 | 2.775534986 | 1.70321146 | 2.295010851 |
| hsa-miR-4266 | 1.686574547 | 1.715878138 | 1.67332072 | 1.619564052 | 1.773135043 | 1.7198075 |
| hsa-miR-4267 | 1.64111706 | 1.746312175 | 1.716579909 | 1.706749928 | 1.756591525 | 1.704006388 |
| hsa-miR-4268 | 1.69335241 | 1.744083339 | 1.643964268 | 1.630213958 | 3.185719039 | 1.707663355 |
| hsa-miR-4269 | 1.679227547 | 1.655839504 | 1.818575417 | 1.703074939 | 1.686336031 | 1.732518753 |
| hsa-miR-4270 | 5.075438582 | 2.83805325 | 2.623867975 | 7.071512137 | 2.225968989 | 5.01970851 |
| hsa-miR-4271 | 7.456377109 | 7.165224401 | 7.7052108 | 7.562668106 | 2.84043714 | 7.485690841 |
| hsa-miR-4272 | 1.815488119 | 2.026292399 | 1.87598263 | 1.671136063 | 1.920639816 | 1.747605625 |
| hsa-miR-4273 | 1.780992424 | 1.739275261 | 1.682618516 | 1.802214708 | 1.683177195 | 1.713467189 |
| hsa-miR-4274 | 2.352394558 | 3.497124334 | 2.146779236 | 2.397134294 | 3.366707193 | 2.215443364 |
| hsa-miR-4275 | 1.772530868 | 1.90059227 | 1.814202897 | 1.68534109 | 1.775132874 | 1.680613062 |
| hsa-miR-4276 | 1.745441985 | 1.721463816 | 1.714126856 | 1.696903787 | 1.705498262 | 1.771627615 |
| hsa-miR-4277 | 1.685753175 | 1.757691583 | 1.718620436 | 3.672976732 | 1.805750867 | 1.783847367 |
| hsa-miR-4278 | 1.659455263 | 1.757265915 | 1.751735125 | 1.705764937 | 1.802133686 | 1.701145575 |
| hsa-miR-4279 | 1.807787552 | 1.701641768 | 1.773882975 | 1.736639027 | 1.82512514 | 1.697822816 |
| hsa-miR-4280 | 1.642228085 | 1.768874998 | 1.735549172 | 1.772089725 | 1.889325788 | 1.736926884 |
| hsa-miR-4281 | 9.826045493 | 10.6197678 | 10.17974033 | 10.72916624 | 10.29159123 | 8.829522853 |
| hsa-miR-4282 | 2.462940302 | 2.047996706 | 1.774740506 | 3.031208906 | 1.998037584 | 1.883566532 |
| hsa-miR-4283 | 1.68783071 | 1.740545701 | 1.900532213 | 1.605124912 | 1.794752509 | 1.829387139 |
| hsa-miR-4284 | 3.743999626 | 5.531743128 | 5.254515703 | 3.189098699 | 6.206856583 | 4.759505099 |
| hsa-miR-4285 | 1.610217728 | 1.677280758 | 1.809093183 | 1.62764503 | 2.114143157 | 1.743911886 |
| hsa-miR-4286 | 3.909604103 | 5.466073169 | 1.977250373 | 4.628377528 | 2.323063152 | 5.672976447 |
| hsa-miR-4287 | 1.730295691 | 1.703461878 | 1.614044727 | 1.650569266 | 1.911544148 | 1.700389329 |
| hsa-miR-4288 | 1.660141983 | 2.123999519 | 1.710758265 | 1.656254564 | 1.990488565 | 1.872473888 |
| hsa-miR-4289 | 1.700226289 | 1.687265153 | 1.711571656 | 1.650159496 | 1.896997642 | 1.709350453 |
| hsa-miR-429 | 1.86747757 | 1.775008843 | 1.71112587 | 1.66885885 | 2.135778455 | 1.802755159 |
| hsa-miR-4290 | 1.728467898 | 1.91701779 | 1.920314802 | 1.691714152 | 1.790083804 | 1.838000793 |
| hsa-miR-4291 | 2.161195677 | 1.921107478 | 1.782305713 | 3.460577863 | 1.7154699 | 4.542284549 |
| hsa-miR-4292 | 1.604677921 | 1.698092168 | 1.70030354 | 1.656873173 | 1.734365621 | 1.630580911 |
| hsa-miR-4293 | 1.757011216 | 1.627437537 | 1.61538635 | 1.629103659 | 1.724754219 | 1.526326453 |
| hsa-miR-4294 | 2.453737942 | 1.707060467 | 1.776648704 | 4.282555872 | 1.721702296 | 2.262740995 |
| hsa-miR-4295 | 1.911546154 | 1.767224603 | 1.932128227 | 1.670193305 | 2.103676278 | 1.842223204 |
| hsa-miR-4296 | 2.072220335 | 1.825956284 | 2.063090403 | 1.78632602 | 1.807870631 | 1.849036328 |
| hsa-miR-4297 | 1.747102131 | 1.837197997 | 1.724349569 | 1.765471034 | 1.840617129 | 1.667147898 |
| hsa-miR-4298 | 7.499831638 | 7.265317028 | 7.953944765 | 11.02345602 | 2.059235592 | 8.240823551 |
| hsa-miR-4299 | 7.801021597 | 2.688992954 | 3.101968032 | 6.359979521 | 1.950877515 | 3.274609201 |
| hsa-miR-4300 | 1.640181671 | 1.780557845 | 2.042872315 | 1.686260507 | 1.744421733 | 1.760532721 |
| hsa-miR-4301 | 1.939230045 | 2.250675428 | 1.805157754 | 1.742761033 | 1.84373051 | 1.655660799 |
| hsa-miR-4302 | 1.609788652 | 1.930237474 | 1.742288076 | 1.769689038 | 1.760828919 | 1.7134885 |
| hsa-miR-4303 | 1.726602628 | 1.755222785 | 1.776852991 | 1.772748784 | 1.717569082 | 1.699270283 |
| hsa-miR-4304 | 1.869012516 | 1.757280575 | 1.776969466 | 1.875866817 | 1.878572254 | 1.816622614 |
| hsa-miR-4305 | 1.694032398 | 1.773906248 | 1.670320563 | 1.683967068 | 1.73134441 | 1.666752896 |
| hsa-miR-4306 | 8.267260538 | 6.947427087 | 8.030428833 | 9.218528873 | 2.713408945 | 10.48502308 |
| hsa-miR-4307 | 1.836677028 | 1.811740324 | 1.939074039 | 1.70733447 | 2.094464831 | 1.840474239 |
| hsa-miR-4308 | 1.656745299 | 1.764065906 | 1.746294332 | 1.701464123 | 1.907098123 | 1.78164565 |
| hsa-miR-4309 | 1.786717611 | 1.66031949 | 1.771671157 | 1.634339259 | 1.794727213 | 1.744979459 |
| hsa-miR-431-3p | 1.763289543 | 1.891150054 | 1.765076128 | 1.904911764 | 1.91176708 | 1.752410667 |
| hsa-miR-431-5p | 1.622228692 | 1.822494358 | 1.746440731 | 1.799996329 | 1.739022961 | 1.696863033 |
| hsa-miR-4310 | 2.525154028 | 2.813057128 | 3.407140353 | 2.196282847 | 6.168374046 | 4.153616741 |
| hsa-miR-4311 | 1.653213003 | 1.916138406 | 1.754894284 | 2.494574712 | 1.772899123 | 1.938777903 |
| hsa-miR-4312 | 1.793155697 | 1.952231346 | 2.543249265 | 1.946533826 | 2.021469196 | 1.868875403 |
| hsa-miR-4313 | 5.330593741 | 6.532768043 | 7.481677955 | 4.445869234 | 9.825340322 | 6.804548721 |
| hsa-miR-4314 | 5.743808411 | 2.630588794 | 1.981998557 | 5.319649237 | 1.714379667 | 1.811669909 |
| hsa-miR-4315 | 1.651982239 | 1.954161596 | 1.764341762 | 1.676947477 | 2.812011855 | 1.908760231 |
| hsa-miR-4316 | 1.692337285 | 1.711603681 | 1.884619884 | 3.939728153 | 1.95885101 | 1.792925191 |
| hsa-miR-4317 | 1.981625427 | 1.734672946 | 1.695026699 | 2.13332503 | 1.764535064 | 1.785906483 |
| hsa-miR-4318 | 1.758873227 | 1.67639825 | 1.708372712 | 1.89385264 | 1.856062068 | 1.675786282 |
| hsa-miR-4319 | 1.744558106 | 1.723730473 | 1.701550273 | 1.651020873 | 1.843509115 | 1.736281594 |
| hsa-miR-432-3p | 1.674538336 | 1.706424258 | 1.731156459 | 1.618027754 | 2.002882451 | 1.772967256 |
| hsa-miR-432-5p | 1.78721239 | 1.757879402 | 1.766945993 | 3.706075982 | 1.768910172 | 4.695463683 |
| hsa-miR-4320 | 1.882332576 | 1.832987218 | 1.91215477 | 1.716096997 | 3.075100183 | 1.691571465 |
| hsa-miR-4321 | 1.708930001 | 1.98409087 | 1.65123597 | 1.794473705 | 1.671993922 | 1.672254732 |
| hsa-miR-4322 | 2.412257543 | 2.162901759 | 1.914497317 | 5.081202782 | 1.964478304 | 2.950918566 |
| hsa-miR-4323 | 3.379323876 | 4.815070583 | 4.039303128 | 2.684623366 | 5.197711173 | 3.779194963 |
| hsa-miR-4324 | 1.621463336 | 1.578767606 | 1.60902494 | 3.012696855 | 1.666776171 | 1.903019536 |
| hsa-miR-4325 | 1.800444117 | 1.77586737 | 1.789253332 | 1.731587627 | 1.710898688 | 1.742685808 |
| hsa-miR-4326 | 1.664899622 | 2.002415252 | 1.909299108 | 1.644301104 | 1.717769615 | 1.817507478 |
| hsa-miR-4327 | 7.498203567 | 5.068887493 | 3.184009789 | 6.86083548 | 1.670279067 | 2.100801324 |
| hsa-miR-4328 | 1.664643537 | 2.13994624 | 1.781689527 | 2.404230734 | 1.783685373 | 2.089520348 |
| hsa-miR-4329 | 1.689906338 | 1.7350823 | 1.746108564 | 1.693566765 | 1.918892346 | 1.764626181 |
| hsa-miR-433-3p | 1.736998098 | 1.766028435 | 1.678593476 | 2.059474816 | 1.761430678 | 1.910943408 |
| hsa-miR-433-5p | 1.728710346 | 1.811381962 | 1.85500245 | 1.69266115 | 1.805767413 | 1.708955481 |
| hsa-miR-4330 | 1.655853753 | 1.897014348 | 1.801831851 | 1.664186104 | 1.657754178 | 1.773379646 |
| hsa-miR-4417 | 1.806573242 | 1.709435229 | 1.677152147 | 2.917167485 | 1.741232202 | 1.748485539 |
| hsa-miR-4418 | 1.718603002 | 1.931530949 | 1.786194971 | 1.682512292 | 1.759272855 | 1.719942349 |
| hsa-miR-4419a | 4.149626753 | 2.744082684 | 4.121702339 | 6.083412554 | 2.094225238 | 3.200987935 |
| hsa-miR-4419b | 2.571355127 | 2.108557553 | 2.045657547 | 6.101474581 | 1.646738564 | 1.807801254 |
| hsa-miR-4420 | 1.707017263 | 1.733807053 | 1.85257798 | 1.732962918 | 1.950637087 | 1.77608394 |
| hsa-miR-4421 | 1.719391042 | 1.6681518 | 1.734510447 | 1.810097564 | 1.897558779 | 1.785536306 |
| hsa-miR-4422 | 1.707424689 | 1.682151504 | 1.663603866 | 5.824654987 | 1.732190015 | 1.65760237 |
| hsa-miR-4423-3p | 1.69351174 | 1.690766815 | 1.756551331 | 1.639754633 | 1.739613498 | 1.740266298 |
| hsa-miR-4423-5p | 1.900515591 | 1.773458312 | 2.027305516 | 1.761104298 | 1.760474079 | 1.729718273 |
| hsa-miR-4424 | 1.802588926 | 1.684968705 | 1.971219873 | 1.753189528 | 1.902484501 | 1.656169976 |
| hsa-miR-4425 | 1.752457279 | 1.728936347 | 1.690997545 | 1.699643121 | 1.834274316 | 1.806098696 |
| hsa-miR-4426 | 1.835069347 | 1.751320639 | 1.716995198 | 1.65015741 | 1.725666815 | 1.680675356 |
| hsa-miR-4427 | 1.636919329 | 1.700655875 | 1.843435524 | 1.668712579 | 1.954232567 | 2.059622601 |
| hsa-miR-4428 | 3.685376733 | 2.020139441 | 2.144511614 | 3.05561364 | 1.804229793 | 3.759404889 |
| hsa-miR-4429 | 1.799787649 | 1.863107099 | 1.751623209 | 2.141369947 | 1.766937814 | 1.715053034 |
| hsa-miR-4430 | 3.76233389 | 2.136092017 | 2.818070353 | 3.785652757 | 2.078594968 | 2.929030816 |
| hsa-miR-4431 | 1.634397886 | 1.863795206 | 1.692279409 | 1.714924351 | 1.908215192 | 1.686789651 |
| hsa-miR-4432 | 1.645474918 | 1.719129548 | 1.666000388 | 1.631542888 | 1.963914205 | 1.756128179 |
| hsa-miR-4433a-3p | 4.493972998 | 2.2244172 | 2.448288301 | 5.691239496 | 2.058208513 | 1.863000055 |
| hsa-miR-4433a-5p | 5.330077762 | 6.686716025 | 7.291461616 | 3.69505766 | 9.601365742 | 7.028151551 |
| hsa-miR-4433b-3p | 2.152774177 | 2.058163718 | 1.737918624 | 3.06100472 | 1.737898269 | 1.632592823 |
| hsa-miR-4434 | 2.360874987 | 1.738069643 | 1.690120236 | 2.936202715 | 1.769442785 | 1.625491643 |
| hsa-miR-4435 | 1.649718788 | 1.628068052 | 1.71794938 | 1.701521747 | 1.774630623 | 1.646931721 |
| hsa-miR-4436a | 1.684511852 | 1.753785397 | 1.792562812 | 3.065669699 | 1.745676919 | 1.845795667 |
| hsa-miR-4436b-3p | 2.90538672 | 1.76754754 | 1.760345313 | 4.749093217 | 1.682259829 | 1.672008579 |
| hsa-miR-4436b-5p | 4.463970435 | 4.200056224 | 5.620850933 | 3.439530952 | 4.891357731 | 3.945902933 |
| hsa-miR-4437 | 1.814976212 | 1.692681051 | 1.745470835 | 1.761534455 | 1.788381912 | 1.707905689 |
| hsa-miR-4438 | 1.605328354 | 1.702944044 | 2.016558122 | 1.585881423 | 2.70347503 | 1.814816123 |
| hsa-miR-4439 | 1.700546022 | 1.852509083 | 1.689923932 | 1.631697229 | 1.763328578 | 1.718600722 |
| hsa-miR-4440 | 1.756804038 | 2.056541312 | 2.340764076 | 1.677359168 | 1.802874635 | 1.647614847 |
| hsa-miR-4441 | 2.387599163 | 1.735637764 | 1.743211046 | 5.795335984 | 1.94448111 | 1.772104257 |
| hsa-miR-4442 | 6.948259395 | 6.644019775 | 8.014930691 | 9.13998745 | 1.847098602 | 8.979133469 |
| hsa-miR-4443 | 8.194761881 | 2.784561925 | 1.92518747 | 3.717706123 | 1.720140773 | 2.13101844 |
| hsa-miR-4444 | 1.774320634 | 1.810421958 | 1.887136104 | 2.826601187 | 1.782229167 | 1.752184284 |
| hsa-miR-4445-3p | 1.650312962 | 1.71518822 | 1.775974189 | 1.624218892 | 1.664350542 | 1.704984231 |
| hsa-miR-4445-5p | 1.635861678 | 1.742057621 | 1.811301995 | 1.582309952 | 1.810208813 | 1.806126301 |
| hsa-miR-4446-3p | 1.726424491 | 1.649041463 | 1.789306357 | 1.70228561 | 1.698463046 | 1.763249788 |
| hsa-miR-4446-5p | 1.63947297 | 1.684272017 | 1.774209736 | 1.673321436 | 1.875116287 | 1.702081558 |
| hsa-miR-4447 | 1.815162462 | 1.794283097 | 1.923835042 | 2.780366994 | 1.829707422 | 1.963342273 |
| hsa-miR-4448 | 1.829615544 | 1.693572741 | 2.560614206 | 1.878698696 | 1.737751653 | 1.834028828 |
| hsa-miR-4449 | 1.907488076 | 1.662781919 | 1.658507352 | 1.696470755 | 1.694034456 | 1.720807926 |
| hsa-miR-4450 | 2.203895312 | 1.785524776 | 1.728294166 | 1.850069929 | 2.333975582 | 2.134008263 |
| hsa-miR-4451 | 1.811743301 | 1.824660628 | 1.984045233 | 1.871808086 | 1.791813085 | 1.810857776 |
| hsa-miR-4452 | 1.70890629 | 1.745552145 | 1.790289946 | 1.697252553 | 1.975511252 | 1.712628749 |
| hsa-miR-4453 | 1.699501944 | 1.666051443 | 1.705832709 | 1.709131905 | 1.741604544 | 1.735512132 |
| hsa-miR-4455 | 8.939781556 | 8.461219614 | 10.71356999 | 6.468584311 | 5.351244693 | 6.352972606 |
| hsa-miR-4456 | 1.696644931 | 2.191937323 | 1.917470801 | 1.696002951 | 1.775381311 | 1.727398182 |
| hsa-miR-4457 | 1.703609031 | 1.744883315 | 1.689527089 | 1.683776586 | 1.830846298 | 1.742222968 |
| hsa-miR-4458 | 2.210466452 | 1.791070396 | 1.666457152 | 1.826828449 | 1.688495717 | 1.71369718 |
| hsa-miR-4459 | 12.328463 | 11.30487768 | 11.71648192 | 12.43091801 | 12.31477819 | 9.083683524 |
| hsa-miR-4460 | 1.88100281 | 1.878555073 | 1.717402787 | 1.79634536 | 1.995218888 | 1.748580253 |
| hsa-miR-4461 | 1.719670052 | 1.816734754 | 1.744640251 | 1.724536037 | 1.805115026 | 1.767244339 |
| hsa-miR-4462 | 4.839876024 | 1.904836447 | 1.788233275 | 3.107712567 | 1.683175674 | 1.799100635 |
| hsa-miR-4463 | 2.384265417 | 1.865408253 | 1.715726675 | 4.905178938 | 1.873371154 | 1.699428748 |
| hsa-miR-4464 | 1.656933429 | 1.760719874 | 2.106447096 | 1.74208988 | 1.830830886 | 1.804236606 |
| hsa-miR-4465 | 6.517035509 | 3.442888096 | 4.92315145 | 4.078710075 | 1.905213052 | 6.735878703 |
| hsa-miR-4466 | 9.164011188 | 9.422338573 | 7.555187753 | 10.37988185 | 5.962586328 | 8.091237709 |
| hsa-miR-4467 | 1.680947583 | 1.701704895 | 1.873327538 | 1.722815199 | 1.803576982 | 1.749583004 |
| hsa-miR-4468 | 1.809578384 | 1.840975477 | 1.792547576 | 2.457987166 | 1.843123223 | 1.906167529 |
| hsa-miR-4469 | 1.673857862 | 1.728916918 | 1.665145662 | 1.654333589 | 1.771050183 | 1.753233274 |
| hsa-miR-4470 | 2.050126497 | 1.666455732 | 1.67926788 | 3.628855713 | 1.688307988 | 1.698218732 |
| hsa-miR-4471 | 1.754004431 | 1.744154867 | 1.793996248 | 1.695222521 | 1.887292422 | 1.719570926 |
| hsa-miR-4472 | 1.80135458 | 1.817225857 | 1.846080056 | 2.283023633 | 1.867862575 | 1.720591543 |
| hsa-miR-4473 | 1.685767166 | 1.781004389 | 1.802978111 | 1.578564638 | 1.992405005 | 1.749008964 |
| hsa-miR-4474-3p | 1.663601126 | 1.713927804 | 1.676920063 | 1.606504101 | 1.732005287 | 1.703142447 |
| hsa-miR-4474-5p | 1.769073656 | 1.757737434 | 1.747252485 | 1.644930253 | 2.039181697 | 1.705055858 |
| hsa-miR-4475 | 1.740563112 | 1.786342231 | 1.941054098 | 1.735266589 | 2.167430497 | 1.837225108 |
| hsa-miR-4476 | 5.19501084 | 1.780182219 | 1.687565624 | 3.158464108 | 1.725432622 | 1.845900924 |
| hsa-miR-4477a | 1.741654877 | 1.761352081 | 1.810389951 | 1.745253904 | 2.648326175 | 1.739797394 |
| hsa-miR-4477b | 1.891646188 | 1.706224986 | 1.885616279 | 1.823158803 | 1.863020632 | 1.733971966 |
| hsa-miR-4478 | 2.996263574 | 1.989083266 | 2.129293537 | 4.000702651 | 1.792276719 | 1.947014694 |
| hsa-miR-4479 | 1.648291361 | 1.772646741 | 1.670426743 | 2.02535425 | 1.789294183 | 1.716819053 |
| hsa-miR-448 | 1.70015159 | 1.855006089 | 1.82546924 | 1.636416794 | 1.878389729 | 1.844892928 |
| hsa-miR-4480 | 1.623079363 | 1.834683594 | 1.812111515 | 1.729694632 | 1.840800265 | 1.800044186 |
| hsa-miR-4481 | 2.75083065 | 2.136942845 | 1.765454655 | 2.665598303 | 2.025218373 | 1.791187357 |
| hsa-miR-4482-3p | 1.687621468 | 1.764678845 | 1.742859674 | 2.131285073 | 1.760484179 | 1.682504196 |
| hsa-miR-4482-5p | 1.786956933 | 1.657493695 | 1.810002932 | 1.782559061 | 1.722481215 | 1.710055729 |
| hsa-miR-4483 | 1.879764588 | 1.847837092 | 1.746755793 | 1.718649414 | 1.866793272 | 1.783352956 |
| hsa-miR-4484 | 5.879070144 | 5.378349136 | 5.443792981 | 9.164168933 | 2.546275884 | 6.266071341 |
| hsa-miR-4485-3p | 1.795554106 | 1.705075776 | 1.731833014 | 1.723299145 | 1.682984381 | 1.656310779 |
| hsa-miR-4485-5p | 5.528870796 | 4.852995941 | 5.281395308 | 4.230946592 | 2.331499018 | 5.401520244 |
| hsa-miR-4486 | 2.640992764 | 1.8194293 | 1.944551771 | 2.602772848 | 1.680957039 | 1.793475983 |
| hsa-miR-4487 | 2.720140044 | 1.916571282 | 1.716805622 | 2.726083027 | 1.719357794 | 1.747736749 |
| hsa-miR-4488 | 1.766712323 | 1.87228191 | 1.673325263 | 1.853367948 | 1.692845653 | 1.689384907 |
| hsa-miR-4489 | 1.711547759 | 1.910912536 | 1.75751594 | 1.753845303 | 1.832684873 | 1.748781837 |
| hsa-miR-4490 | 1.665975223 | 1.744338302 | 2.430536437 | 1.708969011 | 1.90018318 | 1.867943343 |
| hsa-miR-4491 | 1.670080404 | 1.741411661 | 1.679682255 | 1.619426981 | 2.190796589 | 1.809930883 |
| hsa-miR-4492 | 1.657435146 | 1.740469106 | 1.67920371 | 1.803223163 | 1.748665823 | 1.734318992 |
| hsa-miR-4493 | 1.8437308 | 1.748199369 | 1.788962771 | 1.717772887 | 1.946630308 | 1.703573079 |
| hsa-miR-4494 | 1.833337791 | 1.682743841 | 1.614263886 | 1.699947514 | 1.690039094 | 1.677511098 |
| hsa-miR-4495 | 1.716827639 | 2.170381253 | 1.893142832 | 1.708428544 | 2.036205395 | 1.815715212 |
| hsa-miR-4496 | 3.131324789 | 2.096776602 | 3.01675035 | 4.781914371 | 1.731473061 | 4.569602406 |
| hsa-miR-4497 | 5.241629736 | 4.811556812 | 1.880784166 | 5.951830983 | 1.703941291 | 1.977363016 |
| hsa-miR-4498 | 1.77876901 | 1.744332248 | 1.745224264 | 1.692141741 | 1.65528881 | 1.788378654 |
| hsa-miR-4499 | 7.113846086 | 4.93995969 | 6.943325079 | 8.93852283 | 2.0518218 | 7.808627892 |
| hsa-miR-449a | 1.768339519 | 1.717672201 | 1.850504734 | 6.789711435 | 2.075122742 | 5.735239735 |
| hsa-miR-449b-3p | 1.705042131 | 1.734679568 | 1.706570316 | 1.678322342 | 1.817016115 | 1.714832824 |
| hsa-miR-449b-5p | 1.772606972 | 1.746511899 | 1.867657774 | 1.869136678 | 1.771880625 | 1.83793044 |
| hsa-miR-449c-3p | 1.641212025 | 1.798860265 | 1.733286797 | 1.738384527 | 2.227550126 | 1.713187965 |
| hsa-miR-449c-5p | 1.728621458 | 1.652037218 | 1.66649004 | 1.719854079 | 1.689205286 | 1.662456497 |
| hsa-miR-4500 | 1.740096122 | 1.783254597 | 1.767329225 | 1.682930289 | 2.198714272 | 1.887075273 |
| hsa-miR-4501 | 1.616977089 | 1.737369822 | 1.727124224 | 1.646996622 | 1.877567002 | 1.744160567 |
| hsa-miR-4502 | 1.796236733 | 1.809684373 | 1.63745321 | 1.799258365 | 1.936862876 | 1.780391158 |
| hsa-miR-4503 | 1.865211758 | 1.729488419 | 1.716839908 | 1.707446086 | 1.745151706 | 1.929481636 |
| hsa-miR-4504 | 1.759401896 | 1.766838414 | 1.868119031 | 1.705680282 | 2.561250788 | 1.825207284 |
| hsa-miR-4505 | 7.616785545 | 6.262479885 | 4.46964013 | 6.190560529 | 1.848817734 | 4.948334349 |
| hsa-miR-4506 | 1.678287767 | 1.745126148 | 1.765436433 | 1.640859786 | 2.118338191 | 1.782093169 |
| hsa-miR-4507 | 6.670021089 | 5.635025784 | 4.673913491 | 6.529577764 | 1.636044829 | 2.469819134 |
| hsa-miR-4508 | 1.682705112 | 1.827457401 | 1.817679351 | 3.105511017 | 1.999444639 | 1.816850251 |
| hsa-miR-4509 | 1.858294792 | 1.655706273 | 2.008907975 | 1.757357638 | 2.149060124 | 1.903869367 |
| hsa-miR-450a-1-3p | 1.724511928 | 1.698462286 | 1.841970177 | 1.748018026 | 1.825793463 | 1.763011718 |
| hsa-miR-450a-2-3p | 1.679203984 | 1.803537882 | 2.088474202 | 1.756364836 | 2.424368387 | 2.004258123 |
| hsa-miR-450a-5p | 1.748392903 | 1.767967737 | 1.910563414 | 2.832220962 | 2.481430068 | 2.140725512 |
| hsa-miR-450b-3p | 1.753705712 | 1.924539585 | 1.896831402 | 1.737276657 | 1.895182776 | 1.663960007 |
| hsa-miR-450b-5p | 1.871370222 | 1.775153487 | 1.801780329 | 1.762452092 | 1.82082469 | 1.7302881 |
| hsa-miR-4510 | 1.738031654 | 2.048384023 | 1.841847368 | 1.750554451 | 2.022993321 | 1.842186673 |
| hsa-miR-4511 | 1.749671973 | 1.798969961 | 1.866420091 | 1.688883669 | 1.741222453 | 1.748785607 |
| hsa-miR-4512 | 1.754325685 | 1.954682965 | 1.796447597 | 1.698239527 | 1.939836965 | 1.811383883 |
| hsa-miR-4513 | 4.330741666 | 2.329910994 | 1.728339658 | 2.108436169 | 1.729081507 | 2.011802604 |
| hsa-miR-4514 | 4.859600975 | 1.843231718 | 1.959580934 | 2.493017374 | 1.71080357 | 5.071130046 |
| hsa-miR-4515 | 5.816095621 | 4.336324435 | 3.948854637 | 6.671962062 | 2.239726622 | 3.113645036 |
| hsa-miR-4516 | 10.41923364 | 11.45643564 | 10.88815817 | 8.443305467 | 2.112438776 | 7.085612855 |
| hsa-miR-4517 | 1.642507117 | 1.836833528 | 1.787668899 | 1.577183292 | 1.797152704 | 1.781614982 |
| hsa-miR-4518 | 1.699340223 | 2.000758252 | 1.834773432 | 1.639915029 | 1.958829701 | 1.797934224 |
| hsa-miR-4519 | 1.765385137 | 1.807649187 | 1.638729956 | 1.683845596 | 1.816221656 | 1.641552233 |
| hsa-miR-451a | 8.050124304 | 6.688714994 | 7.806425514 | 11.61348098 | 11.59268603 | 13.08498063 |
| hsa-miR-451b | 1.67608794 | 1.821120188 | 1.841510742 | 1.626416675 | 3.224512833 | 1.809291319 |
| hsa-miR-452-3p | 1.645993762 | 1.642854307 | 1.679394407 | 1.64284972 | 1.664598731 | 1.672923533 |
| hsa-miR-452-5p | 1.699320149 | 1.888929235 | 1.78615755 | 1.975765167 | 1.868856902 | 1.936495343 |
| hsa-miR-4520-2-3p | 1.673234519 | 1.72235825 | 1.739161767 | 1.751044366 | 1.876743251 | 1.704764386 |
| hsa-miR-4520-3p | 1.748027804 | 1.630218874 | 1.674158398 | 2.583763214 | 1.64464962 | 1.629173044 |
| hsa-miR-4520-5p | 1.753434051 | 1.712625594 | 1.691243067 | 1.709075316 | 1.696194404 | 1.868401045 |
| hsa-miR-4521 | 1.821973864 | 1.769050413 | 1.894100283 | 1.645114252 | 1.676774578 | 1.752591053 |
| hsa-miR-4522 | 1.861062236 | 1.723832332 | 1.688042223 | 1.683793698 | 1.624543125 | 1.647289163 |
| hsa-miR-4523 | 1.705176247 | 1.905014036 | 1.688476356 | 1.573262685 | 1.739697181 | 1.654476636 |
| hsa-miR-4524a-3p | 1.734721199 | 1.723822098 | 1.876057002 | 1.662541622 | 1.778180343 | 1.695021058 |
| hsa-miR-4524a-5p | 1.724862154 | 1.710876526 | 1.673011253 | 1.68720171 | 1.752448499 | 1.715750634 |
| hsa-miR-4524b-3p | 1.785626032 | 2.026956642 | 1.758609752 | 1.699068392 | 1.726160855 | 1.712790384 |
| hsa-miR-4524b-5p | 1.70490659 | 1.730582902 | 1.717789806 | 1.73497227 | 1.798676994 | 1.737622245 |
| hsa-miR-4525 | 1.687948041 | 1.907053698 | 1.763758504 | 1.612347192 | 2.008334872 | 1.823739947 |
| hsa-miR-4526 | 1.567746049 | 1.714007539 | 1.709973086 | 1.645161853 | 1.703976259 | 1.684396726 |
| hsa-miR-4527 | 1.649159508 | 1.677171009 | 1.691935532 | 1.663275832 | 1.839007224 | 1.74146354 |
| hsa-miR-4528 | 1.791376218 | 2.364034149 | 1.713242674 | 1.895900721 | 1.875918513 | 1.966065936 |
| hsa-miR-4529-3p | 1.662170886 | 1.790937435 | 1.730500563 | 1.651125236 | 1.757229997 | 1.758246788 |
| hsa-miR-4529-5p | 1.598549523 | 1.750993516 | 1.708171388 | 1.686136059 | 1.738027426 | 1.799679231 |
| hsa-miR-4530 | 10.47242229 | 8.445660299 | 5.469272799 | 4.94466908 | 1.852966594 | 2.406914282 |
| hsa-miR-4531 | 3.569325959 | 1.727018664 | 1.741900629 | 3.106109018 | 1.710202277 | 1.680571362 |
| hsa-miR-4532 | 4.265202503 | 5.407328878 | 2.092707899 | 6.432108872 | 1.73532807 | 1.892552744 |
| hsa-miR-4533 | 1.700478121 | 1.738465605 | 1.695128534 | 1.742516448 | 1.730040328 | 1.729460619 |
| hsa-miR-4534 | 5.886823501 | 4.067628267 | 4.437981555 | 8.622523595 | 1.828410276 | 4.374627106 |
| hsa-miR-4535 | 3.079464279 | 2.316532118 | 2.218321995 | 2.166491703 | 1.824426142 | 2.033482795 |
| hsa-miR-4536-3p | 1.757651859 | 1.885023693 | 1.766017806 | 1.668569619 | 2.412144679 | 2.159895538 |
| hsa-miR-4536-5p | 1.71386358 | 1.679412645 | 1.840013578 | 1.729549804 | 1.767175847 | 1.744023142 |
| hsa-miR-4537 | 1.887615822 | 1.718006204 | 1.708848649 | 1.674837888 | 1.739035241 | 1.648334006 |
| hsa-miR-4538 | 2.081428408 | 2.936471046 | 1.926487767 | 3.337927988 | 2.206018524 | 1.994598871 |
| hsa-miR-4539 | 1.885717365 | 2.055518389 | 1.88183857 | 2.807140487 | 1.88013246 | 1.906279851 |
| hsa-miR-454-3p | 2.282604895 | 1.749832409 | 1.745966334 | 1.78862316 | 1.871474376 | 1.706101047 |
| hsa-miR-454-5p | 1.697583034 | 1.791299902 | 1.81518517 | 1.704897804 | 2.422504093 | 1.928377631 |
| hsa-miR-4540 | 1.723260939 | 1.788358469 | 1.709014674 | 1.670081994 | 1.8252878 | 1.886939091 |
| hsa-miR-455-3p | 3.20106107 | 1.704080924 | 1.724152038 | 2.139531735 | 1.709877174 | 1.755574131 |
| hsa-miR-455-5p | 1.777177702 | 1.862399629 | 1.714836115 | 1.831156939 | 1.796563694 | 1.747531339 |
| hsa-miR-4632-3p | 1.59922457 | 1.759906637 | 1.817490064 | 1.614029645 | 2.11714392 | 1.771740848 |
| hsa-miR-4632-5p | 8.935317164 | 1.93537422 | 2.147077747 | 7.434367651 | 1.864304655 | 3.673742156 |
| hsa-miR-4633-3p | 1.72948287 | 1.742655971 | 1.70947297 | 1.605820634 | 1.792353268 | 1.738728821 |
| hsa-miR-4633-5p | 1.906630631 | 2.272430288 | 1.925290463 | 1.71245635 | 1.794573734 | 1.790676604 |
| hsa-miR-4634 | 6.280273135 | 6.720434537 | 3.112027346 | 4.873155807 | 1.77478045 | 1.741433745 |
| hsa-miR-4635 | 2.002880261 | 1.653984383 | 1.631130864 | 1.68584335 | 1.679627072 | 1.67720377 |
| hsa-miR-4636 | 1.725524106 | 1.687354905 | 1.80749818 | 1.694124062 | 2.06197128 | 1.760347517 |
| hsa-miR-4637 | 1.685082175 | 1.708588895 | 1.909618221 | 1.640675168 | 2.383280337 | 1.818494084 |
| hsa-miR-4638-3p | 1.689755647 | 1.679193206 | 1.73954432 | 1.797819312 | 1.693431622 | 1.661459617 |
| hsa-miR-4638-5p | 1.733856868 | 2.142439803 | 1.687110882 | 1.632172335 | 1.732023113 | 1.689173177 |
| hsa-miR-4639-3p | 1.823423538 | 1.914850612 | 1.644596554 | 1.730056498 | 1.867146278 | 1.730012604 |
| hsa-miR-4639-5p | 1.758230385 | 1.727034003 | 2.061837071 | 1.712106084 | 1.856330224 | 1.861710166 |
| hsa-miR-4640-3p | 1.799146799 | 1.729092528 | 1.925380611 | 1.773723401 | 1.617485233 | 1.633690355 |
| hsa-miR-4640-5p | 2.889121062 | 2.753991808 | 2.735132091 | 5.721091057 | 1.8341369 | 4.578043161 |
| hsa-miR-4641 | 1.789468854 | 1.793780166 | 1.721508928 | 1.670250582 | 2.849099726 | 1.764787216 |
| hsa-miR-4642 | 1.68903209 | 1.679098836 | 1.700352487 | 1.825617528 | 1.787202898 | 1.78642337 |
| hsa-miR-4643 | 1.701002003 | 1.728063913 | 1.929971966 | 1.673999728 | 1.919228466 | 1.796415767 |
| hsa-miR-4644 | 8.018025085 | 4.363193419 | 5.278213524 | 9.059734611 | 1.793943978 | 4.290145351 |
| hsa-miR-4645-3p | 1.730666497 | 2.215464665 | 1.794778054 | 1.602802687 | 1.7626987 | 1.774939848 |
| hsa-miR-4645-5p | 1.731781332 | 1.721962499 | 1.679911521 | 1.712588464 | 2.009936031 | 1.753160748 |
| hsa-miR-4646-3p | 2.399391486 | 2.210550667 | 2.23821847 | 2.147798304 | 2.135728412 | 2.128599615 |
| hsa-miR-4646-5p | 6.499529583 | 2.477530953 | 1.960111298 | 4.99604353 | 1.74572505 | 5.208421536 |
| hsa-miR-4647 | 1.817317501 | 1.748050502 | 1.684369161 | 1.819646784 | 1.750111975 | 1.751167012 |
| hsa-miR-4648 | 1.793940334 | 1.778477913 | 1.856223187 | 2.787402348 | 1.864208165 | 1.913462 |
| hsa-miR-4649-3p | 5.193116498 | 7.277379639 | 7.500974644 | 3.684306928 | 9.92792122 | 6.777026883 |
| hsa-miR-4649-5p | 1.675264023 | 1.745731342 | 1.737869397 | 1.667396231 | 1.814808544 | 1.838641408 |
| hsa-miR-4650-3p | 1.653360808 | 1.764625664 | 1.776961203 | 1.617773955 | 1.717784039 | 1.761134314 |
| hsa-miR-4650-5p | 1.729994813 | 1.909048507 | 1.768545933 | 1.648172468 | 1.928926105 | 1.759098575 |
| hsa-miR-4651 | 2.942558355 | 1.740538692 | 1.673782075 | 2.658137366 | 1.791804063 | 1.699279849 |
| hsa-miR-4652-3p | 4.151213949 | 4.380284741 | 6.220881941 | 2.804020722 | 4.825863875 | 5.676739166 |
| hsa-miR-4652-5p | 1.714321049 | 1.785225881 | 1.789541934 | 1.728585311 | 1.787384065 | 1.751152436 |
| hsa-miR-4653-3p | 2.107457253 | 2.128731678 | 1.87590504 | 3.543538733 | 1.685912562 | 1.83664816 |
| hsa-miR-4653-5p | 1.698184634 | 1.785005592 | 1.82833635 | 1.634857909 | 2.309020835 | 1.769424681 |
| hsa-miR-4654 | 1.73055625 | 1.746978945 | 1.6424051 | 1.664176223 | 1.653798085 | 1.64435035 |
| hsa-miR-4655-3p | 1.800130236 | 2.022321514 | 1.678957634 | 2.768970216 | 1.661780351 | 1.636728209 |
| hsa-miR-4655-5p | 4.899439403 | 4.308038959 | 4.967897684 | 3.620060407 | 1.888772897 | 4.770918217 |
| hsa-miR-4656 | 1.8591071 | 1.891500945 | 1.877816088 | 2.272936139 | 1.690821926 | 2.298373148 |
| hsa-miR-4657 | 1.783033543 | 1.949498005 | 1.78876721 | 1.757952163 | 1.74070258 | 1.691388555 |
| hsa-miR-4658 | 1.765914074 | 1.851440365 | 1.741874907 | 1.731233205 | 1.863041262 | 1.704771852 |
| hsa-miR-4659a-3p | 2.916814109 | 1.716534221 | 1.873446735 | 4.717874596 | 1.694757702 | 1.746206333 |
| hsa-miR-4659a-5p | 1.663886001 | 1.703110288 | 1.652789661 | 1.682784928 | 1.86583772 | 1.723074326 |
| hsa-miR-4659b-3p | 1.694660332 | 1.66019619 | 1.668361717 | 1.701021405 | 1.684505285 | 1.833987076 |
| hsa-miR-4659b-5p | 1.767101028 | 1.685296223 | 1.975960908 | 1.673987648 | 1.996081402 | 1.746664058 |
| hsa-miR-466 | 1.829029349 | 1.712704324 | 1.865204049 | 1.709666778 | 1.822609071 | 1.711090732 |
| hsa-miR-4660 | 2.031485279 | 2.222194193 | 1.829280337 | 2.043028611 | 1.867483856 | 1.747354525 |
| hsa-miR-4661-3p | 1.649903707 | 1.836783484 | 1.755361477 | 1.632248534 | 1.630564029 | 1.732927647 |
| hsa-miR-4661-5p | 1.580053264 | 1.808004389 | 1.840265069 | 1.640159481 | 1.737878101 | 1.772162056 |
| hsa-miR-4662a-3p | 1.644130831 | 1.977534892 | 2.086682145 | 1.680520185 | 2.46434888 | 1.828440592 |
| hsa-miR-4662a-5p | 1.743202233 | 1.679210812 | 1.703806755 | 1.674205678 | 1.887153378 | 1.80309752 |
| hsa-miR-4662b | 1.750008667 | 1.880247152 | 1.785547607 | 1.60562946 | 1.836180061 | 1.740474301 |
| hsa-miR-4663 | 1.608360173 | 1.774586977 | 1.79351389 | 1.590640145 | 1.909488723 | 1.807609223 |
| hsa-miR-4664-3p | 3.346852508 | 4.125760951 | 3.57325398 | 2.987121134 | 2.709623107 | 2.884831904 |
| hsa-miR-4664-5p | 1.679141239 | 1.762219075 | 1.803640788 | 1.764513433 | 1.72430084 | 1.829660408 |
| hsa-miR-4665-3p | 8.132761556 | 9.641039214 | 9.413683516 | 6.960026176 | 11.54165395 | 8.607899426 |
| hsa-miR-4665-5p | 1.844974073 | 1.751121293 | 1.789082785 | 2.146400804 | 1.735975277 | 1.795499703 |
| hsa-miR-4666a-3p | 1.834988106 | 1.867827737 | 1.77721911 | 1.751066763 | 1.940367001 | 1.918937271 |
| hsa-miR-4666a-5p | 1.763771059 | 1.984679843 | 1.787571194 | 1.718774816 | 2.017801058 | 1.766776642 |
| hsa-miR-4666b | 1.715452314 | 1.873788317 | 2.220595318 | 1.776649487 | 3.087198019 | 2.739219416 |
| hsa-miR-4667-3p | 1.902820134 | 2.059541728 | 2.021538865 | 1.694681778 | 1.770220115 | 1.787870793 |
| hsa-miR-4667-5p | 4.258035032 | 1.817637061 | 2.94233805 | 6.147671457 | 2.160218848 | 2.126800441 |
| hsa-miR-4668-3p | 1.946357 | 1.986172709 | 1.722903805 | 1.716688618 | 1.795398496 | 1.667077584 |
| hsa-miR-4668-5p | 1.786515064 | 1.93431053 | 1.850832126 | 1.795988655 | 2.092831994 | 1.79740185 |
| hsa-miR-4669 | 7.60335945 | 7.212416506 | 8.648004582 | 10.12735733 | 1.837033776 | 8.186512576 |
| hsa-miR-4670-3p | 1.805062875 | 1.865200099 | 2.004240435 | 1.656924669 | 1.766687717 | 1.659527063 |
| hsa-miR-4670-5p | 1.737171325 | 1.780522859 | 1.806333129 | 1.72186357 | 1.826793327 | 1.840474657 |
| hsa-miR-4671-3p | 1.797160647 | 1.761753916 | 1.786952173 | 1.717675577 | 2.466580361 | 1.784169298 |
| hsa-miR-4671-5p | 1.716795771 | 1.695508059 | 2.317429282 | 1.688874783 | 1.869793215 | 1.735819906 |
| hsa-miR-4672 | 3.954797819 | 1.819086657 | 1.762448814 | 3.479802153 | 1.72182902 | 1.736813331 |
| hsa-miR-4673 | 1.707465823 | 1.816645393 | 1.841346995 | 2.94004017 | 1.774428231 | 1.920091429 |
| hsa-miR-4674 | 1.700233163 | 1.682658831 | 1.692193638 | 1.652167798 | 1.644400219 | 1.596893648 |
| hsa-miR-4675 | 1.748750002 | 1.787826836 | 1.892034799 | 2.633990784 | 1.84557339 | 1.756482247 |
| hsa-miR-4676-3p | 1.788277844 | 1.826825306 | 1.898238982 | 1.778283847 | 1.782834298 | 1.781793609 |
| hsa-miR-4676-5p | 1.631620946 | 1.742345759 | 1.802152519 | 1.793583578 | 1.812089332 | 1.826046609 |
| hsa-miR-4677-3p | 1.700157199 | 1.893611004 | 1.686039909 | 1.662153424 | 2.042599301 | 1.909477555 |
| hsa-miR-4677-5p | 1.658770736 | 1.795114604 | 1.674201191 | 1.652115933 | 1.758335254 | 1.609618289 |
| hsa-miR-4678 | 1.748621791 | 1.944097152 | 2.444967399 | 1.71960835 | 2.581893706 | 2.07528623 |
| hsa-miR-4679 | 2.010710175 | 1.790627236 | 1.733217302 | 1.697982386 | 3.049100649 | 1.809884807 |
| hsa-miR-4680-3p | 1.86569392 | 1.762184634 | 1.733372268 | 1.722188622 | 1.912204041 | 1.683673135 |
| hsa-miR-4680-5p | 1.650617663 | 1.837729594 | 2.272956565 | 1.734358433 | 1.899043271 | 1.793541516 |
| hsa-miR-4681 | 1.705546894 | 2.047871949 | 1.856318749 | 1.737917867 | 2.020190353 | 1.738659595 |
| hsa-miR-4682 | 1.713265578 | 1.783085803 | 1.841138434 | 1.691717341 | 1.749282405 | 1.68722318 |
| hsa-miR-4683 | 1.64673414 | 1.839774972 | 1.769326013 | 1.630086579 | 1.761539838 | 1.700256724 |
| hsa-miR-4684-3p | 2.14714355 | 1.716317336 | 1.67517492 | 1.858621193 | 1.690482743 | 1.677682862 |
| hsa-miR-4684-5p | 1.667352251 | 1.833011372 | 1.815992172 | 1.678791826 | 1.838096568 | 1.992906544 |
| hsa-miR-4685-3p | 1.653570679 | 1.833428492 | 1.859272372 | 1.705966945 | 1.816872824 | 1.803686115 |
| hsa-miR-4685-5p | 2.846860826 | 2.03112379 | 3.083885114 | 3.435368476 | 2.00572531 | 2.204093617 |
| hsa-miR-4686 | 1.776530563 | 1.716471463 | 1.739602448 | 1.755432959 | 1.843555481 | 1.769496927 |
| hsa-miR-4687-3p | 8.696465929 | 9.052354874 | 7.230131103 | 8.11486438 | 1.736984665 | 6.525949084 |
| hsa-miR-4687-5p | 1.850314899 | 2.240275736 | 1.834193657 | 1.852576018 | 1.810330302 | 2.04755743 |
| hsa-miR-4688 | 3.668550842 | 2.888486187 | 2.270416176 | 4.403102707 | 1.802530914 | 3.90080465 |
| hsa-miR-4689 | 2.275174473 | 1.956172502 | 1.825452649 | 2.537246327 | 1.761719958 | 1.982219092 |
| hsa-miR-4690-3p | 1.65068368 | 1.721102803 | 1.714830502 | 1.593145196 | 2.137193496 | 1.756362265 |
| hsa-miR-4690-5p | 4.513273036 | 1.757353853 | 1.929897122 | 4.23305061 | 1.728642527 | 1.675410679 |
| hsa-miR-4691-3p | 1.739456537 | 1.796758149 | 1.697283493 | 1.759127924 | 1.783519298 | 1.633395591 |
| hsa-miR-4691-5p | 1.60162291 | 1.756892472 | 1.781598945 | 1.718800518 | 1.812330831 | 1.666390546 |
| hsa-miR-4692 | 1.767853415 | 1.648135959 | 1.779682324 | 1.8025735 | 1.695760221 | 1.637441175 |
| hsa-miR-4693-3p | 1.784045583 | 1.768314636 | 1.795508327 | 1.651867894 | 1.789934449 | 1.830632258 |
| hsa-miR-4693-5p | 1.833801195 | 1.686121533 | 1.871794347 | 1.69101675 | 1.906319177 | 1.741988946 |
| hsa-miR-4694-3p | 1.7019612 | 1.922652712 | 1.834270626 | 1.694876432 | 1.796682959 | 1.909859688 |
| hsa-miR-4694-5p | 1.707931804 | 2.780736577 | 1.833094255 | 1.738610588 | 2.959981138 | 1.834107214 |
| hsa-miR-4695-3p | 1.668159404 | 1.95488218 | 1.863693592 | 1.655622683 | 1.714295486 | 1.765361145 |
| hsa-miR-4695-5p | 2.690219237 | 2.160230155 | 2.212786557 | 4.246218865 | 1.640477696 | 2.175356964 |
| hsa-miR-4696 | 1.677860548 | 1.779323011 | 1.789143301 | 1.68392709 | 2.052029544 | 1.839147242 |
| hsa-miR-4697-3p | 1.767159641 | 2.056730359 | 1.835359856 | 1.935331371 | 2.111490206 | 1.952405817 |
| hsa-miR-4697-5p | 1.720637741 | 1.716533927 | 1.692578921 | 1.65775892 | 1.773693179 | 1.636527507 |
| hsa-miR-4698 | 4.582823156 | 3.542134416 | 2.034848957 | 9.287332433 | 2.260826289 | 2.999403164 |
| hsa-miR-4699-3p | 1.727418814 | 1.750291575 | 1.804188093 | 1.750620049 | 1.873860048 | 1.731531336 |
| hsa-miR-4699-5p | 1.697617755 | 1.669337849 | 1.786864643 | 1.621150616 | 1.778224741 | 1.640947412 |
| hsa-miR-4700-3p | 1.983679584 | 1.915258253 | 2.238343722 | 1.966610489 | 1.810434873 | 2.153068938 |
| hsa-miR-4700-5p | 1.723702899 | 1.875156338 | 1.798465006 | 1.71211158 | 1.777683526 | 1.795487979 |
| hsa-miR-4701-3p | 3.980799739 | 2.108531443 | 2.762689835 | 4.828293932 | 1.906851113 | 1.715855425 |
| hsa-miR-4701-5p | 3.097837129 | 2.976088588 | 2.26273286 | 2.47816108 | 2.689457851 | 2.825166894 |
| hsa-miR-4703-3p | 1.914577622 | 1.789517775 | 1.752144608 | 1.787471803 | 2.596508503 | 1.708446681 |
| hsa-miR-4703-5p | 1.887514387 | 1.684304397 | 1.771273521 | 1.936756388 | 1.716207117 | 1.690933371 |
| hsa-miR-4704-3p | 1.791034543 | 2.025036636 | 1.973590117 | 1.775231876 | 1.79060659 | 1.763487167 |
| hsa-miR-4704-5p | 1.709097829 | 1.748305726 | 1.75381726 | 1.794464625 | 1.959795837 | 1.701125805 |
| hsa-miR-4705 | 1.934340519 | 1.78537042 | 1.780420122 | 1.735398391 | 1.808442828 | 1.877208541 |
| hsa-miR-4706 | 2.074044594 | 1.822469477 | 1.903070595 | 1.861145326 | 1.745207671 | 2.088519755 |
| hsa-miR-4707-3p | 1.859476859 | 1.833808062 | 1.749611704 | 1.697065666 | 1.79504601 | 1.772977058 |
| hsa-miR-4707-5p | 1.772028957 | 2.002536706 | 1.898840145 | 5.375747154 | 1.910342024 | 1.861672925 |
| hsa-miR-4708-3p | 1.695420506 | 1.672042389 | 1.805416391 | 1.640003395 | 1.74551038 | 1.750272495 |
| hsa-miR-4708-5p | 1.760877442 | 1.863993591 | 1.793217598 | 1.674736675 | 1.762835692 | 1.642106132 |
| hsa-miR-4709-3p | 1.626450631 | 1.763730346 | 1.821400692 | 1.641371915 | 1.749287488 | 1.729844724 |
| hsa-miR-4709-5p | 1.743056136 | 2.203472006 | 1.694919942 | 1.702945289 | 1.690885103 | 1.767721779 |
| hsa-miR-4710 | 2.9135398 | 2.369034924 | 2.12654181 | 4.433920979 | 1.777127323 | 1.78855609 |
| hsa-miR-4711-3p | 1.682990706 | 2.090335816 | 1.866521501 | 1.688105319 | 1.80592573 | 1.805499837 |
| hsa-miR-4711-5p | 1.606017951 | 1.664667248 | 1.697915998 | 1.741215103 | 1.695262906 | 1.765412995 |
| hsa-miR-4712-3p | 1.872545255 | 1.668638705 | 1.763188275 | 1.786817717 | 1.679895407 | 1.640268328 |
| hsa-miR-4712-5p | 1.754610097 | 1.739193873 | 1.993370307 | 1.71005081 | 3.232254486 | 2.001573557 |
| hsa-miR-4713-3p | 4.15887947 | 3.325392443 | 3.737134905 | 3.809352716 | 1.834093728 | 6.571296588 |
| hsa-miR-4713-5p | 1.853541697 | 1.807749268 | 1.83552765 | 1.714817316 | 1.721589121 | 1.686240168 |
| hsa-miR-4714-3p | 1.862502022 | 1.808102875 | 1.635047433 | 1.813683595 | 3.221049206 | 2.03795267 |
| hsa-miR-4714-5p | 1.84865046 | 1.887291594 | 2.091799697 | 1.963995182 | 2.842926651 | 1.920847153 |
| hsa-miR-4715-3p | 1.66443233 | 1.753353632 | 1.747020313 | 1.620685851 | 1.861348791 | 1.768367017 |
| hsa-miR-4715-5p | 1.732091606 | 1.682448157 | 1.715150324 | 1.790699847 | 1.808772814 | 1.787094911 |
| hsa-miR-4716-3p | 4.073022384 | 1.819439548 | 2.229882855 | 6.139718885 | 1.660571009 | 1.877327404 |
| hsa-miR-4716-5p | 2.098759573 | 3.794347794 | 2.840589243 | 2.18954392 | 2.868957649 | 2.598338794 |
| hsa-miR-4717-3p | 1.730728013 | 1.829178994 | 1.795321395 | 1.813043974 | 1.873813755 | 1.710605344 |
| hsa-miR-4717-5p | 1.629311186 | 1.775286263 | 1.642975583 | 1.66516258 | 1.734262899 | 1.767149917 |
| hsa-miR-4718 | 1.809718475 | 1.666282894 | 1.754894284 | 1.753233305 | 1.62873189 | 1.719374801 |
| hsa-miR-4719 | 3.660741069 | 1.921047923 | 2.077178073 | 4.568129207 | 2.22187526 | 1.847140649 |
| hsa-miR-4720-3p | 1.63133098 | 1.831569298 | 1.846346812 | 1.627965133 | 2.158155971 | 2.287174804 |
| hsa-miR-4720-5p | 1.686791318 | 1.748006143 | 1.957521019 | 1.718445985 | 2.916965956 | 1.862634933 |
| hsa-miR-4721 | 4.552937367 | 2.757757933 | 2.477703269 | 4.508410565 | 1.798875019 | 2.573684504 |
| hsa-miR-4722-3p | 1.6870948 | 1.714808542 | 1.764114133 | 1.66106119 | 1.73692784 | 1.715595219 |
| hsa-miR-4722-5p | 1.612982165 | 1.751046886 | 1.724015927 | 1.620993905 | 1.986256246 | 1.765304474 |
| hsa-miR-4723-3p | 2.426401441 | 2.264821329 | 3.332546762 | 2.283623955 | 4.878532648 | 2.663770989 |
| hsa-miR-4723-5p | 1.668815422 | 1.656183158 | 1.646182481 | 1.66674796 | 1.759136705 | 1.655814048 |
| hsa-miR-4724-3p | 1.738002444 | 1.915903292 | 1.936005005 | 1.673619637 | 2.704857241 | 1.835302018 |
| hsa-miR-4724-5p | 1.580339792 | 1.750962136 | 1.598923509 | 1.581986499 | 1.761424672 | 1.702931019 |
| hsa-miR-4725-3p | 3.128993646 | 2.061341852 | 3.061945355 | 5.168380486 | 1.940971015 | 2.585197752 |
| hsa-miR-4725-5p | 5.263137967 | 7.483448269 | 7.171184851 | 3.609142824 | 9.817366271 | 6.796629821 |
| hsa-miR-4726-3p | 1.665882248 | 1.766525224 | 1.740943962 | 1.656156814 | 1.693945648 | 1.755697728 |
| hsa-miR-4726-5p | 1.934399903 | 1.758217949 | 1.792636715 | 2.977034583 | 1.729445665 | 1.696346661 |
| hsa-miR-4727-3p | 3.858957794 | 1.779140994 | 1.965420439 | 1.669016912 | 1.724361461 | 3.426378365 |
| hsa-miR-4727-5p | 1.654801293 | 1.798890739 | 1.7599992 | 1.585551394 | 1.841385699 | 1.699984966 |
| hsa-miR-4728-3p | 3.322395407 | 3.159415635 | 2.976543052 | 2.712705213 | 2.484321953 | 2.084554847 |
| hsa-miR-4728-5p | 4.038327102 | 2.488325561 | 3.598425637 | 3.91325814 | 1.85533574 | 2.943126708 |
| hsa-miR-4729 | 2.424330002 | 2.325764078 | 1.910793658 | 1.792631284 | 2.148993723 | 1.99413995 |
| hsa-miR-4730 | 1.783387698 | 1.905769642 | 1.714417964 | 1.678876159 | 2.022774564 | 1.721611654 |
| hsa-miR-4731-3p | 3.075471314 | 2.299772259 | 2.77900479 | 2.53587795 | 2.897079325 | 2.869661606 |
| hsa-miR-4731-5p | 1.670620002 | 1.731047959 | 1.668476067 | 1.635363846 | 1.775589402 | 1.741812243 |
| hsa-miR-4732-3p | 1.70589349 | 1.846836639 | 2.059543942 | 1.687144846 | 2.626975774 | 1.820503351 |
| hsa-miR-4732-5p | 3.714908076 | 5.53884767 | 4.372035055 | 2.431781987 | 2.101387519 | 1.90453657 |
| hsa-miR-4733-3p | 1.696933721 | 1.790859209 | 1.822894127 | 1.67581148 | 1.807523061 | 1.770374213 |
| hsa-miR-4733-5p | 1.718807881 | 1.72446633 | 1.616111186 | 1.657552714 | 1.783331018 | 1.697121074 |
| hsa-miR-4734 | 1.840586196 | 1.678609598 | 1.688851311 | 3.532235364 | 1.699800727 | 1.735549311 |
| hsa-miR-4735-3p | 1.709111612 | 1.858853769 | 1.759896835 | 1.684496991 | 1.875971776 | 1.790664918 |
| hsa-miR-4735-5p | 1.626000858 | 1.65619729 | 1.70364918 | 1.648146595 | 1.664096053 | 1.584329648 |
| hsa-miR-4736 | 1.91079177 | 1.750768746 | 1.915616843 | 1.667888023 | 1.815537325 | 1.817700581 |
| hsa-miR-4737 | 1.712903269 | 1.823119922 | 1.814992065 | 1.685419629 | 1.809749217 | 1.755724521 |
| hsa-miR-4738-3p | 2.133347555 | 1.945353201 | 1.951921367 | 2.198529897 | 1.818390962 | 2.672645636 |
| hsa-miR-4738-5p | 1.652375425 | 2.075677866 | 1.747269396 | 1.643751754 | 1.722041447 | 1.79719339 |
| hsa-miR-4739 | 3.597502556 | 2.168819335 | 1.791845525 | 4.73940061 | 1.788366225 | 2.403062685 |
| hsa-miR-4740-3p | 1.769359782 | 1.993619573 | 1.791468494 | 1.792697029 | 1.807476269 | 1.758502438 |
| hsa-miR-4740-5p | 2.150113741 | 1.987011629 | 1.777613009 | 3.312260642 | 1.707327701 | 1.822682495 |
| hsa-miR-4741 | 6.689404309 | 3.376436438 | 3.137905328 | 6.571557464 | 1.944900217 | 3.262250531 |
| hsa-miR-4742-3p | 1.630268847 | 1.808984346 | 1.761187408 | 1.695359082 | 2.465716871 | 1.72498558 |
| hsa-miR-4742-5p | 1.695604367 | 1.672824779 | 1.769997007 | 1.6080331 | 1.965590485 | 1.714546951 |
| hsa-miR-4743-3p | 1.80694354 | 1.632831924 | 1.836334013 | 1.627658359 | 1.787115468 | 1.70017685 |
| hsa-miR-4743-5p | 2.045113337 | 1.860987079 | 1.813307112 | 3.974177916 | 1.685747285 | 1.681736037 |
| hsa-miR-4744 | 1.676367512 | 1.807557291 | 1.716057008 | 1.646290676 | 1.711826457 | 1.80317751 |
| hsa-miR-4745-3p | 1.732587977 | 1.78845269 | 1.73340436 | 1.685260332 | 1.70948145 | 1.75399072 |
| hsa-miR-4745-5p | 5.954596483 | 3.265838419 | 1.884005108 | 4.67178767 | 1.836827244 | 1.673581718 |
| hsa-miR-4746-3p | 2.292268419 | 1.93279556 | 1.855769524 | 4.758756388 | 1.684984541 | 1.865304108 |
| hsa-miR-4746-5p | 1.752635445 | 1.738355795 | 1.866074967 | 1.707168065 | 1.692851312 | 1.678984289 |
| hsa-miR-4747-3p | 1.699797313 | 1.752508953 | 1.761651703 | 1.766355195 | 1.700586403 | 1.743136447 |
| hsa-miR-4747-5p | 1.918733798 | 1.671832664 | 1.698843971 | 1.683663218 | 1.688802762 | 1.63621354 |
| hsa-miR-4748 | 2.280972381 | 1.968716029 | 2.033316499 | 2.327814772 | 1.775663367 | 1.71286104 |
| hsa-miR-4749-3p | 4.705855099 | 5.822896624 | 6.085622437 | 3.228197185 | 7.312469876 | 5.359635629 |
| hsa-miR-4749-5p | 1.804732393 | 1.790817797 | 1.782151592 | 2.331139309 | 1.776259235 | 1.930858033 |
| hsa-miR-4750-3p | 2.906261584 | 3.858989758 | 3.933619409 | 3.027250909 | 4.73876949 | 3.804289165 |
| hsa-miR-4750-5p | 1.898603678 | 2.397645826 | 1.960864859 | 2.718056283 | 1.842415644 | 1.972166783 |
| hsa-miR-4751 | 1.705267657 | 1.728890321 | 1.639401615 | 1.756859775 | 1.775835472 | 1.710965389 |
| hsa-miR-4752 | 1.776248377 | 1.77007819 | 1.71293534 | 1.650947943 | 1.689443369 | 1.669730737 |
| hsa-miR-4753-3p | 1.774743768 | 1.813064962 | 1.809908936 | 1.674020036 | 1.832332284 | 1.817819898 |
| hsa-miR-4753-5p | 5.531619796 | 2.593028641 | 2.729470385 | 4.663254017 | 1.801857492 | 1.825526477 |
| hsa-miR-4754 | 1.705454038 | 1.681829334 | 1.706690445 | 5.544197386 | 1.675132516 | 1.815716713 |
| hsa-miR-4755-3p | 2.293163568 | 1.70216776 | 1.838594219 | 3.374426279 | 1.793546865 | 1.988895263 |
| hsa-miR-4755-5p | 1.735092352 | 1.664222823 | 1.763327816 | 1.675853937 | 1.828885488 | 1.643705526 |
| hsa-miR-4756-3p | 1.737151576 | 1.821473308 | 1.698314816 | 1.758966727 | 1.822856996 | 1.774304004 |
| hsa-miR-4756-5p | 1.680693414 | 1.68684767 | 1.905639502 | 1.640748646 | 1.943028998 | 1.779946562 |
| hsa-miR-4757-3p | 1.727419054 | 1.772392992 | 1.875052773 | 1.726518514 | 1.808714867 | 1.685829446 |
| hsa-miR-4757-5p | 1.673146676 | 1.737068395 | 1.686645814 | 1.689315202 | 1.733117729 | 1.645507647 |
| hsa-miR-4758-3p | 3.360754189 | 3.638417397 | 3.164974258 | 2.436011766 | 2.898882058 | 3.187357989 |
| hsa-miR-4758-5p | 2.858790446 | 1.787910307 | 1.68151452 | 4.198028647 | 1.697225594 | 1.692008056 |
| hsa-miR-4759 | 1.835156212 | 1.754255418 | 1.703616395 | 1.744774437 | 1.746378998 | 1.69421423 |
| hsa-miR-4760-3p | 1.812630656 | 1.725849529 | 1.765357521 | 1.751636018 | 1.764517768 | 1.889231978 |
| hsa-miR-4760-5p | 1.794626413 | 1.87620872 | 1.705482416 | 1.74894037 | 2.005456102 | 1.72087016 |
| hsa-miR-4761-3p | 1.681501022 | 1.845490455 | 1.713585343 | 1.641549002 | 1.794231729 | 1.723076643 |
| hsa-miR-4761-5p | 1.72046468 | 1.716864163 | 1.77083734 | 1.599829197 | 1.711491428 | 1.688767355 |
| hsa-miR-4762-3p | 1.825562006 | 1.980776607 | 1.801498915 | 1.728553819 | 2.080669056 | 1.731929581 |
| hsa-miR-4762-5p | 1.677630728 | 1.951445762 | 1.745814197 | 1.65576066 | 1.868058126 | 1.818943438 |
| hsa-miR-4763-3p | 8.219676516 | 8.584656844 | 7.820116885 | 8.468258318 | 7.25240101 | 6.651601917 |
| hsa-miR-4763-5p | 1.951067695 | 1.942203778 | 1.902355199 | 1.827639959 | 1.704811139 | 1.739963928 |
| hsa-miR-4764-3p | 1.633189952 | 1.900475255 | 1.688503361 | 1.670347698 | 1.72780285 | 1.778771658 |
| hsa-miR-4764-5p | 1.786705519 | 1.739216828 | 1.890604167 | 3.237606193 | 1.840810554 | 1.822449723 |
| hsa-miR-4765 | 1.637820545 | 1.805947183 | 1.914200501 | 1.751005646 | 2.71298117 | 1.762224265 |
| hsa-miR-4766-3p | 1.754353519 | 1.71812632 | 1.806338089 | 1.677045285 | 1.724351096 | 1.811054844 |
| hsa-miR-4766-5p | 1.778661361 | 1.864179173 | 1.817841776 | 1.738331052 | 2.179684149 | 1.79539147 |
| hsa-miR-4767 | 6.536159598 | 5.271335421 | 3.873252006 | 6.52735074 | 11.22186637 | 4.000372051 |
| hsa-miR-4768-3p | 1.849822207 | 1.961605134 | 1.892460352 | 3.697878875 | 1.839832689 | 1.787070807 |
| hsa-miR-4768-5p | 1.614152843 | 1.744594087 | 1.909212402 | 1.642178432 | 1.933902214 | 1.997840591 |
| hsa-miR-4769-3p | 6.993865079 | 6.390785927 | 5.403218423 | 3.029392905 | 7.825628669 | 6.295834193 |
| hsa-miR-4769-5p | 2.732148662 | 1.711111088 | 1.787836419 | 5.688788853 | 1.700902198 | 1.704913171 |
| hsa-miR-4770 | 1.668030991 | 1.965220522 | 1.859812369 | 1.758768518 | 1.899314591 | 2.265210729 |
| hsa-miR-4771 | 1.654016202 | 1.858381452 | 1.758398696 | 1.749848212 | 1.838966347 | 1.813923842 |
| hsa-miR-4772-3p | 1.801165564 | 1.833912499 | 1.81426362 | 1.640255348 | 2.023554337 | 1.708920648 |
| hsa-miR-4772-5p | 1.566186697 | 1.802442448 | 2.018616901 | 1.549562888 | 1.980973937 | 1.968404612 |
| hsa-miR-4773 | 1.713157944 | 1.702942485 | 1.770437493 | 1.651884845 | 1.730080956 | 1.792705823 |
| hsa-miR-4774-3p | 1.74048274 | 1.8264075 | 1.754361743 | 1.620214842 | 1.640538456 | 1.677025236 |
| hsa-miR-4774-5p | 1.886365488 | 1.871549549 | 1.82648088 | 1.672429317 | 2.454403987 | 1.731446224 |
| hsa-miR-4775 | 1.788984098 | 1.916103483 | 1.717971214 | 1.728531685 | 1.84219639 | 1.94423359 |
| hsa-miR-4776-3p | 1.785295848 | 1.784726248 | 1.710679033 | 1.712064004 | 1.811589123 | 1.691646794 |
| hsa-miR-4776-5p | 9.605324687 | 1.780137066 | 8.660158005 | 5.173086043 | 1.717106468 | 1.703668215 |
| hsa-miR-4777-3p | 1.780688719 | 1.778935436 | 1.745667669 | 1.663494446 | 1.832109381 | 1.913995303 |
| hsa-miR-4777-5p | 1.767455555 | 1.778419753 | 2.050599768 | 1.762618772 | 2.038854906 | 1.961924955 |
| hsa-miR-4778-3p | 1.69872078 | 1.791768657 | 1.693370256 | 1.666057957 | 1.748534184 | 1.735170085 |
| hsa-miR-4778-5p | 8.654792103 | 7.754376698 | 9.423026915 | 10.20610766 | 1.852750665 | 7.50515431 |
| hsa-miR-4779 | 1.697553637 | 1.753604145 | 1.721965312 | 1.667172061 | 1.968400664 | 1.885272023 |
| hsa-miR-4780 | 1.801913255 | 1.74949616 | 1.77327385 | 1.774859112 | 1.704396067 | 1.63860512 |
| hsa-miR-4781-3p | 1.697717889 | 1.879890943 | 1.831721885 | 1.652879319 | 1.853188968 | 1.814998066 |
| hsa-miR-4781-5p | 1.783126979 | 1.615602833 | 1.611615549 | 1.712324279 | 1.682233155 | 1.645051325 |
| hsa-miR-4782-3p | 1.893386498 | 1.789781387 | 1.690073669 | 1.796408563 | 2.061797211 | 1.811370365 |
| hsa-miR-4782-5p | 1.642786554 | 1.717809342 | 1.787880871 | 1.682143134 | 1.879424776 | 1.897666347 |
| hsa-miR-4783-3p | 1.725975386 | 1.744674381 | 1.918085934 | 1.654215353 | 1.769429911 | 1.801923839 |
| hsa-miR-4783-5p | 1.750236374 | 1.746879277 | 1.822232582 | 1.593834125 | 1.81098202 | 1.805677484 |
| hsa-miR-4784 | 1.817721825 | 1.720342899 | 2.104952776 | 1.727900853 | 1.753371611 | 1.664966677 |
| hsa-miR-4785 | 3.275198393 | 2.071469979 | 1.873108578 | 3.644771219 | 2.062058274 | 2.010957145 |
| hsa-miR-4786-3p | 1.772821122 | 1.761050543 | 1.627292698 | 1.791627595 | 1.739087358 | 1.715527862 |
| hsa-miR-4786-5p | 1.69463121 | 1.66081566 | 1.718192399 | 1.555148065 | 1.872704825 | 1.747105766 |
| hsa-miR-4787-3p | 5.054883314 | 3.638556485 | 5.018034229 | 4.372051642 | 2.452799183 | 3.162200855 |
| hsa-miR-4787-5p | 5.848452558 | 3.899637073 | 2.278933248 | 2.90147283 | 2.281189787 | 2.146311896 |
| hsa-miR-4788 | 5.071625541 | 6.180750453 | 7.476351284 | 6.894344342 | 2.325220333 | 10.85310546 |
| hsa-miR-4789-3p | 1.742304865 | 1.836505762 | 1.976006025 | 1.798902822 | 2.225726244 | 1.92413413 |
| hsa-miR-4789-5p | 1.633452091 | 1.723841708 | 1.72681602 | 1.915559298 | 2.105354758 | 1.721091295 |
| hsa-miR-4790-3p | 1.748415399 | 1.660197163 | 1.648988436 | 1.668960156 | 1.682784862 | 1.707970433 |
| hsa-miR-4790-5p | 1.793634612 | 1.813551266 | 2.004696148 | 1.661248103 | 2.681450112 | 1.940969727 |
| hsa-miR-4791 | 1.688749724 | 1.795108029 | 1.942580791 | 1.647026347 | 2.285154166 | 1.804678534 |
| hsa-miR-4792 | 1.580071264 | 1.950192722 | 1.869089502 | 3.106255322 | 1.796976474 | 1.737494823 |
| hsa-miR-4793-3p | 1.710892145 | 1.913595308 | 2.267871763 | 1.666433621 | 1.74211307 | 1.750677584 |
| hsa-miR-4793-5p | 6.205617177 | 2.643914763 | 2.457370221 | 5.698238128 | 1.684660144 | 1.76573729 |
| hsa-miR-4794 | 1.698291239 | 1.753812811 | 1.763407772 | 1.656983208 | 1.749339371 | 1.730599255 |
| hsa-miR-4795-3p | 1.756472402 | 1.687057592 | 1.77573664 | 1.656263121 | 1.98372721 | 1.657089438 |
| hsa-miR-4795-5p | 1.727430708 | 1.980470059 | 2.216787735 | 1.724786752 | 3.513904393 | 2.363921916 |
| hsa-miR-4796-3p | 1.867344823 | 1.862634142 | 1.725143449 | 1.625003259 | 2.066573583 | 1.736299355 |
| hsa-miR-4796-5p | 2.074643955 | 1.731559161 | 2.014130208 | 1.808551693 | 3.264877947 | 1.889963317 |
| hsa-miR-4797-3p | 1.770468028 | 1.647773535 | 1.761169754 | 1.698762964 | 1.776548529 | 1.735497413 |
| hsa-miR-4797-5p | 1.71008098 | 1.731631378 | 1.852003079 | 1.615686045 | 1.701500171 | 1.704510664 |
| hsa-miR-4798-3p | 1.746306443 | 1.686778416 | 1.765022279 | 1.63020368 | 1.744443615 | 1.709431125 |
| hsa-miR-4798-5p | 1.815115394 | 1.64782534 | 1.805003402 | 1.870467345 | 1.925756295 | 1.841642337 |
| hsa-miR-4799-3p | 1.867187792 | 1.808295824 | 1.819602334 | 1.7189377 | 1.804892777 | 1.732541647 |
| hsa-miR-4799-5p | 1.793867548 | 1.873364946 | 1.982524918 | 1.586529787 | 1.749885178 | 1.791975567 |
| hsa-miR-4800-3p | 1.728236294 | 1.794271791 | 1.69764917 | 1.686151677 | 1.719560779 | 1.69824241 |
| hsa-miR-4800-5p | 8.929292379 | 8.785834241 | 9.779426363 | 11.34647169 | 2.26294085 | 8.479476926 |
| hsa-miR-4801 | 1.746185691 | 1.646147002 | 1.731370392 | 1.68216089 | 1.659383468 | 1.638805635 |
| hsa-miR-4802-3p | 1.661763203 | 1.650231109 | 1.671999174 | 1.735258877 | 1.739080473 | 1.61767936 |
| hsa-miR-4802-5p | 1.679234508 | 1.796679295 | 1.761860371 | 1.641654854 | 1.782674356 | 1.84173556 |
| hsa-miR-4803 | 1.762429505 | 1.842357723 | 1.705169355 | 1.6735646 | 3.206774443 | 2.595638815 |
| hsa-miR-4804-3p | 1.681491911 | 1.695299398 | 1.654184981 | 1.722496123 | 1.670183309 | 1.667556583 |
| hsa-miR-4804-5p | 1.755088344 | 1.697640503 | 1.631328468 | 1.634417321 | 1.709730446 | 1.655504529 |
| hsa-miR-483-3p | 1.934660952 | 2.221550172 | 2.045761593 | 1.67739454 | 2.513605714 | 2.404350706 |
| hsa-miR-483-5p | 9.802648159 | 9.465717183 | 10.5069347 | 10.66444572 | 1.998682084 | 7.47710684 |
| hsa-miR-484 | 3.856910138 | 3.86975267 | 3.402371874 | 3.525231048 | 3.745424658 | 4.851051601 |
| hsa-miR-485-3p | 1.653996108 | 1.948254033 | 2.304970502 | 1.649006641 | 2.915461193 | 2.03529903 |
| hsa-miR-485-5p | 1.692838686 | 1.691493069 | 1.808152493 | 1.605455405 | 1.786829382 | 1.82686594 |
| hsa-miR-486-3p | 1.593017862 | 1.846703701 | 1.853028852 | 1.720956265 | 1.859944513 | 1.709604567 |
| hsa-miR-486-5p | 4.410843801 | 2.357647514 | 2.864251444 | 4.03999903 | 1.756117292 | 4.361492666 |
| hsa-miR-487a-3p | 1.622227607 | 1.834218907 | 1.761581401 | 1.645961732 | 1.826295262 | 1.757417508 |
| hsa-miR-487a-5p | 1.785726145 | 1.993210009 | 1.816977399 | 1.636543243 | 1.717167245 | 2.081699527 |
| hsa-miR-487b-3p | 1.689214366 | 1.650906329 | 1.707700579 | 3.299956671 | 1.836190834 | 3.972094937 |
| hsa-miR-487b-5p | 1.700792483 | 2.005781834 | 1.762027973 | 1.674069937 | 1.739428252 | 1.731454773 |
| hsa-miR-488-3p | 1.823031047 | 1.750308296 | 1.887511828 | 1.787399873 | 1.93388456 | 1.744427582 |
| hsa-miR-488-5p | 1.724324866 | 1.792621811 | 1.98601478 | 1.721532564 | 2.756386221 | 2.015889028 |
| hsa-miR-489-3p | 1.671327446 | 1.759523763 | 1.862733108 | 1.630860392 | 1.86308069 | 1.804664682 |
| hsa-miR-489-5p | 1.810922457 | 1.697193382 | 1.654509323 | 1.696813124 | 1.762944131 | 1.597881213 |
| hsa-miR-490-3p | 1.753775385 | 1.709304524 | 1.681047675 | 2.207417672 | 1.701562664 | 1.786566761 |
| hsa-miR-490-5p | 1.860232771 | 2.257417104 | 1.76878612 | 2.011312474 | 1.722858931 | 1.825915084 |
| hsa-miR-491-3p | 1.683347249 | 1.831783594 | 1.962512605 | 1.81466374 | 1.892786698 | 1.710602523 |
| hsa-miR-491-5p | 1.69386475 | 1.693476516 | 1.738869895 | 1.839011395 | 1.702139581 | 1.717814462 |
| hsa-miR-492 | 1.750219732 | 1.857132612 | 1.791701348 | 1.599294003 | 2.534787121 | 1.852181474 |
| hsa-miR-493-3p | 1.656379345 | 1.791357849 | 1.752486545 | 1.729184355 | 2.177836591 | 1.863102796 |
| hsa-miR-493-5p | 1.840616702 | 1.81446892 | 1.688987623 | 1.831673958 | 1.8886164 | 1.818771626 |
| hsa-miR-494-3p | 4.841753427 | 4.081836207 | 1.850838245 | 5.191072115 | 1.878202971 | 3.062119358 |
| hsa-miR-494-5p | 1.782211162 | 1.800493058 | 2.175424109 | 1.730840808 | 1.84344422 | 1.655321356 |
| hsa-miR-495-3p | 1.707330265 | 1.818400119 | 1.869696162 | 2.894876585 | 2.598066397 | 4.133239818 |
| hsa-miR-495-5p | 1.718779857 | 1.768615305 | 1.844924418 | 1.728502334 | 2.486032988 | 2.011103992 |
| hsa-miR-496 | 1.700183274 | 1.782364778 | 1.867144257 | 1.672837652 | 2.02892476 | 1.784415285 |
| hsa-miR-497-3p | 1.664346496 | 1.8148722 | 1.990292619 | 1.65532827 | 1.805437025 | 1.755086087 |
| hsa-miR-497-5p | 1.775065134 | 1.797924783 | 1.942855701 | 5.473615272 | 2.896391434 | 8.930839591 |
| hsa-miR-498 | 1.97106091 | 1.772061243 | 1.780249929 | 2.694771815 | 2.030842766 | 1.836824233 |
| hsa-miR-4999-3p | 1.604601738 | 1.746058726 | 1.77898134 | 1.647549739 | 3.170606447 | 2.069708499 |
| hsa-miR-4999-5p | 1.886941484 | 1.811559508 | 1.81097164 | 1.812286339 | 1.909261508 | 1.994711777 |
| hsa-miR-499a-3p | 1.648885283 | 1.700534058 | 1.736097932 | 1.629085706 | 2.079148915 | 1.636445426 |
| hsa-miR-499a-5p | 1.955063652 | 2.105905123 | 1.884591884 | 2.188153692 | 1.771923091 | 1.915176915 |
| hsa-miR-499b-3p | 1.668959058 | 1.706543793 | 1.812240692 | 1.634187121 | 1.908374624 | 1.826132285 |
| hsa-miR-499b-5p | 1.862792357 | 1.834989981 | 1.939678266 | 1.713111362 | 1.933518005 | 1.789292389 |
| hsa-miR-5000-3p | 1.778871496 | 1.741992203 | 1.733525742 | 1.730118499 | 2.102276888 | 2.058341766 |
| hsa-miR-5000-5p | 1.783258283 | 1.678606391 | 1.880655303 | 1.732422444 | 1.775604579 | 1.710419366 |
| hsa-miR-5001-3p | 1.721845591 | 1.67867696 | 1.99470197 | 1.628093813 | 1.936943773 | 1.694680018 |
| hsa-miR-5001-5p | 7.427892891 | 4.168553854 | 3.040603232 | 3.801901029 | 1.8373385 | 4.962751227 |
| hsa-miR-5002-3p | 1.685736215 | 1.699393223 | 1.791483079 | 1.689823947 | 1.70982115 | 1.660386479 |
| hsa-miR-5002-5p | 1.687725273 | 1.759686338 | 1.801249586 | 1.70625856 | 1.754274352 | 1.807778535 |
| hsa-miR-5003-3p | 2.047407801 | 2.114214062 | 1.802818133 | 4.066406826 | 2.051302396 | 3.987622431 |
| hsa-miR-5003-5p | 1.812652296 | 1.697371969 | 1.652395767 | 1.68591622 | 1.662728393 | 1.672560424 |
| hsa-miR-5004-3p | 1.729134894 | 1.670108667 | 1.659894838 | 1.622060568 | 1.86410622 | 1.667618665 |
| hsa-miR-5004-5p | 1.596564702 | 1.722322889 | 1.7072516 | 1.666628535 | 1.651896927 | 1.71954378 |
| hsa-miR-5006-3p | 1.743902928 | 1.84088668 | 1.825524207 | 1.696433389 | 1.721339191 | 1.799910224 |
| hsa-miR-5006-5p | 6.570169647 | 3.198325135 | 3.717315871 | 5.875949679 | 1.750545801 | 2.411773354 |
| hsa-miR-5007-3p | 1.761007043 | 1.894284435 | 2.291315814 | 1.709821095 | 1.87050138 | 1.869775302 |
| hsa-miR-5007-5p | 1.700513955 | 1.71142312 | 1.748200027 | 1.713490766 | 1.797439456 | 1.813281701 |
| hsa-miR-5008-3p | 1.684170976 | 1.654313885 | 1.654754177 | 1.811086319 | 1.837841533 | 1.729455252 |
| hsa-miR-5008-5p | 3.37748093 | 1.858222791 | 1.843635813 | 3.222639184 | 1.734202545 | 1.676100796 |
| hsa-miR-5009-3p | 1.744572185 | 1.76186375 | 1.843974237 | 1.734254944 | 2.149299623 | 1.735396609 |
| hsa-miR-5009-5p | 1.769949047 | 2.093671811 | 1.865875429 | 1.708854424 | 2.09373305 | 1.862395531 |
| hsa-miR-500a-3p | 2.356656534 | 1.796628239 | 1.760769062 | 5.818152307 | 1.890360077 | 7.615396979 |
| hsa-miR-500a-5p | 1.928146142 | 1.872644153 | 1.848030279 | 1.924747454 | 1.748879028 | 1.708245296 |
| hsa-miR-500b-3p | 1.690555102 | 1.752722049 | 1.784553025 | 1.683739878 | 1.866110372 | 1.817025451 |
| hsa-miR-500b-5p | 1.775967565 | 1.839378302 | 1.746924648 | 2.653759931 | 1.680914837 | 2.100978044 |
| hsa-miR-501-3p | 1.782179424 | 1.744953524 | 1.73400111 | 2.583164668 | 1.749347381 | 1.870324867 |
| hsa-miR-501-5p | 2.412551566 | 1.931157477 | 2.278119868 | 2.322530772 | 1.828062093 | 2.323879888 |
| hsa-miR-5010-3p | 2.085332214 | 2.535583913 | 2.536155708 | 2.316362619 | 3.525898957 | 3.126786807 |
| hsa-miR-5010-5p | 2.036617549 | 2.048555698 | 2.375901235 | 1.983857287 | 1.984642626 | 2.133146566 |
| hsa-miR-5011-3p | 1.850524897 | 1.827867241 | 1.901127478 | 1.592194702 | 1.83966214 | 1.857744227 |
| hsa-miR-5011-5p | 1.675234013 | 1.692004741 | 1.790867905 | 1.680399059 | 1.75447721 | 1.952381161 |
| hsa-miR-502-3p | 1.891285438 | 1.799112581 | 1.684820766 | 6.556579858 | 1.778362332 | 7.616958666 |
| hsa-miR-502-5p | 1.779250151 | 1.718234789 | 1.742220533 | 2.126899984 | 1.871739479 | 1.864710907 |
| hsa-miR-503-3p | 1.770508997 | 1.767722968 | 1.715814271 | 1.80500994 | 1.632251245 | 1.706185308 |
| hsa-miR-503-5p | 1.756534111 | 1.684346082 | 1.650727388 | 1.716542889 | 1.818027735 | 1.711374355 |
| hsa-miR-504-3p | 1.862574348 | 1.783170476 | 1.884972486 | 5.089037115 | 1.766166218 | 1.769240344 |
| hsa-miR-504-5p | 1.730282094 | 1.960489551 | 1.682410335 | 1.651719098 | 1.919602261 | 1.750982163 |
| hsa-miR-5047 | 1.85779994 | 1.820868009 | 1.654944915 | 1.623627165 | 1.735959426 | 1.735021806 |
| hsa-miR-505-3p | 3.548974053 | 1.807766555 | 2.088071605 | 4.085462155 | 2.153271207 | 3.410961035 |
| hsa-miR-505-5p | 2.03973087 | 1.869320759 | 1.808868579 | 1.873827799 | 1.795774085 | 1.981993549 |
| hsa-miR-506-3p | 1.619526389 | 1.871195097 | 2.2111069 | 1.6571458 | 1.859868602 | 1.99134609 |
| hsa-miR-506-5p | 1.752580526 | 1.752590344 | 1.791683255 | 3.943904938 | 1.81405303 | 2.243025308 |
| hsa-miR-507 | 1.691584939 | 1.873946916 | 1.826677703 | 1.628825937 | 2.318867277 | 2.020709942 |
| hsa-miR-508-3p | 1.765276845 | 1.799925915 | 1.900626526 | 1.815202896 | 1.760335906 | 1.848270533 |
| hsa-miR-508-5p | 1.711055471 | 1.719643743 | 1.641692337 | 1.633529116 | 1.775989266 | 1.744688458 |
| hsa-miR-5087 | 1.644482852 | 1.687444967 | 1.761340923 | 1.648307957 | 1.85333645 | 1.759827008 |
| hsa-miR-5088-3p | 1.759510959 | 1.750703903 | 1.659012495 | 1.69215354 | 1.617906769 | 1.660546139 |
| hsa-miR-5088-5p | 7.083452142 | 1.684617294 | 2.081776798 | 4.022540024 | 1.791991791 | 1.735273994 |
| hsa-miR-5089-3p | 1.79104575 | 1.631401633 | 1.718398198 | 1.598845655 | 1.686258784 | 1.788360914 |
| hsa-miR-5089-5p | 1.736221376 | 1.732193745 | 1.67380319 | 1.720797566 | 1.699091572 | 1.694684475 |
| hsa-miR-509-3-5p | 1.836627952 | 1.818458522 | 1.745246473 | 1.755588947 | 1.794564106 | 1.712431442 |
| hsa-miR-509-3p | 1.728478603 | 1.73026079 | 1.903413195 | 1.749344185 | 1.989763018 | 1.804541592 |
| hsa-miR-509-5p | 1.695813846 | 1.829436503 | 1.677333733 | 7.27037869 | 2.008264071 | 9.838529795 |
| hsa-miR-5090 | 1.782423075 | 1.752399093 | 1.816378494 | 3.874115319 | 1.832360962 | 1.607823853 |
| hsa-miR-5091 | 1.725038398 | 1.667495207 | 1.695083756 | 1.632338155 | 1.751648524 | 1.648443249 |
| hsa-miR-5092 | 1.646874272 | 1.771591814 | 2.222591253 | 1.577329965 | 1.69811157 | 1.789354257 |
| hsa-miR-5093 | 1.719655029 | 1.685520512 | 1.865620801 | 1.682981832 | 1.652763574 | 1.723972531 |
| hsa-miR-5094 | 1.669938475 | 2.098953668 | 1.721849237 | 1.604197914 | 1.920689921 | 1.739543187 |
| hsa-miR-5095 | 1.736433766 | 2.176100622 | 1.884195929 | 1.807564837 | 1.750175965 | 1.847779693 |
| hsa-miR-5096 | 2.025407984 | 2.513558825 | 1.901796452 | 1.64547233 | 1.839473944 | 1.690138154 |
| hsa-miR-510-3p | 1.858566006 | 1.890121394 | 1.74920836 | 1.7858446 | 2.113271226 | 1.899498576 |
| hsa-miR-510-5p | 1.733021522 | 1.696447648 | 1.779838626 | 1.673676231 | 1.667862469 | 1.752788834 |
| hsa-miR-5100 | 10.87488161 | 9.150872322 | 5.232889281 | 6.975063615 | 8.093206905 | 10.03759297 |
| hsa-miR-511-3p | 1.622902769 | 1.78222354 | 1.663969876 | 1.726815429 | 1.722797874 | 1.625653886 |
| hsa-miR-511-5p | 1.635048435 | 1.834770831 | 1.751944385 | 1.598028739 | 1.815405305 | 1.886468872 |
| hsa-miR-512-3p | 1.852081916 | 1.848230304 | 1.927206674 | 1.674455826 | 2.157552302 | 1.754471585 |
| hsa-miR-512-5p | 1.702918498 | 1.805826305 | 1.663901414 | 1.850187504 | 1.957604695 | 1.761451951 |
| hsa-miR-513a-3p | 2.404609073 | 1.63617666 | 1.586685639 | 3.078291667 | 1.702787259 | 1.623065091 |
| hsa-miR-513a-5p | 3.370572849 | 3.641997522 | 4.434746441 | 2.891568207 | 2.061336876 | 2.796744738 |
| hsa-miR-513b-3p | 2.136043329 | 1.76352788 | 1.808640807 | 1.836470526 | 2.118654953 | 1.870372412 |
| hsa-miR-513b-5p | 1.750665413 | 1.879541728 | 1.792106167 | 1.76199608 | 2.139635985 | 1.802896711 |
| hsa-miR-513c-3p | 1.845263953 | 1.619650527 | 1.82351857 | 1.992600039 | 2.215375784 | 1.71845418 |
| hsa-miR-513c-5p | 1.808426466 | 1.871524064 | 1.694822761 | 1.930006526 | 2.184263088 | 1.832372955 |
| hsa-miR-514a-3p | 1.738311132 | 1.779681631 | 1.824503005 | 1.670243111 | 2.119824222 | 1.979126394 |
| hsa-miR-514a-5p | 1.845352806 | 1.747207255 | 2.513979049 | 4.812468366 | 1.69987928 | 3.446733725 |
| hsa-miR-514b-3p | 1.828767261 | 1.700045866 | 1.720881416 | 1.732247808 | 2.53507256 | 1.807865221 |
| hsa-miR-514b-5p | 4.640987827 | 3.133726415 | 4.092360746 | 7.345201439 | 2.336673349 | 4.752360802 |
| hsa-miR-515-3p | 2.010363973 | 1.679368171 | 1.802513868 | 1.727539978 | 2.070549057 | 1.754796682 |
| hsa-miR-515-5p | 2.021211004 | 1.709172333 | 1.726227395 | 1.747143797 | 1.807932339 | 1.705936017 |
| hsa-miR-516a-3p | 1.82233989 | 1.720955549 | 1.72736619 | 1.695957944 | 1.708506825 | 1.706055048 |
| hsa-miR-516a-5p | 2.471918284 | 1.926604157 | 1.969616502 | 3.146691239 | 1.789857382 | 1.828141378 |
| hsa-miR-516b-5p | 1.776610302 | 1.705120369 | 2.000271796 | 3.727062673 | 2.121302816 | 1.768091458 |
| hsa-miR-517-5p | 1.673603182 | 1.834866386 | 1.868244331 | 1.720174589 | 2.018406162 | 1.949408899 |
| hsa-miR-517a-3p | 1.744572241 | 1.729499955 | 1.653296756 | 1.780717424 | 1.629428364 | 1.678787463 |
| hsa-miR-517c-3p | 1.704590709 | 1.796079669 | 1.760558116 | 1.704546583 | 1.863317622 | 1.848296423 |
| hsa-miR-5186 | 1.787023304 | 1.842553954 | 1.686295833 | 1.762825201 | 1.694390867 | 1.652967445 |
| hsa-miR-5187-3p | 1.729225088 | 1.911306618 | 2.174982516 | 1.705952666 | 1.854268013 | 1.823597824 |
| hsa-miR-5187-5p | 1.771006357 | 1.646072935 | 1.742567649 | 1.652720455 | 1.820153197 | 1.866753972 |
| hsa-miR-5188 | 1.797418635 | 1.835039545 | 1.703901835 | 2.010194402 | 1.628804197 | 1.670185927 |
| hsa-miR-5189-3p | 1.857823534 | 1.7726087 | 1.857160267 | 2.20081913 | 1.793793977 | 1.769487002 |
| hsa-miR-5189-5p | 2.611057846 | 1.869052563 | 1.982043622 | 2.477563942 | 1.628152235 | 1.822445382 |
| hsa-miR-518a-3p | 1.71974526 | 1.771622669 | 1.752472013 | 1.738184713 | 1.732067533 | 1.657762037 |
| hsa-miR-518a-5p | 1.766971097 | 1.810595682 | 1.801125078 | 1.671435073 | 1.849056494 | 1.718894608 |
| hsa-miR-518b | 1.806341165 | 1.785840129 | 2.016239474 | 1.755164475 | 2.534214708 | 1.867379292 |
| hsa-miR-518c-3p | 1.994237603 | 1.748623255 | 1.848114144 | 1.753043061 | 1.769476107 | 1.820890195 |
| hsa-miR-518c-5p | 1.921729424 | 1.927709485 | 1.809509698 | 2.003453182 | 1.683372114 | 1.834104967 |
| hsa-miR-518d-3p | 1.590657164 | 1.978241377 | 1.894931451 | 1.639563215 | 1.921031951 | 1.790375329 |
| hsa-miR-518e-3p | 1.715028225 | 1.657370363 | 1.697020162 | 1.696696885 | 1.661567346 | 1.617259171 |
| hsa-miR-518e-5p | 2.266062126 | 1.860047552 | 2.406306743 | 2.195762617 | 1.965108543 | 2.040422792 |
| hsa-miR-518f-3p | 1.983925188 | 1.622334758 | 1.701270391 | 1.917491116 | 1.761827148 | 1.754844914 |
| hsa-miR-518f-5p | 1.927022696 | 1.894082004 | 1.758311184 | 1.692726011 | 1.899997462 | 1.707781387 |
| hsa-miR-5190 | 2.00096386 | 1.852254661 | 1.868164981 | 2.387917823 | 1.843291989 | 1.961242077 |
| hsa-miR-5191 | 1.743077534 | 1.785868355 | 1.909333446 | 1.987614771 | 1.937444976 | 1.834309929 |
| hsa-miR-5192 | 1.790248297 | 1.636112397 | 1.663254711 | 1.676964763 | 1.720162587 | 1.638479628 |
| hsa-miR-5193 | 1.899921827 | 1.752621006 | 1.749057047 | 1.713911732 | 1.783850074 | 1.667890629 |
| hsa-miR-5194 | 2.721914637 | 1.792882469 | 1.933471242 | 2.267901742 | 2.447443734 | 2.457289843 |
| hsa-miR-5195-3p | 4.355580448 | 1.960702889 | 1.900357379 | 5.636237729 | 1.812326897 | 2.322231235 |
| hsa-miR-5195-5p | 1.776154564 | 1.785167682 | 1.904878338 | 1.793639862 | 1.743438394 | 1.762098623 |
| hsa-miR-5196-3p | 2.984470832 | 1.939319681 | 2.068784136 | 1.990225144 | 1.829042725 | 1.878058204 |
| hsa-miR-5196-5p | 7.264014705 | 8.537729435 | 8.66767825 | 6.499227091 | 2.170531343 | 8.106555988 |
| hsa-miR-5197-3p | 1.666475121 | 1.775366707 | 1.708838507 | 1.712536142 | 1.687433077 | 1.798902199 |
| hsa-miR-5197-5p | 1.81737882 | 1.658773816 | 1.743504404 | 1.693918529 | 1.78025539 | 1.646837966 |
| hsa-miR-519b-3p | 1.826366472 | 1.743997469 | 1.782177018 | 1.772431989 | 2.104947219 | 1.740272458 |
| hsa-miR-519c-3p | 1.792911167 | 1.724768398 | 1.86623807 | 1.681595085 | 1.831601659 | 1.918016822 |
| hsa-miR-519d-3p | 1.74250624 | 1.772379839 | 1.70197337 | 1.689366957 | 2.060511456 | 1.719896161 |
| hsa-miR-519d-5p | 1.795375082 | 1.763885901 | 1.784780044 | 1.654984781 | 1.74058035 | 1.816085497 |
| hsa-miR-519e-3p | 1.772654903 | 1.915540268 | 1.802980329 | 1.738355576 | 2.419842183 | 1.775162335 |
| hsa-miR-519e-5p | 1.809935285 | 1.724754491 | 1.857740449 | 2.108008074 | 1.933975495 | 1.909828086 |
| hsa-miR-520a-3p | 1.823074154 | 1.986483125 | 1.758977198 | 1.700322344 | 1.929692874 | 1.929803075 |
| hsa-miR-520a-5p | 1.824923155 | 1.805421848 | 1.72389505 | 1.792442875 | 1.730157854 | 1.677833035 |
| hsa-miR-520b | 2.440206645 | 2.495004971 | 2.266361079 | 3.979809195 | 1.880368102 | 3.442148261 |
| hsa-miR-520c-3p | 1.852405447 | 1.827274251 | 1.921415401 | 1.643689774 | 1.879973883 | 1.94414283 |
| hsa-miR-520d-3p | 1.77689189 | 1.809915509 | 2.19479713 | 1.754013675 | 1.924627714 | 2.087175921 |
| hsa-miR-520e | 2.041870127 | 1.774151606 | 1.883217644 | 3.97138133 | 1.91178753 | 3.667515636 |
| hsa-miR-520f-3p | 1.76346714 | 1.83554682 | 1.861293983 | 1.940932082 | 1.954101164 | 1.92529821 |
| hsa-miR-520f-5p | 1.73813679 | 1.658439832 | 1.691581312 | 1.676463792 | 1.674838672 | 1.637755162 |
| hsa-miR-520g-3p | 1.784104563 | 2.288342957 | 1.853938856 | 1.667073178 | 1.842568671 | 1.773283076 |
| hsa-miR-520g-5p | 1.643413011 | 1.823222939 | 1.784768877 | 1.665259338 | 1.953269078 | 1.889441115 |
| hsa-miR-520h | 1.770950108 | 1.682906698 | 1.793035223 | 1.700995949 | 1.773498465 | 1.722475301 |
| hsa-miR-521 | 1.650418 | 2.050001799 | 1.784991692 | 1.623263471 | 1.749764549 | 1.781346356 |
| hsa-miR-522-3p | 1.721948849 | 1.878628799 | 1.982347023 | 1.723176925 | 2.389091287 | 2.02501335 |
| hsa-miR-523-3p | 1.699637127 | 1.688453661 | 1.806357401 | 1.578922093 | 1.810711586 | 1.757777999 |
| hsa-miR-524-3p | 1.647010966 | 1.6632566 | 1.744764206 | 1.666976683 | 1.686004919 | 1.634771721 |
| hsa-miR-525-3p | 1.83087997 | 1.801058317 | 1.65325158 | 1.71316559 | 1.877441317 | 1.736986499 |
| hsa-miR-525-5p | 1.779982617 | 2.225815746 | 1.972134366 | 2.043495239 | 2.008111001 | 2.181708881 |
| hsa-miR-526b-3p | 1.775380261 | 1.728890411 | 1.788942877 | 1.81484758 | 1.909686983 | 1.771725335 |
| hsa-miR-526b-5p | 3.219124215 | 2.070820545 | 2.011358786 | 3.123272685 | 2.298535021 | 1.829256984 |
| hsa-miR-532-3p | 3.801512181 | 1.705741556 | 1.817185196 | 4.827480708 | 1.789074448 | 1.980479868 |
| hsa-miR-532-5p | 3.46151182 | 1.895841979 | 1.941001117 | 1.848599685 | 1.985232313 | 2.215693568 |
| hsa-miR-539-3p | 1.766815705 | 1.731586413 | 1.734389228 | 1.779259888 | 1.993925607 | 1.803811761 |
| hsa-miR-539-5p | 1.869692285 | 1.955184602 | 1.810009037 | 1.972624435 | 1.903957211 | 1.684059226 |
| hsa-miR-541-3p | 1.664150837 | 1.692103176 | 1.754269862 | 1.624612608 | 2.307251739 | 1.772707944 |
| hsa-miR-541-5p | 1.755768375 | 1.671126304 | 1.666419341 | 1.609593862 | 1.699233693 | 1.640760093 |
| hsa-miR-542-3p | 1.811459059 | 1.714041765 | 1.755799126 | 2.551811556 | 2.223004965 | 2.253271889 |
| hsa-miR-542-5p | 1.832519838 | 1.707824338 | 1.700875434 | 3.884214488 | 1.840181387 | 2.80769397 |
| hsa-miR-543 | 1.664741145 | 1.768279624 | 1.916598853 | 2.308892358 | 1.939517972 | 2.146236284 |
| hsa-miR-544a | 2.032355641 | 2.607629577 | 1.864191414 | 1.734695701 | 1.80253187 | 1.806357942 |
| hsa-miR-544b | 1.66954538 | 1.910263652 | 1.963760021 | 1.709284247 | 1.907675649 | 2.201208974 |
| hsa-miR-545-3p | 1.809659977 | 1.688718084 | 1.739595155 | 1.737935282 | 2.089902035 | 1.767397683 |
| hsa-miR-545-5p | 1.857115943 | 1.743339615 | 2.233828322 | 1.758082544 | 2.803520486 | 2.049750425 |
| hsa-miR-548a-3p | 1.842074756 | 1.921614492 | 1.881690108 | 1.65472073 | 1.874053866 | 1.861827255 |
| hsa-miR-548a-5p | 1.915039036 | 1.733389437 | 1.861142327 | 1.713076454 | 2.169237284 | 1.919477492 |
| hsa-miR-548aa | 1.74595067 | 1.801656701 | 1.859807824 | 1.779093467 | 1.928139031 | 1.825291986 |
| hsa-miR-548ab | 1.850007274 | 2.044810568 | 1.674341335 | 1.787034333 | 1.731341583 | 1.694692478 |
| hsa-miR-548ac | 1.629752537 | 1.985876485 | 1.869414094 | 1.626365321 | 2.041690338 | 2.049412161 |
| hsa-miR-548ad-3p | 1.646024689 | 1.828856896 | 2.018184613 | 1.639132977 | 1.920074397 | 1.811079892 |
| hsa-miR-548ad-5p | 2.218330243 | 1.849504184 | 2.474320465 | 1.84434432 | 2.66648825 | 1.939004589 |
| hsa-miR-548ae-3p | 1.870749778 | 1.755968733 | 1.869628598 | 1.815703929 | 2.172166085 | 1.825774322 |
| hsa-miR-548ag | 1.792342496 | 2.466096372 | 1.850427451 | 1.71147199 | 2.341403216 | 2.272511029 |
| hsa-miR-548ah-5p | 1.677928866 | 2.007541464 | 1.745660006 | 1.704512108 | 1.864540959 | 1.942739905 |
| hsa-miR-548ai | 1.862843456 | 1.968804899 | 1.886728966 | 1.778713146 | 2.076807234 | 1.848161873 |
| hsa-miR-548aj-3p | 1.729225514 | 1.839575774 | 1.780955718 | 1.721197797 | 1.772876902 | 1.960315146 |
| hsa-miR-548aj-5p | 1.853012726 | 1.890379645 | 2.088786953 | 1.761653814 | 2.007243316 | 2.027815693 |
| hsa-miR-548ak | 1.803875954 | 1.950168388 | 1.74663393 | 1.657665544 | 1.759461932 | 1.797309181 |
| hsa-miR-548al | 1.637765562 | 1.79447694 | 2.044550397 | 1.659032427 | 2.130285701 | 1.919279066 |
| hsa-miR-548am-3p | 1.794448716 | 1.793917126 | 1.749446084 | 1.728647862 | 1.882033731 | 1.780066563 |
| hsa-miR-548am-5p | 1.729264799 | 1.95821188 | 1.902547716 | 1.686347018 | 2.113570431 | 2.151539653 |
| hsa-miR-548an | 1.727666574 | 1.999641116 | 2.106960606 | 1.686900526 | 1.90592422 | 1.694292304 |
| hsa-miR-548ao-3p | 1.75859123 | 1.703467462 | 1.680491147 | 1.60218334 | 1.87612188 | 1.740930762 |
| hsa-miR-548ao-5p | 1.73784817 | 1.748546382 | 1.664341588 | 1.675891571 | 2.441908324 | 1.736533284 |
| hsa-miR-548ap-3p | 1.675737477 | 2.385679302 | 1.801430076 | 1.800414849 | 1.803375792 | 1.900000464 |
| hsa-miR-548ap-5p | 1.83055247 | 1.921270048 | 1.955151064 | 1.721314156 | 2.196288955 | 2.996109112 |
| hsa-miR-548aq-5p | 1.851294015 | 1.790637479 | 1.81882261 | 1.790635688 | 2.439453922 | 1.774266149 |
| hsa-miR-548ar-3p | 1.745035699 | 1.739734425 | 1.849018542 | 1.764419517 | 1.87156029 | 1.685699821 |
| hsa-miR-548ar-5p | 1.741647939 | 1.83959444 | 2.060935783 | 1.668330794 | 1.956768082 | 2.116618067 |
| hsa-miR-548as-3p | 1.829410358 | 1.836371693 | 1.698534368 | 1.815398109 | 1.799537007 | 1.699233802 |
| hsa-miR-548as-5p | 1.643930914 | 1.780148342 | 1.719915632 | 1.663442485 | 1.793469912 | 1.734789302 |
| hsa-miR-548at-3p | 1.741696411 | 1.773736233 | 1.733589353 | 1.715311193 | 1.700586844 | 1.673659333 |
| hsa-miR-548at-5p | 1.734426571 | 2.251762291 | 1.664473026 | 1.843829218 | 1.917826445 | 1.796285578 |
| hsa-miR-548au-3p | 1.899059261 | 1.793490997 | 1.904558491 | 1.700444947 | 2.121885624 | 1.848827753 |
| hsa-miR-548au-5p | 1.920688665 | 1.963138751 | 1.817506161 | 1.82014871 | 1.83743753 | 1.796305944 |
| hsa-miR-548av-3p | 1.818415379 | 1.841231468 | 1.759372632 | 1.787928631 | 1.834195863 | 1.659345138 |
| hsa-miR-548av-5p | 1.808622149 | 2.693493033 | 1.843248083 | 1.671196492 | 1.959722048 | 1.760686677 |
| hsa-miR-548aw | 1.733598221 | 1.648899526 | 1.689895868 | 1.633117996 | 1.915848066 | 1.858207076 |
| hsa-miR-548ax | 1.696950177 | 1.73380966 | 1.635752237 | 1.655283718 | 1.771997072 | 1.704373917 |
| hsa-miR-548ay-3p | 1.689908648 | 1.85295744 | 1.735449995 | 1.616860847 | 1.821671523 | 1.776068447 |
| hsa-miR-548ay-5p | 2.039846912 | 2.009866668 | 1.804102108 | 1.766134295 | 2.802552988 | 1.979416238 |
| hsa-miR-548az-3p | 1.725128169 | 2.018596248 | 1.716412069 | 1.643866732 | 1.977095778 | 1.750399017 |
| hsa-miR-548az-5p | 1.654305672 | 1.910607164 | 1.735533465 | 1.674565606 | 1.868696906 | 2.045748854 |
| hsa-miR-548b-3p | 1.781909296 | 1.859391351 | 1.737825471 | 1.740550463 | 1.773180787 | 1.778852143 |
| hsa-miR-548b-5p | 1.683611187 | 2.076441099 | 2.318805496 | 1.736010371 | 1.932697963 | 1.972667645 |
| hsa-miR-548ba | 1.814824618 | 1.812473179 | 2.332566252 | 1.687602258 | 1.933632945 | 1.851809067 |
| hsa-miR-548bb-3p | 1.728808482 | 1.777058534 | 2.071386095 | 1.679733819 | 1.988276023 | 1.782054162 |
| hsa-miR-548bb-5p | 1.794411363 | 1.921855162 | 1.783940335 | 1.743803236 | 2.279563866 | 1.888305764 |
| hsa-miR-548c-3p | 1.721229023 | 2.40846915 | 1.842073967 | 1.795060953 | 1.825608244 | 2.002729185 |
| hsa-miR-548d-3p | 1.738545538 | 1.708486695 | 1.683921005 | 1.65295314 | 1.826700363 | 1.6726743 |
| hsa-miR-548d-5p | 1.855695615 | 2.091619492 | 2.139691107 | 1.691836687 | 1.920501163 | 2.025965748 |
| hsa-miR-548e-3p | 1.842268599 | 1.689154691 | 1.756638985 | 1.753157182 | 1.904868127 | 1.724187088 |
| hsa-miR-548e-5p | 1.698558933 | 1.700591797 | 1.787355406 | 1.63605961 | 1.692904175 | 1.693801864 |
| hsa-miR-548f-3p | 2.15074623 | 2.02162752 | 2.42887006 | 1.739315187 | 4.265593319 | 2.61565681 |
| hsa-miR-548f-5p | 2.032607019 | 1.75409148 | 1.799611652 | 1.816755688 | 2.068653846 | 1.759030711 |
| hsa-miR-548g-3p | 1.789411074 | 1.952415793 | 2.501798286 | 1.918330939 | 3.135625933 | 2.290643361 |
| hsa-miR-548h-3p | 1.656364466 | 1.830530065 | 2.415773099 | 1.611312214 | 2.159558675 | 2.190901615 |
| hsa-miR-548h-5p | 1.75421625 | 1.731505057 | 1.766798125 | 1.61671439 | 1.815301221 | 1.70072444 |
| hsa-miR-548i | 1.859664827 | 1.797326981 | 1.748633295 | 1.652569739 | 1.844592512 | 1.741970521 |
| hsa-miR-548j-3p | 1.639373766 | 1.682645958 | 1.899812788 | 1.714991516 | 2.336471446 | 1.786473431 |
| hsa-miR-548j-5p | 1.682337447 | 2.075334946 | 1.780332097 | 1.598920439 | 1.919024368 | 1.783362796 |
| hsa-miR-548k | 1.764388313 | 1.735251321 | 1.855180824 | 1.67024054 | 1.818105654 | 1.752742689 |
| hsa-miR-548l | 1.728124049 | 1.741070847 | 1.778602203 | 1.680211747 | 1.909339804 | 1.815957996 |
| hsa-miR-548m | 1.858306472 | 1.886642116 | 2.059816206 | 1.696285398 | 2.445710115 | 1.87516912 |
| hsa-miR-548n | 1.895944606 | 2.373225976 | 2.303950975 | 1.781163824 | 3.747373558 | 2.096764344 |
| hsa-miR-548p | 1.758975651 | 1.704120981 | 2.220557524 | 1.724406049 | 1.995413581 | 1.830061762 |
| hsa-miR-548q | 1.810528938 | 1.754501092 | 1.815210152 | 1.671648535 | 1.88321323 | 1.715116859 |
| hsa-miR-548s | 1.791822784 | 1.663872696 | 2.193976442 | 1.747661412 | 1.784044551 | 1.687412829 |
| hsa-miR-548t-5p | 1.921248148 | 1.799397909 | 1.79470141 | 1.762887989 | 1.730875563 | 1.721901706 |
| hsa-miR-548u | 1.711981615 | 1.737727394 | 1.801144984 | 1.649818023 | 2.730343933 | 1.961983875 |
| hsa-miR-548v | 1.730635571 | 1.852019299 | 1.842709441 | 1.772475372 | 2.088033574 | 1.799110974 |
| hsa-miR-548w | 1.734588637 | 1.80384885 | 2.011618109 | 1.629268676 | 1.721279097 | 1.70017798 |
| hsa-miR-548x-3p | 1.65742156 | 1.839055403 | 1.724730446 | 1.805921873 | 2.428543987 | 1.78463464 |
| hsa-miR-548y | 1.760510911 | 2.049678977 | 1.75721469 | 1.676753661 | 2.436907133 | 1.837781706 |
| hsa-miR-549a | 1.787197849 | 1.650643915 | 1.634271803 | 1.719809591 | 1.774453544 | 1.691284001 |
| hsa-miR-550a-3-5p | 2.451833656 | 1.769666895 | 1.786351303 | 6.333741162 | 1.883802573 | 1.854837427 |
| hsa-miR-550a-3p | 2.311023307 | 2.153436524 | 1.641014309 | 1.638446585 | 1.746568501 | 1.698666005 |
| hsa-miR-550a-5p | 1.751561104 | 2.401308405 | 2.046459932 | 2.381823051 | 2.471439236 | 2.999377443 |
| hsa-miR-550b-2-5p | 1.943692345 | 1.7542133 | 1.951666373 | 5.316904891 | 2.025056519 | 1.885045396 |
| hsa-miR-550b-3p | 1.644802166 | 1.71830042 | 1.61535658 | 1.64600827 | 1.66995995 | 1.642366691 |
| hsa-miR-551a | 1.647899545 | 1.76860731 | 1.917031408 | 1.612603398 | 2.079563472 | 1.837563471 |
| hsa-miR-551b-3p | 1.863997694 | 1.705123715 | 1.774295383 | 1.756836469 | 1.961887268 | 1.723644786 |
| hsa-miR-551b-5p | 1.621731523 | 1.783479396 | 1.724259528 | 1.832426706 | 1.736970395 | 1.819110558 |
| hsa-miR-552-3p | 1.717096508 | 1.733428042 | 1.652118552 | 1.706391454 | 2.002406321 | 1.687454209 |
| hsa-miR-552-5p | 1.727676884 | 1.768317505 | 1.681217122 | 1.66273049 | 1.70294689 | 1.658782544 |
| hsa-miR-553 | 1.711241542 | 1.757034344 | 1.868135375 | 1.709445535 | 2.29770862 | 1.951224224 |
| hsa-miR-554 | 1.57491843 | 1.786550106 | 1.748134829 | 1.612194961 | 1.877400256 | 1.71577748 |
| hsa-miR-555 | 1.710996572 | 2.069235548 | 1.993336971 | 1.631118377 | 1.958313137 | 1.760599792 |
| hsa-miR-556-3p | 1.818503793 | 1.995486146 | 1.791821477 | 1.717455131 | 2.265235037 | 1.785408272 |
| hsa-miR-556-5p | 1.846273842 | 1.845075004 | 1.768739926 | 1.73009829 | 2.000182688 | 1.757821507 |
| hsa-miR-557 | 1.796707166 | 1.80943665 | 1.804468019 | 3.519686281 | 1.723327403 | 1.884607103 |
| hsa-miR-5571-3p | 1.740963735 | 1.832368652 | 1.772096586 | 1.767541364 | 1.781853676 | 1.743400824 |
| hsa-miR-5571-5p | 2.599535065 | 2.722791155 | 4.131020157 | 2.71660081 | 4.758213004 | 4.159107553 |
| hsa-miR-5572 | 2.019480567 | 1.810192809 | 1.939525163 | 2.377431404 | 1.675246471 | 1.754331785 |
| hsa-miR-5579-3p | 1.70995908 | 1.695939487 | 1.627645081 | 1.655693541 | 1.947284656 | 1.655731207 |
| hsa-miR-5579-5p | 1.629345001 | 1.770374934 | 1.814198775 | 1.632877411 | 1.874940546 | 1.732268606 |
| hsa-miR-558 | 1.77311388 | 1.696445302 | 1.776798234 | 1.633709473 | 1.747144758 | 1.802821852 |
| hsa-miR-5580-3p | 1.664413113 | 1.929648044 | 1.788679518 | 1.830467394 | 1.783766139 | 1.881494826 |
| hsa-miR-5580-5p | 1.870070343 | 1.936117416 | 1.785605532 | 1.706627967 | 2.26717043 | 1.906814728 |
| hsa-miR-5581-3p | 1.693951607 | 1.746114419 | 1.707134837 | 1.679544515 | 1.768673458 | 1.713122431 |
| hsa-miR-5581-5p | 1.928621484 | 1.768973694 | 1.769785771 | 2.71926375 | 1.661560258 | 1.955301187 |
| hsa-miR-5582-3p | 1.690036292 | 1.718995938 | 1.755716361 | 1.727919737 | 1.814061728 | 2.10496081 |
| hsa-miR-5582-5p | 1.660717906 | 1.961246437 | 1.743417685 | 1.656496203 | 2.536142429 | 1.792210507 |
| hsa-miR-5583-3p | 1.858566432 | 1.970963825 | 2.101290069 | 1.916825927 | 3.886221739 | 1.909334396 |
| hsa-miR-5583-5p | 1.689957799 | 1.812593985 | 1.815660473 | 1.719854079 | 1.797340916 | 1.905152725 |
| hsa-miR-5584-3p | 1.749275402 | 1.780456234 | 1.796699196 | 1.671973008 | 1.801143059 | 1.680295499 |
| hsa-miR-5584-5p | 1.922479795 | 1.75563938 | 1.745692817 | 2.031314114 | 1.947605023 | 1.859760648 |
| hsa-miR-5585-3p | 5.101097117 | 4.940055046 | 3.076660935 | 1.796856366 | 1.786602851 | 1.767312109 |
| hsa-miR-5585-5p | 1.658095415 | 1.72207011 | 1.89948187 | 1.714667337 | 1.795917126 | 1.805557309 |
| hsa-miR-5586-3p | 1.625642067 | 1.721498503 | 1.710449411 | 1.683710921 | 1.785341937 | 1.78155652 |
| hsa-miR-5586-5p | 1.733202098 | 1.805145109 | 1.696041885 | 1.747059028 | 1.826170624 | 1.712451277 |
| hsa-miR-5587-3p | 1.820372944 | 1.637788764 | 1.678488376 | 1.683313213 | 1.66216492 | 1.663213572 |
| hsa-miR-5587-5p | 5.675168242 | 3.25661308 | 1.936793657 | 3.770357322 | 1.862529772 | 1.766279192 |
| hsa-miR-5588-3p | 1.608495553 | 1.796129326 | 1.716294461 | 1.672469818 | 2.172182486 | 1.71489338 |
| hsa-miR-5588-5p | 1.799485485 | 1.916719985 | 1.923809655 | 1.729597518 | 1.836833016 | 1.733156244 |
| hsa-miR-5589-3p | 1.654415561 | 1.699984937 | 1.671854083 | 1.599230329 | 1.757889966 | 1.714712342 |
| hsa-miR-5589-5p | 1.673812032 | 1.793239134 | 1.980644499 | 1.577523552 | 1.85290455 | 1.730320027 |
| hsa-miR-559 | 1.73010994 | 1.727421782 | 1.641436383 | 1.860509295 | 1.709712735 | 1.659851786 |
| hsa-miR-5590-3p | 1.787732341 | 1.784664156 | 1.952205455 | 1.771388794 | 2.027428108 | 1.818628346 |
| hsa-miR-5590-5p | 1.873169133 | 2.375257049 | 1.977285702 | 1.6587872 | 1.953495289 | 2.26129307 |
| hsa-miR-5591-3p | 1.706568151 | 1.863212634 | 1.782581184 | 1.689739113 | 1.707905824 | 1.744435145 |
| hsa-miR-5591-5p | 1.883830414 | 1.631172743 | 1.686045028 | 1.699243968 | 1.752469367 | 1.805217043 |
| hsa-miR-561-3p | 1.772444339 | 1.953600225 | 1.83066402 | 1.688394381 | 2.071499677 | 1.693057075 |
| hsa-miR-561-5p | 1.782568152 | 2.304145624 | 1.899160547 | 1.692041582 | 1.720540933 | 1.811002766 |
| hsa-miR-562 | 1.764029305 | 1.815940889 | 1.78211519 | 1.643875585 | 2.389751389 | 1.86924765 |
| hsa-miR-563 | 1.888791981 | 1.787665576 | 2.061553025 | 1.87995472 | 1.921867813 | 1.969089157 |
| hsa-miR-564 | 1.890221259 | 2.330320532 | 1.684637209 | 1.96130421 | 1.801399066 | 1.791402547 |
| hsa-miR-566 | 1.718145598 | 1.67966834 | 1.769795689 | 1.700224312 | 1.737372039 | 1.608850871 |
| hsa-miR-567 | 1.89907443 | 1.694952202 | 1.649101426 | 1.718286509 | 1.755990315 | 1.655655951 |
| hsa-miR-568 | 1.756545787 | 2.061304601 | 1.944673971 | 1.703539311 | 2.583346568 | 2.352394058 |
| hsa-miR-5680 | 1.69937606 | 1.675324127 | 1.672775873 | 1.727147247 | 1.768809756 | 1.60099959 |
| hsa-miR-5681a | 1.697707152 | 1.98451806 | 1.865129214 | 1.66909519 | 1.73273872 | 1.802251846 |
| hsa-miR-5681b | 1.699396014 | 1.886583138 | 1.803872671 | 1.823377928 | 1.894989516 | 1.747579841 |
| hsa-miR-5682 | 1.725369291 | 1.818861988 | 1.805196454 | 1.657747344 | 1.679890053 | 1.734071128 |
| hsa-miR-5683 | 1.761663397 | 1.721929474 | 1.739637306 | 1.697900162 | 1.748599395 | 1.751104752 |
| hsa-miR-5684 | 1.726219597 | 1.772339498 | 1.74491111 | 1.590242897 | 1.712167467 | 1.917755729 |
| hsa-miR-5685 | 4.969744703 | 2.05131862 | 3.757239886 | 6.163360989 | 1.88253644 | 5.039041348 |
| hsa-miR-5687 | 1.756184315 | 1.737367817 | 1.750814995 | 1.786271701 | 1.882080947 | 1.766419926 |
| hsa-miR-5688 | 1.698172028 | 1.580825548 | 1.75983802 | 1.628388333 | 1.676909891 | 1.64433023 |
| hsa-miR-5689 | 1.76987666 | 1.942034232 | 1.932576068 | 1.646414493 | 1.885157007 | 1.710991536 |
| hsa-miR-569 | 1.905775065 | 1.759636426 | 1.908622574 | 1.650643674 | 1.894229088 | 1.702705367 |
| hsa-miR-5690 | 1.896803589 | 1.746768683 | 1.815149278 | 1.970509933 | 2.202233038 | 1.81105395 |
| hsa-miR-5691 | 1.660389102 | 1.903285115 | 1.759237711 | 1.740463421 | 1.72727402 | 1.78103565 |
| hsa-miR-5692a | 1.677707732 | 1.650863005 | 1.848264459 | 1.659026315 | 1.751553891 | 1.812468714 |
| hsa-miR-5692b | 1.783026159 | 1.690487787 | 1.708358755 | 1.761662857 | 2.083002695 | 1.82592762 |
| hsa-miR-5692c | 1.65348397 | 1.675168691 | 1.774023863 | 1.757390736 | 2.043525547 | 1.758303244 |
| hsa-miR-5693 | 1.75226857 | 1.710896873 | 1.670461183 | 1.630955637 | 1.876667723 | 1.727210875 |
| hsa-miR-5694 | 1.765206963 | 1.713909927 | 1.78158718 | 1.650413837 | 1.736678837 | 1.867555754 |
| hsa-miR-5695 | 1.699231211 | 1.691069251 | 1.704743694 | 1.625312368 | 2.005014 | 1.759230526 |
| hsa-miR-5696 | 1.804535876 | 2.115764161 | 2.322139295 | 1.719889274 | 2.146885498 | 1.964109499 |
| hsa-miR-5697 | 1.782181471 | 1.808648821 | 1.859160407 | 2.107391702 | 2.06880606 | 1.836166942 |
| hsa-miR-5698 | 1.659771904 | 1.996007134 | 1.824881819 | 1.773605126 | 1.857588535 | 1.741770296 |
| hsa-miR-5699-3p | 1.906199351 | 1.735909094 | 1.968765539 | 1.727092835 | 2.588297181 | 1.823057785 |
| hsa-miR-5699-5p | 3.938202424 | 2.97266468 | 3.334692351 | 8.321428931 | 2.072326832 | 3.439976882 |
| hsa-miR-570-3p | 1.697490627 | 1.747416245 | 1.855303712 | 1.594840472 | 2.005052888 | 2.149066279 |
| hsa-miR-5700 | 1.794670317 | 1.871295427 | 1.823190197 | 2.000421332 | 2.534404051 | 1.911165673 |
| hsa-miR-5701 | 1.853137008 | 1.91143998 | 1.921520532 | 1.664698899 | 2.17309587 | 2.085170023 |
| hsa-miR-5702 | 1.617655781 | 1.734395233 | 1.765488187 | 1.679785356 | 1.894223267 | 1.742462252 |
| hsa-miR-5703 | 11.10530879 | 10.32098609 | 10.54175095 | 10.68125172 | 1.983065228 | 7.229070363 |
| hsa-miR-5704 | 2.079859236 | 1.726635995 | 1.792430388 | 1.726754324 | 1.698122993 | 1.762072033 |
| hsa-miR-5705 | 1.656818276 | 1.781905623 | 1.82592479 | 1.66534992 | 1.886975607 | 1.785077113 |
| hsa-miR-5706 | 1.745491697 | 1.758820746 | 1.765244666 | 1.678301206 | 1.959659129 | 1.708550537 |
| hsa-miR-5707 | 1.670761015 | 1.736269087 | 1.800318647 | 1.638511052 | 1.930747208 | 1.889258913 |
| hsa-miR-5708 | 1.776167536 | 1.692394942 | 1.754492837 | 1.877124209 | 1.657841006 | 1.702825795 |
| hsa-miR-571 | 1.714027002 | 1.780763293 | 1.754473698 | 1.701949942 | 1.879539241 | 1.83120129 |
| hsa-miR-572 | 4.100675892 | 1.807611458 | 1.733568721 | 1.810118681 | 1.697928664 | 1.658687371 |
| hsa-miR-573 | 1.888048942 | 1.813526957 | 1.754987554 | 1.620721408 | 1.895120854 | 1.716041775 |
| hsa-miR-5739 | 8.061054066 | 6.717670663 | 6.690805991 | 8.112797536 | 3.19688329 | 6.422862134 |
| hsa-miR-574-3p | 5.125228479 | 8.996223335 | 7.038170517 | 6.244969019 | 3.834507085 | 7.726093664 |
| hsa-miR-574-5p | 11.07572536 | 10.90852166 | 11.9342318 | 8.431316411 | 10.0700579 | 9.678705277 |
| hsa-miR-575 | 10.06685453 | 5.189090185 | 2.586722661 | 4.089353159 | 1.757529523 | 1.818560939 |
| hsa-miR-576-3p | 1.788030955 | 1.852145453 | 1.667777972 | 1.708842462 | 1.7007267 | 1.686795153 |
| hsa-miR-576-5p | 1.885115282 | 1.871848637 | 1.657144564 | 1.784737481 | 1.85781713 | 1.743164688 |
| hsa-miR-577 | 1.844523565 | 1.714724859 | 1.92229618 | 1.661765248 | 2.161297064 | 1.800203253 |
| hsa-miR-578 | 1.738223278 | 1.831109431 | 1.869349473 | 1.754224424 | 1.842478917 | 1.684215746 |
| hsa-miR-5787 | 8.020377298 | 7.598424435 | 7.376681502 | 10.81619102 | 2.171501347 | 6.867157185 |
| hsa-miR-579-3p | 1.724569258 | 2.104549173 | 1.875820839 | 1.696839652 | 2.439030123 | 1.765435799 |
| hsa-miR-579-5p | 1.654920827 | 1.823402175 | 1.782266462 | 1.593957305 | 1.780676078 | 1.730047894 |
| hsa-miR-580-3p | 1.669067531 | 1.691096691 | 1.752810247 | 1.808862562 | 1.740392593 | 1.830513528 |
| hsa-miR-580-5p | 1.824087998 | 1.723236368 | 1.865330337 | 1.659042001 | 2.588863783 | 2.167783311 |
| hsa-miR-581 | 1.725670359 | 1.753230946 | 1.799530634 | 1.661900296 | 2.502762883 | 2.172963003 |
| hsa-miR-582-3p | 1.691797317 | 1.736189934 | 1.824853234 | 1.644758548 | 1.836641458 | 1.700983942 |
| hsa-miR-582-5p | 1.968753158 | 1.858852571 | 1.84308277 | 1.824057513 | 1.895058904 | 1.760724232 |
| hsa-miR-583 | 2.17703254 | 1.600203939 | 1.928312378 | 5.495526337 | 1.586273109 | 1.5807046 |
| hsa-miR-584-3p | 2.280015152 | 2.211294006 | 2.082593072 | 2.056804184 | 1.786436698 | 1.968951578 |
| hsa-miR-584-5p | 5.330513157 | 4.667451751 | 4.790932026 | 3.721534386 | 1.949228691 | 6.650567863 |
| hsa-miR-585-3p | 1.806792878 | 1.614312 | 1.736479556 | 1.787942317 | 1.750522571 | 1.676675485 |
| hsa-miR-585-5p | 1.644446914 | 1.639005169 | 1.663448384 | 1.677981517 | 1.668578907 | 1.67810282 |
| hsa-miR-586 | 1.809711489 | 2.170362126 | 2.030263864 | 1.660159297 | 1.909085877 | 1.949750961 |
| hsa-miR-587 | 1.66708611 | 1.714470439 | 1.767239694 | 1.608510453 | 1.709662501 | 1.68837639 |
| hsa-miR-588 | 1.667151658 | 1.702774234 | 1.679851585 | 1.972185484 | 1.897666214 | 1.687256389 |
| hsa-miR-589-3p | 1.671468468 | 1.776401465 | 1.645444566 | 1.648469035 | 1.637579691 | 1.620640876 |
| hsa-miR-589-5p | 1.664833233 | 1.931232446 | 1.777503539 | 1.650395284 | 1.750280531 | 1.712695854 |
| hsa-miR-590-3p | 2.096590117 | 1.976107749 | 1.814006138 | 1.771521514 | 1.720937365 | 1.806342546 |
| hsa-miR-590-5p | 1.917867476 | 1.779568565 | 1.739749609 | 1.750880146 | 1.708287987 | 1.822338985 |
| hsa-miR-591 | 1.809914915 | 1.626870583 | 1.663819456 | 1.797595738 | 1.667646473 | 1.678707174 |
| hsa-miR-592 | 1.779088606 | 1.853688271 | 1.779571813 | 2.205921902 | 2.036415201 | 2.155049073 |
| hsa-miR-593-3p | 1.68054475 | 1.649943222 | 1.854680122 | 1.741224673 | 1.692805117 | 1.844629463 |
| hsa-miR-593-5p | 1.623369011 | 1.688598763 | 1.679146489 | 1.731178777 | 1.893487836 | 1.726294786 |
| hsa-miR-595 | 1.912199962 | 1.858365924 | 1.693489482 | 2.862357689 | 1.678342977 | 1.659296663 |
| hsa-miR-596 | 1.751424182 | 2.133112114 | 1.740929573 | 3.069927452 | 1.951807809 | 1.762243028 |
| hsa-miR-597-3p | 1.735504888 | 1.763846681 | 1.718928704 | 1.683855768 | 1.798844428 | 1.778805818 |
| hsa-miR-597-5p | 1.622963054 | 1.742275788 | 1.601753043 | 1.690188157 | 1.704240457 | 1.737458076 |
| hsa-miR-598-3p | 1.757171881 | 1.960597571 | 1.882787569 | 1.776852011 | 2.362376968 | 1.735947887 |
| hsa-miR-598-5p | 1.870231064 | 1.776674711 | 1.703106925 | 1.812620848 | 1.754896775 | 1.766558134 |
| hsa-miR-599 | 1.799537345 | 1.733389005 | 1.859577974 | 1.744121928 | 1.813387316 | 1.816065577 |
| hsa-miR-600 | 1.691372867 | 1.625270261 | 1.732351505 | 1.717098485 | 1.73083296 | 1.844275454 |
| hsa-miR-601 | 3.863723096 | 1.963508382 | 2.070292944 | 6.239649078 | 1.802332467 | 2.79444554 |
| hsa-miR-602 | 1.765633273 | 1.783871077 | 1.691382111 | 2.10445668 | 1.830299219 | 1.835128135 |
| hsa-miR-603 | 1.697504913 | 1.751473918 | 1.788247587 | 1.658137412 | 1.979579145 | 1.762464338 |
| hsa-miR-604 | 1.639761732 | 1.816480697 | 1.741277837 | 1.641212025 | 1.747761942 | 1.723961577 |
| hsa-miR-605-3p | 1.730574118 | 1.752507608 | 1.710131781 | 1.621878939 | 1.818736146 | 1.752698692 |
| hsa-miR-605-5p | 1.755923293 | 1.727811361 | 1.732317487 | 1.674896165 | 2.012901159 | 1.760753766 |
| hsa-miR-606 | 1.941728261 | 1.749749564 | 1.721333831 | 1.617194406 | 1.813747187 | 1.655452901 |
| hsa-miR-6068 | 8.362094731 | 7.546555943 | 3.297634513 | 3.452589163 | 1.712342943 | 3.229196146 |
| hsa-miR-6069 | 6.239798676 | 7.776228563 | 8.230185263 | 4.064015524 | 11.00112674 | 7.750651704 |
| hsa-miR-607 | 1.688771815 | 1.7871305 | 1.777071568 | 1.74141086 | 2.434921345 | 1.954904622 |
| hsa-miR-6070 | 1.719122293 | 1.801410669 | 1.695759111 | 1.655016051 | 1.617494929 | 1.634718908 |
| hsa-miR-6071 | 1.650445541 | 1.606129 | 1.646088007 | 1.72913302 | 1.706527404 | 1.631016615 |
| hsa-miR-6072 | 1.802660063 | 1.733382655 | 1.770826456 | 1.66798117 | 2.403123819 | 1.765284205 |
| hsa-miR-6073 | 3.745240659 | 5.023024684 | 5.632854405 | 4.656664788 | 2.507243108 | 4.359451751 |
| hsa-miR-6074 | 1.742528477 | 1.980335357 | 1.979091239 | 1.664448836 | 2.469427348 | 1.938301657 |
| hsa-miR-6075 | 2.902989578 | 1.665978242 | 1.680729933 | 1.673176644 | 1.682312845 | 1.636922424 |
| hsa-miR-6076 | 9.487731581 | 8.650579264 | 9.495783279 | 8.119257993 | 2.619453459 | 8.836880252 |
| hsa-miR-6077 | 1.647249959 | 1.647655702 | 1.607034206 | 1.601796483 | 1.685556719 | 1.63647148 |
| hsa-miR-6078 | 1.67778322 | 1.673993992 | 1.683460168 | 1.721359643 | 1.773491619 | 1.684118298 |
| hsa-miR-6079 | 1.690297573 | 1.752677565 | 1.707870599 | 1.638765571 | 1.726931893 | 1.649066657 |
| hsa-miR-608 | 1.690365214 | 1.660506909 | 1.776827123 | 1.660341533 | 1.697910546 | 1.71814541 |
| hsa-miR-6080 | 1.601382617 | 2.086069024 | 1.739281494 | 1.588105579 | 1.822573528 | 1.731309033 |
| hsa-miR-6081 | 1.722364063 | 1.731162419 | 1.733741224 | 1.663155973 | 1.754792681 | 1.687581726 |
| hsa-miR-6082 | 1.726076846 | 1.916842621 | 1.864899403 | 1.647117631 | 1.893561951 | 1.716181102 |
| hsa-miR-6083 | 2.102070005 | 3.088341353 | 2.09309284 | 4.490811096 | 1.913334736 | 4.259121199 |
| hsa-miR-6084 | 1.619015582 | 1.69941755 | 1.786228398 | 1.65025307 | 1.670384861 | 1.729321443 |
| hsa-miR-6085 | 7.113127888 | 7.913468239 | 7.945094919 | 6.117249819 | 3.162988219 | 7.344550595 |
| hsa-miR-6086 | 5.38577391 | 3.823009823 | 3.728349901 | 6.318678399 | 1.74746735 | 2.570448646 |
| hsa-miR-6087 | 9.608623448 | 10.1721535 | 9.08904452 | 9.722755917 | 4.364258419 | 7.629333689 |
| hsa-miR-6088 | 7.532529704 | 7.457379125 | 5.874478448 | 8.606974254 | 9.288029015 | 3.659701594 |
| hsa-miR-6089 | 11.72261136 | 11.92048522 | 11.12407667 | 9.14540094 | 9.526195614 | 9.668715009 |
| hsa-miR-609 | 1.805299585 | 1.653564429 | 1.688819923 | 1.692811882 | 1.860860614 | 1.820048725 |
| hsa-miR-6090 | 10.70662332 | 10.94842358 | 10.35873601 | 10.87207815 | 10.96663648 | 7.893939214 |
| hsa-miR-610 | 4.026295781 | 1.864815966 | 1.866445417 | 2.019894469 | 1.727248761 | 2.062943279 |
| hsa-miR-611 | 1.813339235 | 1.697555425 | 1.72736619 | 1.701637393 | 1.698854824 | 1.627899221 |
| hsa-miR-612 | 1.649224739 | 1.789483223 | 1.924411631 | 1.729171914 | 1.884175967 | 1.79461754 |
| hsa-miR-6124 | 8.555864817 | 8.74043007 | 9.281458005 | 10.85352577 | 2.265337561 | 8.679711023 |
| hsa-miR-6125 | 10.84805895 | 10.59693864 | 8.876600088 | 5.662948938 | 2.254970667 | 6.851687636 |
| hsa-miR-6126 | 7.678198104 | 8.317358393 | 5.5976953 | 7.798495545 | 1.995902297 | 4.696536001 |
| hsa-miR-6127 | 9.5502189 | 10.60740659 | 10.72209693 | 9.179105545 | 9.594262831 | 11.91215073 |
| hsa-miR-6128 | 1.677468477 | 1.714149834 | 1.844648592 | 1.655952136 | 1.672100606 | 1.665643977 |
| hsa-miR-6129 | 1.843711681 | 1.934590493 | 1.710171138 | 1.892113636 | 1.842634412 | 1.845007797 |
| hsa-miR-613 | 1.698281756 | 1.83636009 | 2.401828455 | 1.752892349 | 2.051763255 | 1.776700138 |
| hsa-miR-6130 | 2.208762694 | 1.684726889 | 1.803899401 | 1.937571336 | 2.474993251 | 1.743119864 |
| hsa-miR-6131 | 1.747251203 | 2.113733717 | 2.052030685 | 2.156503825 | 1.876801523 | 1.852390913 |
| hsa-miR-6132 | 1.868231055 | 1.741735346 | 1.690461626 | 1.993196262 | 1.798388181 | 1.670056053 |
| hsa-miR-6133 | 3.352466637 | 1.964568678 | 2.043758176 | 7.793988331 | 1.670840543 | 2.843786056 |
| hsa-miR-6134 | 1.709016253 | 1.762230822 | 1.808367235 | 1.848212258 | 2.063996733 | 1.943107223 |
| hsa-miR-614 | 1.737580626 | 1.726262563 | 1.745244296 | 1.664450533 | 1.699470892 | 1.745810827 |
| hsa-miR-615-3p | 1.857246607 | 1.762836689 | 1.998902317 | 1.788735455 | 1.807111052 | 1.93512548 |
| hsa-miR-615-5p | 1.728435218 | 1.65191198 | 1.773715814 | 1.692946521 | 1.756506934 | 1.806640725 |
| hsa-miR-616-3p | 1.688988642 | 1.648714249 | 1.755080306 | 1.854212347 | 1.742456312 | 1.631306063 |
| hsa-miR-616-5p | 1.791992998 | 1.752978181 | 1.651861863 | 1.644009096 | 1.754895851 | 1.608306494 |
| hsa-miR-6165 | 7.702692043 | 8.440052071 | 8.682316143 | 7.945972229 | 6.002536802 | 8.400764907 |
| hsa-miR-617 | 1.805070294 | 1.731215318 | 1.667023793 | 2.35395537 | 1.791498056 | 1.689611228 |
| hsa-miR-618 | 1.686490219 | 1.949688504 | 2.072539428 | 1.686754215 | 1.931898359 | 1.899015868 |
| hsa-miR-619-3p | 1.776256598 | 1.761951315 | 1.716046984 | 1.778918278 | 1.739416238 | 1.699812712 |
| hsa-miR-619-5p | 3.636901123 | 2.954340854 | 4.567013802 | 2.397291767 | 1.831998149 | 2.204717628 |
| hsa-miR-620 | 1.640540867 | 1.908430105 | 1.792770323 | 1.705316857 | 1.737957 | 1.72529035 |
| hsa-miR-621 | 1.692704068 | 1.708587927 | 1.822457629 | 1.610277389 | 1.665252286 | 1.67781636 |
| hsa-miR-622 | 9.984471001 | 1.7553944 | 6.918229874 | 4.133801357 | 1.741117558 | 1.780656103 |
| hsa-miR-623 | 1.968189177 | 1.676520161 | 1.743847542 | 3.418134914 | 1.582219081 | 1.728265327 |
| hsa-miR-624-3p | 1.878981749 | 1.97057055 | 1.702354427 | 1.763561759 | 1.992653216 | 1.73877212 |
| hsa-miR-624-5p | 1.736262425 | 1.774786183 | 1.997509054 | 1.737359553 | 2.225218169 | 3.045852394 |
| hsa-miR-625-3p | 1.980554517 | 1.831047872 | 1.986837784 | 1.920984108 | 2.208219663 | 2.730602959 |
| hsa-miR-625-5p | 1.80392003 | 1.730740713 | 1.95398594 | 1.804116529 | 1.847990357 | 1.733743604 |
| hsa-miR-626 | 1.701904221 | 1.877922001 | 2.027580055 | 1.739497233 | 2.363158985 | 1.882697138 |
| hsa-miR-627-3p | 1.837019674 | 1.81375401 | 2.051029574 | 1.725895291 | 3.061626763 | 1.777160011 |
| hsa-miR-627-5p | 2.012238632 | 1.7410107 | 1.781279288 | 1.865099482 | 2.091111985 | 2.337745427 |
| hsa-miR-628-3p | 1.652532701 | 1.798764248 | 1.78119875 | 1.623700757 | 1.896980959 | 1.731554917 |
| hsa-miR-628-5p | 1.742342175 | 1.789555152 | 1.939458903 | 1.681588292 | 2.152141671 | 1.825487712 |
| hsa-miR-629-3p | 1.705165782 | 1.818555557 | 1.83694867 | 1.803429995 | 2.059250766 | 1.643717445 |
| hsa-miR-629-5p | 1.872979595 | 1.679274 | 1.772742451 | 1.931072151 | 1.678019977 | 1.732350889 |
| hsa-miR-630 | 11.14262143 | 10.53636218 | 10.75482434 | 11.90544671 | 1.99087122 | 7.885651719 |
| hsa-miR-631 | 4.741749052 | 4.195570876 | 4.222789544 | 3.260828086 | 3.041601512 | 4.214085269 |
| hsa-miR-632 | 2.239293486 | 1.762628612 | 1.787319933 | 1.82241431 | 1.707790827 | 2.236227081 |
| hsa-miR-633 | 1.822185996 | 1.828109908 | 1.700680283 | 1.710626176 | 1.999943293 | 1.697620746 |
| hsa-miR-634 | 2.289073253 | 1.929015507 | 2.394428623 | 2.503069544 | 2.160227041 | 2.343238385 |
| hsa-miR-635 | 1.655345279 | 1.741192217 | 1.999707877 | 1.619397083 | 1.955569008 | 1.983141054 |
| hsa-miR-636 | 3.398498916 | 5.155354293 | 4.944671013 | 3.139676031 | 4.211314755 | 3.570513288 |
| hsa-miR-637 | 1.765348051 | 1.676917193 | 1.709982821 | 1.690530176 | 1.835053334 | 1.680067485 |
| hsa-miR-638 | 9.130345079 | 8.62100926 | 6.124596589 | 5.029948335 | 2.17983916 | 3.757697996 |
| hsa-miR-639 | 1.622419364 | 1.770609437 | 1.742868242 | 1.660525802 | 1.830033871 | 1.825310609 |
| hsa-miR-640 | 1.757302161 | 1.824320521 | 1.738262543 | 1.763799433 | 1.778444524 | 1.743876468 |
| hsa-miR-641 | 1.61370553 | 1.802471895 | 1.749327423 | 1.615178454 | 1.791179964 | 1.735369422 |
| hsa-miR-642a-3p | 7.890441085 | 8.572197277 | 8.61207171 | 7.456156551 | 3.787693806 | 8.127325438 |
| hsa-miR-642a-5p | 1.707666919 | 1.858012964 | 1.85355635 | 1.70614413 | 2.22267091 | 1.92406153 |
| hsa-miR-642b-3p | 5.7844982 | 4.963782017 | 3.511752518 | 7.088408817 | 5.042655728 | 7.940595921 |
| hsa-miR-642b-5p | 1.860099305 | 1.86993326 | 1.775766339 | 1.75824213 | 2.043295766 | 1.696365449 |
| hsa-miR-643 | 1.706163869 | 1.733041184 | 1.69339613 | 1.604957637 | 1.737623483 | 1.755217824 |
| hsa-miR-644a | 1.639746167 | 1.696291062 | 1.67006608 | 1.634004427 | 1.621364453 | 1.744696869 |
| hsa-miR-645 | 1.626716169 | 1.82126431 | 1.780500317 | 1.650380201 | 1.838896855 | 1.711522239 |
| hsa-miR-646 | 1.686956109 | 1.701810782 | 1.623058987 | 1.600346378 | 1.642073275 | 1.662679048 |
| hsa-miR-647 | 1.670859124 | 1.727116247 | 1.727526542 | 1.658276266 | 2.088532903 | 1.780943452 |
| hsa-miR-648 | 1.696068224 | 1.64956015 | 1.657161267 | 1.690180063 | 1.675282478 | 1.61751811 |
| hsa-miR-649 | 1.676995192 | 1.666850818 | 1.767097429 | 1.676440724 | 1.694700771 | 1.774860571 |
| hsa-miR-6499-3p | 1.731638798 | 2.138880253 | 1.892724715 | 1.656666985 | 1.961267415 | 1.800900052 |
| hsa-miR-6499-5p | 1.701050175 | 1.748942706 | 1.73733701 | 1.646022915 | 1.880222466 | 1.809955267 |
| hsa-miR-650 | 2.078720408 | 1.745128444 | 1.645320629 | 1.649312453 | 1.750878419 | 1.717266003 |
| hsa-miR-6500-3p | 1.732175494 | 1.77683058 | 1.701571354 | 2.526473186 | 1.703252271 | 1.755928298 |
| hsa-miR-6500-5p | 1.803406006 | 2.016661563 | 1.717284388 | 1.70076044 | 1.734544805 | 1.691510891 |
| hsa-miR-6501-3p | 1.661382599 | 1.694782828 | 1.712286921 | 1.627981473 | 1.743837697 | 1.758066126 |
| hsa-miR-6501-5p | 1.709396579 | 1.72470971 | 1.603499069 | 1.670966274 | 1.791645527 | 1.61453459 |
| hsa-miR-6502-3p | 1.710579847 | 1.94118483 | 1.745402457 | 1.648643685 | 2.307019662 | 1.800644418 |
| hsa-miR-6502-5p | 1.711094 | 1.737807833 | 1.951378632 | 1.699795512 | 3.026079402 | 2.002900632 |
| hsa-miR-6503-3p | 1.740522569 | 1.880205307 | 1.754093747 | 1.75375314 | 1.739172376 | 1.851259228 |
| hsa-miR-6503-5p | 1.915165949 | 1.787760385 | 1.942907028 | 1.720530219 | 1.72579312 | 1.938140445 |
| hsa-miR-6504-3p | 1.741013819 | 2.000397066 | 1.839624771 | 1.717250317 | 1.79277049 | 1.722680587 |
| hsa-miR-6504-5p | 1.684044177 | 1.770717676 | 1.829911393 | 1.654177941 | 3.065427448 | 1.888095387 |
| hsa-miR-6505-3p | 1.762191998 | 1.750407862 | 1.765356616 | 1.66815789 | 1.79155444 | 1.767242585 |
| hsa-miR-6505-5p | 1.792505746 | 1.953399595 | 1.710524617 | 1.717531647 | 1.662839375 | 1.781058266 |
| hsa-miR-6506-3p | 1.696279435 | 1.738253059 | 1.859009665 | 1.64169507 | 2.269137211 | 1.740225346 |
| hsa-miR-6506-5p | 1.676029913 | 2.236121242 | 1.777275419 | 1.673666502 | 2.103239725 | 1.750732746 |
| hsa-miR-6507-3p | 1.82161373 | 1.82592349 | 1.857932396 | 1.778378997 | 1.981911778 | 1.897103638 |
| hsa-miR-6507-5p | 1.72213968 | 1.833357392 | 1.776603945 | 1.883024164 | 1.840511128 | 1.876845083 |
| hsa-miR-6508-3p | 1.671701336 | 1.850303989 | 2.016375127 | 1.685382952 | 2.70588424 | 2.044703198 |
| hsa-miR-6508-5p | 5.528678308 | 7.1055018 | 7.647344326 | 3.886748259 | 10.43300229 | 7.320531131 |
| hsa-miR-6509-3p | 1.83914643 | 1.740766362 | 1.848829794 | 1.705170299 | 2.023128559 | 2.098881911 |
| hsa-miR-6509-5p | 1.838651317 | 2.052710249 | 1.756317487 | 2.116794257 | 1.703559064 | 1.981574822 |
| hsa-miR-651-3p | 1.692149685 | 1.70556364 | 1.698867333 | 1.710809933 | 1.877694548 | 1.796531033 |
| hsa-miR-651-5p | 1.886832832 | 1.688151745 | 1.696632006 | 1.657494384 | 1.933774167 | 1.680098406 |
| hsa-miR-6510-3p | 1.721608846 | 1.969758986 | 1.819261153 | 1.637782652 | 1.893540541 | 1.766423886 |
| hsa-miR-6510-5p | 11.6632117 | 10.56648117 | 11.17732767 | 12.91246917 | 7.767630907 | 10.96389806 |
| hsa-miR-6511a-3p | 1.771905717 | 1.748864951 | 1.67260315 | 1.751913026 | 1.736948193 | 1.655620419 |
| hsa-miR-6511a-5p | 6.157135979 | 1.803571904 | 3.439473996 | 2.579963458 | 1.649894679 | 1.722075681 |
| hsa-miR-6511b-3p | 1.861172041 | 1.792258662 | 1.763093799 | 1.965128621 | 1.801882448 | 1.774092665 |
| hsa-miR-6511b-5p | 7.80202968 | 2.013352799 | 5.921516854 | 3.73116939 | 1.749816636 | 1.872983751 |
| hsa-miR-6512-3p | 1.682544091 | 1.804986497 | 1.768183349 | 1.740857328 | 1.821903008 | 1.738411089 |
| hsa-miR-6512-5p | 1.80101173 | 1.845622566 | 1.811873174 | 1.992859583 | 1.824041619 | 1.834270644 |
| hsa-miR-6513-3p | 1.638002027 | 1.72428146 | 1.854643481 | 1.616098167 | 1.970480108 | 1.791653103 |
| hsa-miR-6513-5p | 1.703342251 | 1.788286794 | 1.926320233 | 1.667026584 | 2.053132802 | 1.84792758 |
| hsa-miR-6514-3p | 1.691505177 | 1.683837277 | 1.950404758 | 1.758474985 | 1.793093284 | 1.764949492 |
| hsa-miR-6514-5p | 1.622265226 | 1.752771094 | 1.701366641 | 1.644454934 | 1.809716522 | 1.679674961 |
| hsa-miR-6515-3p | 5.522442329 | 6.676630942 | 7.007706579 | 3.594785742 | 9.987830898 | 6.609530873 |
| hsa-miR-6515-5p | 1.781705393 | 1.982846707 | 2.168815885 | 1.837387666 | 1.904730224 | 2.202541903 |
| hsa-miR-6516-3p | 2.861880585 | 1.767754797 | 2.146128512 | 1.786573424 | 1.899649024 | 1.905994259 |
| hsa-miR-6516-5p | 2.027946023 | 2.007389593 | 1.831537398 | 1.722125248 | 1.838660286 | 1.730715029 |
| hsa-miR-652-3p | 2.983557225 | 2.817771208 | 3.762835706 | 3.294193114 | 1.778974548 | 3.91792008 |
| hsa-miR-652-5p | 2.292703676 | 1.812898998 | 1.861657949 | 3.357985365 | 1.785923646 | 2.419921338 |
| hsa-miR-653-3p | 1.714531812 | 1.775238145 | 2.203665536 | 1.713619451 | 2.075372436 | 1.746098183 |
| hsa-miR-653-5p | 1.834377182 | 1.726207545 | 1.860087086 | 1.655170811 | 1.770514088 | 1.722576297 |
| hsa-miR-654-3p | 1.637018635 | 1.876863287 | 1.733330204 | 1.631301239 | 1.926537816 | 1.793246517 |
| hsa-miR-654-5p | 3.581565699 | 2.881359212 | 2.123607361 | 1.784078755 | 1.872031388 | 1.864660436 |
| hsa-miR-655-3p | 1.873212658 | 1.716454446 | 1.845308753 | 1.751825833 | 3.242853629 | 1.732238542 |
| hsa-miR-655-5p | 2.309296898 | 1.80008782 | 2.396086286 | 1.79841795 | 2.63974387 | 1.78585131 |
| hsa-miR-656-3p | 2.019679582 | 2.019060447 | 1.787726914 | 1.856116455 | 1.991257921 | 1.984707496 |
| hsa-miR-656-5p | 1.652304899 | 1.692085247 | 1.693923484 | 1.636170595 | 1.788009338 | 1.831653545 |
| hsa-miR-657 | 1.728381928 | 1.731412743 | 2.239876996 | 1.682902766 | 1.951221451 | 1.790629403 |
| hsa-miR-658 | 1.74405008 | 1.759230436 | 1.687113366 | 1.707066987 | 1.723551064 | 1.663516465 |
| hsa-miR-659-3p | 1.859980659 | 1.803479565 | 1.718687915 | 4.289441338 | 1.789975537 | 4.246937728 |
| hsa-miR-659-5p | 1.870115864 | 1.754672237 | 1.641261249 | 1.661728534 | 1.702355365 | 1.698141741 |
| hsa-miR-660-3p | 1.874635839 | 1.757200626 | 1.785181535 | 1.776416119 | 1.909743832 | 1.780889337 |
| hsa-miR-660-5p | 3.272002354 | 1.805069318 | 2.020205137 | 5.956134951 | 2.443748253 | 8.76220538 |
| hsa-miR-661 | 1.678250374 | 1.737591818 | 1.677223216 | 1.672924726 | 1.64178396 | 1.644638685 |
| hsa-miR-662 | 1.803857724 | 1.783151622 | 1.733996488 | 1.786976108 | 1.688440535 | 1.820894286 |
| hsa-miR-663a | 4.187308626 | 1.681901585 | 1.904018777 | 3.273127609 | 1.794907934 | 1.714186162 |
| hsa-miR-663b | 1.756232973 | 1.784575748 | 1.707244264 | 1.650989891 | 1.80557063 | 1.838218324 |
| hsa-miR-664a-3p | 2.252610991 | 2.390848552 | 2.091581949 | 2.103083254 | 2.693619499 | 2.404495576 |
| hsa-miR-664a-5p | 2.937246063 | 1.760215078 | 1.857260767 | 1.673769721 | 1.78793924 | 1.823655032 |
| hsa-miR-664b-3p | 4.472177689 | 2.243626783 | 1.941705308 | 1.902993092 | 1.774492377 | 1.79703632 |
| hsa-miR-664b-5p | 6.102250241 | 1.86830354 | 1.788900696 | 1.819116499 | 1.836690536 | 1.906229255 |
| hsa-miR-665 | 1.843258214 | 1.720549671 | 1.845672088 | 2.143186893 | 1.676449761 | 1.820252913 |
| hsa-miR-668-3p | 1.685997593 | 1.809674486 | 2.082502477 | 1.71666719 | 1.850401577 | 1.713564047 |
| hsa-miR-668-5p | 1.784312455 | 1.766431535 | 1.684406493 | 1.736751578 | 1.688356652 | 1.65535085 |
| hsa-miR-670-3p | 2.070633013 | 2.016904872 | 2.063590131 | 1.730805032 | 1.991106229 | 1.745190642 |
| hsa-miR-670-5p | 1.690956926 | 1.785972579 | 1.866276922 | 1.745496039 | 2.084921939 | 2.122468295 |
| hsa-miR-671-3p | 1.868734778 | 1.636247036 | 1.679595717 | 1.723798274 | 1.690130724 | 1.642754732 |
| hsa-miR-671-5p | 7.821082974 | 9.361622969 | 9.4162628 | 6.10588962 | 3.524211204 | 9.404194469 |
| hsa-miR-6715a-3p | 1.653987388 | 1.772868375 | 1.670492735 | 1.650430592 | 1.758449791 | 1.648595968 |
| hsa-miR-6715b-3p | 1.686652889 | 1.696356209 | 1.696749342 | 1.682334707 | 2.038003483 | 1.750844096 |
| hsa-miR-6715b-5p | 1.811030693 | 1.694656358 | 1.820180377 | 1.675454368 | 1.770437121 | 1.749977052 |
| hsa-miR-6716-3p | 1.710581251 | 2.005848961 | 1.840806238 | 1.612554007 | 3.036848264 | 1.839015029 |
| hsa-miR-6716-5p | 1.777892244 | 1.870979203 | 1.777823465 | 3.049066399 | 1.601778466 | 1.701405308 |
| hsa-miR-6717-5p | 2.231476219 | 1.748312534 | 1.714281576 | 2.369309237 | 1.699470892 | 1.734843438 |
| hsa-miR-6718-5p | 1.660297969 | 1.654942451 | 1.896089997 | 1.818746595 | 1.735788611 | 1.71251411 |
| hsa-miR-6719-3p | 1.949193725 | 2.012879707 | 2.19231899 | 1.739571644 | 1.974096161 | 1.781001461 |
| hsa-miR-6720-3p | 1.730535409 | 1.770432551 | 1.683163287 | 1.657682784 | 1.771529817 | 1.782847891 |
| hsa-miR-6720-5p | 1.559208644 | 1.754464393 | 1.607214081 | 1.767294905 | 1.645885603 | 1.668937387 |
| hsa-miR-6721-5p | 1.823962348 | 1.766367944 | 1.743866644 | 1.687753872 | 1.776083329 | 1.729537901 |
| hsa-miR-6722-3p | 2.449958385 | 1.810119249 | 1.916083947 | 3.571870116 | 1.754673618 | 1.739896789 |
| hsa-miR-6722-5p | 1.806751769 | 1.809179 | 1.692149215 | 1.857187329 | 1.80162944 | 1.71645271 |
| hsa-miR-6723-5p | 1.970077239 | 1.9679729 | 1.749941847 | 2.43804642 | 1.685121846 | 1.720536984 |
| hsa-miR-6724-5p | 7.399689856 | 5.698863401 | 6.403933383 | 6.74544588 | 1.763564183 | 5.648033105 |
| hsa-miR-6726-3p | 1.678376307 | 1.709594531 | 1.683245345 | 1.740346937 | 1.82260856 | 1.681727553 |
| hsa-miR-6726-5p | 1.966767838 | 2.182147764 | 1.76440889 | 2.06784212 | 1.924516534 | 1.9680148 |
| hsa-miR-6727-3p | 1.690149581 | 1.692976493 | 2.018688681 | 1.662257059 | 1.848536322 | 1.798530327 |
| hsa-miR-6727-5p | 4.063282647 | 1.78746348 | 1.819224547 | 6.654677771 | 1.75741937 | 1.672820293 |
| hsa-miR-6728-3p | 1.773169701 | 1.941621302 | 1.806327554 | 1.878148837 | 1.75964639 | 1.66571073 |
| hsa-miR-6728-5p | 4.487524789 | 6.272432016 | 5.512351876 | 5.358423076 | 1.682501144 | 5.476467635 |
| hsa-miR-6729-3p | 1.73972081 | 1.769994364 | 1.717446869 | 1.632736146 | 2.033893041 | 1.772877497 |
| hsa-miR-6729-5p | 2.109442219 | 2.094770135 | 2.038657674 | 1.865471186 | 1.773077583 | 2.140881921 |
| hsa-miR-6730-3p | 2.153114882 | 2.538497564 | 3.224725935 | 1.96387345 | 1.895060403 | 2.961255572 |
| hsa-miR-6730-5p | 2.393251286 | 1.794921972 | 2.096224826 | 2.808323862 | 1.69691575 | 1.822173302 |
| hsa-miR-6731-3p | 3.132820744 | 3.820304468 | 3.748375878 | 2.745493746 | 5.147420044 | 3.622777443 |
| hsa-miR-6731-5p | 1.873022299 | 1.875950981 | 1.671097709 | 1.777851563 | 1.855627045 | 1.765434439 |
| hsa-miR-6732-3p | 2.847550947 | 4.192283613 | 2.882537719 | 2.417102808 | 4.700471939 | 3.211451691 |
| hsa-miR-6732-5p | 1.715088237 | 1.648767913 | 1.763003039 | 1.902061494 | 1.782086011 | 4.007499251 |
| hsa-miR-6733-3p | 1.960634592 | 1.757559807 | 1.660524097 | 1.869192449 | 1.868055392 | 1.793421592 |
| hsa-miR-6733-5p | 1.761418565 | 1.864946033 | 1.955021976 | 1.666797336 | 2.179704912 | 2.226879646 |
| hsa-miR-6734-3p | 1.70698072 | 1.661786539 | 1.883276988 | 1.719628099 | 1.731786365 | 1.828160519 |
| hsa-miR-6734-5p | 7.248452728 | 1.947258758 | 4.056790472 | 4.769891815 | 1.85579865 | 3.371110931 |
| hsa-miR-6735-3p | 1.676834059 | 1.973245871 | 1.810369594 | 1.653007806 | 1.962730981 | 1.86644568 |
| hsa-miR-6735-5p | 1.606174967 | 1.75700405 | 1.701040427 | 1.910563716 | 1.721947676 | 1.799501718 |
| hsa-miR-6736-3p | 1.713498047 | 1.771847641 | 1.810267509 | 1.741078739 | 1.926146533 | 1.692071813 |
| hsa-miR-6736-5p | 1.746614566 | 1.852312176 | 1.915059909 | 1.717612593 | 2.221409766 | 1.818658222 |
| hsa-miR-6737-3p | 5.181731024 | 7.009118045 | 7.679351399 | 3.676599509 | 10.40866495 | 7.118654658 |
| hsa-miR-6737-5p | 1.788250819 | 1.76564805 | 1.687324298 | 1.875691243 | 1.916865923 | 1.682661192 |
| hsa-miR-6738-3p | 1.715993546 | 1.8320477 | 1.820527058 | 1.683954996 | 1.802776639 | 1.76784782 |
| hsa-miR-6738-5p | 2.984984283 | 2.028671015 | 1.936516524 | 4.623218598 | 1.888334091 | 3.865532681 |
| hsa-miR-6739-3p | 1.706388897 | 1.918580298 | 1.750378804 | 1.803158517 | 1.855162613 | 1.839442821 |
| hsa-miR-6739-5p | 7.889275485 | 9.07910016 | 8.995904799 | 9.607162419 | 3.308032395 | 7.261025441 |
| hsa-miR-6740-3p | 1.806427518 | 1.725938001 | 1.798154434 | 1.729268033 | 1.800593831 | 1.810894739 |
| hsa-miR-6740-5p | 10.44621369 | 8.955918593 | 10.27926236 | 11.85128742 | 2.538769839 | 9.567028857 |
| hsa-miR-6741-3p | 1.843741463 | 1.842113803 | 1.982247973 | 1.82981004 | 2.469824658 | 2.212547785 |
| hsa-miR-6741-5p | 6.169736087 | 3.866419722 | 5.689315108 | 4.557268324 | 2.429937587 | 5.247128807 |
| hsa-miR-6742-3p | 1.747552651 | 1.825006793 | 2.005375736 | 1.695682585 | 1.924779008 | 1.739829271 |
| hsa-miR-6742-5p | 1.590208385 | 1.745174288 | 1.736599642 | 1.585824064 | 2.243259726 | 1.719373056 |
| hsa-miR-6743-3p | 2.314016557 | 2.145920823 | 2.25647875 | 1.886000145 | 2.613442814 | 1.868243308 |
| hsa-miR-6743-5p | 1.688816939 | 1.720057796 | 1.994353133 | 1.771930955 | 1.853014722 | 1.865126419 |
| hsa-miR-6744-3p | 1.73129367 | 1.765579744 | 1.738116418 | 1.664406421 | 1.810759921 | 1.73512045 |
| hsa-miR-6744-5p | 1.622328605 | 1.704006588 | 1.72735765 | 1.619895388 | 1.781600565 | 1.735091234 |
| hsa-miR-6745 | 3.925572779 | 1.9648003 | 3.785603534 | 5.342465545 | 1.725188158 | 2.027137669 |
| hsa-miR-6746-3p | 1.701728866 | 1.788983736 | 1.766188703 | 1.646686403 | 1.697977714 | 1.840540844 |
| hsa-miR-6746-5p | 1.747717399 | 1.787764096 | 1.794958152 | 1.908567001 | 1.62806838 | 1.658217298 |
| hsa-miR-6747-3p | 1.678923155 | 1.846616022 | 1.641945154 | 1.675252698 | 1.800702968 | 1.704468083 |
| hsa-miR-6747-5p | 3.465636127 | 2.068277217 | 2.38510398 | 4.494588566 | 1.695129245 | 1.936100744 |
| hsa-miR-6748-3p | 1.678990766 | 2.073851339 | 1.729226295 | 1.721190713 | 1.772598644 | 1.752208431 |
| hsa-miR-6748-5p | 5.53048094 | 2.037123591 | 2.005889944 | 6.058036803 | 1.855310837 | 2.065587789 |
| hsa-miR-6749-3p | 1.571079573 | 1.691376056 | 1.656288004 | 1.635762321 | 1.672719012 | 1.734088335 |
| hsa-miR-6749-5p | 8.15012866 | 8.626897011 | 8.324853051 | 7.594190143 | 2.547515796 | 7.658611626 |
| hsa-miR-675-3p | 1.742452424 | 1.966846544 | 1.711924825 | 1.666806408 | 1.820703379 | 1.724578254 |
| hsa-miR-675-5p | 1.764449685 | 1.733147651 | 1.663782419 | 1.652483577 | 1.675034901 | 1.66934763 |
| hsa-miR-6750-3p | 1.658335016 | 1.709720449 | 1.768907997 | 1.64011397 | 1.873778577 | 1.8201126 |
| hsa-miR-6750-5p | 1.651445992 | 1.874912821 | 1.931679026 | 1.73022329 | 1.777982216 | 1.775217639 |
| hsa-miR-6751-3p | 1.910647927 | 2.190553287 | 2.016455228 | 1.665871864 | 1.886677501 | 1.852684682 |
| hsa-miR-6751-5p | 1.686897465 | 1.988451629 | 1.965702806 | 2.189739335 | 1.832312395 | 1.862947423 |
| hsa-miR-6752-3p | 3.31309886 | 4.122654151 | 3.576720849 | 3.153575572 | 4.699404137 | 4.034393041 |
| hsa-miR-6752-5p | 4.224780615 | 2.64561154 | 1.975298586 | 5.160253681 | 2.024358974 | 2.647870838 |
| hsa-miR-6753-3p | 1.755907234 | 1.674644075 | 1.91347186 | 1.735234326 | 1.748625675 | 1.697849024 |
| hsa-miR-6753-5p | 3.870489334 | 3.556769617 | 3.740999642 | 3.009318733 | 1.77963373 | 2.039838388 |
| hsa-miR-6754-3p | 1.728397666 | 1.805399848 | 1.788236454 | 1.702751923 | 2.020714732 | 1.798405038 |
| hsa-miR-6754-5p | 2.530515533 | 2.107783633 | 3.380832507 | 2.295391408 | 1.889273485 | 5.275173192 |
| hsa-miR-6755-3p | 1.741437126 | 1.834768374 | 1.971646915 | 1.846671403 | 1.89265412 | 1.892858906 |
| hsa-miR-6755-5p | 1.72968058 | 1.743980742 | 1.651475644 | 1.633535536 | 1.870515621 | 1.639052724 |
| hsa-miR-6756-3p | 2.566031257 | 2.870872894 | 3.314726274 | 2.42503893 | 1.918161226 | 2.506766581 |
| hsa-miR-6756-5p | 6.644829567 | 4.94902964 | 5.518016418 | 5.889267831 | 1.806137036 | 6.363453527 |
| hsa-miR-6757-3p | 1.754468277 | 2.150082107 | 2.150655803 | 1.796437804 | 2.042202971 | 1.87457136 |
| hsa-miR-6757-5p | 4.09548975 | 3.370528369 | 2.143717407 | 4.018234553 | 1.873396025 | 5.372866194 |
| hsa-miR-6758-3p | 1.813082776 | 1.763792636 | 1.722825567 | 1.631910481 | 1.743543895 | 1.629581038 |
| hsa-miR-6758-5p | 3.537959471 | 2.482058326 | 2.425014642 | 5.018507445 | 1.927563573 | 1.874203431 |
| hsa-miR-6759-3p | 2.896016786 | 3.81531019 | 3.882135381 | 2.620274371 | 4.485962082 | 3.339571641 |
| hsa-miR-6759-5p | 1.69644176 | 1.849432111 | 1.799043027 | 1.665283004 | 1.937642471 | 2.053687495 |
| hsa-miR-676-3p | 1.709311455 | 1.824177762 | 1.765767184 | 2.02553867 | 2.139083473 | 1.865681176 |
| hsa-miR-676-5p | 1.669061307 | 1.849329633 | 1.702202792 | 1.59509115 | 1.751768993 | 1.796719954 |
| hsa-miR-6760-3p | 4.143987704 | 5.297761164 | 6.400166164 | 3.176091986 | 8.032798829 | 5.772992584 |
| hsa-miR-6760-5p | 9.795972683 | 2.22947684 | 2.305630086 | 4.477062206 | 1.863614298 | 2.651158688 |
| hsa-miR-6761-3p | 1.694458941 | 1.967837728 | 1.861042835 | 1.714565611 | 1.779625195 | 1.826881781 |
| hsa-miR-6761-5p | 1.693300333 | 1.715356959 | 1.64725954 | 1.63210305 | 1.762428512 | 1.699113265 |
| hsa-miR-6762-3p | 1.591935519 | 2.19446448 | 1.798580902 | 1.605244336 | 2.100370634 | 1.724813637 |
| hsa-miR-6762-5p | 1.772456566 | 1.672038619 | 1.646847557 | 1.757871771 | 1.647112457 | 1.635398995 |
| hsa-miR-6763-3p | 4.074965455 | 4.391323804 | 5.172918329 | 3.107951235 | 7.05777072 | 4.756394601 |
| hsa-miR-6763-5p | 7.152628365 | 4.771566983 | 4.10410681 | 5.39652158 | 2.16104311 | 4.451103328 |
| hsa-miR-6764-3p | 1.698316493 | 1.813929413 | 1.69092591 | 1.737746707 | 1.787730134 | 1.732779653 |
| hsa-miR-6764-5p | 1.642974399 | 1.689959368 | 1.659692982 | 1.679330024 | 1.639182265 | 1.65460498 |
| hsa-miR-6765-3p | 3.512467873 | 3.509705443 | 4.806613345 | 2.898894477 | 3.976819106 | 3.782461854 |
| hsa-miR-6765-5p | 1.815062903 | 2.547449844 | 2.00590761 | 3.214193772 | 1.801175031 | 1.872808203 |
| hsa-miR-6766-3p | 3.221456999 | 3.010937287 | 4.655422605 | 2.657003579 | 3.243933881 | 3.422663697 |
| hsa-miR-6766-5p | 1.725243028 | 1.805149872 | 1.900514862 | 1.72916491 | 2.812309762 | 1.894078479 |
| hsa-miR-6767-3p | 1.756671934 | 1.735105451 | 1.978353821 | 1.744256966 | 2.28441344 | 1.668728704 |
| hsa-miR-6767-5p | 2.035982303 | 1.745074602 | 1.999665586 | 2.657858048 | 1.831216564 | 1.985796842 |
| hsa-miR-6768-3p | 1.672855464 | 1.987323373 | 1.736977707 | 1.638995981 | 1.825105404 | 1.754219501 |
| hsa-miR-6768-5p | 1.790563349 | 1.871776794 | 1.648974476 | 2.140212691 | 1.78703679 | 1.715457338 |
| hsa-miR-6769a-3p | 1.656023746 | 1.705203664 | 1.832407681 | 1.59766851 | 1.772287881 | 1.804878875 |
| hsa-miR-6769a-5p | 1.758923649 | 1.771972935 | 1.81400559 | 2.808237732 | 2.266976597 | 2.146635872 |
| hsa-miR-6769b-3p | 2.071300786 | 3.511719067 | 5.171267083 | 1.95289873 | 4.709548819 | 2.982652299 |
| hsa-miR-6769b-5p | 7.904564452 | 7.440623083 | 8.832032823 | 9.723322444 | 2.68565416 | 7.885013063 |
| hsa-miR-6770-3p | 1.708813518 | 1.748069569 | 1.787216024 | 1.693342151 | 1.720675768 | 1.672618716 |
| hsa-miR-6770-5p | 1.760945363 | 1.626028978 | 1.752074878 | 1.673848664 | 1.713689685 | 1.666325659 |
| hsa-miR-6771-3p | 1.703122018 | 1.771150694 | 1.660913554 | 1.658689882 | 1.716601449 | 1.736464365 |
| hsa-miR-6771-5p | 1.609911394 | 1.7542691 | 1.736439883 | 1.678306378 | 1.789123989 | 1.762081027 |
| hsa-miR-6772-3p | 1.704289513 | 1.665168811 | 1.818221878 | 1.668637171 | 1.847675304 | 1.765922362 |
| hsa-miR-6772-5p | 1.805783645 | 1.866434023 | 1.837085608 | 3.356434244 | 1.750559536 | 1.841854836 |
| hsa-miR-6773-3p | 1.707254943 | 2.29902617 | 1.789403483 | 1.658506639 | 1.885985214 | 1.798828313 |
| hsa-miR-6773-5p | 1.672197613 | 1.772759299 | 1.771900839 | 1.673934315 | 1.911862159 | 1.904832954 |
| hsa-miR-6774-3p | 1.801117487 | 1.759870452 | 1.741830491 | 1.677785685 | 1.652604534 | 1.665359691 |
| hsa-miR-6774-5p | 1.702952087 | 1.776555688 | 1.770907872 | 2.845858996 | 1.983255001 | 1.812157282 |
| hsa-miR-6775-3p | 1.869051486 | 2.762096787 | 1.885702826 | 1.931137035 | 2.035167851 | 1.759694964 |
| hsa-miR-6775-5p | 7.052893318 | 5.715457339 | 5.714320195 | 8.567699088 | 1.848193275 | 4.217955733 |
| hsa-miR-6776-3p | 1.843223038 | 1.735666564 | 1.779240784 | 1.736358662 | 1.701274838 | 1.761077648 |
| hsa-miR-6776-5p | 4.725756023 | 2.067833445 | 3.413574489 | 3.654980589 | 2.23084577 | 4.241518323 |
| hsa-miR-6777-3p | 4.454264671 | 5.475529041 | 5.747829065 | 3.50172542 | 7.234431836 | 5.713054489 |
| hsa-miR-6777-5p | 2.313036506 | 2.149250419 | 2.324282583 | 3.706527295 | 1.950628338 | 3.818854937 |
| hsa-miR-6778-3p | 1.584043866 | 2.274431949 | 1.849516422 | 1.677825049 | 2.227879854 | 1.8351454 |
| hsa-miR-6778-5p | 4.467243899 | 3.557453796 | 3.245269995 | 5.045612437 | 1.692964423 | 2.472608467 |
| hsa-miR-6779-3p | 1.663718674 | 1.652037218 | 1.948688699 | 1.626115463 | 1.784506737 | 1.707299715 |
| hsa-miR-6779-5p | 2.537037211 | 1.899544739 | 1.869631176 | 3.440713927 | 1.74246722 | 1.744941893 |
| hsa-miR-6780a-3p | 1.856857987 | 1.784644843 | 2.204964476 | 1.71575529 | 1.983949892 | 1.863591933 |
| hsa-miR-6780a-5p | 1.883157521 | 1.704461648 | 1.675271773 | 1.934423578 | 1.648362244 | 1.700201185 |
| hsa-miR-6780b-3p | 1.70666578 | 1.739451597 | 1.898245221 | 1.651619872 | 2.006922431 | 1.88406864 |
| hsa-miR-6780b-5p | 10.08498327 | 7.577019193 | 3.925712013 | 9.888101227 | 1.645537023 | 2.701354054 |
| hsa-miR-6781-3p | 1.623052361 | 1.729632462 | 1.769302004 | 1.638899217 | 1.767173998 | 1.717333166 |
| hsa-miR-6781-5p | 1.933579584 | 1.925621903 | 2.018606148 | 1.839985114 | 1.68870372 | 1.865015441 |
| hsa-miR-6782-3p | 1.880722752 | 2.384465102 | 2.881131366 | 1.919547246 | 3.350939245 | 1.860670536 |
| hsa-miR-6782-5p | 1.724524783 | 1.715365786 | 1.758035867 | 1.782432255 | 1.76423059 | 1.745665603 |
| hsa-miR-6783-3p | 1.605424508 | 1.706651855 | 1.883875105 | 1.666204997 | 1.915056344 | 1.836199187 |
| hsa-miR-6783-5p | 1.63251174 | 1.62287851 | 1.750808579 | 1.582229832 | 1.740645929 | 1.728557548 |
| hsa-miR-6784-3p | 2.326669132 | 2.02449409 | 1.994813512 | 1.8578506 | 1.966918901 | 1.863275252 |
| hsa-miR-6784-5p | 2.511714518 | 2.454727615 | 2.458297441 | 5.203508593 | 1.839502915 | 3.487059989 |
| hsa-miR-6785-3p | 3.389555304 | 4.270528222 | 4.686856056 | 3.045307849 | 5.460308636 | 4.455849117 |
| hsa-miR-6785-5p | 9.291118518 | 4.59216015 | 2.33118728 | 4.569456573 | 1.842760225 | 2.069532458 |
| hsa-miR-6786-3p | 1.729852067 | 1.867700995 | 1.816951218 | 1.727266336 | 2.311711384 | 1.881840289 |
| hsa-miR-6786-5p | 4.377940066 | 2.924920517 | 2.297398762 | 4.832935104 | 1.807102841 | 2.265844108 |
| hsa-miR-6787-3p | 1.649305095 | 1.976556434 | 1.739956519 | 1.683245541 | 1.7399637 | 1.849308475 |
| hsa-miR-6787-5p | 1.69690654 | 1.800693617 | 1.704782654 | 1.713866428 | 1.671372729 | 1.675899658 |
| hsa-miR-6788-3p | 1.791761457 | 1.701689075 | 1.746331269 | 1.661628503 | 1.768146456 | 1.836933619 |
| hsa-miR-6788-5p | 5.701466535 | 1.977112782 | 4.512165479 | 7.217225756 | 1.74721098 | 1.871382771 |
| hsa-miR-6789-3p | 1.710386953 | 1.801024455 | 1.781628987 | 1.638297902 | 2.334828619 | 1.925330868 |
| hsa-miR-6789-5p | 6.381329489 | 4.688005746 | 4.502264186 | 6.776235328 | 1.738625702 | 5.721627875 |
| hsa-miR-6790-3p | 1.975690763 | 1.85386513 | 1.972909829 | 1.889801492 | 1.904156987 | 1.937075303 |
| hsa-miR-6790-5p | 2.067735781 | 1.973659193 | 2.092327309 | 3.162440257 | 2.393958566 | 2.497653572 |
| hsa-miR-6791-3p | 1.658242672 | 1.684757741 | 1.671694759 | 1.728824477 | 1.733216091 | 1.687210697 |
| hsa-miR-6791-5p | 6.102853834 | 3.25996819 | 2.534625935 | 4.887308489 | 2.298836604 | 3.096939181 |
| hsa-miR-6792-3p | 2.114458026 | 2.745372443 | 2.894614765 | 2.099314563 | 3.476858433 | 2.61892558 |
| hsa-miR-6792-5p | 2.188050072 | 2.687355955 | 1.927822437 | 3.473073363 | 1.670185523 | 1.796034363 |
| hsa-miR-6793-3p | 1.723537701 | 1.895427635 | 1.855568254 | 1.746116662 | 1.715799805 | 1.672638718 |
| hsa-miR-6793-5p | 4.301343922 | 3.158154926 | 3.350690707 | 5.244832103 | 1.763597876 | 5.404236618 |
| hsa-miR-6794-3p | 2.246928937 | 2.490474226 | 1.777672446 | 1.949695358 | 1.865934374 | 2.060140763 |
| hsa-miR-6794-5p | 4.580591254 | 1.986136049 | 3.004550665 | 5.233962634 | 1.680596663 | 2.093690996 |
| hsa-miR-6795-3p | 4.256560496 | 5.065945428 | 5.837546902 | 2.572560719 | 7.654484525 | 4.88035582 |
| hsa-miR-6795-5p | 2.762695814 | 1.946899342 | 2.540211952 | 2.237041195 | 1.814867437 | 2.410775782 |
| hsa-miR-6796-3p | 2.879193399 | 2.281926489 | 4.23394671 | 3.020362342 | 3.28326336 | 4.163042493 |
| hsa-miR-6796-5p | 1.977254401 | 1.761403821 | 1.787853642 | 2.06198364 | 1.810670666 | 1.880751628 |
| hsa-miR-6797-3p | 6.348563312 | 8.310366243 | 8.572652738 | 3.927734636 | 11.15294559 | 8.11406759 |
| hsa-miR-6797-5p | 1.905018018 | 1.648367853 | 1.722961057 | 1.840080042 | 1.622151594 | 1.744140055 |
| hsa-miR-6798-3p | 3.185638125 | 4.068758 | 3.940249032 | 2.994039749 | 6.624652618 | 4.181693804 |
| hsa-miR-6798-5p | 2.088673892 | 1.803136969 | 2.033771739 | 3.100924828 | 1.729833229 | 2.606039349 |
| hsa-miR-6799-3p | 1.739215453 | 2.053256338 | 2.002933667 | 1.761303198 | 1.945601627 | 1.799351727 |
| hsa-miR-6799-5p | 1.886964209 | 1.801503356 | 1.71358974 | 3.613234235 | 1.704527258 | 1.79427231 |
| hsa-miR-6800-3p | 5.990394113 | 7.803627343 | 7.953771193 | 3.95806421 | 10.25782403 | 7.310359619 |
| hsa-miR-6800-5p | 8.929383319 | 10.18572317 | 8.471727567 | 6.070101447 | 2.095223391 | 5.909177655 |
| hsa-miR-6801-3p | 1.846210647 | 1.671477049 | 1.731369908 | 3.082463106 | 1.633723949 | 1.694140052 |
| hsa-miR-6801-5p | 1.816796222 | 1.697427983 | 1.731274627 | 4.383118426 | 1.678821779 | 1.718908721 |
| hsa-miR-6802-3p | 1.732803923 | 1.722293258 | 1.719319441 | 1.651387205 | 1.735660231 | 1.651423249 |
| hsa-miR-6802-5p | 1.707246426 | 2.025196978 | 1.840864501 | 1.86978987 | 1.807089374 | 1.855790725 |
| hsa-miR-6803-3p | 1.68531987 | 1.947569382 | 1.740142978 | 1.646341235 | 1.815608 | 1.890591646 |
| hsa-miR-6803-5p | 7.325065263 | 6.145777245 | 4.751191408 | 5.586303419 | 1.904975014 | 3.481732475 |
| hsa-miR-6804-3p | 1.754015742 | 1.789833942 | 1.698812699 | 1.726823873 | 1.754939305 | 1.728446856 |
| hsa-miR-6804-5p | 1.788079222 | 1.676965476 | 1.756667145 | 1.791326189 | 1.701598452 | 1.64847591 |
| hsa-miR-6805-5p | 1.961062582 | 1.804981653 | 1.778943663 | 1.740910296 | 1.670881514 | 1.697044142 |
| hsa-miR-6806-3p | 1.827781281 | 1.756721813 | 1.727219958 | 1.666152788 | 1.796380772 | 1.724040366 |
| hsa-miR-6806-5p | 1.709781862 | 1.830387847 | 1.751611907 | 2.260962436 | 2.187441538 | 1.717280641 |
| hsa-miR-6807-3p | 1.633266399 | 1.677548079 | 1.732819576 | 1.622188027 | 1.72967894 | 1.704692803 |
| hsa-miR-6807-5p | 5.430816759 | 3.70617666 | 5.774532684 | 6.325570917 | 1.849618976 | 2.299749283 |
| hsa-miR-6808-3p | 1.677457771 | 1.685707879 | 1.761571627 | 1.666693021 | 1.744551666 | 1.66688255 |
| hsa-miR-6808-5p | 2.143417014 | 1.854677552 | 1.784587067 | 2.844562432 | 2.250524771 | 1.861752019 |
| hsa-miR-6809-3p | 1.698146057 | 1.756494267 | 1.846007602 | 1.650460902 | 2.219260608 | 1.797251363 |
| hsa-miR-6809-5p | 7.73300492 | 4.820356198 | 8.437206958 | 8.820836469 | 1.600216084 | 5.047359242 |
| hsa-miR-6810-3p | 1.78420046 | 1.849114176 | 1.723742079 | 1.814617762 | 1.859062862 | 1.741090727 |
| hsa-miR-6810-5p | 1.853128966 | 1.9566717 | 1.729144829 | 1.70371926 | 1.67199148 | 1.811831197 |
| hsa-miR-6811-3p | 1.659312705 | 1.701206608 | 1.682787344 | 1.667032196 | 1.929028412 | 1.696420334 |
| hsa-miR-6811-5p | 1.672371169 | 1.670953166 | 1.625407166 | 1.650594036 | 1.702988765 | 1.663091436 |
| hsa-miR-6812-3p | 3.288137695 | 2.679703711 | 3.666853283 | 3.450744931 | 5.000574198 | 3.327277956 |
| hsa-miR-6812-5p | 6.948369434 | 7.057586502 | 8.030321638 | 7.674188907 | 4.467576235 | 7.745796955 |
| hsa-miR-6813-3p | 5.04335735 | 5.817351193 | 6.860953934 | 3.62161617 | 8.955776706 | 6.381709412 |
| hsa-miR-6813-5p | 1.794448091 | 1.715428895 | 1.745442694 | 2.549421692 | 1.818217333 | 1.785978837 |
| hsa-miR-6814-3p | 1.719352155 | 2.049336015 | 1.657296324 | 1.618829871 | 1.749200739 | 1.692241806 |
| hsa-miR-6814-5p | 1.732733536 | 2.037548086 | 1.667075928 | 1.711767424 | 1.738549575 | 1.670486898 |
| hsa-miR-6815-3p | 1.714460777 | 2.041086525 | 1.767360443 | 1.639615077 | 1.861781701 | 1.66413132 |
| hsa-miR-6815-5p | 1.955578822 | 1.750707768 | 1.767586447 | 4.544276098 | 1.70026802 | 2.264075972 |
| hsa-miR-6816-3p | 1.714481476 | 2.097035449 | 1.758764404 | 1.649914385 | 1.746828207 | 1.722417652 |
| hsa-miR-6816-5p | 1.66653966 | 1.73785094 | 1.732105426 | 1.641155175 | 1.810891508 | 1.733484384 |
| hsa-miR-6817-3p | 1.609411787 | 1.869681085 | 1.834994427 | 1.707715286 | 1.791349941 | 1.6937723 |
| hsa-miR-6817-5p | 1.659374056 | 1.74089642 | 1.797424056 | 1.728583938 | 1.900735495 | 1.782219389 |
| hsa-miR-6818-3p | 1.775682816 | 1.772653024 | 1.787810441 | 1.672465383 | 1.821739722 | 1.813293891 |
| hsa-miR-6818-5p | 3.654056249 | 2.709971607 | 5.164869371 | 3.317449774 | 1.837696463 | 5.470472468 |
| hsa-miR-6819-3p | 6.090110402 | 7.434266779 | 7.522788799 | 7.062756886 | 9.844776547 | 7.58787227 |
| hsa-miR-6819-5p | 2.061991095 | 2.438224861 | 1.775539869 | 4.867114915 | 1.813881788 | 1.81048309 |
| hsa-miR-6820-3p | 1.875085599 | 2.045363111 | 2.296395121 | 2.177055271 | 1.854418973 | 1.94959132 |
| hsa-miR-6820-5p | 1.822834689 | 1.760278956 | 2.08588158 | 3.246075793 | 2.062929337 | 2.142900178 |
| hsa-miR-6821-3p | 1.686662703 | 1.707624157 | 1.672769544 | 1.669928701 | 1.609627076 | 1.748033678 |
| hsa-miR-6821-5p | 9.196060006 | 9.278630137 | 7.895760519 | 8.793142117 | 5.076559046 | 6.644356722 |
| hsa-miR-6822-3p | 1.776176977 | 1.681416136 | 1.687060018 | 1.631887823 | 1.712508173 | 1.579606025 |
| hsa-miR-6822-5p | 1.696703272 | 1.785947055 | 1.808677644 | 1.723129156 | 2.092099106 | 1.799714094 |
| hsa-miR-6823-3p | 1.727311923 | 1.736657495 | 1.728106425 | 1.722865834 | 1.958018908 | 1.706079087 |
| hsa-miR-6823-5p | 1.846027935 | 1.637238958 | 1.811665842 | 3.318530193 | 1.745035934 | 1.709602778 |
| hsa-miR-6824-3p | 3.588968147 | 4.932216104 | 3.524717915 | 2.807917243 | 6.850605059 | 4.004135413 |
| hsa-miR-6824-5p | 5.95645021 | 2.557913264 | 2.009835906 | 4.500475002 | 2.484908393 | 3.059209248 |
| hsa-miR-6825-3p | 1.72212295 | 1.792679259 | 1.945910249 | 1.576436384 | 1.852426282 | 1.943604094 |
| hsa-miR-6825-5p | 1.734856216 | 1.70258647 | 1.981835503 | 1.720016907 | 1.720999576 | 1.786985917 |
| hsa-miR-6826-3p | 1.669338778 | 1.770475675 | 1.806615643 | 1.712156941 | 1.928989149 | 1.895138049 |
| hsa-miR-6826-5p | 3.756659716 | 2.202256643 | 1.784462692 | 2.505912847 | 1.820881874 | 2.67022224 |
| hsa-miR-6827-3p | 1.669347331 | 1.979165792 | 1.705307716 | 1.655213108 | 1.785002767 | 1.805935898 |
| hsa-miR-6827-5p | 1.691577379 | 1.876565318 | 1.717085247 | 1.913349129 | 1.739046197 | 1.780461916 |
| hsa-miR-6828-3p | 1.663526413 | 1.94024434 | 1.820390409 | 1.654234592 | 1.771658909 | 1.81406129 |
| hsa-miR-6828-5p | 1.730528428 | 1.817016731 | 1.666288106 | 1.715405896 | 1.713103921 | 1.636427483 |
| hsa-miR-6829-3p | 1.709862774 | 1.766854827 | 1.667383234 | 1.662185984 | 1.782419293 | 1.694393684 |
| hsa-miR-6829-5p | 5.942226988 | 5.887589223 | 4.811508435 | 5.775032341 | 1.844440409 | 6.947260247 |
| hsa-miR-6830-3p | 1.675592425 | 1.809712146 | 1.688413621 | 1.689012428 | 1.810551706 | 1.905152725 |
| hsa-miR-6830-5p | 1.894934993 | 1.718945486 | 2.360312102 | 1.802646574 | 1.806527965 | 1.63011004 |
| hsa-miR-6831-3p | 1.691680691 | 1.774538379 | 1.676145463 | 1.662335751 | 1.752460679 | 1.680902121 |
| hsa-miR-6831-5p | 6.619070081 | 5.761600533 | 6.208360454 | 10.2461832 | 1.715120322 | 7.533650237 |
| hsa-miR-6832-3p | 1.701695041 | 1.779799857 | 1.728202885 | 1.716469763 | 2.110296389 | 1.778588665 |
| hsa-miR-6832-5p | 1.688200785 | 1.742186828 | 1.660368141 | 5.738744638 | 1.770198876 | 1.763560885 |
| hsa-miR-6833-3p | 1.684464722 | 1.891981334 | 1.708048187 | 1.688673931 | 1.781466595 | 1.682240098 |
| hsa-miR-6833-5p | 8.514420085 | 4.748644141 | 7.781538207 | 8.230276859 | 1.673733247 | 5.243094703 |
| hsa-miR-6834-3p | 3.039610952 | 4.547944165 | 4.654897476 | 2.703070536 | 4.003752318 | 4.004354892 |
| hsa-miR-6834-5p | 1.728611682 | 1.695447333 | 1.810801127 | 1.793533439 | 1.859398896 | 1.765895531 |
| hsa-miR-6835-3p | 1.738850981 | 2.014421929 | 1.875368111 | 1.676269517 | 1.850952074 | 1.980637074 |
| hsa-miR-6835-5p | 1.714318698 | 1.925631256 | 1.778669267 | 1.777329392 | 1.788670622 | 1.705589199 |
| hsa-miR-6836-3p | 1.777919367 | 2.229308496 | 1.766798399 | 2.378268577 | 2.022130631 | 1.874917132 |
| hsa-miR-6836-5p | 1.604224197 | 1.703172538 | 1.751009485 | 1.582126546 | 1.668983248 | 1.721415308 |
| hsa-miR-6837-3p | 1.725722575 | 1.87603305 | 1.753458753 | 1.663818478 | 1.716898338 | 1.738939886 |
| hsa-miR-6837-5p | 1.898349229 | 1.710449678 | 1.749594332 | 4.523763194 | 1.708298822 | 1.704164201 |
| hsa-miR-6838-3p | 1.650216332 | 1.803162161 | 1.745130521 | 1.657025624 | 1.669696573 | 1.762483233 |
| hsa-miR-6838-5p | 1.680509292 | 1.706322476 | 1.59219667 | 1.747792007 | 1.713542158 | 1.68178577 |
| hsa-miR-6839-3p | 1.751262746 | 1.745064358 | 2.088106075 | 1.707913963 | 2.326766257 | 1.819281013 |
| hsa-miR-6839-5p | 1.681830327 | 1.733308155 | 1.76540386 | 1.659843988 | 1.701688545 | 1.725251834 |
| hsa-miR-6840-3p | 3.561607481 | 2.176796355 | 3.051603565 | 3.187014086 | 1.739622888 | 2.462807689 |
| hsa-miR-6840-5p | 1.694574346 | 1.794628622 | 1.745044068 | 1.705364064 | 1.745188763 | 1.682516933 |
| hsa-miR-6841-3p | 1.69360306 | 1.892284054 | 1.830488682 | 1.706501851 | 1.826702494 | 2.215700791 |
| hsa-miR-6841-5p | 1.777295305 | 1.946314028 | 1.723028097 | 1.621549064 | 1.851748725 | 1.688505727 |
| hsa-miR-6842-3p | 1.644298615 | 1.692739502 | 1.693440583 | 1.582470515 | 1.687449335 | 1.649228778 |
| hsa-miR-6842-5p | 1.64759923 | 1.709198371 | 1.70060752 | 1.673708157 | 1.849860366 | 1.760513496 |
| hsa-miR-6843-3p | 1.755259593 | 1.74850403 | 1.974663924 | 1.670186183 | 1.816384582 | 1.8122881 |
| hsa-miR-6844 | 1.791028364 | 1.863918263 | 2.570494585 | 1.733796255 | 3.608180168 | 2.105286994 |
| hsa-miR-6845-3p | 1.744301262 | 1.735661829 | 1.772146445 | 1.716363181 | 1.775072111 | 1.682453488 |
| hsa-miR-6845-5p | 4.521753277 | 2.565567542 | 2.106811131 | 7.106882615 | 1.87265805 | 2.373332915 |
| hsa-miR-6846-3p | 1.750490195 | 1.664123003 | 1.722047083 | 1.670780023 | 1.97209112 | 1.790960591 |
| hsa-miR-6846-5p | 2.815728952 | 1.916882151 | 1.877448678 | 4.945074188 | 1.819458585 | 1.785087766 |
| hsa-miR-6847-3p | 1.7012403 | 1.909425855 | 1.877858047 | 1.806318812 | 1.885075221 | 1.699749461 |
| hsa-miR-6847-5p | 2.553632803 | 1.956156231 | 1.691494025 | 2.031695636 | 1.613453159 | 1.66328193 |
| hsa-miR-6848-3p | 3.592604336 | 4.606322122 | 4.582633159 | 3.41564896 | 7.94529732 | 5.344568372 |
| hsa-miR-6848-5p | 1.617859312 | 1.816987067 | 1.809175857 | 1.743892268 | 1.909577848 | 1.814488082 |
| hsa-miR-6849-3p | 1.683200179 | 1.745210353 | 1.822719185 | 1.682001768 | 1.6954802 | 1.727113549 |
| hsa-miR-6849-5p | 3.397042464 | 1.867417764 | 2.222601535 | 3.752623719 | 1.834089399 | 2.051219657 |
| hsa-miR-6850-3p | 1.865639045 | 1.705210181 | 1.739387412 | 1.673456517 | 1.828861347 | 1.82915984 |
| hsa-miR-6850-5p | 4.649166243 | 5.07236596 | 1.821205152 | 6.481130587 | 1.822038768 | 1.834138689 |
| hsa-miR-6851-3p | 5.274482178 | 6.701339916 | 7.293862724 | 3.364862831 | 10.00885728 | 6.833864113 |
| hsa-miR-6851-5p | 1.728657316 | 1.782724848 | 1.70328922 | 1.875693332 | 1.744080676 | 1.738486276 |
| hsa-miR-6852-3p | 1.705018461 | 2.071779252 | 1.784024935 | 1.71274792 | 1.890080075 | 1.748424384 |
| hsa-miR-6852-5p | 1.70806733 | 1.684371161 | 1.648575709 | 1.626258325 | 1.629473129 | 1.650138022 |
| hsa-miR-6853-3p | 1.688068327 | 1.759851604 | 1.684896675 | 1.658945924 | 1.797056885 | 1.669693063 |
| hsa-miR-6853-5p | 1.905455489 | 1.743501645 | 1.723882732 | 1.8323727 | 1.858298558 | 1.805364531 |
| hsa-miR-6854-3p | 1.701685228 | 1.906175279 | 1.795174201 | 1.730543941 | 1.901062658 | 1.837296062 |
| hsa-miR-6854-5p | 1.700021707 | 1.662324091 | 1.858159106 | 1.631154708 | 1.77772693 | 1.758224314 |
| hsa-miR-6855-3p | 2.173601686 | 2.296534412 | 1.976071466 | 2.051630185 | 1.918556157 | 2.036471383 |
| hsa-miR-6855-5p | 1.727279432 | 1.676909838 | 1.842800512 | 1.701372968 | 1.799984094 | 1.698390698 |
| hsa-miR-6856-3p | 2.128895633 | 1.810083299 | 1.718107126 | 1.669604837 | 2.164634179 | 1.695090455 |
| hsa-miR-6856-5p | 1.921962251 | 1.936788634 | 1.865142263 | 7.211004848 | 1.807843928 | 1.851309162 |
| hsa-miR-6857-3p | 1.6999874 | 2.050455541 | 2.036869875 | 1.821758809 | 1.745368022 | 1.820197568 |
| hsa-miR-6857-5p | 1.941702168 | 1.75838247 | 1.695694811 | 3.419438728 | 1.665995743 | 1.737922496 |
| hsa-miR-6858-3p | 2.681349601 | 3.299287573 | 4.406442404 | 2.528304115 | 4.403912119 | 3.398329079 |
| hsa-miR-6858-5p | 5.342853136 | 4.205007725 | 2.640061835 | 4.803767792 | 1.774456716 | 2.159620706 |
| hsa-miR-6859-3p | 1.790658165 | 1.79585333 | 1.73015352 | 1.636576994 | 1.793617734 | 1.736425222 |
| hsa-miR-6859-5p | 2.199988372 | 1.92472382 | 2.240967124 | 3.100347688 | 1.866514362 | 1.896050302 |
| hsa-miR-6860 | 2.051883849 | 1.869641413 | 2.300330156 | 3.800483113 | 1.752780314 | 1.892715583 |
| hsa-miR-6861-3p | 4.130430469 | 5.742509523 | 5.518245125 | 2.95894696 | 7.6783062 | 5.248823166 |
| hsa-miR-6861-5p | 3.526808751 | 1.830438195 | 1.65267118 | 3.816950358 | 1.614228512 | 1.709148717 |
| hsa-miR-6862-3p | 1.738582829 | 1.832709281 | 1.696346063 | 1.672027379 | 1.695753647 | 1.647924409 |
| hsa-miR-6862-5p | 1.747885463 | 1.924022308 | 1.863378928 | 5.061823331 | 1.837337432 | 1.753006399 |
| hsa-miR-6863 | 1.823474548 | 1.752541551 | 1.618946408 | 1.789879655 | 1.783248384 | 1.568114146 |
| hsa-miR-6864-3p | 1.769465158 | 1.700385428 | 1.76155933 | 1.641188487 | 1.848627837 | 1.700248865 |
| hsa-miR-6864-5p | 1.733381164 | 1.718948237 | 1.744246329 | 1.785899579 | 1.71210977 | 1.715934393 |
| hsa-miR-6865-3p | 3.058462012 | 5.212817397 | 4.129099821 | 2.75051727 | 5.878143313 | 4.422573344 |
| hsa-miR-6865-5p | 4.50410074 | 2.604458696 | 5.189292551 | 7.272171042 | 2.138584826 | 2.989710186 |
| hsa-miR-6866-3p | 1.752543425 | 1.737494887 | 1.986121919 | 1.691825304 | 2.5239492 | 1.937903578 |
| hsa-miR-6866-5p | 1.772153641 | 1.714073032 | 1.771793355 | 1.633119859 | 1.790841378 | 1.657144679 |
| hsa-miR-6867-3p | 1.704817999 | 1.793129983 | 2.088665721 | 1.679619619 | 2.074136129 | 1.759292504 |
| hsa-miR-6867-5p | 9.160433078 | 9.126097097 | 10.26133217 | 10.33199978 | 1.83980317 | 8.417651877 |
| hsa-miR-6868-3p | 1.759966862 | 1.6978493 | 1.884602646 | 1.665979674 | 1.769490462 | 1.811765269 |
| hsa-miR-6868-5p | 1.70642384 | 1.728646238 | 1.682282354 | 1.727672244 | 1.817797996 | 1.788979967 |
| hsa-miR-6869-3p | 1.74131085 | 1.752502476 | 1.838052776 | 1.683431985 | 1.761780767 | 1.79557506 |
| hsa-miR-6869-5p | 9.178995942 | 9.540303518 | 6.737838047 | 6.10072932 | 2.156490985 | 5.686146231 |
| hsa-miR-6870-3p | 3.417666273 | 3.660690718 | 4.571246487 | 2.597671529 | 6.221627493 | 4.266804305 |
| hsa-miR-6870-5p | 2.07339457 | 1.79297496 | 1.773348009 | 2.872578983 | 1.787613394 | 3.206873163 |
| hsa-miR-6871-3p | 1.600644426 | 1.649949419 | 1.70435876 | 1.68755695 | 1.736916083 | 1.718318894 |
| hsa-miR-6871-5p | 2.684593945 | 1.802406608 | 2.052326125 | 2.172029854 | 1.726026977 | 1.684014415 |
| hsa-miR-6872-3p | 3.910660847 | 1.72412867 | 1.704244508 | 2.126584893 | 1.890974059 | 1.905832831 |
| hsa-miR-6872-5p | 2.531545138 | 1.840989178 | 2.028960518 | 2.760063573 | 1.695340107 | 1.7983979 |
| hsa-miR-6873-3p | 1.800512699 | 1.785214611 | 1.780443845 | 1.7915071 | 1.750602255 | 1.859509848 |
| hsa-miR-6873-5p | 2.776444321 | 1.703440264 | 2.043145013 | 3.502881292 | 2.345208504 | 1.782653339 |
| hsa-miR-6874-3p | 1.683519894 | 1.782642158 | 1.815415523 | 1.64917423 | 1.853950877 | 1.783734497 |
| hsa-miR-6874-5p | 1.657957961 | 1.808289987 | 1.670733828 | 1.678324666 | 1.652476766 | 1.715510218 |
| hsa-miR-6875-3p | 1.881076492 | 1.778679859 | 1.619090741 | 1.775751211 | 1.703238495 | 1.73765419 |
| hsa-miR-6875-5p | 4.458221062 | 1.883151303 | 1.740382437 | 3.566814646 | 1.702255181 | 1.854591999 |
| hsa-miR-6876-3p | 1.662923337 | 2.686257603 | 1.926303269 | 1.609736492 | 1.876804276 | 1.786504934 |
| hsa-miR-6876-5p | 1.74227748 | 1.867722247 | 1.772248389 | 1.824586989 | 1.778762775 | 1.722817862 |
| hsa-miR-6877-3p | 1.997213058 | 1.765118679 | 1.747828335 | 1.710654472 | 1.744436425 | 1.656285365 |
| hsa-miR-6877-5p | 2.311451459 | 1.846033488 | 1.963998162 | 2.006425881 | 1.788628259 | 2.008506614 |
| hsa-miR-6878-3p | 1.723619822 | 1.764098859 | 1.933879977 | 1.699044011 | 1.880145432 | 1.780830624 |
| hsa-miR-6878-5p | 1.750663265 | 1.780038742 | 1.758820453 | 1.757181584 | 1.944094201 | 1.698919394 |
| hsa-miR-6879-3p | 1.753612153 | 1.827446906 | 1.85926113 | 1.710391217 | 1.751450177 | 1.775676782 |
| hsa-miR-6879-5p | 9.278618669 | 9.750733284 | 10.2527779 | 9.844996369 | 6.718490327 | 10.07963331 |
| hsa-miR-6880-3p | 4.846643694 | 6.409985374 | 6.622295634 | 3.183686104 | 9.608300058 | 6.169659123 |
| hsa-miR-6880-5p | 3.765120413 | 3.891050252 | 4.299055336 | 3.645729219 | 1.825876535 | 3.873597571 |
| hsa-miR-6881-3p | 1.874266782 | 2.01362848 | 1.788517453 | 1.845364237 | 1.777339408 | 1.672369661 |
| hsa-miR-6881-5p | 2.561436827 | 1.824184027 | 1.901790025 | 4.991429372 | 1.811817024 | 1.834810826 |
| hsa-miR-6882-3p | 1.660287762 | 1.710437723 | 1.815644308 | 1.597474984 | 1.882625436 | 1.791516514 |
| hsa-miR-6882-5p | 1.792186245 | 1.716513699 | 1.702609776 | 1.826968277 | 1.646368231 | 1.748685733 |
| hsa-miR-6883-3p | 1.761674019 | 2.088939814 | 1.752318655 | 1.807657337 | 2.054633497 | 1.825798423 |
| hsa-miR-6883-5p | 1.861417284 | 1.95202383 | 2.181271161 | 2.729658689 | 2.176508465 | 1.817386175 |
| hsa-miR-6884-3p | 1.775077854 | 2.255919457 | 1.738481708 | 1.831255552 | 1.896719414 | 1.855945039 |
| hsa-miR-6884-5p | 1.708842594 | 1.779699908 | 1.686357991 | 1.729201068 | 1.749579021 | 1.697568754 |
| hsa-miR-6885-3p | 1.708872004 | 2.193700409 | 1.984574948 | 1.847246677 | 2.335908892 | 2.654887264 |
| hsa-miR-6885-5p | 2.602199927 | 1.864356677 | 1.802140724 | 1.859214667 | 1.776361136 | 2.003410966 |
| hsa-miR-6886-3p | 1.79828194 | 2.577137382 | 1.995184861 | 1.737069853 | 1.773806604 | 2.001035899 |
| hsa-miR-6886-5p | 1.78822559 | 1.892776075 | 1.790306627 | 2.457476159 | 1.9118686 | 1.830342323 |
| hsa-miR-6887-3p | 1.707135405 | 1.835806949 | 1.796199897 | 1.683363782 | 1.794645573 | 1.819755973 |
| hsa-miR-6887-5p | 2.866652383 | 1.830195417 | 1.761157466 | 5.384969273 | 1.641265267 | 1.841659638 |
| hsa-miR-6888-3p | 1.67320641 | 1.913532569 | 1.840632229 | 1.711082492 | 2.200344015 | 1.719185028 |
| hsa-miR-6888-5p | 1.676190889 | 1.742481653 | 1.809465692 | 1.626975999 | 1.816217095 | 1.850078778 |
| hsa-miR-6889-3p | 4.798530392 | 6.038601785 | 7.176746556 | 3.501963969 | 10.36612984 | 6.662362919 |
| hsa-miR-6889-5p | 1.697590132 | 1.824959745 | 1.673966173 | 1.794147407 | 1.788752098 | 1.707325457 |
| hsa-miR-6890-3p | 1.874224716 | 1.819953958 | 2.062416844 | 1.810193941 | 2.223621581 | 1.824303342 |
| hsa-miR-6890-5p | 2.091313663 | 1.76349185 | 1.731526823 | 3.330597075 | 1.78018911 | 1.901591146 |
| hsa-miR-6891-3p | 2.773475447 | 2.886069734 | 3.363344884 | 2.035081745 | 4.17649991 | 3.178781737 |
| hsa-miR-6891-5p | 6.940060313 | 6.068683607 | 5.342928151 | 7.204648686 | 1.922927935 | 6.302894185 |
| hsa-miR-6892-3p | 2.200717661 | 2.033574524 | 2.121075328 | 3.236994561 | 2.576351005 | 1.908498223 |
| hsa-miR-6892-5p | 4.184582525 | 3.848327799 | 3.470870986 | 3.124698261 | 1.698864038 | 3.973223153 |
| hsa-miR-6893-3p | 1.685219661 | 1.831722442 | 1.716515989 | 1.656417902 | 1.701546376 | 1.75730292 |
| hsa-miR-6893-5p | 10.16820542 | 3.833159396 | 7.471723551 | 5.850228615 | 1.937363425 | 3.478375717 |
| hsa-miR-6894-3p | 1.672040391 | 2.013470109 | 1.740106625 | 1.591518276 | 1.844353043 | 1.978705024 |
| hsa-miR-6894-5p | 1.749152891 | 1.924907419 | 1.983793708 | 2.928327391 | 1.828156798 | 1.883360182 |
| hsa-miR-6895-3p | 1.641026318 | 1.717730818 | 1.829062315 | 1.660437035 | 1.967542205 | 1.932757526 |
| hsa-miR-6895-5p | 1.693853968 | 1.844889259 | 1.687776817 | 2.106775602 | 1.711923749 | 1.708144027 |
| hsa-miR-7-1-3p | 1.724383406 | 1.981292968 | 1.825191372 | 2.79323332 | 1.936358829 | 1.8054052 |
| hsa-miR-7-2-3p | 1.692800172 | 1.906693875 | 1.907222344 | 1.66916762 | 1.854678916 | 1.834010497 |
| hsa-miR-7-5p | 3.634864204 | 2.160792248 | 2.264482953 | 3.142712697 | 2.635091073 | 1.987448638 |
| hsa-miR-708-3p | 1.728451064 | 1.687302517 | 1.614342668 | 1.644230772 | 2.032617731 | 1.723666835 |
| hsa-miR-708-5p | 1.976242631 | 1.781985242 | 1.977889602 | 4.119368434 | 1.847910829 | 2.531955115 |
| hsa-miR-7106-3p | 1.738849654 | 1.817204402 | 1.733014845 | 1.755677628 | 2.00651373 | 1.744614097 |
| hsa-miR-7106-5p | 3.774215099 | 3.613677958 | 3.867385327 | 4.557587822 | 1.625688087 | 4.802884556 |
| hsa-miR-7107-3p | 1.674710402 | 2.06806601 | 1.730286477 | 1.803952941 | 2.159591023 | 1.829161525 |
| hsa-miR-7107-5p | 10.31027542 | 10.15854024 | 10.88467154 | 11.68975353 | 3.87628419 | 10.09252485 |
| hsa-miR-7108-3p | 2.015833207 | 1.811179336 | 2.13604644 | 2.044556308 | 2.056882444 | 2.001581956 |
| hsa-miR-7108-5p | 5.809815117 | 4.833047175 | 2.97039729 | 5.343772405 | 1.858494775 | 2.526865255 |
| hsa-miR-7109-3p | 1.64456085 | 1.816205013 | 1.803848168 | 1.68314338 | 1.752302946 | 1.736478139 |
| hsa-miR-7109-5p | 1.894612581 | 1.835782702 | 1.783709721 | 4.379334727 | 1.825815463 | 2.313993935 |
| hsa-miR-711 | 4.240119938 | 2.083671 | 2.104581449 | 8.088300741 | 1.863183734 | 2.557474296 |
| hsa-miR-7110-3p | 1.704071362 | 1.876691641 | 1.765253482 | 1.681093913 | 1.892693945 | 1.905771812 |
| hsa-miR-7110-5p | 11.88876559 | 10.78845624 | 11.46752216 | 12.34675635 | 6.931675672 | 10.79124535 |
| hsa-miR-7111-3p | 4.056201604 | 4.098629014 | 3.574453872 | 2.734994518 | 5.412553325 | 3.845455863 |
| hsa-miR-7111-5p | 1.7196756 | 1.751861861 | 1.711800305 | 3.410610029 | 1.700600427 | 1.728463132 |
| hsa-miR-7112-3p | 1.681390919 | 1.689676376 | 1.737686201 | 1.57605711 | 1.64294757 | 1.687802565 |
| hsa-miR-7112-5p | 1.666975787 | 1.685339677 | 1.700260569 | 1.651180641 | 1.768176466 | 1.73752463 |
| hsa-miR-7113-3p | 1.696211119 | 1.673657521 | 1.924056611 | 1.708447755 | 2.019708605 | 1.751236535 |
| hsa-miR-7113-5p | 1.78453707 | 1.817346217 | 1.866862238 | 1.945331214 | 1.748157689 | 1.845451139 |
| hsa-miR-7114-3p | 3.967889723 | 4.749105751 | 4.442999602 | 2.682941296 | 6.238524476 | 4.994884944 |
| hsa-miR-7114-5p | 2.876856358 | 1.785985852 | 1.765401329 | 3.627718204 | 1.758247951 | 1.829592463 |
| hsa-miR-7150 | 7.826256153 | 6.332017873 | 5.684936081 | 8.884223678 | 1.800099457 | 3.472312184 |
| hsa-miR-7151-3p | 1.673904203 | 1.750266981 | 1.72743689 | 2.391284786 | 1.752399148 | 1.80261514 |
| hsa-miR-7151-5p | 1.846100779 | 1.780408917 | 1.740751409 | 1.637436484 | 1.65045884 | 1.683474109 |
| hsa-miR-7152-3p | 7.39231848 | 1.821615457 | 2.094677134 | 4.111643834 | 1.772647548 | 2.751826584 |
| hsa-miR-7152-5p | 1.83079383 | 1.703548519 | 1.92412742 | 1.930807576 | 1.907378307 | 2.021621191 |
| hsa-miR-7153-3p | 1.641849261 | 1.764828258 | 1.710401177 | 1.60756085 | 1.840457856 | 1.756262752 |
| hsa-miR-7153-5p | 1.659835291 | 2.248117061 | 2.102732002 | 1.77098002 | 1.824830301 | 2.008393428 |
| hsa-miR-7154-3p | 1.747541254 | 1.886050647 | 1.783632579 | 1.700153197 | 1.926032327 | 2.007983382 |
| hsa-miR-7154-5p | 1.669579825 | 1.905777622 | 1.654900347 | 1.714343807 | 1.789387319 | 1.7842603 |
| hsa-miR-7155-3p | 3.310459277 | 2.16027916 | 2.865080937 | 2.49095755 | 1.739956641 | 3.35575045 |
| hsa-miR-7155-5p | 1.648433086 | 2.078184887 | 1.95403954 | 2.322029073 | 1.78707693 | 1.784490813 |
| hsa-miR-7156-3p | 1.762302347 | 1.825467315 | 1.866840431 | 2.068205241 | 2.119003468 | 1.775479184 |
| hsa-miR-7156-5p | 1.762847085 | 1.713965828 | 1.837460802 | 1.680277427 | 1.81713206 | 1.681158406 |
| hsa-miR-7157-3p | 1.7182654 | 1.677635704 | 1.726864935 | 1.812030866 | 1.758090458 | 1.750690746 |
| hsa-miR-7157-5p | 1.96249957 | 1.864261776 | 1.724076191 | 1.9083458 | 1.673848462 | 1.670999197 |
| hsa-miR-7158-3p | 1.632589257 | 1.718582173 | 1.714251709 | 1.697627257 | 1.662425513 | 1.657947038 |
| hsa-miR-7158-5p | 1.65583783 | 1.761845275 | 1.708437851 | 1.617196048 | 1.912088363 | 1.646078163 |
| hsa-miR-7159-3p | 1.85287379 | 1.823257114 | 1.990061018 | 2.302332146 | 1.935046041 | 1.806981664 |
| hsa-miR-7159-5p | 1.791833068 | 1.815350802 | 1.801578372 | 4.967464738 | 1.631140525 | 1.693481809 |
| hsa-miR-7160-3p | 1.672123285 | 1.757658702 | 1.71525082 | 1.648304113 | 1.712103067 | 1.637418614 |
| hsa-miR-7160-5p | 1.646309834 | 1.895084847 | 1.913799717 | 1.650449644 | 1.719109991 | 1.767823128 |
| hsa-miR-7161-3p | 1.683460785 | 1.865147595 | 1.690448635 | 1.770437568 | 1.778011637 | 1.833867055 |
| hsa-miR-7161-5p | 1.693414363 | 1.657334782 | 1.815733776 | 3.388651324 | 1.770275948 | 1.72837769 |
| hsa-miR-7162-3p | 1.738314166 | 1.691393381 | 1.854246362 | 1.7461716 | 1.755146032 | 1.775612202 |
| hsa-miR-7162-5p | 1.707463317 | 1.747073251 | 1.824150749 | 1.669367249 | 1.833666799 | 1.845750325 |
| hsa-miR-718 | 3.41795342 | 2.324220522 | 1.806055255 | 3.68931951 | 1.767969289 | 1.65311263 |
| hsa-miR-744-3p | 1.607420286 | 1.804417171 | 1.760806513 | 1.652775477 | 1.827908045 | 1.775248402 |
| hsa-miR-744-5p | 2.052089262 | 1.765777284 | 1.685274751 | 1.776985674 | 1.690438359 | 1.600814946 |
| hsa-miR-7515 | 1.782206237 | 1.983349509 | 1.698680138 | 1.965277215 | 1.745052782 | 1.717863369 |
| hsa-miR-758-3p | 1.70923838 | 1.695855577 | 1.832910661 | 1.66102088 | 1.766266055 | 1.740261829 |
| hsa-miR-758-5p | 1.771602906 | 1.888408805 | 1.790978187 | 1.826578615 | 1.731248545 | 1.813911405 |
| hsa-miR-759 | 1.711833865 | 1.71491598 | 1.72633889 | 1.696694325 | 1.856971508 | 1.788454957 |
| hsa-miR-760 | 6.033011721 | 2.273689941 | 1.791194951 | 3.719394018 | 1.645029331 | 5.748409419 |
| hsa-miR-761 | 1.662832743 | 1.782728904 | 1.851349657 | 1.516559169 | 1.748477068 | 1.765030894 |
| hsa-miR-762 | 7.153609197 | 7.733163131 | 4.524398607 | 8.754530371 | 3.616901241 | 4.126662562 |
| hsa-miR-764 | 1.70934844 | 1.780590807 | 2.006862675 | 1.661856513 | 1.923811002 | 1.911344921 |
| hsa-miR-7641 | 11.45426198 | 8.037611409 | 6.714387039 | 7.605668766 | 2.253264542 | 5.977945017 |
| hsa-miR-765 | 8.072972579 | 7.702888206 | 8.573638965 | 10.35556929 | 2.285966936 | 6.112413931 |
| hsa-miR-766-3p | 4.110332257 | 4.622092733 | 5.164836876 | 2.969099305 | 2.430720813 | 3.518325623 |
| hsa-miR-766-5p | 1.693119189 | 1.730904239 | 1.855091404 | 1.788133607 | 2.044300999 | 1.798028629 |
| hsa-miR-767-3p | 1.74168037 | 1.841540851 | 2.442716607 | 1.828572509 | 3.380606107 | 2.243609292 |
| hsa-miR-767-5p | 1.701748871 | 1.740198958 | 1.604545883 | 1.795536535 | 1.789469078 | 1.713347423 |
| hsa-miR-769-3p | 1.667736298 | 1.680712079 | 1.706866916 | 1.82260624 | 1.670999548 | 1.691953597 |
| hsa-miR-769-5p | 2.296591066 | 1.805737594 | 1.818393856 | 1.635739194 | 1.957531015 | 1.792103203 |
| hsa-miR-770-5p | 1.713283166 | 1.837625773 | 1.754799941 | 1.803528778 | 1.907909826 | 1.829427333 |
| hsa-miR-7702 | 1.671664962 | 1.720942248 | 1.815221108 | 1.737159787 | 1.692936537 | 1.652184099 |
| hsa-miR-7703 | 1.662167129 | 1.824263632 | 1.622752552 | 1.802279357 | 1.658384654 | 1.700078751 |
| hsa-miR-7704 | 8.119733623 | 5.761363981 | 3.571967031 | 3.347620135 | 1.71453736 | 3.110345304 |
| hsa-miR-7705 | 1.695016259 | 1.814157151 | 1.739340761 | 1.643983229 | 1.792739286 | 1.714416405 |
| hsa-miR-7706 | 1.653711939 | 1.749903496 | 1.708082893 | 1.680951441 | 1.686139607 | 1.746090201 |
| hsa-miR-7843-3p | 1.611747194 | 1.67664442 | 1.714493874 | 1.613611988 | 2.018929075 | 1.691768071 |
| hsa-miR-7843-5p | 1.669310785 | 1.6763221 | 1.775136047 | 1.585982262 | 1.833837306 | 1.809106254 |
| hsa-miR-7844-5p | 1.670817625 | 1.762438233 | 1.70771893 | 1.883472375 | 1.780073086 | 1.777628363 |
| hsa-miR-7845-5p | 4.971743347 | 3.522524685 | 3.154509515 | 4.876974743 | 1.612246756 | 3.268321254 |
| hsa-miR-7846-3p | 3.866307452 | 2.001716568 | 2.008405647 | 5.777103384 | 1.707508334 | 2.20435359 |
| hsa-miR-7847-3p | 7.591892044 | 7.145582685 | 7.701765885 | 11.12252041 | 1.752325952 | 8.896602414 |
| hsa-miR-7848-3p | 1.678807779 | 2.033484753 | 1.66946273 | 1.739261396 | 1.720754622 | 1.712267834 |
| hsa-miR-7849-3p | 1.641852992 | 1.960163325 | 1.932364685 | 1.738989482 | 1.824230959 | 1.956504955 |
| hsa-miR-7850-5p | 1.771375367 | 1.816124223 | 1.981094254 | 1.752271269 | 2.399672487 | 1.877805327 |
| hsa-miR-7851-3p | 2.373483465 | 1.770524923 | 1.698422936 | 1.793000985 | 1.724878314 | 1.923127903 |
| hsa-miR-7852-3p | 1.737153583 | 1.766105593 | 1.725572479 | 1.748360888 | 1.837551457 | 1.775595801 |
| hsa-miR-7853-5p | 1.667565348 | 1.804918507 | 1.77496051 | 1.689107518 | 2.167667476 | 1.686865737 |
| hsa-miR-7854-3p | 2.317905605 | 1.648858954 | 1.666397814 | 1.758309255 | 1.78164904 | 1.804810883 |
| hsa-miR-7855-5p | 1.671051342 | 1.826622201 | 1.965296945 | 1.78521084 | 1.967398471 | 1.810615469 |
| hsa-miR-7856-5p | 1.668315265 | 1.72675649 | 1.705387891 | 1.739949859 | 1.755286693 | 1.742564489 |
| hsa-miR-7973 | 1.674439362 | 2.102363016 | 1.88101177 | 1.626754517 | 2.159731723 | 2.162936087 |
| hsa-miR-7974 | 2.450284688 | 2.044885026 | 1.927474293 | 1.919097936 | 2.306354128 | 2.099534903 |
| hsa-miR-7975 | 10.38829959 | 11.63823624 | 9.883028402 | 10.07308208 | 12.46493894 | 12.11264589 |
| hsa-miR-7976 | 1.694527891 | 1.781509187 | 1.765970429 | 1.744765681 | 1.83376689 | 1.80299205 |
| hsa-miR-7977 | 10.08935937 | 11.83752124 | 9.505263748 | 9.813273668 | 12.25120075 | 11.52891285 |
| hsa-miR-7978 | 1.767232486 | 1.898688778 | 1.79185853 | 1.678619692 | 1.760756558 | 1.725058056 |
| hsa-miR-802 | 1.808688083 | 1.702551859 | 1.941549112 | 1.719431806 | 1.942367363 | 1.756900767 |
| hsa-miR-8052 | 1.708822958 | 1.744211176 | 1.778325237 | 1.981806634 | 1.716371438 | 1.696949198 |
| hsa-miR-8053 | 1.645408441 | 1.741084725 | 1.778394268 | 1.573938359 | 1.911008807 | 1.913266651 |
| hsa-miR-8054 | 1.755583261 | 1.839245978 | 1.771814495 | 1.784725037 | 1.790078095 | 1.800357041 |
| hsa-miR-8055 | 1.771738047 | 1.691424774 | 1.692892005 | 1.775040183 | 1.778343979 | 1.776730322 |
| hsa-miR-8056 | 1.76140002 | 1.71219553 | 1.786052037 | 1.747070573 | 1.995731257 | 1.705116741 |
| hsa-miR-8057 | 1.695870956 | 1.753414973 | 1.705679814 | 1.590344581 | 1.953487111 | 1.88613243 |
| hsa-miR-8058 | 1.835805781 | 1.727797453 | 1.746648851 | 1.754277561 | 2.216466272 | 2.059118263 |
| hsa-miR-8059 | 1.876541988 | 1.704031086 | 1.806248018 | 1.686562802 | 1.666291665 | 1.690902637 |
| hsa-miR-8060 | 5.70336968 | 4.684470197 | 6.439549968 | 5.431750117 | 2.514215379 | 6.241845238 |
| hsa-miR-8061 | 1.676359335 | 1.797557678 | 1.806226339 | 1.790171793 | 1.89589452 | 1.856637261 |
| hsa-miR-8062 | 1.693155956 | 1.896967055 | 1.935608685 | 1.712215921 | 2.009462486 | 1.902340483 |
| hsa-miR-8063 | 4.430014366 | 2.660487523 | 2.282229325 | 6.315267043 | 2.333093735 | 1.784392581 |
| hsa-miR-8064 | 2.738264696 | 2.766166399 | 2.983377852 | 4.089916881 | 1.821452373 | 2.879748956 |
| hsa-miR-8065 | 1.745917972 | 1.809841872 | 1.795303258 | 1.597829256 | 2.732363596 | 1.804781354 |
| hsa-miR-8066 | 1.987466636 | 1.828142045 | 1.834700624 | 1.818244389 | 2.204183645 | 1.801999618 |
| hsa-miR-8067 | 1.707615618 | 1.805696461 | 2.292929603 | 1.699782719 | 2.902607382 | 2.054365866 |
| hsa-miR-8068 | 1.68760597 | 1.71800646 | 1.660529917 | 1.777487213 | 2.493106538 | 1.82187051 |
| hsa-miR-8069 | 11.4345625 | 9.892214861 | 5.617197465 | 8.39639396 | 2.478284076 | 6.74689475 |
| hsa-miR-8070 | 1.966722372 | 1.737268494 | 1.729752558 | 1.652402345 | 1.69890037 | 1.710918241 |
| hsa-miR-8071 | 4.079228175 | 4.179706029 | 2.652207001 | 4.376810524 | 1.652656632 | 2.693055095 |
| hsa-miR-8072 | 5.352112372 | 4.551202802 | 4.655559151 | 6.031273383 | 1.991669135 | 3.661774047 |
| hsa-miR-8073 | 2.160977935 | 2.104221162 | 2.222520222 | 2.061355039 | 1.837796935 | 2.370256429 |
| hsa-miR-8074 | 1.700339214 | 1.814632356 | 2.125582172 | 1.69540755 | 1.766604069 | 1.82044425 |
| hsa-miR-8075 | 1.794488451 | 1.94465236 | 1.964801482 | 1.824653201 | 1.991221047 | 1.890595842 |
| hsa-miR-8076 | 1.674633351 | 2.003948511 | 1.852151407 | 1.59250642 | 2.005111256 | 1.919004337 |
| hsa-miR-8077 | 1.750337342 | 1.760761749 | 1.724470525 | 1.66924713 | 1.738417417 | 1.685555946 |
| hsa-miR-8078 | 1.771799258 | 1.690128091 | 1.681375918 | 1.773418803 | 1.702213895 | 1.74672342 |
| hsa-miR-8079 | 1.659326262 | 1.838713769 | 1.986428454 | 1.685365232 | 1.812173012 | 1.922792851 |
| hsa-miR-8080 | 1.765360747 | 1.967112995 | 1.844214284 | 1.661189738 | 1.748623724 | 1.702355062 |
| hsa-miR-8081 | 1.748195901 | 1.661899109 | 1.754382014 | 1.697799899 | 1.656509844 | 1.714910312 |
| hsa-miR-8082 | 1.834234472 | 1.829105344 | 1.771442857 | 1.657260106 | 1.695123155 | 1.661008754 |
| hsa-miR-8083 | 1.85050126 | 1.744039349 | 1.728175919 | 1.930781737 | 1.62501675 | 1.693623444 |
| hsa-miR-8084 | 1.853327466 | 1.782464405 | 1.811197674 | 1.771314706 | 2.723899865 | 1.831963109 |
| hsa-miR-8085 | 1.920842487 | 1.711654591 | 1.628916504 | 1.821043301 | 1.724149152 | 1.715864384 |
| hsa-miR-8086 | 1.646077932 | 1.773560957 | 2.026405904 | 1.67716465 | 1.987047659 | 1.879594965 |
| hsa-miR-8087 | 2.666551402 | 1.8088832 | 2.028275335 | 4.206859906 | 1.729796091 | 3.251228595 |
| hsa-miR-8088 | 1.779577173 | 1.657836371 | 1.796573155 | 2.45793819 | 1.890278539 | 2.008304917 |
| hsa-miR-8089 | 4.19008358 | 2.242512988 | 3.078000616 | 3.853910021 | 1.936337358 | 3.042526005 |
| hsa-miR-8485 | 5.926653535 | 10.03070869 | 8.416789985 | 4.538922803 | 8.408525513 | 8.712948215 |
| hsa-miR-873-3p | 1.759666672 | 1.714354314 | 1.653259465 | 2.136837504 | 1.792356914 | 1.757311109 |
| hsa-miR-873-5p | 1.826070831 | 1.691452427 | 1.85688456 | 1.729532082 | 1.710309935 | 1.734061332 |
| hsa-miR-874-3p | 4.462009431 | 2.976489169 | 3.120851459 | 6.053204152 | 1.819000144 | 4.604216056 |
| hsa-miR-874-5p | 1.628274165 | 1.790415763 | 1.895062441 | 1.583252515 | 1.84803161 | 1.721850496 |
| hsa-miR-875-3p | 1.643859401 | 2.073611712 | 1.741888876 | 1.609517615 | 1.954933252 | 1.916898351 |
| hsa-miR-875-5p | 1.838140147 | 1.895551981 | 1.809148048 | 1.688828955 | 3.248924654 | 1.811349509 |
| hsa-miR-876-3p | 1.739212464 | 1.889892433 | 2.122068876 | 1.715154157 | 2.189262313 | 1.782806567 |
| hsa-miR-876-5p | 1.795302487 | 1.734121095 | 1.833107107 | 1.611774084 | 2.275762291 | 1.933477792 |
| hsa-miR-877-5p | 2.912274273 | 1.795797949 | 1.855794468 | 3.606284884 | 1.648731083 | 1.85385698 |
| hsa-miR-885-3p | 1.74891508 | 1.710351319 | 1.699470711 | 1.759532705 | 1.640834495 | 1.688397602 |
| hsa-miR-885-5p | 1.762424018 | 1.745363227 | 1.795894819 | 2.418771234 | 1.864267415 | 1.78857027 |
| hsa-miR-887-3p | 1.816196851 | 1.675514573 | 1.649457514 | 3.231549975 | 1.739533801 | 1.687925276 |
| hsa-miR-887-5p | 1.677031569 | 1.733628123 | 1.687806441 | 1.723871118 | 1.721074753 | 1.635197497 |
| hsa-miR-888-3p | 1.705478611 | 1.71160783 | 2.068878492 | 1.630113582 | 1.876434767 | 1.803332882 |
| hsa-miR-888-5p | 1.699938451 | 1.781500843 | 1.708370811 | 1.723328909 | 1.793860877 | 1.741496624 |
| hsa-miR-889-3p | 1.882089408 | 1.720254519 | 2.020538006 | 1.794484847 | 1.771727353 | 1.721517317 |
| hsa-miR-889-5p | 1.655021218 | 2.675940492 | 1.860404921 | 1.62189327 | 1.798272605 | 1.802090621 |
| hsa-miR-890 | 1.658241748 | 1.778228911 | 2.157566832 | 1.72762433 | 1.763028776 | 1.848580515 |
| hsa-miR-891a-3p | 1.700396259 | 1.862378162 | 1.817707761 | 1.719263537 | 2.673926497 | 2.023341604 |
| hsa-miR-891a-5p | 1.718835829 | 1.646645535 | 1.632025507 | 1.655266464 | 1.687442253 | 1.621463251 |
| hsa-miR-891b | 1.778151549 | 1.695086284 | 1.719346846 | 1.765898329 | 1.832541335 | 1.711326528 |
| hsa-miR-892a | 1.900601431 | 1.705660309 | 1.744218801 | 1.762842812 | 1.855459899 | 1.723632816 |
| hsa-miR-892b | 1.767744533 | 1.745659984 | 1.7349056 | 1.687619596 | 1.768898871 | 1.67884682 |
| hsa-miR-892c-3p | 1.688227327 | 2.030719517 | 1.812827224 | 1.667312717 | 2.896753697 | 2.062050589 |
| hsa-miR-892c-5p | 1.727461778 | 1.684989401 | 1.770865781 | 1.605139132 | 1.88733891 | 1.722665889 |
| hsa-miR-9-3p | 1.651108169 | 1.79144609 | 1.880933264 | 2.380139317 | 1.838187568 | 1.784165463 |
| hsa-miR-9-5p | 1.847449657 | 1.852212306 | 2.049005743 | 1.771324239 | 2.287518693 | 1.912881183 |
| hsa-miR-920 | 1.758570305 | 1.651850746 | 1.735984123 | 1.629838332 | 1.822941391 | 1.720202083 |
| hsa-miR-921 | 1.861470243 | 1.700945807 | 1.767651471 | 1.811842578 | 1.970379348 | 1.680837723 |
| hsa-miR-922 | 1.756452344 | 1.626250328 | 1.682395415 | 1.618241539 | 1.689272265 | 1.598778156 |
| hsa-miR-924 | 1.857281105 | 1.735051765 | 1.90806253 | 1.709643421 | 1.920204538 | 1.766389052 |
| hsa-miR-92a-1-5p | 1.620821511 | 1.95084617 | 1.758513798 | 1.668629319 | 1.97166997 | 1.76909274 |
| hsa-miR-92a-2-5p | 1.758036812 | 1.702344507 | 1.663620872 | 1.680365081 | 1.787813586 | 1.795566653 |
| hsa-miR-92a-3p | 9.652035798 | 11.58055766 | 11.7370176 | 9.898841289 | 8.30127693 | 11.31538065 |
| hsa-miR-92b-3p | 2.361720868 | 1.918412075 | 1.810636338 | 2.123870919 | 1.663121938 | 2.037479467 |
| hsa-miR-92b-5p | 1.597250044 | 1.806703708 | 1.711487964 | 1.663786351 | 1.752756204 | 1.720948591 |
| hsa-miR-93-3p | 1.715422259 | 1.78105172 | 1.726087355 | 1.707349364 | 1.765816505 | 1.608258061 |
| hsa-miR-93-5p | 7.77667807 | 4.425614664 | 6.297495573 | 7.62807409 | 1.803872713 | 7.691647928 |
| hsa-miR-933 | 1.900239136 | 3.434461203 | 2.612862348 | 2.061977031 | 2.385937114 | 1.994758343 |
| hsa-miR-934 | 1.713556013 | 1.819161018 | 1.760253409 | 2.915496686 | 1.699287285 | 1.790661317 |
| hsa-miR-935 | 1.748577241 | 1.76099262 | 1.736545812 | 1.671966223 | 1.780788527 | 1.756291136 |
| hsa-miR-936 | 2.658933458 | 2.259648084 | 2.180348601 | 3.423800086 | 1.714608887 | 2.25921743 |
| hsa-miR-937-3p | 1.680113474 | 2.25474201 | 1.636723339 | 1.72385969 | 1.776355137 | 1.687947882 |
| hsa-miR-937-5p | 5.395057931 | 3.613401364 | 5.598460408 | 5.934842142 | 4.133007815 | 4.212800782 |
| hsa-miR-938 | 1.768750472 | 1.679543235 | 1.753988459 | 1.643177625 | 1.758393122 | 1.774028693 |
| hsa-miR-939-3p | 1.817427592 | 1.781092442 | 1.778812097 | 1.782930001 | 1.918990592 | 1.764160011 |
| hsa-miR-939-5p | 5.547857157 | 4.094835227 | 4.407992007 | 4.210773506 | 1.717919924 | 5.365209772 |
| hsa-miR-940 | 8.532357641 | 9.529519786 | 9.471765538 | 6.149272111 | 11.61280471 | 8.55184387 |
| hsa-miR-941 | 1.73508073 | 1.68691105 | 1.643948127 | 1.648502647 | 1.626522097 | 1.578437062 |
| hsa-miR-942-3p | 1.837013122 | 1.783130213 | 1.814511142 | 2.9203076 | 2.016077258 | 1.803188406 |
| hsa-miR-942-5p | 1.824836432 | 1.717008426 | 1.922677892 | 1.758650335 | 2.09623459 | 1.900234447 |
| hsa-miR-943 | 1.690378053 | 1.700982904 | 1.790290867 | 1.642735928 | 1.733312465 | 1.746495452 |
| hsa-miR-944 | 1.68722493 | 1.754442422 | 1.838759247 | 1.727658467 | 1.989030314 | 1.770142058 |
| hsa-miR-95-3p | 1.85130287 | 1.830868173 | 1.905865828 | 1.678586524 | 2.218593237 | 1.941977341 |
| hsa-miR-95-5p | 2.119154358 | 2.128339599 | 1.719337343 | 1.92112736 | 2.224723342 | 1.756969132 |
| hsa-miR-9500 | 2.061085546 | 1.684232719 | 1.663119357 | 1.586822285 | 1.744739361 | 1.682435709 |
| hsa-miR-96-3p | 1.616744957 | 1.717485692 | 1.863393044 | 1.653202049 | 1.704753268 | 1.743205001 |
| hsa-miR-96-5p | 3.056300983 | 1.762325779 | 1.716762304 | 1.874188843 | 1.889518616 | 1.90783087 |
| hsa-miR-98-3p | 1.946296758 | 2.306397138 | 2.307469231 | 1.740538292 | 2.819983033 | 2.060454765 |
| hsa-miR-98-5p | 3.099100738 | 2.345695647 | 2.041877889 | 2.785219676 | 3.26321737 | 2.343996801 |
| hsa-miR-99a-3p | 1.647855214 | 1.721742726 | 1.709812154 | 1.773283429 | 1.797951863 | 1.728602317 |
| hsa-miR-99a-5p | 3.156791131 | 2.091384672 | 1.836748527 | 5.409574551 | 2.003626892 | 6.304087122 |
| hsa-miR-99b-3p | 2.078331606 | 1.9959733 | 1.993116901 | 2.034535013 | 1.719782859 | 1.790526322 |
| hsa-miR-99b-5p | 6.816441846 | 1.903965365 | 1.861181041 | 3.948506441 | 1.662945817 | 1.765643407 |

Table S5 The microRNA can promote islet β cell apoptosis in T lymphocyte extracellular vesicles in GSE27997

( microRNAs marked in red on yellow background are determined by expression values for subsequent analysis)

| microRNA |  | GSM692621 | GSM692622 | GSM692630 | average |
| --- | --- | --- | --- | --- | --- |
| hsa-miR-21 |  | 4.646875662 | 8.378165264 | 5.556236501 | 6.193759142 |
| hsa-miR-29a |  | 3.853615518 | 6.540670162 | 4.089844211 | 4.828043297 |
| hsa-miR-29b |  | 2.736928387 | 6.486188033 | 4.449093944 | 4.557403455 |
| hsa-miR-29c |  | 4.40948989 | 8.378573333 | 6.682470992 | 6.490178072 |
| hsa-miR-155 |  | 3.405051861 | 6.604461812 | 4.496757547 | 4.83542374 |
| hsa-miR-186 |  | 2.210951403 | 4.461487223 | 3.479696006 | 3.384044877 |
| hsa-miR-345 |  | -2.387310297 | 2.838626109 | 2.246875928 | 0.899397247 |
| hsa-miR-425* |  | 4.25405707 | 3.585879969 | 5.336815659 | 4.392250899 |
| hsa-miR-142-3p |  | 4.500539674 | 9.497821839 | 6.210976205 | 6.736445906 |
| hsa-miR-142-5p |  | 1.603136114 | 6.383760521 | 5.13602866 | 4.374308432 |

Table S6 The microRNA can promote islet β cell apoptosis in B lymphocyte extracellular vesicles in GSE27997

(microRNAs marked in red on yellow background are determined by expression levels for subsequent analysis)

| microRNA |  | GSM692617 | GSM692618 | GSM692629 | average |
| --- | --- | --- | --- | --- | --- |
| hsa-miR-21 |  | 9.895565058 | 11.99070527 | 9.58249374 | 10.48958802 |
| hsa-miR-29a |  | 7.713224779 | 9.130349647 | 6.455721755 | 7.76643206 |
| hsa-miR-29b |  | 6.126816927 | 7.169402685 | 5.004304188 | 6.1001746 |
| hsa-miR-29c |  | 5.175764189 | 6.907274575 | 4.662006654 | 5.581681806 |
| hsa-miR-155 |  | 7.401324408 | 9.376834142 | 7.09736436 | 7.958507637 |
| hsa-miR-146a |  | 5.798617026 | 8.463204808 | 6.338376418 | 6.866732751 |
| hsa-miR-10b* |  | 3.715784981 | 6.472776822 | 2.355491092 | 4.181350965 |
| hsa-miR-186 |  | 3.044156036 | 5.065856422 | 3.870623679 | 3.993545379 |
| hsa-miR-345 |  | 2.904356114 | 3.018009716 | 2.234330521 | 2.718898784 |
| hsa-miR-425* |  | 3.911558459 | 3.658056477 | 4.914396302 | 4.161337079 |
| hsa-miR-432 |  | 1.553752571 | 2.449983893 | 2.675020562 | 2.226252342 |
| hsa-miR-629* |  | 3.600475939 | 3.757109196 | 2.215076461 | 3.190887199 |
| hsa-miR-142-3p |  | 6.999211812 | 8.596823753 | 6.24595696 | 7.280664175 |
| hsa-miR-142-5p |  | 3.665924528 | 6.365604825 | 4.947706291 | 4.993078548 |

Table S7 The microRNA can promote islet β cell apoptosis in dendritic cells extracellular vesicles in GSE27997

(microRNAs marked in red on yellow background are determined by expression levels for subsequent analysis)

| microRNA |  | GSM692625 | GSM692626 | average |
| --- | --- | --- | --- | --- |
| hsa-miR-142-5p |  | 2.826347079 | 3.776092927 | 3.301220003 |
| hsa-miR-142-3p |  | 4.532555636 | 4.916091737 | 4.724323687 |
| hsa-miR-21 |  | 9.195723059 | 9.586470134 | 9.391096597 |
| hsa-miR-34a |  | 1.581492089 | 1.564747827 | 1.573119958 |
| hsa-miR-29a |  | 3.172499153 | 2.952378496 | 3.062438825 |
| hsa-miR-29b |  | 2.450543565 | 2.978517359 | 2.714530462 |
| hsa-miR-29c |  | 1.858087274 | 2.133497168 | 1.995792221 |
| hsa-miR-155 |  | 2.84372139 | 3.230384078 | 3.037052734 |
| hsa-miR-146a |  | 2.956541876 | 2.838158811 | 2.897350344 |
| hsa-miR-10b* |  | -1.718992825 | 2.083368602 | 0.182187889 |
| hsa-miR-186 |  | 1.822098497 | 1.768570449 | 1.795334473 |
| hsa-miR-425* |  | 5.403015778 | 5.864416767 | 5.633716273 |

Table S8 The microRNA can promote islet β cell apoptosis in pro-inflammatory macrophage extracellular vesicles in GSE137637

(1.microRNAs marked in red on yellow background are determined by expression levels for subsequent analysis 2 GSM4083583、GSM4083584、GSM4083585 are the control group, the others are the experimental group)

| microRNA | GSM4083583 | GSM4083584 | GSM4083585 | GSM4083590 | GSM4083591 | GSM4083592 | average |
| --- | --- | --- | --- | --- | --- | --- | --- |
| hsa-miR-21-5p | 10.80543694 | 7.71589535 | 8.875328017 | 11.51434162 | 9.499387474 | 12.13710393 | 10.09124889 |
| hsa-miR-34a-5p | 2.581060699 | 1.721929728 | 1.825174662 | 8.897247424 | 2.116640871 | 10.82385506 | 4.660984741 |
| hsa-miR-29a-3p | 9.172587026 | 3.055462357 | 3.759248881 | 8.712283336 | 5.658593322 | 10.32793131 | 6.781017705 |
| hsa-miR-29b-3p | 5.667269201 | 1.876210597 | 2.77192366 | 4.209595993 | 2.310570658 | 5.48998278 | 3.720925482 |
| hsa-miR-29c-3p | 6.053975381 | 1.895553335 | 2.652474097 | 7.743111576 | 3.007891189 | 10.31517934 | 5.27803082 |
| hsa-miR-142-3p | 2.268129977 | 2.927775719 | 2.254799714 | 3.1438144 | 2.401104373 | 5.247530853 | 3.040525839 |
| hsa-miR-142-5p | 1.646601946 | 1.780373367 | 1.760231608 | 3.184070149 | 1.992686124 | 1.921273445 | 2.04753944 |
| hsa-miR-155-5p | 6.931215413 | 6.459838957 | 5.853746214 | 5.877624308 | 1.853742345 | 2.666609126 | 4.940462727 |
| hsa-miR-146a-5p | 2.705359347 | 5.301722062 | 6.721904321 | 9.565863076 | 4.76892167 | 10.88900798 | 6.658796409 |
| hsa-miR-10b-3p | 1.75597864 | 1.803169104 | 1.95862438 | 2.039351748 | 2.071767462 | 2.469814941 | 2.016451046 |
| hsa-miR-186-5p | 3.150630468 | 2.690873108 | 2.4576291 | 5.242870006 | 2.370736619 | 6.149566415 | 3.677050953 |
| hsa-miR-345-5p | 2.087651543 | 1.716034139 | 1.864672068 | 2.051279496 | 1.804862623 | 1.745238921 | 1.878289798 |
| hsa-miR-376c-3p | 1.663265525 | 1.698075612 | 1.732167208 | 1.663687629 | 1.860411013 | 1.988915907 | 1.767753816 |
| hsa-miR-425-3p | 4.711629411 | 4.738605011 | 5.785303114 | 3.405482253 | 8.218575683 | 5.95689001 | 5.469414247 |
| hsa-miR-432-5p | 1.78721239 | 1.757879402 | 1.766945993 | 3.706075982 | 1.768910172 | 4.695463683 | 2.580414604 |
| hsa-miR-493-3p | 1.656379345 | 1.791357849 | 1.752486545 | 1.729184355 | 2.177836591 | 1.863102796 | 1.828391247 |
| hsa-miR-629-3p | 1.705165782 | 1.818555557 | 1.83694867 | 1.803429995 | 2.059250766 | 1.643717445 | 1.811178036 |
